# Supplementary material for: Location-agnostic site-specific protein bioconjugation via Baylis Hillman adducts
Source: Nat Commun. 2024 Jan 29;15:859. doi: 10.1038/s41467-024-45124-2 (PMC10825175; doi:10.1038/s41467-024-45124-2)

Supplementary Information for

Location-agnostic site-specific protein bioconjugation via Baylis Hillman adducts

Mudassir H. Mir[1]†, Sangeeta Parmar[1]†, Chhaya Singh[1], Dimpy Kalia[1]*

[1]Department of Chemistry, Indian Institute of Science Education and Research (IISER) Bhopal, Bhopal Bypass Road, Bhauri, Bhopal–462066, Madhya Pradesh, India

†These authors contributed equally to this work.

*Corresponding author. Email: dimpy@iiserb.ac.in

The PDF file includes:

Materials and Methods

Supplementary Figs. 1 to 92

Supplementary Tables 1 to 16

NMR spectra

Supplementary References (1–20)

Table of Contents			
**Section No.**	**Contents**	**Page No.**	**Supplementary Figures and Tables**

Section No.	Contents	Page No.	Supplementary Figures and Tables
1	Materials and Methods	8–10	-
2	Synthesis and characterization of IBH adducts and their cysteamine/cysteine conjugates	11–25	Figs. 1–4
3	Synthesis and characterization of thiazolidine-appended lipoic acid analogs	26–35	Figs. 5–8
4	Kinetic studies of the conjugation reactions of IBH adducts with cysteamine	35–45	Figs. 9–13
5	Stability studies of the bis-heterocyclic IBH-cysteamine conjugates	46–48	Figs. 14–16
6	Recombinant production of *N*-Cys POI and His-TEV protease	49–55	Figs. 17, 18, Tables 1–4
7	Labelling of *N*-Cys-POI via BHoPAL and the ESI-MS analysis of resultant protein conjugates	55–76	Figs. 19–28, Table 5–7
8	Fluorescent labelling of *N*-Cys-MBP via BHoPAL in a protein mixture	76–78	Fig. 29
9	Fluorescent labelling of *N*-Cys of EGFR-eGFP fusion protein via BHoPAL in HEK293 cells	79–80	Figs. 30, 31
10	Docking studies on lipoic acid ligase-lipoic acid analogs interactions	81–84	Fig. 32, Table 8
11	Recombinant production of LplA^{W37V}	84–85	Fig. 33
12	HPLC analysis of ligation reaction of thiazolidine-appended lipoic acid analogs on to the LAP peptide	86–89	Fig. 34, Table 9
13	Recombinant production of MBP-LAP protein	90–92	Figs. 35, 36, Tables 10–11
14	Procedure for incorporating the 1,2-aminothiol moiety within the MBP-LAP protein followed by its modification via BHoPAL	93–99	Figs. 37–39, Table 12
15	Recombinant production of TEV(C)-MBP-LAP protein and its cleavage with TEV protease	100–103	Fig. 40, 41, Table 13, 14

16	Concomitant and tandem BHoPAL for dual labelling of proteins	103–110	Fig. 42,<br>Table 15, 16
17	Binding assays of mono and dually labelled MBP conjugates	110–111	Fig. 43
18	NMR spectral data	112–178	Figs. 44–92
19	References	179–180	-
20	Abbreviations	180	-
21	Raw images of the gels reported in the Supplementary Figures.	181–184	-

Supplementary Figures and Tables

Figures and Table No.	Title	Page No.
Fig. 1	Synthesis of IBH adducts **1A**–**G**	11
Fig. 2	Synthesis of 3-(butynyloxyl)propanoyl chloride (**S6**)	14
Fig. 3	Synthesis of C=C linked bis-heterocycles (**2A**–**C** and **S2A-Cys**) via IBH adduct-mediated 1,2-aminothiol derivatization	21
Fig. 4	ORTEP diagram of IBH-cysteamine conjugates **2A** and **2C** with thermal ellipsoids at the 50% probability level	24
Fig. 5	Synthesis of N-boc pentafluorophenyl thiazolidine-4-carboxylate (**S8**)	26
Fig. 6	Synthesis of tert-butyl ester intermediates of amino acids (**S15**–**17**)	27
Fig. 7	Synthesis of C3Tz and C7Tz lipoic acid analogs	30
Fig. 8	Synthesis of C2Tz, C4Tz and C5Tz lipoic acid analogs	32
Fig. 9	Conjugation reaction of IBH adducts with cysteamine in the presence NAC at different pH	37
Fig. 10	Conjugation reaction of IBH adducts with cysteamine in the presence of different amino acids	39
Fig. 11	Conjugation reaction of IBH adduct 1A with cysteamine in the presence thiols at different pH	40
Fig. 12	Second-order reaction kinetics between IBH adducts and cysteamine	43
Fig. 13	Comparison of the reactions of IBH, CPO and CBT 1,2-aminothiol-derivatization reagents with cysteamine	45
Fig. 14	Stability of conjugation products at different pH	47
Fig. 15	Stability of conjugation products in presence of cysteine (Cys)	48
Fig. 16	Stability of conjugation products in the presence of glutathione (GSH)	48

Fig. 17	Sequence analysis of plasmid constructs	51
Fig. 18	SDS-PAGE analysis of purified His-tagged and TEV-cleaved proteins	55
Fig. 19	ESI-MS spectra of *N*-Cys-POI conjugates generated via BHoPAL	58
Fig. 20	Labelling of *N*-Cys-eGFP with *N*-acyl IBH adduct at low time points	64
Fig. 21	Optimization of bioconjugation conditions for labelling *N*-Cys-POI with *N*-alkyl IBH adducts	65
Fig. 22	Selective labelling of *N*-Cys residue of proteins via BHoPAL	66
Fig. 23	LC-MS/MS analyses of trypsin digested IBH-labelled *N*-Cys protein conjugates	68
Fig. 24	Labelling of *N*-Cys-POI at lower protein concentration via BHoPAL	71
Fig. 25	Labelling of *N*-Cys-POI with *N*-acyl IBH at low temperatures	72
Fig. 26	Stability of *N*-Cys-IBH protein conjugates in presence of glutathione (GSH)	73
Fig. 27	Structural integrity of IBH-POI conjugates	75
Fig. 28	Retention of intrinsic fluorescence properties in the IBH conjugates of fluorescent proteins	76
Fig. 29	Selective labelling of *N*-Cys-MBP via BHoPAL in a protein pool	78
Fig. 30	Fluorescent labelling of the *N*-Cys GFP-EGFR fusion protein on the surface of HEK293 cells	80
Fig. 31	No fluorescent labelling of the *N*-Cys GFP-EGFR upon treatment with fluorescent azide	80
Fig. 32	Molecular docking of thiazolidine-appended lipoic acid and their AMP complex to LplA	82
Fig. 33	SDS-PAGE analysis of purified His-tagged LplAW37V.	85

Fig. 34	HPLC traces for the screening of LplA^{W37V} mediated ligation of LA and thiazolidine-appended LA analogs (C2–C7Tz) on the LAP peptide	88
Fig. 35	Sequence analysis of LAP tag inserted MBP construct generated via three PCR steps	91
Fig. 36	SDS-PAGE analysis of purified MBP-LAP	92
Fig. 37	Labelling of MBP-LAP protein	95
Fig. 38	LC-MS/MS analyses of chymotrypsin-digested MBP-LAP C7-**1F** conjugate	99
Fig. 39	Isolated yields of a three-step protocol involved in generating C7-**1D** on a milligram scale	99
Fig. 40	Sequence analysis of LAP-tag inserted TEV-MBP construct generated via three PCR steps	101
Fig. 41	SDS-PAGE analysis of purified His-TEV(C)-MBP-LAP and TEV-cleaved protein	102
Fig. 42	Concomitant and tandem dual labelling of protein via BHoPAL	106
Fig. 43	Binding studies on MBP-LAP conjugates	111

Table 1	Sequence of primers employed for cloning	49
Table 2	Summary of protein expression conditions	52
Table 3	Sequence of the expressed recombinant His-tagged proteins employed for TEV cleavage reactions	54
Table 4	Sequence of TEV cleaved proteins employed for *N*-(Cys) bioconjugation.	54
Table 5	Theoretical and observed molecular masses of the unmodified *N*-Cys-POI and their desired cyclic *N*-Cys-IBH conjugates	57
Table 6	Labelling of *N*-Cys proteins with *N*-alkyl IBH adducts.	58
Table 7	Sequence of labelled *N*-Cys protein bioconjugates with their trypsin cleavage sites	67

Table 8	Binding energies (ΔG) of LA analogs C2–C7Tz and their AMP adducts complexed with LplA^{W37V}	84
Table 9	Theoretical and observed masses (HRMS) of LAP-conjugates	89
Table 10	Sequence of primers employed for molecular cloning	90
Table 11	Sequence of expressed recombinant His-MBP-LAP used for bioconjugation	92
Table 12	Theoretical and observed molecular masses of the unmodified and modified MBP-LAP	94
Table 13	Sequence of primers employed for cloning to generate His-TEV(C)-MBP-LAP construct	100
Table 14	Sequence of recombinant His-TEV(C)-MBP-LAP and TEV cleaved *N*-Cys-MBP-LAP proteins	103
Table 15	Theoretical and observed molecular masses of the TEV-MBP-LAP and *N*-Cys-MBP-LAP conjugates	106
Table 16	Reaction conditions screened for one-step mono modification of *N*-Cys-C7AT with IBH **1E**	110

1. Materials and Methods.

All the reactions were performed in oven-dried glassware under an inert atmosphere of N_2 or Ar. Room temperature (RT) refers to 25 °C. All reagents employed for the synthesis were purchased from commercial suppliers (Sigma-Aldrich, Spectrochem, Hi-media, Alfa Aesar and SDFCL) and were used as received without further purification. LAP peptide was custom synthesized from 'S' BioChem, Kerala-India. 2-cyanobenzothioazole (CBT) was purchased from BLD Pharmatech. Distilled water was used for reaction work-ups, and ultrapure Type 1 water (Milli-Q water of resistivity 18.2 MΩ.cm at 25 °C) was used for buffer preparations and LC-MS experiments. Degassed buffers were used for *in vitro* protein bioconjugation. Anhydrous dichloromethane (CH_2Cl_2), acetonitrile (CH_3CN), *N,N*-diisopropylethylamine (DIPEA), and triethylamine (Et_3N) were obtained by distillation over CaH_2 under N_2 atmosphere. Anhydrous tetrahydrofuran (THF) was prepared by distillation from sodium/benzophenone ketyl. All the reactions were monitored by thin-layer chromatography (TLC) using Merck silica gel 60 F_{254} precoated plates (0.25 mm thickness). TLC plates were visualized by exposure to either short (254 nm) or long (365 nm) wave ultraviolet (UV) light. The spots of the UV inactive compounds on the TLC plates were visualized by dipping these plates in staining solutions including phosphomolybdic acid (PMA), ninhydrin and $KMnO_4$, followed by exposing stained TLC plates to the hot air gun in the fume hood. Silica gel of 100–200 mesh was employed for column chromatography. Fluorescent molecules were synthesized under minimal exposure to light.

Nuclear magnetic resonance (NMR) spectra (^1H, ^{13}C, DEPT-135, and ^{19}F) were recorded on Bruker 400, 500 and 700 MHz spectrometers in deuterated solvents using tetramethyl silane (TMS) as internal standard at 298K. The NMR spectra of molecules existing as rotamers at RT were recorded at 353K in DMSO-d6. Chemical shifts are reported in parts per million (δ), and coupling constants (*J* values) are reported in Hertz (Hz). Multiplicities are denoted as follows: s (singlet), d (doublet), br s (broad singlet) br d (broad doublet) t (triplet), q (quartet), dd (doublet of doubles), ddd (doublet of doublet of doublets), dt (doublet of triplets), td (triplet of doublets), dq (doublet of quartet) and m (multiplet). High-resolution mass spectra (HRMS) for the synthesized molecules and the LAP peptide conjugates were recorded on a Bruker Daltonics MicroTOF equipped with electron spray ionization (ESI). Mass characterization of protein conjugates was performed on 6546 LC/Q-TOF coupled with 1290 Infinity II LC System and a triple quadrupole mass spectrometer (QTRAP® 4500 system from AB SCIEX) equipped with an Exion LC, using

Milli-Q water/acetonitrile mobile phase containing 0.1% formic acid. The extracted ion chromatograms (XIC) of protein conjugates were deconvoluted using Sciex PeakView software. Single crystal X-ray diffraction measurements were recorded on Brüker APEX-II CCD diffractometer at 140K. Lyophilization of aqueous solutions were carried out on Labconco freeze dryer. Kinetic studies were performed on Agilent's Carry 3000 UV-Visible spectrophotometer with a quartz cuvette (1 mL with a 10 mm path length). Hydrogenation reactions were performed in parr reactor at 50 psi hydrogen gas pressure.

The HPLC studies were performed on Agilent 1260 Infinity II instrument using reverse-phase column chromatography. The analytical columns, C18-Zorbax ODS (5 μm, 4.6 × 250 mm) and Zorbax 300SB-C18 stable bond (5 μm, 4.6 × 250 mm) were used for screening the reactions of IBH adducts and the LAP peptide, respectively. The mobile phase employed for the HPLC was acetonitrile-water containing 0.1% trifluoroacetic acid (TFA); solvent elution profile employed: 0–5 min elution with 100% water, followed by linear increase to 100% acetonitrile over 30 min. The flow rate was 1 mL/min throughout the HPLC run except for monitoring reactions performed at micromolar concentrations. These reaction mixtures were injected to the HPLC with an initial flow rate of 1.5 mL/min that was gradually decreased to 1 mL/min over 5 min and then kept constant throughout a 30 min run. UV absorbance was monitored at 215 and 254 nm.

Polymerase chain reactions (PCRs) were performed on Nexus gradient thermocycler from Eppendorf. Nanodrop 2000 spectrophotometer from Thermo-scientific was used to measure A280 nm for measuring protein concentrations. Images of protein gels (fluorescence and Coomassie blue staining gels) were captured on Gel Doc System from Analytik Jena. Fluorescence emission spectra of eGFP and mCherry protein conjugates were recorded on Flurolog-3 from HORIBA Jobin Yvon using quartz cuvette (500 μL with a 5 mm path length). Circular dichroism (CD) measurements were recorded in Milli-Q water at RT on JASCO J-815 CD spectropolarimeter using a quartz cuvette (300 μL) with a 1 mm path length.

LC-MS/MS data for digested protein conjugates were acquired using an Agilent 6546 HRLC-QTOF ESI mass spectrometer and analyzed using the Agilent Mass Hunter Bioconfirm Software 11.0. All the primers employed for cloning and sequence analyses were procured from either IDT or Sigma. Sequencing of plasmid constructs were performed at the inhouse sequencing facility at IISER Bhopal, and the sequencing data was analyzed with either Snapgene or Clustal

Omega sequence alignment tools. Molecular weights of proteins and their molar extinction coefficient at 280 nm were determined using ExPASy ProtParam tool.

HEK 293 cells were cultured in Eppendorf Galaxy 170S CO_2 incubator maintained at 37 °C and 5% CO_2. Cell culture consumables such DMEM, FBS, antibiotics, trypsin-EDTA and lipofectamine were purchased from Invitrogen and Thermo Fisher Scientific. Confocal imaging dishes (Cat. no. 100350) were purchased from SPL Life Sciences. Cells were imaged under Olympus FV3000 confocal laser-scanning microscope confocal microscope using a 60× oil objective in three different channels (GFP: λ_{ex} 488nm, λ_{em} 509 nm, Hoechst 33342: λ_{ex} 350 nm, λ_{em} 460 nm and Cy5: λ_{ex} 647 nm, λ_{em} 663 nm) and the images were processed using FV31S-SW software. We thank Prof. Oliver Seitz for his kind gift of the plasmid for expressing Cys-E3-EGFR-eGFP.

2. Synthesis and characterization of IBH adducts and their cysteamine/cysteine conjugates

Supplementary Fig. 1. Synthesis of IBH adducts **1A**–**G**

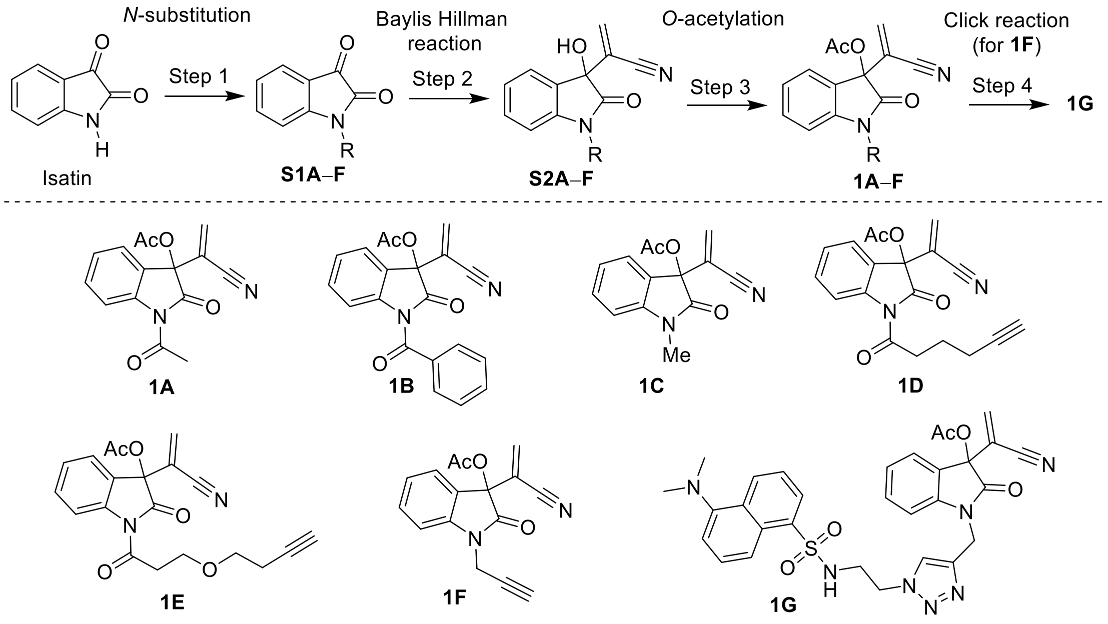

Step1: Synthesis of N-substituted isatin:

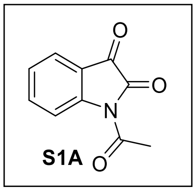
S1A

The compound **S1A** was synthesized according to the reported protocol in the literature[1]. **^{1}H NMR** (500 MHz, DMSO-d_6) δ 8.27 (d, *J* = 8.2 Hz, 1H), 7.79 – 7.75 (m, 2H), 7.37 (t, *J* = 7.5 Hz, 1H), 2.59 (s, 3H); **^{13}C NMR** (126 MHz, DMSO-d_6) δ 180.17, 169.81, 158.24, 147.91, 137.74, 125.50, 124.37, 119.86, 117.18, 26.00.

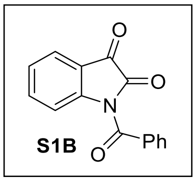
S1B

The compound **S1B** was synthesized according to the reported protocol in the literature[2]. **^{1}H NMR** (500 MHz, DMSO-d_6) δ 7.94 (d, *J* = 8.2 Hz, 1H), 7.92 – 7.88 (m, 2H), 7.80 (t, *J* = 7.5 Hz, 2H), 7.67 (t, *J* = 7.5 Hz, 1H), 7.52 (t, *J* = 7.7 Hz, 2H), 7.39 (t, *J* = 7.5 Hz, 1H); **^{13}C NMR** (126 MHz, DMSO-d_6) δ 180.18, 167.99, 157.12, 148.01, 137.47, 133.77, 133.01, 129.66, 128.07, 125.27, 124.43, 120.01, 116.11.

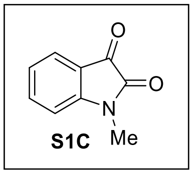
S1C

To a stirred solution of isatin (5 gm, 34 mmoL, 1 eq.) in DMF (40 mL) at 0 °C was added K_2CO_3 (6.5 gm, 47.6 mmoL, 1.5 eq.). After 30 min, methyl iodide (2.5 mL, 40.8 mmoL, 1.2 eq.) was added to the reaction mixture dropwise at 0 °C under N_2 atmosphere. The reaction mixture was allowed to warm to RT and stirred for an additional 12 h. Water (25 mL) was added, and the reaction mixture was

extracted with EtOAc (3 × 50 mL). The organic layers were combined and washed with brine (50 mL), dried (Na_2SO_4), and concentrated *in vacuo* on a rotary evaporator. The resultant crude was purified by column chromatography on silica gel using ethyl acetate/hexane eluent to give compound **S1C** as a red solid (4.3 gm, 78%, R_f 0.4 in hexane/EtOAc 1:1). **^{1}H NMR** (500 MHz, $CDCl_3$) δ 7.57 (t, *J* = 7.8 Hz, 1H), 7.52 (d, *J* = 7.4 Hz, 1H), 7.08 (t, *J* = 7.5 Hz, 1H), 6.87 (d, *J* = 7.9 Hz, 1H), 3.20 (s, 3H); **^{13}C NMR** (126 MHz, $CDCl_3$) δ 183.38, 158.22, 151.45, 138.52, 125.17, 123.84, 117.38, 110.05, 26.23. HRMS (QTOF MS ESI+) *m/z* calcd. for $C_9H_7NO_2$ $[M+Na]^+$ 184.0374; found 184.0369.

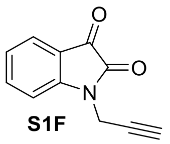

S1F

A similar procedure as described above for **S1C**, was followed using propargyl bromide (1.5 eq.) in place of methyl iodide. The reaction was monitored by TLC and found to be completed in 3 h. The crude of the reaction was subjected to column chromatography on silica gel using ethyl acetate/hexane eluent to give compound **S1F** as a red solid (0.182 gm, 29%, R_f 0.5 in hexane/EtOAc 1:1). **^{1}H NMR** (500 MHz, $CDCl_3$) δ 7.64 (td, *J* = 7.5, 1.3 Hz, 2H), 7.17 (t, *J* = 7.6 Hz, 1H), 7.13 (d, *J* = 8.0 Hz, 1H), 4.53 (s, 2H), 2.31 (s, 1H); **^{13}C NMR** (126 MHz, $CDCl_3$) δ 182.64, 157.27, 149.73, 138.55, 125.58, 124.32, 117.81, 111.21, 77.41, 77.16, 76.91, 75.80, 73.47, 29.58. **HRMS** (QTOF MS ESI+) *m/z* calcd. for $C_{11}H_7N_2O_2$ $[M+H]^+$ 186.0555, found 186.0550.

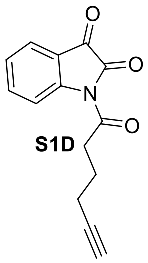

S1D

To a suspension of sodium hydride (0.372 mg, 15 mmoL, 1.2 eq.) in THF (15 mL) was added a solution of isatin (1.9 gm, 13 mmoL, 1 eq.) in THF (25 mL) dropwise at 0 °C under N_2 atmosphere. A dark purple colour appeared upon the addition of the isatin solution. The reaction was stirred for 30 min, and a solution of freshly prepared 5-hexynoyl chloride **S3** (1.7 gm, 1 3mmoL, 1 eq.), following the procedure described below, in THF (10 mL) was added dropwise to the reaction mixture at 0 ^{o}C. After complete addition, the purple colour disappeared, and the reaction mixture turned pale yellow. After 15 min of stirring, the reaction was allowed to warm to RT and stirred for an additional 17 h. The reaction was quenched with water (5 mL), and the THF was removed *in vacuo* using a rotary evaporator. The resultant crude was diluted with EtOAc (50 mL) and water (30 mL). The two layers were separated and the organic layer was washed with brine solution (30 mL), dried (Na_2SO_4), and concentrated *in vacuo.* The crude was purified by column chromatography on silica gel using ethyl acetate/hexane (3:7) eluent to

afford **S1D** as a yellow solid (1.414 gm, 45% yield, R_f 0.6 in EtOAc/hexane 3:7). **^{1}H NMR** (500 MHz, $CDCl_3$) δ 8.41 (d, J = 8.3 Hz, 1H), 7.77 (d, J = 7.5 Hz, 1H), 7.72 (t, J = 7.9 Hz, 1H), 7.33 (t, J = 7.5 Hz, 1H), 3.25 (t, J = 7.1 Hz, 2H), 2.36 (td, J = 6.9, 2.6 Hz, 2H), 2.05 – 1.95 (m, 3H). **^{13}C NMR** (126 MHz, $CDCl_3$) δ 180.24, 172.44, 157.95, 148.82, 139.04, 126.27, 125.45, 119.39, 118.41, 83.29, 69.59, 37.05, 22.88, 17.82. **HRMS** (QTOF MS ESI+) *m/z* calcd. for $C_{14}H_{11}NO_3$[M+Na] 264.0637, found 264.0631.

Synthesis of 5-hexynoyl chloride (**S3**)

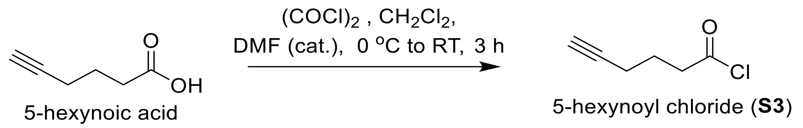

5-hexynoyl chloride (**S3**) was synthesized according to the reported protocol in the literature[3]. Briefly, to a stirred solution of 5-hexynoic acid (1.5 gm, 13 mmoL, 1 eq.) in anhydrous CH_2Cl_2 (20 mL) containing catalytic DMF (2 drops) was added oxalyl chloride (1.7 mL, 20 mmoL, 1.5 eq.) dropwise at 0 °C under N_2 atmosphere. After the addition, the reaction mixture was allowed to warm to RT and stirred for 3 h. The reaction mixture was concentrated thoroughly *in vacuo* on a rotary evaporator, and the resultant product **S3** obtained as pink solid was used immediately without any further purification for the synthesis of **S1D** as described above.

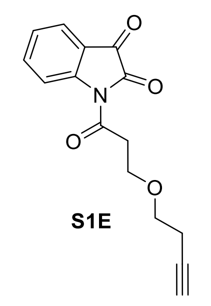

S1E was synthesized following a similar protocol as described above for the synthesis of **S1D** using isatin (0.342 gm, 2.3 mmoL, 1 eq.), NaH (67 mg, 2.8 mmoL, 1.2 eq.) and 3-(butynyloxyl)propanoyl chloride **S6** (374 mg, 2.3 mmoL, 1 eq.). **S6** was prepared in 3 steps following scheme described below (Scheme S2). For purification, the crude of the reaction obtained after work-up was subjected to column chromatography on silica gel using EtOAc/hexane (2:3) eluent to afford **S1E** as yellow solid (0.154 gm, 24%, R_f 0.6 in EtOAc/hexane 2:3). **^{1}H NMR** (500 MHz, $CDCl_3$) δ 8.41 (d, J = 8.3 Hz, 1H), 7.77 (d, J = 7.6 Hz, 1H), 7.71 (t, J = 7.2 Hz, 1H), 7.33 (t, J = 7.5 Hz, 1H), 3.90 (t, J = 6.0 Hz, 2H), 3.63 (t, J = 6.9 Hz, 2H), 3.40 (t, J = 6.0 Hz, 2H), 2.45 (td, J = 6.9, 2.6 Hz, 2H), 1.92 (t, J = 2.7 Hz, 1H). **^{13}C NMR** (126 MHz, $CDCl_3$) δ 180.21, 170.77, 157.98, 148.77, 139.10, 126.36, 125.48, 119.42, 118.50, 81.28, 69.51, 69.35, 65.44, 38.79, 19.91. **HRMS** (QTOF MS ESI+) *m/z* calcd. for $C_{15}H_{13}NO_4$ $[M+H]^+$ 272.0923, found 272.0917.

Supplementary Fig. 2. Synthesis of 3-(butynyloxyl)propanoyl chloride (**S6**)

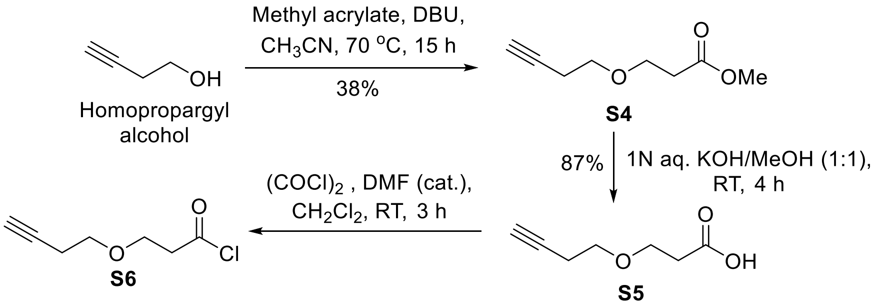

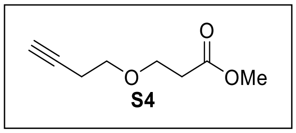

To a stirred mixture of homopropargyl alcohol (0.5 mL, 7.1 mmoL, 1 eq.) and methyl acrylate (1.3 mL, 14.2 mmoL, 2 eq.) in CH_3CN (25 mL) was added 1,8-Diazabicyclo (5.4.0) undec-7-ene (DBU) (1 mL, 7.1 mmoL, 1 eq.) at RT. The reaction mixture was heated to 70 °C for 15 h. The solvent was concentrated *in vacuo* on a rotary evaporator, and the resultant crude was diluted with EtOAc (30 mL) and water (20 mL). The two layers were separated and the aqueous layer was extracted with EtOAc (20 mL × 3). All the organic layers were combined and washed with brine solution (30 mL), dried over Na_2SO_4, and concentrated *in vacuo*. The resultant crude was purified by silica gel column chromatography on silica gel using hexane/EtOAc (7:3) eluent to afford **S4** as a colourless oil (0.424 gm, 38% yield, R_f 0.4 in hexane/EtOAc 4:1). TLC of the reaction was visualized using $KMnO_4$ staining solution. **^{1}H NMR** (500 MHz, $CDCl_3$) δ 3.74 (t, *J* = 6.4 Hz, 2H), 3.68 (s, 3H), 3.57 (t, *J* = 7.0 Hz, 2H), 2.59 (t, *J* = 6.4 Hz, 2H), 2.44 (td, *J* = 6.9, 2.6 Hz, 2H), 1.96 (t, *J* = 2.7 Hz, 1H). **^{13}C NMR** (126 MHz, $CDCl_3$) δ 172.06, 81.27, 69.43, 69.19, 66.37, 51.83, 34.95, 19.84. **HRMS** (QTOF MS ESI+) *m/z* calcd. for $C_8H_{12}O_3$ $[M+Na]^+$ 179.0684, found 179.0679.

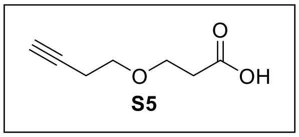

To a stirred solution of homopropargyl ester **S4** (0.415 gm, 2.6 mmoL, 1eq.) in MeOH (8 mL) was added 1N KOH solution in water (8 mL, 7.9 mmoL, 3 eq.) at RT. After 4 h, the reaction mixture was concentrated *in vacuo* on a rotary evaporator to remove MeOH. The resultant aqueous solution was further diluted with water (10 mL), and the pH of the mixture was adjusted to ~5–6 with 1N HCl solution in water. The acidic aqueous solution was extracted with EtOAc (50 mL × 3). The organic layers were combined, washed with brine solution (40 mL), dried over Na_2SO_4, and concentrated *in vacuo* to afford analytically pure **S5** as a colourless oil (0.331 gm, 87% yield, R_f 0.25 in CH_2Cl_2/MeOH 19:1). TLC of the reaction was visualized using $KMnO_4$ staining solution.

^{1}H NMR (500 MHz, $CDCl_3$) δ 3.76 (t, *J* = 6.3 Hz, 2H), 3.60 (t, *J* = 6.9 Hz, 2H), 2.64 (t, *J* = 6.3 Hz, 2H), 2.46 (td, *J* = 6.9, 2.7 Hz, 2H), 1.98 (t, *J* = 2.7 Hz, 1H). **^{13}C NMR** (126 MHz, $CDCl_3$) δ 177.47, 81.16, 69.59, 69.27, 66.04, 34.94, 19.83. **HRMS** (QTOF MS ESI+) *m/z* calcd. for $C_7H_{10}O_3$ $[M+Na]^+$ 165.0528, found 165.0522.

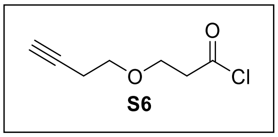

3-(butynyloxyl)propanoyl chloride **S6** was prepared following a similar procedure as described above for **S3** by employing acid **S5** (0.331 gm, 2.3 mmoL, 1eq.), oxalyl chloride (0.6 mL,6.9 mmoL, 3 eq.) and catalytic amounts of DMF. The crude product obtained in the reaction was used directly for the synthesis of **S1g** without any further purification.

Step2: Baylis Hillman reaction of N-substituted isatin with acrylonitrile

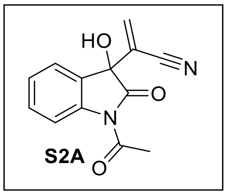

To a stirred solution of *N*-acyl isatin **S1A** (1 gm, 5.28 mmoL, 1 eq.) in anhydrous THF (15 mL) was added acrylonitrile (1.4 mL, 21.1 mmoL, 4 eq.) and DABCO (0.593 gm, 5.28 mmoL, 1 eq.) at RT under N_2 atmosphere. The progress of the reaction was monitored by TLC. After 3.5 h, the reaction mixture was concentrated *in vacuo* on a rotary evaporator to remove THF. The resultant crude was diluted with EtOAc (50 mL) and water (20 mL). The two layers were separated, and the organic layer was washed with brine (30 ml), dried (Na_2SO_4), and concentrated *in vacuo*. The crude was subjected to column chromatography on silica gel using EtOAc/hexane (1:1) eluent to afford **S2A** as an off-white solid (0.853 gm, 66%, R_f 0.46 in hexane/EtOAc 1:1).**^{1}H NMR** (500 MHz, CDCl3) δ 8.26 (d, *J* = 8.2 Hz, 1H), 7.48 (t, *J* = 7.9 Hz, 1H), 7.43 (d, *J* = 7.5 Hz, 1H), 7.32 (t, *J* = 7.5 Hz, 1H), 6.26 (s, 1H), 6.24 (s, 1H), 3.49 (s, 1H), 2.66 (s, 3H). **^{13}C NMR** (126 MHz, $CDCl_3$) δ 175.03, 170.40, 140.30, 132.44, 131.90, 126.54, 125.85, 124.65, 122.93, 117.48, 115.18, 76.51, 26.56. **HRMS** (QTOF MS ESI+) *m/z* calcd. for $C_{13}H_{10}N_2O_3[M+Na]^+$ 265.0589, found 265.0584.

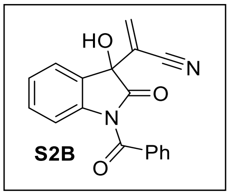

Compound **S2B** was synthesized following similar protocol as described above for the synthesis of **S2A** by employing **S1B** (1 gm, 3.97 mmoL, 1 eq.), acrylonitrile (1 mL, 15.91 mmoL, 4 eq.) and DABCO (0.223 gm, 1.99 mmoL, 0.5 eq.). The reaction was continued for 14 h at RT, and the crude of the reaction obtained after work up was purified using column chromatography on silica gel

using hexane/EtOAc (3:2) eluent to afford **S2B** as a dark red solid (0.161 gm, 13%, R_f 0.38 in hexane/EtOAc 3:2) **^{1}H NMR** (500 MHz, $CDCl_3$) δ 7.86 (d, *J* = 8.2 Hz, 1H), 7.80 (d, *J* = 7.5 Hz, 2H), 7.63 (t, *J* = 7.5 Hz, 1H), 7.54 – 7.44 (m, 4H), 7.33 (t, *J* = 7.2 Hz, 1H), 6.42 (s, 1H), 6.28 (s, 1H). **^{13}C NMR** (126 MHz, $CDCl_3$) δ 174.19, 168.45, 140.34, 133.59, 133.18, 131.86, 131.68, 129.44, 128.56, 126.10, 126.08, 124.72, 122.69, 115.82, 115.33, 76.86. **HRMS** (QTOF MS ESI+) *m/z* calcd. for $C_{18}H_{12}N_2O_3$ $[M+Na]^+$ 327.0746, found 327.0740.

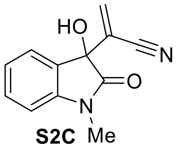

S2C

Following the procedure as described above for **S2A**, compound **S2C** was synthesized by employing **S1C** (1 gm, 6.2 mmoL, 1 eq.), acrylonitrile (1.62 mL, 24.8 mmoL, 4 eq.) and DABCO (0.696 gm, 6.2 mmol, 1 eq.). The reaction was continued for 6 h at RT, and the crude of the reaction obtained after work up was purified using column chromatography on silica gel using hexane/EtOAc (1:1) eluent to afford **S2C** as off-white coloured solid (1.2 gm, 91%, R_f 0.38 in hexane/EtOAc 1:1). **^{1}H NMR** (500 MHz, $CDCl_3$) δ 7.41 (ddt, *J* = 6.0, 4.7, 1.9 Hz, 2H), 7.17 (t, *J* = 7.6 Hz, 1H), 6.92 (d, *J* = 8.0 Hz, 1H), 6.35 (s, 1H), 6.17 (s, 1H), 4.20 (s, 1H), 3.25 (s, 3H). **^{13}C NMR** (126 MHz, $CDCl_3$) δ 174.37, 143.35, 131.46, 131.12, 127.16, 124.66, 124.10, 123.13, 115.57, 109.27, 76.56, 26.74. **HRMS** (QTOF MS ESI+) *m/z* calcd. for $C_{12}H_{10}N_2O_2$ $[M+Na]^+$ 237.0640; found 237.0634.

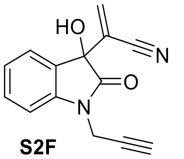

S2F

Following the procedure as described above for **S2A**, compound **S2F** was obtained as an off-white solid (0.196 gm, 84%, R_f 0.39 in hexane/EtOAc 3:2) from **S1F** (0.182 gm, 0.982 mmoL, 1 eq.), acrylonitrile (0.25 mL, 3.93 mmoL, 4 eq.) and DABCO (0.110 gm, 0.982 mmoL, 1 eq.). The reaction was continued for 14 h at RT, and the crude of the reaction obtained after work up was purified using column chromatography on silica gel using hexane/EtOAc (3:2) eluent. **^{1}H NMR** (500 MHz, $CDCl_3$) δ 7.45 (t, *J* = 7.3 Hz, 2H), 7.20 (t, *J* = 7.6 Hz, 1H), 7.13 (d, *J* = 8.0 Hz, 1H), 6.31 (s, 1H), 6.17 (s, 1H), 4.52 (d, *J* = 2.5 Hz, 2H), 3.85 (s, 1H), 2.29 (t, *J* = 2.5 Hz, 1H). **^{13}C NMR** (126 MHz, $CDCl_3$) δ 173.51, 141.60, 131.80, 131.29, 127.03, 124.91, 124.55, 123.01, 115.52, 110.45, 76.49, 75.91, 73.40, 30.02. **HRMS** (QTOF MS ESI+) *m/z* calcd. for $C_{14}H_{10}N_2O_2$ [M+Na] 261.0640, found 261.0634.

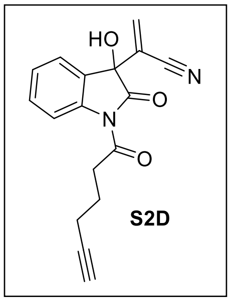

Following the procedure as described above for **S2A**, compound **S2D** was synthesized by employing **S1D** (1.414 gm, 5.86 mmoL, 1 eq.), acrylonitrile (0.25 mL, 35.16 mmoL, 4 eq.) and DABCO (0.657 gm, 5.86 mmoL, 1 eq.). The reaction was continued for 14 h at RT, and the crude of the reaction obtained after work up was purified using column chromatography on silica gel using hexane/EtOAc (7:3) eluent to afford **S2D** as off-white solid (0.607 gm, 35%, R_f 0.37 in hexane/EtOAc 7:3). **^{1}H NMR** (500 MHz, $CDCl_3$) δ 8.28 (d, *J* = 8.3 Hz, 1H), 7.49 (t, *J* = 7.8 Hz, 1H), 7.43 (d, *J* = 7.4 Hz, 1H), 7.33 (d, *J* = 7.5 Hz, 1H), 6.26 (s, 1H), 6.24 (s, 1H), 3.21 (dt, *J* = 23.2, 7.2 Hz, 3H), 2.36 (td, *J* = 6.9, 2.6 Hz, 2H), 1.99 (tt, *J* = 7.3, 4.2 Hz, 3H). **^{13}C NMR** (126 MHz, $CDCl_3$) δ 174.97, 172.86, 140.46, 132.34, 131.93, 126.50, 125.85, 124.68, 123.06, 117.54, 115.18, 83.35, 76.55, 69.58, 37.10, 22.95, 17.88. **HRMS** (QTOF MS ESI+) *m/z* calcd. for $C_{17}H_{14}N_2O_3$ $[M+Na]^+$ 317.0902, found 317.0897.

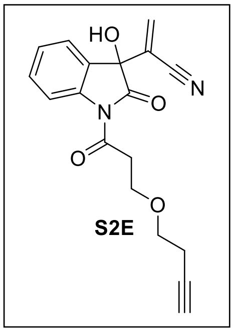

Following the procedure as described above for **S2A**, compound **S2E** was synthesized by employing **S1E** (0.144 gm, 0.53 mmoL, 1 eq.), acrylonitrile (0.13 mL, 13 mmoL, 4 eq.) and DABCO (0.059 gm, 0.053 mmoL, 1 eq.). The reaction was continued for 9 h at RT, and the crude of the reaction obtained after work up was purified using column chromatography on silica gel using hexane/EtOAc (3:2) eluent to afford **S2E** as off-white solid (0.050 gm, 29%, R_f 0.4 in hexane/EtOAc 3:2). **^{1}H NMR** (500 MHz, $CDCl_3$) δ 8.21 (d, *J* = 8.2 Hz, 1H), 7.44 (t, *J* = 8.3 Hz, 1H), 7.39 (d, *J* = 7.4 Hz, 1H), 7.29 (t, *J* = 7.5 Hz, 1H), 6.22 (s, 1H), 6.20 (s, 1H), 4.27 (s, 1H), 3.83 (tt, *J* = 7.1, 3.8 Hz, 2H), 3.59 (t, *J* = 6.9 Hz, 2H), 3.28 (qt, *J* = 17.6, 6.0 Hz, 2H), 2.42 (td, *J* = 6.9, 2.7 Hz, 2H), 1.92 (t, *J* = 2.7 Hz, 1H). **^{13}C NMR** (126 MHz, $CDCl_3$) δ 174.87, 171.30, 140.15, 132.72, 131.69, 126.42, 126.23, 124.62, 122.69, 117.36, 115.25, 81.27, 76.35, 69.61, 69.26, 65.47, 38.55, 19.73. **HRMS** (QTOF MS ESI+) *m/z* calcd. for $C_{18}H_{16}N_2O_4$ $[M+Na]^+$ 347.1008, found 347.1002.

Step3: O-acetylation of Baylis Hillman adducts

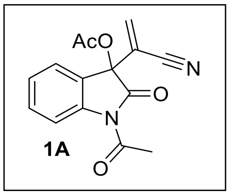

To a stirred solution of **S2A (**0.853 gm, 3.52 mmoL, 1 eq.) and acetic anhydride (2 mL, 21.12 mmoL, 6 eq.) in anhydrous CH_3CN (20 mL) was added a solution of $Sc(OTf)_3$ **(**0.139 gm, 0.281 mmoL, 0.08 eq.) in CH_3CN (1 mL) slowly and dropwise at RT under Ar atmosphere. After 1 h, the

reaction was quenched with sat. $NaHCO_3$ solution in water (10 ml) followed addition of EtOAc (30 mL). The two layers were separated, and the organic layer was washed with brine solution (20 mL), dried (Na_2SO_4), and concentrated *in vacuo* on a rotary evaporator. The resultant crude was subjected to column chromatography on silica gel using EtOAc/hexane (1:1) eluent to afford **1A** as an off-white solid (0.866 gm, 87%, R_f 0.56 in hexane/EtOAc 1:1). **^{1}H NMR** (500 MHz, $CDCl_3$) δ 8.31 (d, J = 8.3 Hz, 1H), 7.49 (t, J = 7.8 Hz, 1H), 7.37 – 7.27 (m, 2H), 6.24 (s, 1H), 5.95 (s, 1H), 2.69 (s, 3H), 2.17 (s, 3H). **^{13}C NMR** (126 MHz, $CDCl_3$) δ 171.24, 170.45, 168.60, 141.01, 134.53, 131.97, 126.14, 123.75, 123.27, 120.58, 117.43, 114.74, 78.57, 26.63, 20.49. **HRMS** (QTOF MS ESI+) *m/z* calcd. for $C_{13}H_{10}N_2O_3$ $[M+Na]^+$ 307.0695, found 307.0689.

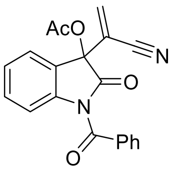

1B

Following the procedure as described above for the synthesis of **1A**, compound **1B** was prepared from **S2B** (0.345 gm, 1.1 mmoL, 1 eq.), acetic anhydride (0.6 mL, 6.8 mmoL, 6eq) and $Sc(OTf)_3$ (48 mg, 0.09 mmoL, 0.08 eq). The crude obtained after the work up of the reaction was subjected to column chromatography on silica gel using hexane/EtOAc (1:1) eluent to afford **1B** as dark red solid (0.273 gm, 60%, R_f 0.45 in hexane/EtOAc 3:2). **^{1}H NMR** (500 MHz, $CDCl_3$ δ 7.88 (d, J = 8.2 Hz, 1H), 7.83 (d, J = 7.7 Hz, 2H), 7.60 (t, J = 7.5 Hz, 1H), 7.49 (dt, J = 19.5, 8.0 Hz, 3H), 7.39 (d, J = 7.5 Hz, 1H), 7.31 (t, J = 7.6 Hz, 1H), 6.24 (s, 1H), 6.01 (s, 1H), 2.18 (s, 3H). **^{13}C NMR** (126 MHz, $CDCl_3$) δ 169.62, 168.65, 168.60, 140.96, 134.41, 133.42, 133.30, 131.78, 129.70, 128.31, 125.75, 123.96, 123.45, 120.37, 115.62, 114.71, 78.54, 20.40. **HRMS** (QTOF MS ESI+) *m/z* calcd. for $C_{20}H_{14}N_2O_4$ $[M+Na]^+$ 369.0851, found 369.0846.

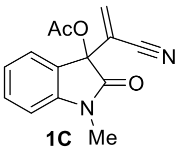

1C

To a stirred and cooled (0 ^{o}C) solution of **S2C** (0.59 gm, 2.7 mmoL, 1 eq.) in anhydrous CH_2Cl_2 (20 mL) was added acetyl chloride (0.6 mL, 8.2 mmoL, 3 eq.) dropwise under N_2 atmosphere. After complete addition, K_2CO_3 (0.761 gm, 5.5 mmoL, 2 eq.) was quickly added to the reaction mixture and the reaction was allowed to stir for 15 min at 0 ^{o}C. The reaction was allowed to warm to RT and stirred for 43 h. The reaction was quenched with water (20 mL) and the aqueous mixture was extracted with CH_2Cl_2 (30 mL). The two layers were separated and the organic layer was washed with brine (20 mL), dried (Na_2SO_4), and concentrated *in vacuo* on a rotary evaporator. The resultant crude was subjected to column chromatography on silica gel using

EtOAc/hexane (1:1) eluent to afford **1C** as an off-white solid (127 mg, 18%, R_f 0.51 in hexane/EtOAc 1:1). **^{1}H NMR** (500 MHz, $CDCl_3$) δ 7.42 (t, *J* = 7.8 Hz, 1H), 7.36 (d, *J* = 7.4 Hz, 1H), 7.13 (t, *J* = 7.6 Hz, 1H), 6.91 (d, *J* = 7.9 Hz, 1H), 6.17 (s, 1H), 6.12 (s, 1H), 3.28 (s, 3H), 2.13 (s, 3H). **^{13}C NMR** (126 MHz, $CDCl_3$) δ 170.63, 168.08, 144.14, 133.11, 131.27, 124.39, 123.76, 123.49, 120.35, 115.10, 109.12, 78.52, 26.88, 20.56. **HRMS** (QTOF MS ESI+) *m/z* calcd. for $C_{14}H_{12}N_2O_3$ [M+Na] 279.0746, found 279.0740.

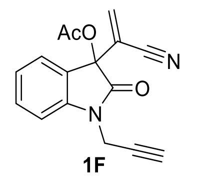

1F

Following the procedure as described above for the synthesis of **1A**, compound **1F** was synthesized from **S2F** (0.195 gm, 0.082 mmoL, 1 eq.), acetic anhydride (0.46 mL, 4.91 mmoL, 6eq) and $Sc(OTf)_3$ (32 mg, 0.0654 mmoL, 0.08 eq.). The crude obtained after the work up of the reaction was subjected to column chromatography on silica gel using hexane/EtOAc (3:2) eluent to afford **1F** as yellow solid (0.153 gm, 60%, R_f 0.46 in hexane/EtOAc 3:2).**^{1}H NMR** (400 MHz, $CDCl_3$) δ 7.43 (t, *J* = 7.8 Hz, 1H), 7.37 (d, *J* = 7.4 Hz, 1H), 7.19 – 7.08 (m, 2H), 6.16 (s, 1H), 6.08 (s, 1H), 4.61 (dd, *J* = 17.8, 2.6 Hz, 1H), 4.46 (dd, *J* = 17.8, 2.5 Hz, 1H), 2.29 (t, *J* = 2.5 Hz, 1H), 2.12 (s, 3H). **^{13}C NMR** (126 MHz, $CDCl_3$) δ 169.82, 168.24, 142.28, 133.50, 131.30, 124.31, 123.93, 123.90, 120.12, 115.04, 110.20, 78.43, 75.95, 73.04, 30.02, 20.49. **HRMS** (QTOF MS ESI+) *m/z* calcd. for $C_{16}H_{12}N_2O_3$ [M+Na] 303.0746, found 303.0740

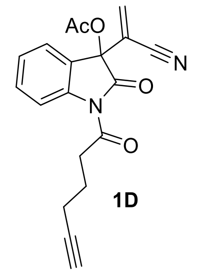

1D

Following the procedure as described above for the synthesis of **1A**, compound **1D** was synthesized from **S2D** (0.257 gm, 0.087 mmoL, 1 eq.), acetic anhydride (0.5 mL, 5.2 mmoL, 6 eq.) and $Sc(OTf)_3$ (34 mg, 0.069 mmoL, 0.08 eq). The crude obtained after the work up of the reaction was subjected to column chromatography on silica gel using hexane/EtOAc (7:3) eluent to afford **1D** as an off-white solid (0.14 gm, 47%, R_f 0.5 in hexane/EtOAc 7:3). **^{1}H NMR** (500 MHz, $CDCl_3$) δ 8.31 (d, *J* = 8.3 Hz, 1H), 7.48 (t, *J* = 8.5 Hz, 1H), 7.31 (dt, *J* = 14.9, 6.9 Hz, 2H), 6.24 (s, 1H), 5.95 (s, 1H), 3.29 – 3.12 (m, 2H), 2.35 (td, *J* = 7.0, 2.7 Hz, 2H), 2.17 (s, 3H), 2.06 – 1.90 (m, 3H). **^{13}C NMR** (126 MHz, $CDCl_3$) δ 173.01, 171.12, 168.60, 141.09, 134.54, 131.97, 126.13, 123.78, 123.39, 120.61, 117.45, 114.76, 83.46, 78.59, 69.44, 37.20, 23.03, 20.51, 17.95. **HRMS** (QTOF MS ESI+) *m/z* calcd. for $C_{19}H_{16}N_2O_4$ $[M+Na]^+$ 359.1008, found 359.1002.

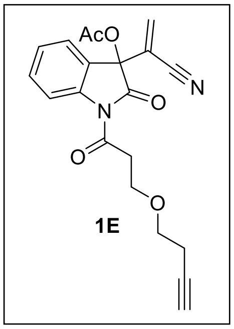

Following the procedure as described above for the synthesis of **1A**, compound **1E** was synthesized from **S2E** (50 mg, 0.15 mmoL, 1 eq.), acetic anhydride (87 µL, 0.92 mmoL, 6 eq.) and $Sc(OTf)_3$ (6 mg, 0.0012 mmoL, 0.08 eq.). The crude obtained after the work up of the reaction was subjected to column chromatography on silica gel using hexane/EtOAc (3:2) eluent to afford **1E** as grey solid (43 mg, 77%, R_f 0.55 in hexane/EtOAc 3:2). **^{1}H NMR** (500 MHz, $CDCl_3$) δ 8.30 (d, J = 8.3 Hz, 1H), 7.47 (t, J = 7.8 Hz, 1H), 7.35 – 7.27 (m, 2H), 6.23 (s, 1H), 5.93 (s, 1H), 3.96 – 3.80 (m, 2H), 3.62 (t, J = 7.0 Hz, 2H), 3.36 (td, J = 6.1, 3.1 Hz, 2H), 2.46 (td, J = 6.9, 2.6 Hz, 2H), 2.15 (s, 3H), 1.95 (t, J = 2.7 Hz, 1H). **^{13}C NMR** (126 MHz, $CDCl_3$) δ 171.22, 171.08, 168.55, 140.91, 134.65, 131.90, 126.14, 123.72, 123.34, 120.47, 117.43, 114.71, 81.33, 78.52, 69.46, 69.27, 65.50, 38.78, 20.45, 19.86. **HRMS** (QTOF MS ESI+) m/z calcd. for $C_{20}H_{18}N_2O_5$ [M+Na] 389.1113, found 389.1108.

Step 4: Click reaction (CuAAC) between N-propargyl IBH and Dansyl azide

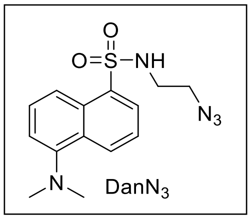

The compound $DanN_3$ was synthesized according to the reported protocol in the literature[4]. **^{1}H NMR** (500 MHz, $CDCl_3$) δ 8.56 (d, J = 8.5 Hz, 1H), 8.26 (t, J = 7.3 Hz, 2H), 7.60 (d, J = 8.2 Hz, 1H), 7.53 (t, J = 7.9 Hz, 1H), 7.20 (d, J = 7.5 Hz, 1H), 5.06 (t, J = 6.4 Hz, 1H), 3.30 (t, J = 5.7 Hz, 2H), 3.06 (q, J = 6.0 Hz, 2H), 2.89 (s, 6H). **^{13}C NMR** (126 MHz, $CDCl_3$) δ 152.26, 134.60, 130.95, 130.10, 129.76, 129.62, 128.82, 123.28, 118.58, 115.51, 51.06, 45.54, 42.52. **HRMS** (QTOF MS ESI+) m/z calcd. for $C_{14}H_{17}N_5O_2S$ $[M+H]^+$ 320.1181, found 320.1176.

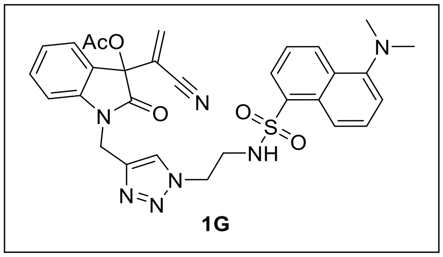

To a stirred mixture of **1F** (0.105 gm, 0.037 mmoL, 1 eq.) and $DanN_3$ (0.12 gm, 0.037 mmoL, 1 eq.) in CH_3CN (10 mL) was added CuI (142 mg, 0.0749 mmoL, 2 eq.) followed by the addition of DIPEA (0.2 mL, 1.12 mmoL, 3 eq.) at RT. The progress of the reaction was monitored by TLC. After the complete consumption of both the starting materials in about 1 h, the reaction was quenched with 10% citric acid solution in water (10 mL) and the aqueous mixture was

extracted with EtOAc (3 × 20 mL). The organic layers were combined and washed with brine solution (30 mL), dried over Na_2SO_4, and concentrated *in vacuo* on a rotary evaporator. The resultant crude was subjected to column chromatography on silica gel using hexane/EtOAc (2:3) eluent to afford **1G** as a brown sticky solid (129 mg, 57% yield, R_f 0.36 in hexane/EtOAc 2:3). **^{1}H NMR** (500 MHz, $CDCl_3$) δ 8.54 (d, *J* = 8.5 Hz, 1H), 8.20 (d, *J* = 7.3 Hz, 1H), 8.13 (d, *J* = 8.6 Hz, 1H), 7.57 (s, 1H), 7.51 (q, *J* = 8.5 Hz, 2H), 7.37 (t, *J* = 7.7 Hz, 1H), 7.33 – 7.29 (m, 1H), 7.19 (d, *J* = 7.6 Hz, 1H), 7.13 (t, *J* = 7.3 Hz, 2H), 6.23 (s, 1H), 6.21 (s, 1H), 5.58 (t, *J* = 6.8 Hz, 1H), 5.18 (d, *J* = 15.8 Hz, 1H), 4.90 (d, *J* = 15.7 Hz, 1H), 4.26 (ddd, *J* = 14.2, 6.5, 3.4 Hz, 1H), 4.10 (ddd, *J* = 14.1, 7.6, 3.5 Hz, 1H), 3.50 – 3.26 (m, 2H), 2.88 (s, 6H), 2.20 (s, 3H).**^{13}C NMR** (176 MHz, $CDCl_3$) δ 170.39, 169.27, 152.21, 142.53, 141.96, 134.66, 133.47, 131.62, 130.98, 130.03, 129.69, 129.47, 128.90, 124.09, 123.96, 123.80, 123.26, 120.15, 118.55, 115.55, 115.06, 78.81, 77.34, 50.62, 45.55, 43.04, 36.01, 20.72. **^{13}C DEPT-135** (126 MHz, $CDCl_3$) δ *133.43*, 131.61, 130.98, 129.68, 128.90, 123.95, 123.80, 123.26, 118.57, 115.57, 110.65, 77.36, *50.62*, 45.55, *43.05*, *36.03* (Negative signals are denoted in *italics*). **HRMS** (QTOF MS ESI+) *m/z* calcd. for $C_{30}H_{29}N_7O_5S$ $[M+Na]^+$ 622.1849, found 622.1843.

Supplementary Fig. 3. Synthesis of C=C linked bis-heterocycles (**2A**–**C** and **S2A-Cys**) via IBH adduct-mediated 1,2-aminothiol derivatization.

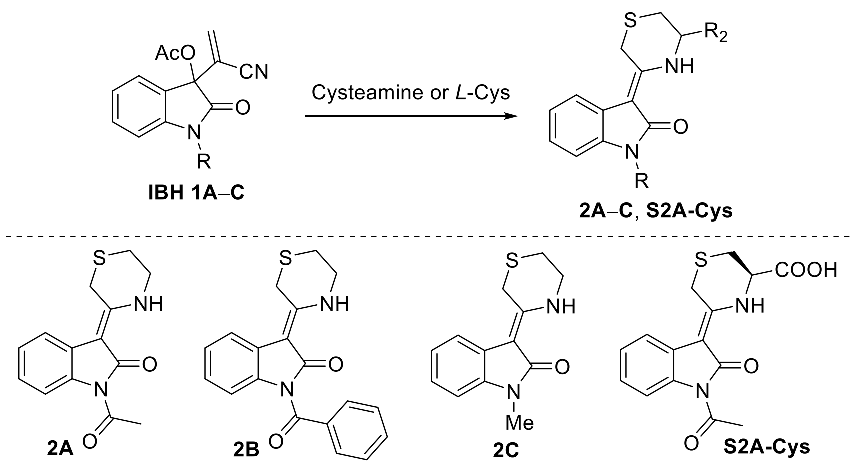

General procedures for screening reaction conditions

<u>Procedure 1: Reactions performed in organic solvents (entries 1–7, Fig. 2B).</u>

A solution of cysteamine·HCl (20 mg, 0.17 mmoL, final conc. 4 mM, 1eq.) and Et_3N (24.5 µL, 0.17 mmoL, 1eq.) was prepared in 2 mL (4.5% of the total reaction volume) of the reaction solvent and the resulting clear solution was added to a solution of **1A** (50 mg, 0.17 mmoL, final conc.

4 mM, 1eq.) in 42 mL of reaction solvent at RT (entries 1–3). In case of reactions performed in a mixture of two organic solvents (entries 4–7), the solution of cysteamine·HCl and Et_3N was made in 2 mL DMSO. After the addition, the reaction mixture was stirred at RT for 15 min. The volatile solvents (MeOH/CH_3CN/EtOAc/THF) were removed *in vacuo* before the aqueous workup. The reaction mixture was diluted with water (10 mL) and extracted with EtOAc (20 mL × 3). The organic layers were combined and washed with brine (10 mL), dried (anhydrous Na_2SO_4), and concentrated *in vacuo*. The resultant crude was subjected to column chromatography on silica gel using hexane/EtOAc (1:1) eluent to afford **2A** as a light-yellow solid in 35–95% isolated yields.

Procedure 2: Reactions performed in a mixture of organic solvent and sodium phosphate buffer (entries 8–10, Fig. 2B).

A solution of cysteamine·HCl (20 mg, 0.17 mmoL, final conc. 4 mM, 1eq.) in sodium phosphate buffer (1 mL, 50 mM, pH 7) was slowly added to a solution of **1A** (50 mg, 0.17 mmoL, final conc. 4 mM, 1eq.) in a mixture of sodium phosphate buffer (25 mL) and organic solvent (18 mL, 40% of the total reaction volume). After stirring for 15 min at RT, the reaction mixture was extracted with EtOAc (20 mL × 3). The organic layers were combined, washed with brine (10 mL), dried (anhydrous Na_2SO_4) and concentrated *in vacuo*. The resultant crude was subjected to column chromatography on silica gel using hexane/EtOAc (1:1) eluent to afford **2A** as light-yellow solid in 71–89% isolated yields. A similar procedure was followed for the synthesis of conjugates **2B**, **2C** and **S2A-Cys** employing **1B** and cysteamine·HCl for **2B**; **1C** and cysteamine·HCl for **2C**; **1A** and *L*-cysteine for **S2A-Cys**.

Characterization and purification of conjugates **2A**–**C**, **S2A-Cys**.

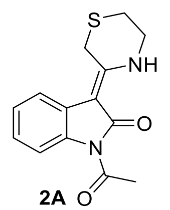

2A

Compound **2A** was synthesized following procedures 1 and 2 as described above. The crude obtained after the work up of the reaction was subjected to column chromatography on silica gel using hexane/EtOAc (1:1) eluent to afford **2A** as light-yellow solid (R_f 0.54 in hexane/EtOAc 1:1). **^{1}H NMR** (500 MHz, CD_3CN) δ 10.79 (s, 1H), 8.24 (d, *J* = 8.0 Hz, 1H), 7.36 (d, *J* = 7.5 Hz, 1H), 7.19 – 6.91 (m, 2H), 3.96 (s, 2H), 3.84 – 3.66 (m, 2H), 2.97 – 2.92 (m, 2H), 2.65 (s, 3H). **^{13}C NMR** (126 MHz, CD_3CN) δ 172.15, 169.26, 161.58, 134.35, 126.48, 124.66, 123.46, 118.74, 116.22, 92.16, 42.13, 27.20, 26.15, 25.71. **^{13}C DEPT-135** (126 MHz, CD_3CN) δ 124.66,

123.46, 118.74, 116.22, *42.13*, 27.20, *26.15*, *25.71* (Negative signals are denoted in *italics*). HRMS (QTOF MS ESI+) *m/z* calcd. for $C_{14}H_{14}N_2O_2S$ $[M+Na]^+$ 297.0674, found 297.0668.

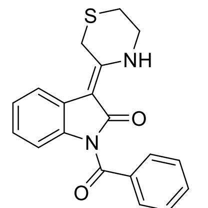
2B

The reaction was performed with **1B** (32 mg, 0.09 mmoL, 1 eq.) and cysteamine·HCl (10.49 mg, 0.09 mmoL, 1 eq.) at RT following procedure 2 as described above. The crude of the reaction was subjected to column chromatography on silica gel using hexane/EtOAc (1:1) eluent to afford **2B** as dark red solid (29 mg, 93%, R_f 0.4 in hexane/EtOAc 1:1). **^{1}H NMR** (500 MHz, CD_3CN) δ 10.52 (s, 1H), 7.86 (d, *J* = 8.7 Hz, 1H), 7.69 (d, *J* = 7.1 Hz, 2H), 7.57 (t, *J* = 7.5 Hz, 1H), 7.46 (t, *J* = 7.9 Hz, 3H), 7.18 – 7.07 (m, 2H), 4.03 (s, 2H), 3.70 – 3.63 (m, 2H), 2.97 – 2.90 (m, 2H). **^{13}C NMR** (126 MHz, CD_3CN) δ 170.84, 168.48, 161.79, 137.19, 134.67, 132.76, 130.04, 128.85, 126.88, 124.66, 123.52, 119.21, 114.91, 91.94, 42.17, 26.24, 25.86. **^{13}C DEPT-135** (126 MHz, CD_3CN) δ 132.76, 130.03, 128.84, 124.65, 123.52, 119.20, 114.90, *42.17*, *26.24*, *25.85* (Negative signals are denoted in *italics*). **HRMS** (QTOF MS ESI+) *m/z* calcd. for $C_{19}H_{16}N_2O_2S$ $[M+Na]^+$ 359.0830, found 359.0825.

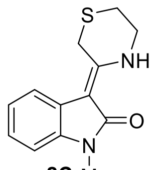
2C

The reaction was performed with **1C** (10 mg, 0.039 mmoL, 1 eq.) and cysteamine·HCl (4.43 mg, 0.039 mmoL, 1 eq.) for 5 h at RT following procedure 2 as described above. The crude of the reaction was subjected to column chromatography on silica gel using hexane/EtOAc (1:1) eluent to afford **2C** as light-yellow solid (8 mg, 83% yield, R_f 0.36 in hexane/EtOAc 1:1). **^{1}H NMR** (700 MHz, CD_3CN -d_3 δ 10.66 (s, 1H), 7.35 (d, *J* = 7.7 Hz, 1H), 7.07 – 7.03 (m, 1H), 6.98 – 6.93 (m, 2H), 3.99 (s, 2H), 3.70 (ddd, *J* = 6.7, 5.7, 4.0 Hz, 2H), 3.27 (s, 3H), 2.96 – 2.93 (m, 2H).**^{13}C NMR** (176 MHz, CD_3CN) δ 169.45, 159.09, 138.30, 124.77, 123.06, 121.49, 118.98, 108.37, 92.75, 41.76, 26.41, 25.78, 25.77. **^{13}C DEPT-135** (176 MHz, CD_3CN) δ 123.06, 121.49, 118.99, 108.37, *41.76*, *26.42*, *25.79*, 25.77 (Negative signals are denoted in *italics*). **HRMS** (QTOF MS ESI+) *m/z* calcd. for $C_{13}H_{14}N_2OS$ $[M+Na]^+$ 269.0725, found 269.0719.

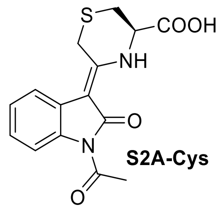
S2A-Cys

The reaction was performed with **1A** (20 mg, 0.07 mmoL, 1 eq.) and *L*-cysteine hydrochloride monohydrate (12 mg, 0.07 mmoL, 1 eq.) for 30 min at RT following procedure 2 as described above with slightly modified reaction work up. After 30 min, the reaction mixture was kept

at -80 °C for 1 h and the solvents were freeze dried. To the resultant sodium phosphate salts obtained after freeze drying, was added DMSO-d_6 (0.5 mL) to dissolve the organic components from the salts. The NMR analysis of the sample showed signals exclusively for the desired product **S2A-Cys** demonstrated 100% conversion. **^{1}H NMR** (500 MHz, DMSO-d_6) δ 11.17 (s, 1H), 8.17 (d, *J* = 8.0 Hz, 1H), 7.48 (d, *J* = 7.7 Hz, 1H), 7.09 (t, *J* = 7.6 Hz, 1H), 7.02 (t, *J* = 7.7 Hz, 1H), 4.15 – 4.04 (m, 2H), 3.95 (d, *J* = 15.8 Hz, 1H), 3.35 (dd, *J* = 12.0, 4.3 Hz, 1H), 2.77 (t, *J* = 11.5 Hz, 1H), 2.67 (s, 3H). **^{13}C NMR** (126 MHz, DMSO-d_6) δ 170.87, 170.04, 167.29, 159.75, 132.67, 125.51, 123.84, 122.24, 117.82, 114.91, 90.20, 54.08, 28.54, 26.71, 24.68. **^{13}C 135-DEPT** (126 MHz, DMSO) δ 123.59, 121.99, 117.57, 114.65, 53.82, *28.28*, 26.46, *24.43* (Negative signals are denoted in *italics*). **HRMS** (QTOF MS ESI-) *m/z* calcd. for $C_{15}H_{14}N_2O_4S$ $[M-H]^-$ 317.0596, found 317.0591.

X-ray crystallographic data for IBH-cysteamine conjugates 2A and 2C

Data for the conjugates **2A** and **2C** were recorded by using monochromatic Mo-Kα radiation (α = 0.71073 Å) on a Brüker APEX-II CCD diffractometer at low temperature (140K). Structures were solved using WinGX-Version 2020.1 software, and the refinement was performed using SHELXL[5].

Supplementary Fig. 4. ORTEP diagram of IBH-cysteamine conjugates 2A and 2C with thermal ellipsoids at the 50% probability level. The black dotted line denotes the N–H···O=C hydrogen bond.

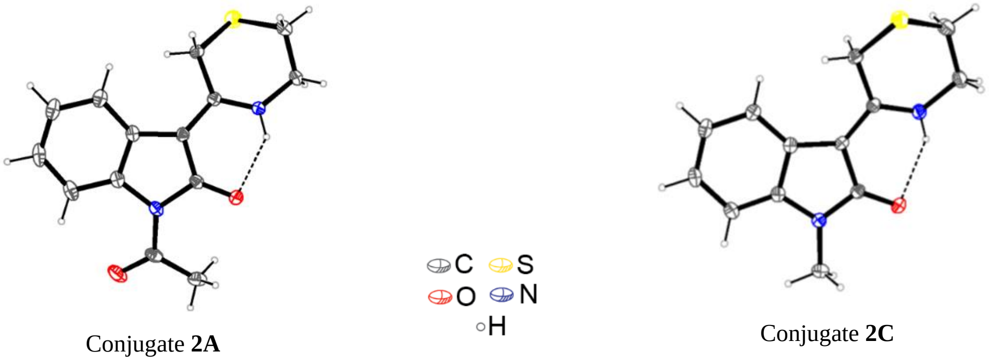

Conjugate **2A** Conjugate **2C**

Hydrogen bonding interactions in conjugates **2A** and **2C**. D and A denote donor and acceptor, respectively.

Compound	D-H --- A	Bond length D-H	H-bond length H --- A	Distance between D-A	Bond angle D-H --- A
**2A**	N-H --- O=C	0.88 Å	1.977 Å	2.698 Å	138.18°
**2C**	N-H --- O=C	0.88 Å	2.049 Å	2.737 Å	134.3°

Crystal data and structure refinement parameters for conjugates **2A** and **2C**

Parameter	2A	2C
**CCDC number**	2259911	2259910
**Empirical formula**	$C_{14}H_{14}N_2O_2S$	$C_{13}H_{14}N_2OS$
**Formula weight**	274.33	246.32
**Temperature/K**	140	140
**Crystal system**	monoclinic	monoclinic
**Space group**	C2/c	$P2_1/c$
**a/Å**	24.5467(19)	9.956(4)
**b/Å**	6.5458(6)	5.415(4)
**c/Å**	18.1901(15)	21.524(7)
**α/°**	90	90
**β/°**	120.986(3)	98.750(10)
**γ/°**	90	90
**Volume/Å^3**	2505.6(4)	1146.9(10)
**Z**	8	4
**ρ calc. g/cm^3**	1.444	1.427
**μ/mm^{-1}**	0.257	0.266
**F(000)**	1136.0	520.0
**Radiation**	MoKα (λ = 0.71073)	MoKα (λ = 0.71073)
**2Θ range for data collection/°**	4.632 to 55.792	4.14 to 57.476
**Index ranges**	-32 ≤ h ≤ 32, -8 ≤ k ≤ 8, -23 ≤ l ≤ 23	-11 ≤ h ≤ 13, -7 ≤ k ≤ 7, -24 ≤ l ≤ 29
**Reflections collected**	30247	13764
**Independent reflections**	2995 [R_{int} = 0.0449, R_{sigma} = 0.0234]	2976 [R_{int} = 0.0803, R_{sigma} = 0.0653]
**Data/restraints/parameters**	2995/0/173	2976/0/154
**Goodness-of-fit on F^2**	0.929	1.061
**Final R indexes [I>=2σ (I)]**	R_1 = 0.0396, wR_2 = 0.1017	R_1 = 0.0544, wR_2 = 0.1475
**Final R indexes [all data]**	R_1 = 0.0512, wR_2 = 0.1101	R_1 = 0.0656, wR_2 = 0.1609
**Largest diff. peak/hole/e Å^{-3}**	0.78/-0.33	0.76/-0.67

3. Synthesis and characterization of thiazolidine-appended lipoic acid analogs

Supplementary Fig. 5. Synthesis of *N*-boc pentafluorophenyl thiazolidine-4-carboxylate (**S8**)

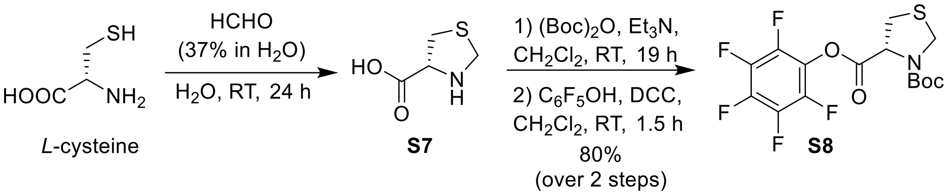

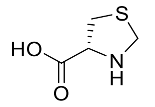

S7

The compound **S7** was synthesized according to the reported protocol in the literature[6]. **^{1}H NMR** (500 MHz, D_2O) δ 4.48 (t, *J* = 8.4 Hz, 2H), 4.37 (d, *J* = 10.3 Hz, 1H), 3.44 (dd, *J* = 12.1, 7.5 Hz, 1H), 3.35 (dd, *J* = 12.2, 5.7 Hz, 1H). **^{13}C NMR** (126 MHz, D_2O) δ 171.90, 63.93, 48.59, 32.95. **^{13}C 135-DEPT** (126 MHz, D_2O) δ 63.93, *48.58, 32.94* (Negative signals are denoted in *italics*). **HRMS** (QTOF MS ESI+) *m/z* calcd. for $C_4H_7NO_2S$ $[M+H]^+$ 134.0276, found 134.0270.

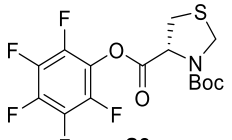

S8

To a stirred solution of **S7** (0.2 gm, 1.49 mmoL, 1 eq.) and Et_3N (0.34 mL, 1.49 mmoL, 1 eq.) in anhydrous CH_2Cl_2 (15 mL) was added $(Boc)_2O$ (0.33 mL, 2.39 mmoL, 1.6 eq.) at 0 ^oC under N_2 atmosphere. After 15 min, the reaction mixture was allowed to warm to RT and stirred for 19 h. Next, the reaction mixture was concentrated *in vacuo* to remove CH_2Cl_2 and the resultant reaction mixture was diluted with EtOAc (30 mL) and 10% citric acid solution in water (10 mL). The two layers were separated and the aqueous layer was re-extracted with EtOAc (20 mL × 2). The organic layers were combined and washed with brine solution (30 mL), dried over Na_2SO_4, and concentrated *in vacuo* to afford analytically pure *N*-Boc thiazolidine acid as colourless oil and was used directly for the next step without purification (**^{1}H NMR** (500 MHz, $CDCl_3$) δ 4.97 – 4.31 (m, 3H), 3.31 (br d, 2H), 1.49 (s, 9H)). *N*-boc thiazolidine acid (0.322 gm, 1.38 mmoL, 1 eq.) was dissolved in anhydrous CH_2Cl_2 (15 mL), and to this solution was added pentafluoro phenol (0.254 gm, 1.38 mmoL, 3 eq.) and DCC (0.285 gm, 1.38 mmoL, 3 eq.) at 0 ^oC under N_2 atmosphere. The reaction mixture was allowed to warm to RT and stirred for 1.5 h during which time the insoluble dicyclohexylurea (DCU) precipitated in the reaction mixture. The reaction mixture was filtered through celite to remove DCU and the filtrate was concentrated *in vacuo*. The resultant mixture was suspended in cold diethyl ether (20 mL) and kept at 4 ^oC for 1 h to precipitate most of the DCU in the mixture. The clear solution was

carefully decanted to another round bottom flask and concentrated *in vacuo* on a rotatory evaporator. The crude was subjected to column chromatography on silica gel using hexane/ethyl acetate (4:1) eluent to afford **S8** as colourless oil (0.443 gm, 80% yield, R_f 0.55 in hexane/EtOAc 4:1). The TLC of the reaction was visualized using ninhydrin staining solution. **^{1}H NMR** (500 MHz, $CDCl_3$), major/minor rotamers (3:2) δ 4.99 (d, *J* = 3.2 Hz, 1H), 4.77–4.41 (m, 2H), 3.59–3.28 (m, 2H), 1.48 (d, *J* = 10.3 Hz, 9H). **^{13}C NMR** (126 MHz, $CDCl_3$) δ 167.27, *166.95*, *153.18*, 152.85, 142.26–142.07 (mix), 141.03–140.80 (mix), 140.26–140.06 (mix), 139.18–138.81 (mix), 137.14–136.94 (mix), 124.90–124.93 (mix), 82.27, *81.99*, *61.51*, 61.35, 49.41, *48.54*, 34.97, *33.46*, *28.35*, 28.20. **^{13}C 135-DEPT** (126 MHz, $CDCl_3$) δ *61.37*, 61.21, 49.27, *48.40*, 34.83, *33.32*, *28.21*, 28.06. ^{13}C chemical shifts denoted in *italic* correspond to the minor rotamer. **^{19}F NMR** (471 MHz $CDCl_3$) δ -152.09 (d, *J* = 18.4 Hz, 0.75 F), -152.78 (d, *J* = 18.0 Hz, 1.13 F), -157.05 (t, *J* = 21.6 Hz, 0.58 F), -157.50 (t, *J* = 21.6 Hz, 0.35 F), -161.73 (td, *J* = 23.0, 5.5 Hz, 1.09 F), -162.10 (t, *J* = 19.9 Hz, 0.76 F). **HRMS** (QTOF MS ESI+) *m/z* calcd. for $C_{15}H_{14}F_5NO_4S$ $[M+Na]^+$ 422.0461, found 422.0456.

Supplementary Fig. 6. Synthesis of *tert*-butyl ester intermediates of amino acids (**S15**–**17**)

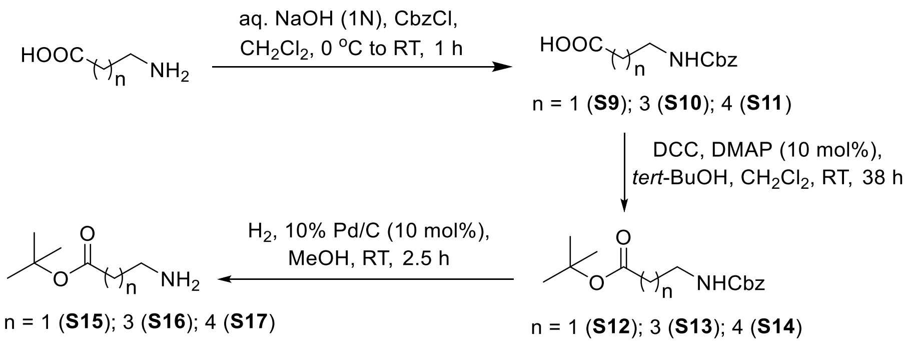

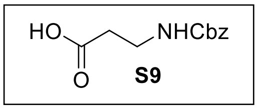

The compound **S9** was synthesized according to the reported protocol in the literature[7]. ^{1}H NMR (500 MHz, $CDCl_3$) δ 7.35 (s, 5H), 5.27 (s, 1H), 5.10 (s, 2H), 3.48 (q, *J* = 6.2 Hz, 2H), 2.62 (t, *J* = 5.9 Hz, 2H).

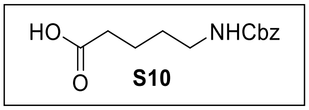

The compound **S10** was synthesized according to the reported protocol in the literature[7]. **^{1}H NMR** (500 MHz, CDCl3) δ 7.35 (s, 5H), 5.09 (s, 2H), 4.85 (s, 1H), 3.21 (q, *J* = 6.8 Hz, 2H), 2.37 (q, *J* = 10.3, 8.8 Hz, 2H), 1.61 (dp, *J* = 50.8, 7.2 Hz, 4H).

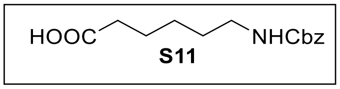

The compound **S11** was synthesized according to the reported protocol in the literature[7]. **^{1}H NMR** (500 MHz, CDCl3) δ 7.35 (s, 5H), 5.27 (s, 1H), 5.10 (s, 2H), 3.48 (q, *J* = 6.2 Hz, 2H), 2.62 (t, *J* = 5.9 Hz, 2H).

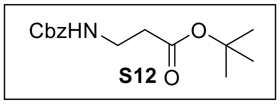

To a stirred solution of **S9** (3.4 gm, 15.2 mmoL, 1 eq.), DMAP (0.186 gm, 1.5 mmoL, 0.1 eq.) and *tert*-BuOH (14.5 mL, 152.3 mmoL, 10 eq.) in anhydrous CH_2Cl_2 (40 mL) was added DCC (3.8 gm, 18.2 mmoL, 1.2 eq.) at 0 °C under N_2 atmosphere. After addition, the reaction mixture was allowed to warm to RT and stirred for an additional 38 h. The precipitated DCU in the mixture was removed by filtration through a celite pad, and the filtrate was concentrated *in vacuo*. The resultant crude mixture was purified by column chromatography on silica gel using hexane/EtOAc (7:3) eluent to afford **S12** as colourless oil (1.92 gm, 45%, R_f 0.65 in hexane/EtOAc 7:3). The TLC of the reaction was visualized using PMA staining solution. **^{1}H NMR** (500 MHz, $CDCl_3$) δ 7.38–7.27 (m, 5H), 5.08 (s, 2H), 4.80 (s, 1H), 3.19 (q, *J* = 6.8 Hz, 2H), 2.20 (t, *J* = 7.4 Hz, 2H), 1.59 (p, *J* = 7.4 Hz, 2H), 1.50 (q, *J* = 7.4 Hz, 2H), 1.43 (d, *J* = 1.1 Hz, 9H), 1.37–1.27 (m, 2H). **^{13}C NMR** (126 MHz, $CDCl_3$) δ 171.79, 156.40, 136.68, 128.63, 128.21, 81.17, 66.78, 36.86, 35.66, 28.23. **HRMS** (QTOF MS ESI+) *m/z* calcd. for $C_{15}H_{21}NO_4$ $[M+Na]^+$ 302.1368, found 302.1363.

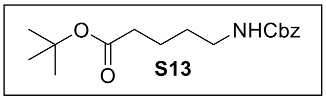

Following the procedure as described above for the synthesis of **S12**, compound **S13** was synthesized from **S10** (2.864 gm, 11.39 mmoL, 1eq.), DCC (2.82 gm, 13.6 mmoL, 1.2 eq.), DMAP (0.139 gm, 1.13 mmoL, 0.1 eq.) and *tert*-BuOH (11 mL, 113.9 mmoL, 10 eq.). The crude obtained after the work up of the reaction was subjected to column chromatography on silica gel using hexane/EtOAc (7:3) eluent to afford **S13** as colourless oil (0.111 gm, 39% yield, R_f 0.63 in hexane/EtOAc 7:3). **^{1}H NMR** (500 MHz, $CDCl_3$) δ 7.37–7.28 (m, 5H), 5.08 (s, 2H), 4.87 (s, 1H), 3.19 (q, *J* = 6.6 Hz, 2H), 2.22 (t, *J* = 7.2 Hz, 2H), 1.56 (dp, *J* = 42.3, 6.9 Hz, 4H), 1.43 (s, 9H). **^{13}C NMR** (126 MHz, $CDCl_3$) δ 172.92, 156.48, 136.74, 128.60, 128.17, 80.34, 77.41, 77.16, 76.91, 66.69, 40.77, 35.10, 29.44, 28.21, 22.21. **HRMS** (QTOF MS ESI+) *m/z* calcd. for $C_{17}H_{25}NO_4$ $[M+Na]^+$ 330.1681, found 330.1676.

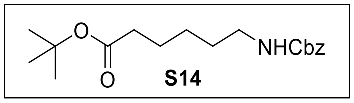

Following the procedure as described above for the synthesis of **S12**, compound **S14** was prepared from **S11** (2.55 gm, 9.6 mmoL, 1 eq.), DCC (2.38 gm, 11.5 mmoL, 1.2 eq.), DMAP (0.117 gm, 0.96 mmoL, 0.1 eq.) and

tert-BuOH (9 mL, 96.1 mmoL, 10 eq.). The crude obtained after the work up of the reaction was subjected to column chromatography on silica gel using hexane/EtOAc (7:3) eluent to afford **S14** as colourless oil (1.429 gm, 46%, R_f 0.67 in hexane/EtOAc 7:3). ^{1}**H NMR** (500 MHz, CDCl3) δ 7.38–7.27 (m, 5H), 5.08 (s, 2H), 4.80 (s, 1H), 3.19 (q, *J* = 6.8 Hz, 2H), 2.20 (t, *J* = 7.4 Hz, 2H), 1.59 (p, *J* = 7.4 Hz, 2H), 1.50 (q, *J* = 7.4 Hz, 2H), 1.43 (d, *J* = 1.1 Hz, 9H), 1.37–1.27 (m, 2H). ^{13}**C NMR** (126 MHz, $CDCl_3$) δ 173.10, 156.49, 136.77, 128.62, 128.21, 128.18, 80.21, 66.69, 40.99, 35.49, 29.77, 28.23, 26.28, 24.77. **HRMS** (QTOF MS ESI+) *m/z* calcd. for $C_{18}H_{27}NO_4$ $[M+Na]^+$ 344.1838, found 344.1832.

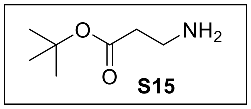

A solution of compound **S12** (0.5 gm, 1.78 mmoL, 1 eq.) in anhydrous MeOH (25 mL) was purged with N_2 gas for 15 min before the addition of 10% Pd/C catalyst (10 mol%, 50 mg). The resultant heterogenous mixture was hydrogenated at 50 psi pressure of H_2 in a Parr reactor. The reaction was monitored by TLC. After 2.5 h, the reaction mixture was filtered through a celite bed and the filtrate was concentrated in *vacuo* to afford analytically pure **S15** as colourless oil (0.23 gm, 89%, R_f 0.24 in CH_2Cl_2/MeOH 9:1). The TLC of the reaction was visualized using PMA and ninhydrin staining solutions. ^{1}**H NMR** (500 MHz, CD_3OD) δ 2.64 (dd, *J* = 7.9, 3.7 Hz, 2H), 2.22 (dt, *J* = 11.3, 7.3 Hz, 2H), 1.64–1.55 (m, 2H), 1.53–1.40 (m, 11H), 1.35 (tdd, *J* = 10.9, 7.3, 4.0 Hz, 2H). ^{13}**C NMR** (126 MHz, CD_3OD) δ 174.92, 81.35, 42.19, 36.29, 32.97, 28.37, 27.37, 25.97. **HRMS** (QTOF MS ESI+) *m/z* calcd. for $C_7H_{15}NO_2$ $[M+H]^+$ 146.1181, found 146.1177.

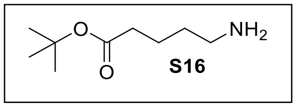

Following the procedure described above for the synthesis of **S15**, compound **S16** was obtained as colourless oil (0.386 gm, 86% yield, R_f 0.18 in CH_2Cl_2/MeOH 9:1) after the hydrogenation of **S13** (0.8 g, 2.6 mmoL, 1 eq). ^{1}**H NMR** (500 MHz, CD_3OD) δ 2.68 (t, *J* = 7.2 Hz, 2H), 2.25 (t, *J* = 7.2 Hz, 2H), 1.61 (h, *J* = 6.1, 5.1 Hz, 2H), 1.52 (p, *J* = 7.5 Hz, 2H), 1.45 (s, 9H). ^{13}**C NMR** (126 MHz, CD_3OD) δ 174.67, 81.39, 49.00, 41.85, 36.06, 32.32, 28.38, 23.39. **HRMS** (QTOF MS ESI+) *m/z* calcd. for $C_9H_{19}NO_2$ $[M+Na]^+$ 174.1494, found 174.1489.

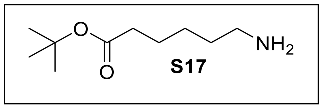

Following the procedure described above for the synthesis of **S15**, compound **S17** was obtained as colourless oil (0.327gm, 90%, R_f 0.27 in hexane/EtOAc 1:9) after the hydrogenation of **S14** (0.62 gm, 1.9 mmoL, 1 eq.). ^{1}**H NMR**

(500 MHz, CD_3OD) δ 2.64 (dd, J = 7.9, 3.7 Hz, 2H), 2.22 (dt, J = 11.3, 7.3 Hz, 2H), 1.64–1.55 (m, 2H), 1.53–1.40 (m, 11H), 1.35 (tdd, J = 10.9, 7.3, 4.0 Hz, 2H). **^{13}C NMR** (126 MHz, MeOD) δ 174.92, 81.35, 42.19, 36.29, 32.97, 28.37, 27.37, 25.97. **HRMS** (QTOF MS ESI+) *m/z* calcd. for $C_{10}H_{21}NO_2$ $[M+H]^+$ 188.1651, found 188.1645.

Supplementary Fig. 7. Synthesis of C3Tz and C7Tz lipoic acid analogs

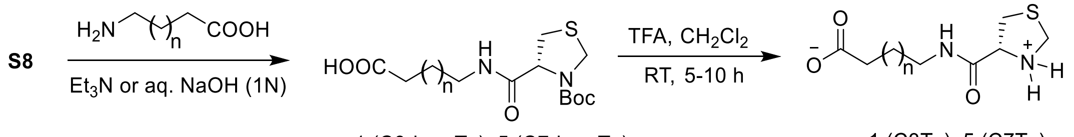

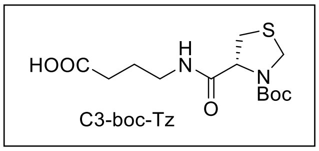

To a stirred solution of **S8** (0.439 gm, 1.09 mmoL, 1eq.) in anhydrous CH_2Cl_2 (20 mL) was added 4-aminobutyric acid (0.113 gm, 1.09 mmoL, 1 eq.) and Et_3N (0.3 mL, 2.19 mmoL, 2 eq.) at RT under N_2 atmosphere. After 5.5 h, the reaction mixture was quenched with 10% citric acid solution in water (20 mL). The resultant two layers were separated and the aqueous layer was extracted with CH_2Cl_2 (30 mL × 3). The organic layers were combined and washed with brine solution (30 mL), dried over Na_2SO_4, and concentrated *in vacuo*. The resultant crude was subjected to column chromatography on silica gel using CH_2Cl_2/MeOH (9:1) eluent to afford C3-boc-Tz as a colourless oil (0.102 gm, 29% yield, R_f 0.42 in CH_2Cl_2/MeOH 9:1). The TLC of the reaction was visualized using ninhydrin staining solution. **^{1}H NMR** (500 MHz, DMSO-d_6) δ 12.01 (s, 1H), 8.02 (s, 1H), 4.45 (dd, J = 113.4, 9.0 Hz, 3H), 3.27 (s, 1H), 3.05 (dddd, J = 37.6, 24.5, 12.4, 5.6 Hz, 3H), 2.21 (t, J = 7.5 Hz, 2H), 1.63 (t, J = 7.2 Hz, 2H), 1.37 (s, 9H). **^{13}C NMR** (126 MHz, DMSO) δ 174.21, 170.06, 152.77, 79.88, 62.44, 49.38, 39.85, 38.03, 35.01, 30.91, 27.93, 24.52. **HRMS** (QTOF MS ESI+) *m/z* calcd. for $C_{13}H_{22}N_2O_5S$ $[M+Na]^+$ 341.1147, found 341.1142.

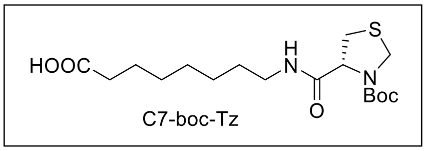

To a stirred solution of 8-aminooctanoic acid (0.1 gm, 0.62 mmoL, 1 eq.) in 1N NaOH solution in water (1.25 mL, 1.2 mmoL, 2 eq.) was added a solution of **S8** (0.5 gm, 1.2 mmoL, 2 eq.) in THF (2 mL) dropwise at 0 ^{o}C. After 15 min of stirring at 0 ^{o}C, the reaction mixture was allowed to warm to RT and stirred for additional 30 min. Next, the reaction mixture

was neutralized (pH 7) with 1N HCl solution in water and the THF was removed *in vacuo*. The neutral aqueous solution was diluted with water (3 mL), and the pH was adjusted to acidic (~6) with 1N HCl aqueous solution. The resultant acidic aqueous solution was extracted with EtOAc (30 mL × 3). The organic layers were combined and washed with brine solution (30 mL), dried over Na_2SO_4, and concentrated *in vacuo*. The resultant crude mixture was subjected to column chromatography on silica gel using CH_2Cl_2/MeOH (9:1) eluent to afford C7-boc-Tz as colourless oil (29 mg, 26% yield, R_f 0.37 in CH_2Cl_2/MeOH 9:1). The TLC of the reaction was visualized using ninhydrin staining solution. **^{1}H NMR** (500 MHz, $CDCl_3$) δ 6.53 (s, 1H), 4.79 - 4.52 (m, 2H), 4.35 (s, 1H), 3.25 (qq, *J* = 13.2, 6.5 Hz, 4H), 2.31 (t, *J* = 7.4 Hz, 2H), 1.60 (t, *J* = 7.2 Hz, 2H), 1.45 (s, 11H), 1.31 (s, 6H). **^{13}C NMR** (126 MHz, $CDCl_3$) δ 178.54, 170.47, 154.46, 82.29, 61.54, 49.79, 39.70, 34.00, 29.49, 29.01, 28.90, 28.38, 26.63, 24.66. **HRMS** (QTOF MS ESI+) *m/z* calcd. for $C_{17}H_{30}N_2O_5S$ $[M+Na]^+$ 379.1773, found 379.1774.

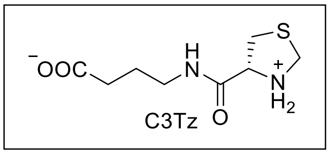

To a stirred solution of C3-boc-Tz (80 mg, 0.254 mmoL, 1eq.) in anhydrous CH_2Cl_2 (20 mL) was added TFA (0.2 mL, 2.54 mmoL, 10 eq.) dropwise at 0 ^{o}C under N_2 atmosphere. After 15 min, the reaction mixture was allowed to warm to RT. After 5 h of stirring at RT, the reaction mixture was concentration *in vacuo* on a rotary evaporator. The traces of TFA were removed from the resultant mixture by evaporating it with toluene (10 mL × 3) under reduced pressure *in vacuo* followed by subjecting the mixture to column chromatography on silica gel using CH_2Cl_2/MeOH (9:1) eluent to afford zwitter ionic C3Tz as white solid (32 mg, 58% yield, R_f 0.27 in CH_2Cl_2/MeOH 9:1). The TLC of the reaction was visualized using ninhydrin staining solution. **^{1}H NMR** (500 MHz, CD_3OD) δ 4.13 (q, *J* = 9.4 Hz, 2H), 3.93 (t, *J* = 6.4 Hz, 1H), 3.25 (td, *J* = 6.9, 2.5 Hz, 2H), 3.08 (dd, *J* = 6.4, 2.2 Hz, 2H), 2.32 (t, *J* = 7.4 Hz, 2H), 1.80 (p, *J* = 7.2 Hz, 2H). **^{13}C NMR** (126 MHz, CD_3OD) δ 176.97, 173.46, 67.25, 54.05, 39.77, 36.51, 32.31, 25.83. **^{13}C DEPT-135** (126 MHz, CD_3OD) δ 67.25, *54.05*, *39.77*, *36.52*, *32.31*, *25.84* (Negative signals are denoted *italics*). **HRMS** (QTOF MS ESI+) *m/z* calcd. for $C_8H_{14}N_2O_3S$ $[M+H]^+$ 219.0803, found 219.0811.

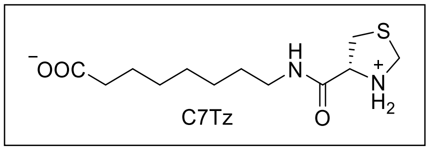

Following the procedure described above for the synthesis of C3Tz, compound C7Tz was prepared from C7-boc-Tz (29 mg, 0.077 mmoL, 1 eq.) and TFA (71 µL, 0.9 mmoL, 12 eq.). The reaction was continued for 12 h, and the crude obtained after the work up of the reaction was subjected to column chromatography on silica gel using CH_2Cl_2/MeOH (9:1) eluent to afford C7Tz as zwitter ionic white solid (13.8 mg, 81%, R_f 0.3 in CH_2Cl_2/MeOH 9:1). The TLC of the reaction was visualized using ninhydrin staining solution. **^{1}H NMR** (500 MHz, CD_3OD) δ 4.19 – 4.05 (m, 2H), 3.91 (t, J = 6.4 Hz, 1H), 3.21 (dp, J = 13.4, 6.7 Hz, 2H), 3.07 (d, J = 6.5 Hz, 2H), 2.28 (t, J = 7.4 Hz, 2H), 1.60 (q, J = 7.1 Hz, 2H), 1.52 (t, J = 6.9 Hz, 2H), 1.42 – 1.29 (m, 6H). **^{13}C NMR** (126 MHz, $CDCl_3$) δ 177.85, 173.20, 67.24, 54.05, 40.32, 36.57, 35.05, 30.32, 30.11, 30.00, 27.74, 26.03. **HRMS** (QTOF MS ESI+) *m/z* calcd. for $C_{12}H_{22}N_2O_3S$ $[M+H]^+$ 275.1429, found 275.1434.

Supplementary Fig. 8. Synthesis of C2Tz, C4Tz and C5Tz lipoic acid analogs.

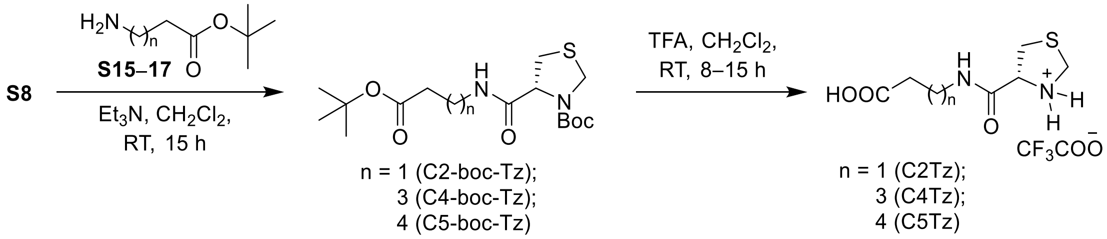

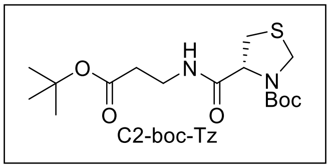

To a stirred solution of **S8** (0.618 gm, 1.54 mmoL, 1eq.) in anhydrous CH_2Cl_2 (10 mL) was added a solution of **S15** (0.225 mg, 1.54 mmoL, 1 eq.) in anhydrous CH_2Cl_2 (5 mL) followed by the addition of Et_3N (0.4 mL, 3.09 mmoL, 2 eq.) at RT under N_2 atmosphere. After 15 h, the reaction mixture was concentrated *in vacuo* on a rotary evaporator and the resultant crude was diluted with EtOAc (40 mL) and 10% citric acid solution in water (20 mL). The two layers were separated, and the aqueous layer was extracted with EtOAc (30 mL × 3). The organic layers were combined and washed with brine solution (30 mL), dried over Na_2SO_4, and concentrated *in vacuo*. The resultant crude mixture was subjected to column chromatography on silica gel using hexane/EtOAc (7:3) eluent to afford C2-boc-Tz as colourless oil (0.204 gm, 37%, R_f 0.41 in hexane/EtOAc 7:3). The TLC of the reaction was visualized using

ninhydrin staining solution. **^{1}H NMR** at 353K (500 MHz, DMSO-d_6) δ 7.69 (d, *J* = 6.8 Hz, 1H), 4.62 (d, *J* = 9.1 Hz, 1H), 4.52 (dd, *J* = 7.4, 4.6 Hz, 1H), 4.32 (d, *J* = 9.0 Hz, 1H), 3.30 (tdd, *J* = 12.8, 10.0, 5.8 Hz, 3H), 3.04 (dd, *J* = 11.5, 4.6 Hz, 1H), 2.37 (t, *J* = 6.9 Hz, 2H), 1.41 (d, *J* = 4.2 Hz, 18H). **^{13}C NMR** at 353K (126 MHz, DMSO-d_6) δ 170.09, 169.63, 152.58, 79.80, 79.62, 61.97, 48.74, 34.75, 34.73, 33.81, 27.64, 27.48. **^{13}C 135 DEPT** at 353K (126 MHz, DMSO-d_6) δ 61.97, *48.74*, *34.73*, *33.86*, 27.65, 27.49 (Negative signals are denoted in *italics*). **HRMS** (QTOF MS ESI+) *m/z* calcd. for $C_{16}H_{28}N_2O_5S$ $[M+Na]^+$ 383.1617, found 383.1620.

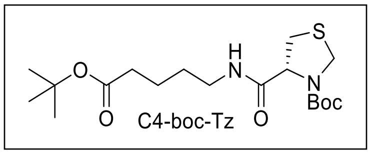

Following the procedure described above for the synthesis of C2-boc-Tz, compound C4-boc-Tz was prepared from **S8** (0.276 gm, 0.69 mmoL, 1eq.), **S16** (0.119 gm, 0.69 mmoL, 1eq.) and Et_3N (0.2 mL, 1.38 mmoL, 2 eq.). The crude obtained after the work up of the reaction was subjected to column chromatography on silica gel using hexane/EtOAc (3:2) eluent to afford C4-boc-Tz as white solid (0.167 gm, 62% yield, R_f 0.46 in hexane/EtOAc 3:2). **^{1}H NMR** at 353K (500 MHz, DMSO-d_6) δ 7.67 (s, 1H), 4.63 (d, *J* = 9.0 Hz, 1H), 4.52 (dd, *J* = 7.4, 4.7 Hz, 1H), 4.36 (d, *J* = 9.1 Hz, 1H), 3.31 (dd, *J* = 11.5, 7.3 Hz, 1H), 3.17 – 3.06 (m, 2H), 3.03 (d, *J* = 4.7 Hz, 1H), 2.19 (t, *J* = 7.1 Hz, 2H), 1.57 – 1.44 (m, 4H), 1.42 (s, 18H). **^{13}C NMR** at 353K (126 MHz, DMSO-d_6) δ 171.58, 169.37, 152.49, 79.59, 78.93, 62.00, 48.72, 37.96, 34.17, 28.05, 27.58, 27.44, 21.58. **^{13}C DEPT-135** at 353K (126 MHz, DMSO-d_6) δ 61.99, *48.72*, *37.95*, *34.16*, *28.04*, 27.58, 27.44, *21.57* (Negative signals are denoted in *italics*). **HRMS** (QTOF MS ESI+) *m/z* calcd. for $C_{18}H_{32}N_2O_5S$ $[M+Na]^+$ 411.1930, found 411.1924.

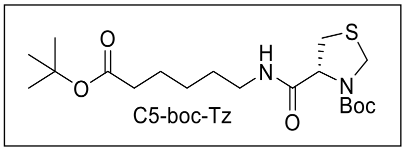

Following the procedure as described above for the synthesis of C2-boc-Tz, compound C5-boc-Tz was prepared from **S8** (0.767 gm, 1.9 mmoL, 1 eq.), **S17** (0.360 mg, 1.9 mmoL, 1 eq.) and Et_3N (0.5 mL, 3.8 mmoL, 2 eq.). The crude mixture obtained after the work up of the reaction was subjected to column chromatography on silica gel using hexane/EtOAc (7:3) eluent to afford C5-boc-Tz as colourless oil (0.523 gm, 68%, R_f 0.5 in hexane/EtOAc 7:3). The TLC of the reaction was visualized using ninhydrin staining solution. **^{1}H NMR** at 353K (500 MHz, DMSO-d_6) δ 7.64 (s, 1H), 4.63 (d, *J* = 9.0 Hz, 1H), 4.52 (dd, *J* = 7.4, 4.7 Hz, 1H), 4.36 (d, *J* = 9.0 Hz, 1H), 3.30 (dd, *J* = 11.6, 7.3 Hz, 1H), 3.10 (td, *J* = 13.4, 6.6 Hz, 2H), 3.03 (d, *J* = 4.7 Hz,

1H), 2.17 (t, *J* = 7.3 Hz, 2H), 1.53 (q, *J* = 7.4 Hz, 2H), 1.42 (s, 18H), 1.30 (ddd, *J* = 14.1, 7.6, 5.5 Hz, 2H). **^{13}C NMR** at 353K (126 MHz, DMSO-d_6) δ 171.64, 169.34, 152.50, 79.60, 78.91, 62.00, 48.73, 38.18, 34.44, 33.96, 28.25, 27.59, 27.45, 25.32, 23.87. **^{13}C DEPT-135** at 353K (126 MHz, DMSO-d_6) δ 62.00, *48.74*, *38.18*, *34.44*, *33.95*, *28.26*, 27.60, 27.42, *25.33*, *23.87* (Negative signals are denoted in *italics*). **HRMS** (QTOF MS ESI+) *m/z* calcd. for $C_{19}H_{34}N_2O_5S$ $[M+H]^+$ 403.2267, found 403.2272.

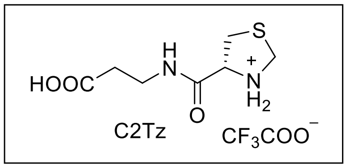

To a stirred solution of C2-boc-Tz (0.174 gm, 0.482 mmoL, 1 eq.) in anhydrous CH_2Cl_2 (20 mL) was added TFA (0.4 mL, 5.79 mmoL, 12 eq.) dropwise at 0 °C under N_2 atmosphere. After 15 min, the reaction mixture was allowed to warm to RT. After 12 h of stirring, the reaction mixture was concentration *in vacuo* on a rotary evaporator. The traces of TFA were removed from the resultant mixture by evaporating it with toluene (10 mL × 3) under reduced pressure *in vacuo* followed by subjecting the mixture to column chromatography on silica gel using CH_2Cl_2/MeOH (9:1) eluent to afford C2Tz TFA salt as white solid (85 mg, 55%, R_f 0.14 in CH_2Cl_2/MeOH 9:1). The TLC of the reaction was visualized using ninhydrin staining solution. **^{1}H NMR** (500 MHz, CD_3OD) δ 4.17 (d, *J* = 9.5 Hz, 1H), 4.10 (d, *J* = 9.5 Hz, 1H), 4.06 – 4.00 (m, 1H), 3.46 (dt, *J* = 9.1, 4.6 Hz, 2H), 3.11 (qd, *J* = 10.5, 6.4 Hz, 2H), 2.52 (t, *J* = 6.6 Hz, 2H). **^{13}C NMR** (126 MHz, CD_3OD) δ 175.44, 173.06, 66.93, 53.68, 36.35, 36.16, 34.63. **^{13}C 135-DEPT** (126 MHz, CD_3OD) δ 66.90, *53.66*, *36.32*, *36.14*, *34.60* (Negative signals are denoted *italics*). **^{19}F NMR** (376 MHz, CD_3OD) δ -76.99. **HRMS** (QTOF MS ESI+) *m/z* calcd. for $C_7H_{13}N_2O_3S^+$ $[M^+]$ 205.0641, found 205.0650.

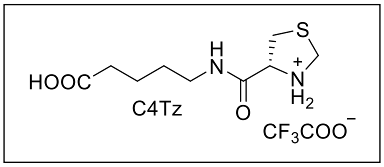

Following the procedure as described above for the synthesis of C2Tz, compound C4Tz was prepared from C4-boc-Tz (0.29 gm, 0.74 mmoL, 1 eq.) and TFA (0.7 mL, 8.9 mmoL, 12 eq.). The reaction was continued for 15 h and the crude obtained after the work up of the reaction was subjected to column chromatography on silica gel using CH_2Cl_2/MeOH (9:1) eluent to afford C4Tz TFA salt as white solid (0.254 gm, 97%, R_f 0.23 in CH_2Cl_2/MeOH 9:1). The TLC of the reaction was visualized using ninhydrin staining solution. **^{1}H NMR** (500 MHz, CD_3OD) δ 4.46 – 4.33 (m, 3H), 3.47 (dd, *J* = 11.6, 7.3 Hz, 1H), 3.27 (t, *J*

= 6.7 Hz, 2H), 3.21 (dd, *J* = 11.7, 7.0 Hz, 1H), 2.33 (t, *J* = 7.0 Hz, 2H), 1.68 – 1.53 (m, 4H). **^{13}C NMR** (126 MHz, CD_3OD) δ 177.18, 168.39, 64.46, 50.51, 40.39, 34.85, 34.32, 29.67, 23.20. **^{13}C 135-DEPT** (126 MHz, CD_3OD) δ 64.46, *50.50, 40.39, 34.85, 34.33, 29.68, 23.21* (Negative signals are denoted in *italics*). **^{19}F NMR** (376 MHz, CD_3OD) δ -76.97. **HRMS** (QTOF MS ESI+) *m/z* calcd. for $C_9H_{17}N_2O_3S^+$ [M^+] 233.0954, found 233.0962.

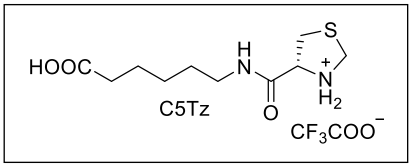

Following the procedure as described above for the synthesis of C2Tz, compound C5Tz was prepared from C5-boc-Tz (0.416 gm, 1.03 mmoL, 1 eq.) and TFA (0.9 mL, 12.4 mmoL, 12 eq.). The reaction was continued for 8 h and the crude obtained after the work up of the reaction was subjected to column chromatography on silica gel using CH_2Cl_2/MeOH (9:1) eluent to afford C5Tz TFA salt as white solid (0.274 gm, 74%, R_f 0.27 in CH_2Cl_2/MeOH 9:1). The TLC of the reaction was visualized using ninhydrin staining solution. **^{1}H NMR** (500 MHz, CD_3OD) δ 4.18–4.08 (m, 2H), 3.92 (t, *J* = 6.5 Hz, 1H), 3.21 (td, *J* = 6.9, 5.3 Hz, 2H), 3.07 (d, *J* = 6.5 Hz, 2H), 2.29 (t, *J* = 7.4 Hz, 2H), 1.62 (p, *J* = 7.4 Hz, 2H), 1.54 (p, *J* = 7.2 Hz, 2H), 1.37 (ddt, *J* = 13.0, 9.7, 6.1 Hz, 2H). **^{13}C NMR** (126 MHz, CD_3OD) δ 177.51, 173.19, 67.23, 54.05, 40.16, 36.56, 34.84, 30.07, 27.41, 25.70. **^{13}C 135-DEPT** (126 MHz, CD_3OD) δ 67.23, *54.05, 40.16, 36.56, 34.85, 30.07, 27.41*, *25.71* (Negative signals are denoted in *italics*). **^{19}F NMR** (376 MHz, CD_3OD) δ -76.87. HRMS (QTOF MS ESI+) *m/z* calcd. for $C_{10}H_{19}N_2O_3S^+$ [M^+] 247.1111, found 247.1125.

4. Kinetic study of the conjugation reaction of IBH adducts with cysteamine

<u>Procedure for the conjugation reaction of IBH adducts **1A**–**C** with cysteamine in sodium phosphate buffer solutions of different pH (6–8).</u>

To a solution of IBH adduct **1A**/**1B**/**1C** (25 µL, 2 mM stock in CH_3CN, final conc. 50 µM) in sodium phosphate buffer (575 µL, 50 mM) of desired pH (6, 7 or 8) and CH_3CN (375 µL) was added a solution of cysteamine hydrochloride (25 µL, 2 mM stock in 50 mM sodium phosphate buffer, final conc. 50 µM) at RT. The reaction mixture was mixed by pipetting up and down. Aliquots (150 µL) from the mixture were periodically withdrawn and subjected to HPLC. Peaks were collected and characterized by HRMS. The complete characterization (^{1}H and ^{13}C NMR) of

the products **2A**/**2B**/**2C** formed in these reactions are provided above in section 2. The HPLC traces of these experiments are provided in Fig. 3a of the main text. In addition to this, similar experiments were performed by quenching the reaction mixture before injecting to the HPLC. Specifically, in these experiments, aliquots (150 µL) from the reaction mixture were withdrawn at different time points and quenched with 1.5 µL of 1M HCl solution before subjecting them to HPLC analysis. The results of these experiments, as depicted by the HPLC traces below, demonstrate no change in the reaction profiles whether the aliquots are injected into the HPLC with or without quenching. The experiment was performed two times independently, and yielded similar results each time.

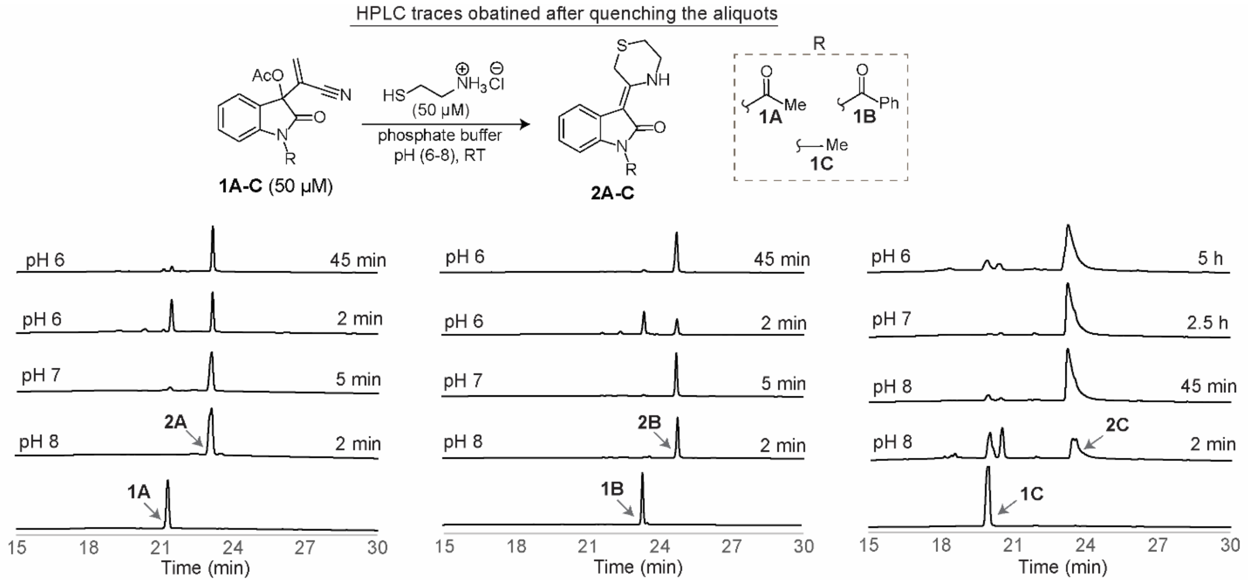

<u>Procedure for the conjugation reaction of **1A**/**1C** with cysteamine in the presence of *N*-acetyl cysteine (NAC) in sodium phosphate buffers at different pH (6, 6.5 and 7).</u>

A solution of IBH adduct **1A**/**1C** (10 µL, 50 mM stock in CH_3CN, final conc.1 mM, 1eq.) was added to a solution containing NAC (10 µL, 50 mM stock in 50 mM sodium phosphate buffer, 1 eq.) and cysteamine hydrochloride (10 µL, 50 mM stock in 50 mM sodium phosphate buffer 1 eq.) in CH_3CN (190 µL)/sodium phosphate buffer (280 µL, 50 mM). The reaction mixture was mixed by pipetting up and down, and incubated at RT. After 40 min (for **1A**) and 4 h (for **1C**), the reaction mixture was spiked with internal standard, 2-oxindole (5 µL, 50 mM stock in CH_3CN) followed by injecting 5 µL of the reaction mixture to the HPLC. For quantitation, a separate HPLC

experiment was performed wherein 10 µL solution of product (**2A** or **2C**) from its 50 mM stock in CH_3CN was added to CH_3CN/sodium phosphate buffer (2:3) solution to give a final conc. of 1 mM, and this solution was subjected to HPLC analysis using 2-oxindole as the internal standard as described above to give the area under peak for the product corresponding to 100% conversion. The HPLC traces of the reactions and the bar graph depicting the percentage product formed in these reactions are provided in Supplementary Fig. 9. All the reactions were performed in triplicate.

Supplementary Fig. 9. Conjugation reaction of IBH adducts with cysteamine in the presence NAC at different pH. (**a**) Formation of product **2A** from **1A** and (**b**) Formation of **2C** from **1C.** The HPLC traces depicting the formation of product are shown on the left and the bar graph depicting the quantitation of product formed in these reactions on the right. The reactions were performed by incubating equimolar concentrations (1 mM each) of **1A**/**1C**, cysteamine and NAC in sodium phosphate buffer (pH 6–7) at RT. The detailed procedure is provided above. All experiments were performed three times independently, and yielded similar results each time.

a

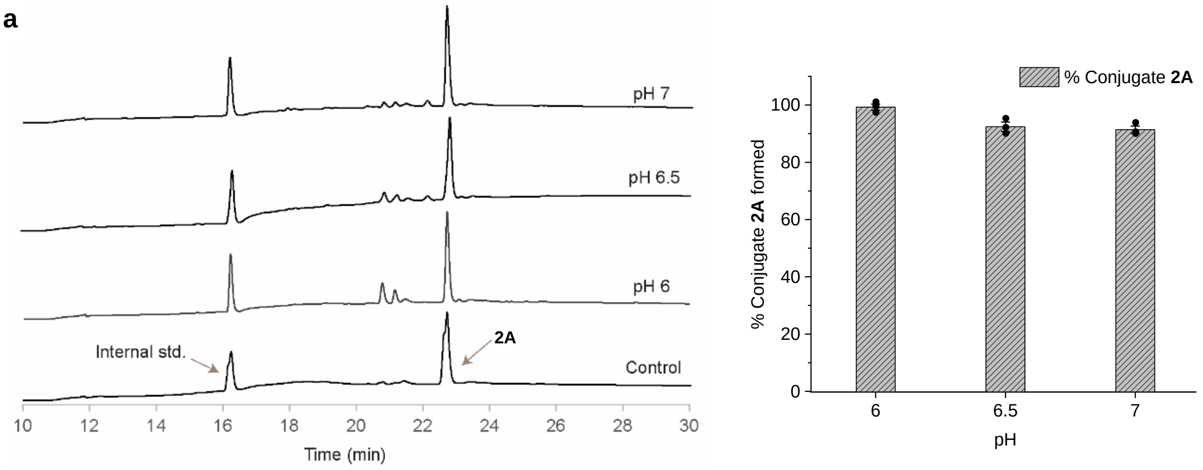

b

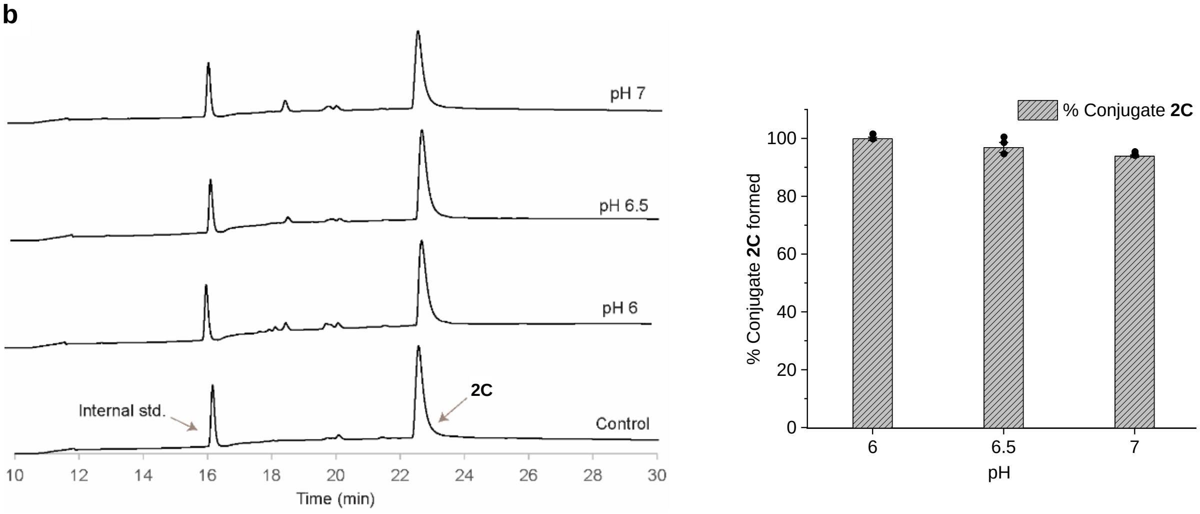

Procedure for the conjugation reaction of **1A**/**1C** with cysteamine in the presence of different amino acids (alanine, arginine, asparagine, aspartic acid, *N*-acetyl cysteine, glutamine, glutamic acid, glycine, histidine, isoleucine, leucine, lysine, methionine, phenylalanine, proline, serine, threonine, tryptophan, tyrosine, valine)

A solution of IBH adduct **1A**/**1C** (10 µL, 50 mM stock in CH_3CN, final conc. 1 mM, 1eq.) was added to a solution containing amino acid (10 µL, 50 mM stock, 1 eq.) and cysteamine hydrochloride (10 µL, 50 mM stock in buffer, 1 eq.) in CH_3CN (190 µL)/sodium phosphate buffer (280 µL, 50 mM, pH 6.5). *Note:* The stocks of all the amino acids were prepared in 50 mM sodium phosphate buffer of pH 6.5, except for tyrosine which was prepared in 1N HCl. The reaction mixture was mixed by pipetting up and down, and incubated at RT. After 40 min (for **1A**) or 4 h (for **1C**), the reaction mixture was spiked with internal standard 2-oxindole (5 µL, 50 mM stock in CH_3CN), followed by injecting 5 µL of the reaction mixture to the HPLC. A similar procedure was followed for the IBH reaction with cysteamine without the amino acids; 10 µL of buffer was added in place of the amino acid solution, followed by the HPLC analysis using an internal standard to give the area under peak for the product corresponding to 100% conversion. The HPLC traces of the experiments are provided in Supplementary Fig. 10a. The bar graph depicting the percentage of product formed in these reactions with **1A** is depicted in Fig. 3b of the main text and with **1C** in Supplementary Fig. 10b. All the reactions were performed in triplicate.

Supplementary Fig. 10. Conjugation reaction of IBH adducts with cysteamine in the presence of different amino acids. (**a**) HPLC traces depicting the formation of **2A** (left panel) and **2C** (right panel) from **1A** and **1C**, respectively. (**b**) Bar graph depicting the quantitation of product **2C** formed in these reactions. The bar graph for the quantitation of product **2A** is provided in Fig. 3b of the main text. The reactions were performed by incubating equimolar concentrations (1 mM each) of **1A**/**1C**, cysteamine, and other amino acids in sodium phosphate buffer (pH 6.5) at RT. The detailed procedure is provided above. All experiments were performed three times independently, and yielded similar results each time.

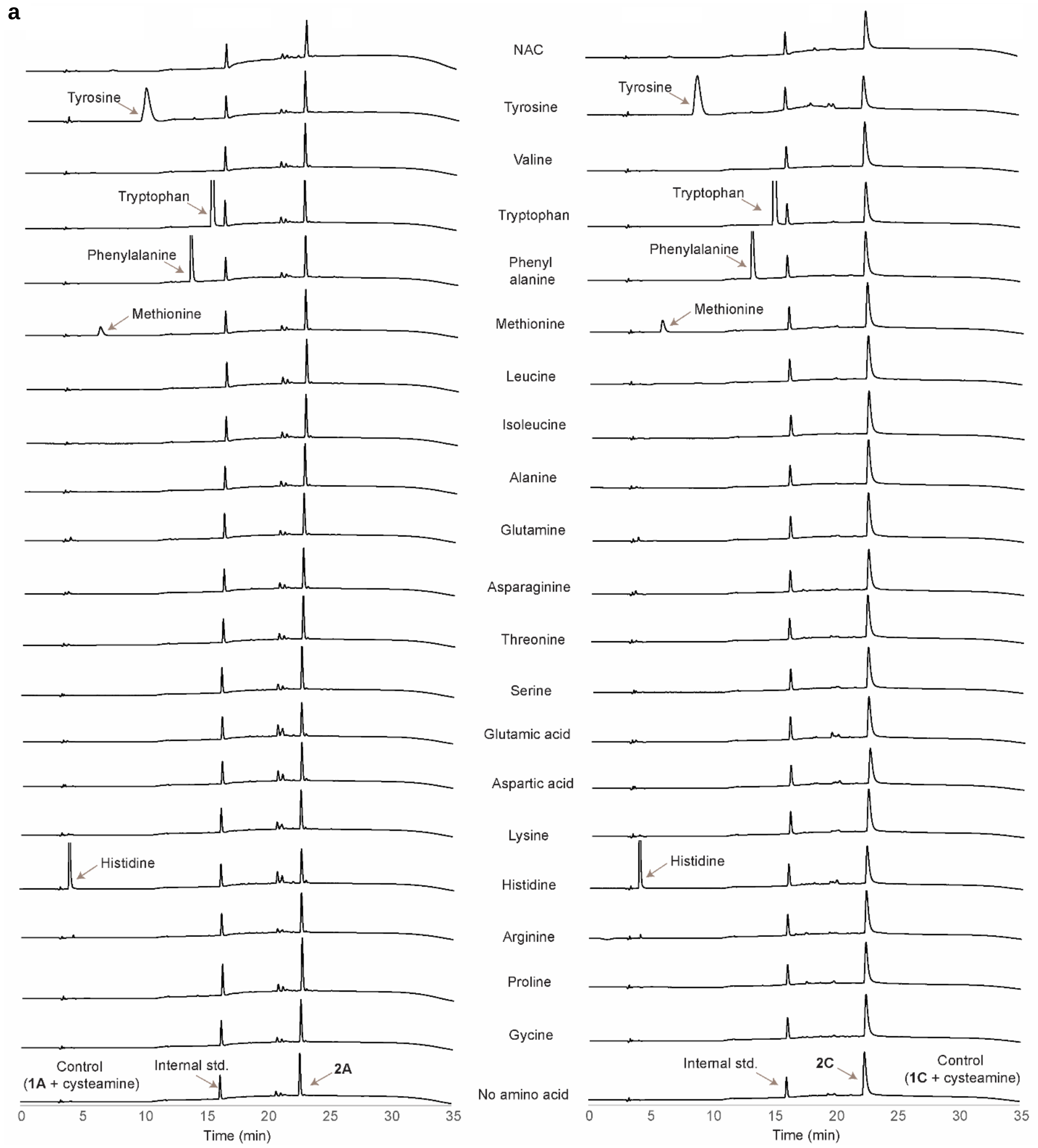

b

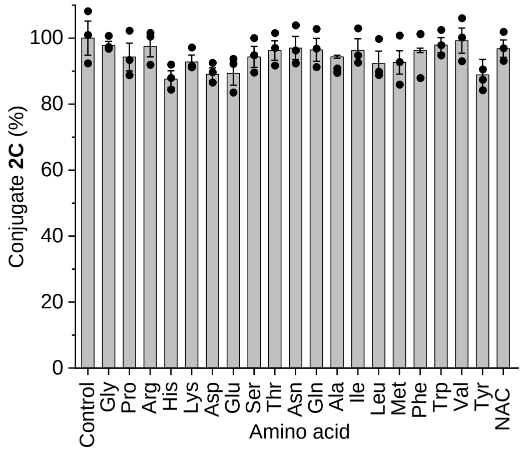

<u>Procedure for the conjugation reaction of **1A** with cysteamine in the presence of competing thiols at pH (6.5 and 8).</u>

A solution of IBH adduct **1A** (10 µL, 50 mM stock in CH_3CN, final conc. 1 mM, 1eq.) was added to a solution containing competing thiol NAC/1-hexanethiol (10 µL, 50–250 mM stock, 1–5 eq.) and cysteamine hydrochloride (10 µL, 50 mM stock in buffer, 1 eq.) in CH_3CN (190 µL)/sodium phosphate buffer (280 µL, 50 mM, pH 6.5 or 8). The stock of NAC was prepared in 50 mM reaction buffer and that of 1-hexanethiol in CH_3CN. The reaction mixture was mixed by pipetting up and down, and incubated at RT. Aliquots (2 µL) from the mixture were periodically withdrawn and subjected to LC-MS. The LC traces were recorded at 254 nm. Peaks were characterized by HRMS. A similar procedure was followed for the IBH reaction with cysteamine without the competing thiols; 10 µL of stock solvent was added in place of the competing thiol solution. The results obtained in the experiment are summarized in Supplementary Fig. 11 below.

Supplementary Fig. 11. Conjugation reaction of IBH adduct 1A with cysteamine in the presence of thiols at different pH. (**a**) Top: Schematic representation of the general reaction. The structures of different thiols are shown on the right and the estimated pKa values of their sulfhydryl groups are mentioned within parentheses. Bottom: Table summarizing the screened reaction conditions. (**b**) The HPLC traces depicting the formation of product under different reaction conditions. Traces obtained pH 8 and 6.5 are shown on the left and right, respectively. The reactions were performed in sodium phosphate buffer (pH 6.5–8) at RT and the detailed procedure is provided above. HRMS (ESI) analysis of **2A**, *m/z* calcd. for $C_{14}H_{14}N_2O_2S$ $[M + H]^+$ 275.0854, found 275.0852; PDT-COMP of NAC, *m/z* calcd. for $C_{18}H_{17}N_3O_5S$ $[M + H]^+$ 388.0967, found 388.0962; PDT-COMP of 1-hexanethiol, *m/z* calcd. for $C_{19}H_{22}N_2O_2S$ $[M + H]^+$ 343.1480, found 343.1478.

a

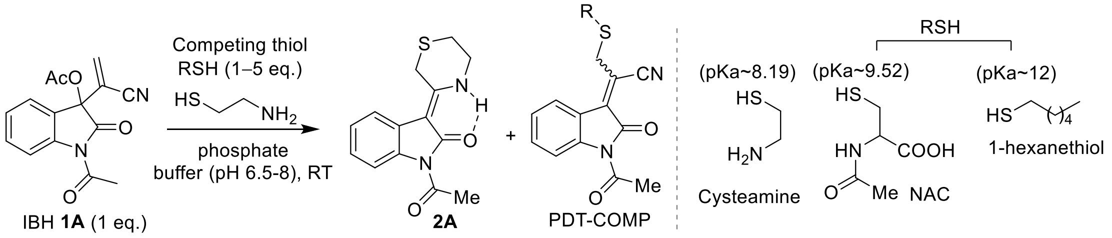

S. No.	pH	IBH 1A (eq.)	Cysteamine (eq.)	NAC (eq.)	1-hexanethiol (eq.)	Product 2A (%)
1		1	1	0	-	100
2		1	1	1	-	91
3	8	1	1	5	-	76
4		1	1	-	1	96
5		1	1	-	5	92
6		1	1	0	-	100
7		1	1	1	-	97
8	6.5	1	1	5	-	90
9		1	1	-	1	97
10		1	1	-	5	97

b

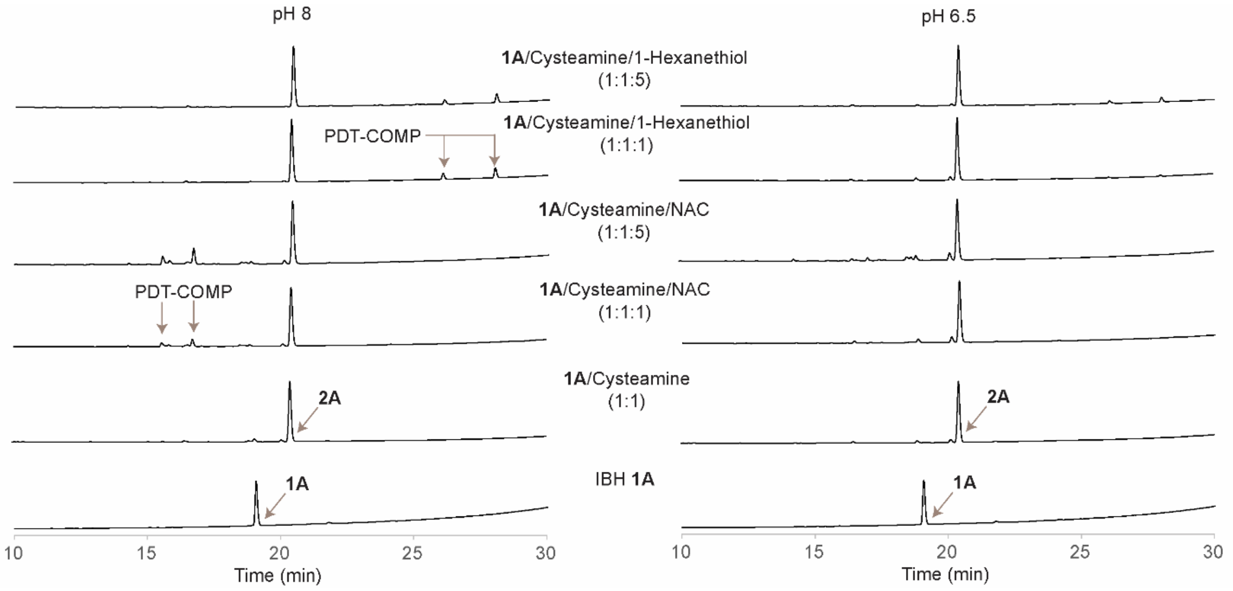

Procedure for measuring rates of **2A**/**2B**/**2C** formation in buffer solutions of different pH (6–8)

To a solution of IBH adduct **1A**/**1B**/**1C** (25 µL, 2 mM stock in CH_3CN, final conc. 50 µM, 1 eq.) in sodium phosphate buffer (575 µL, 50 mM) of desired pH (6, 7, 7.5 or 8) and CH_3CN (375 µL) was added a solution of cysteamine hydrochloride (25 µL, 2 mM in 50 mM sodium phosphate buffer, final conc. 50 µM, 1 eq.) at RT. The reaction mixture was mixed, and the UV absorbance at 350 nm corresponding to the product was recorded with time. The second-order rate constants (k) were determined by fitting the rate equation (1) with two reactants of equimolar concentrations (50 µM) to the UV absorbance values obtained at 350 nm (A_t). The absorbance at 350 nm of isolated products **2A**/**2B**/**2C** (final conc. 50 µM) was considered 100% conversion and the A_t values obtained with time during the kinetics were normalized to % conversions accordingly. The control spectra acquired for the reactants (**1A**/**1B**/**1C**, 50 µM) and their corresponding cysteamine conjugate products (**2A**/**2B**/**2C**, 50 µM) at RT are provided in Supplementary Fig. 12a. The kinetic plots obtained with **1A** at different pH values up to 120 s are provided in Fig. 3c of main text and their corresponding extended plots up to 600 s are provided in Supplementary Fig. 12b, in addition to the kinetic plots obtained with **1B** and **1C**. A table summarizing the rate constants observed in these reactions are summarized in Supplementary Fig. 12c.

$$A_t = \frac{A_{max} \times k \times t \times 0.00005}{1 + k \times t \times 0.00005} \qquad \text{equation (1)}$$

where,

$A_t = Absorbance\ at\ time\ t$

$A_{max} = The\ maximum\ absorbance\ intensity\ value\ obtained\ at\ t_{max}$

$k = second\ order\ rate\ constant$

Supplementary Fig. 12. Second-order reaction kinetics between IBH adducts and cysteamine. (**a**) UV-Vis spectra of reactants (**1A**/**1B**/**1C**, 50 μM) and products (**2A**/**2B**/**2C**, 50 μM). (**b**) Kinetic traces of the reactions recorded in buffer of different pH. (**c**) Table summarizing the calculated second-order rate constants. The reactions were performed by incubating equimolar (50 μM) concentrations of **1A**/**1B**/**1C** and cysteamine in sodium phosphate buffer of different pH (6–8) values at RT followed by recording the A350 nm corresponding to the formation of product with time. Each data point in the kinetic trace is an average of three recordings, and the error bar corresponds to standard deviation value. All experiments were performed three times independently, and yielded similar results each time.

a

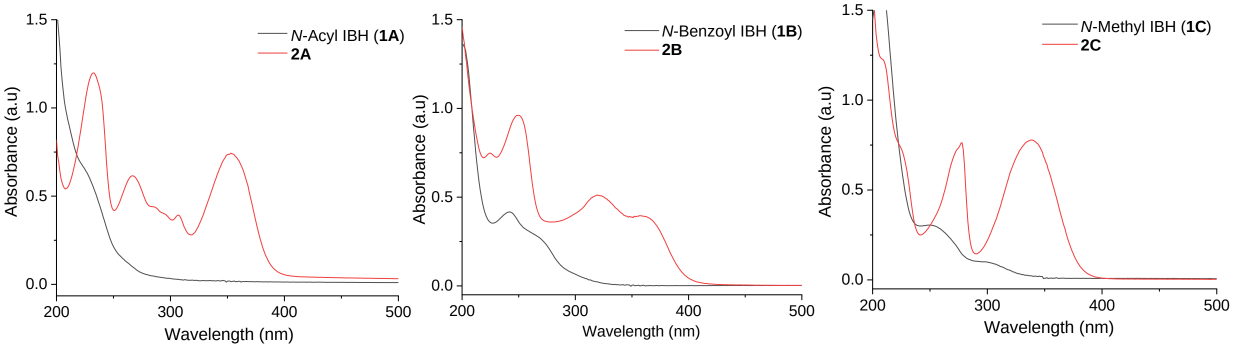

b

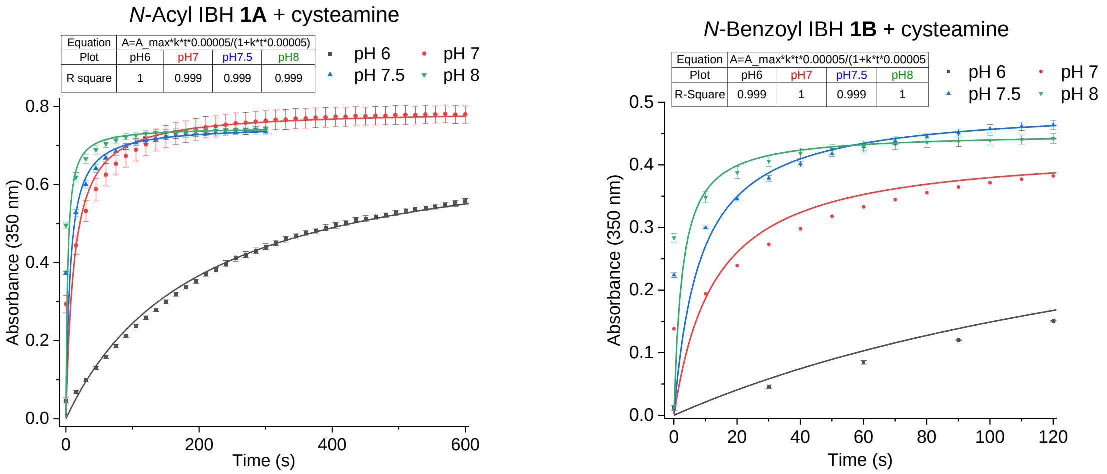

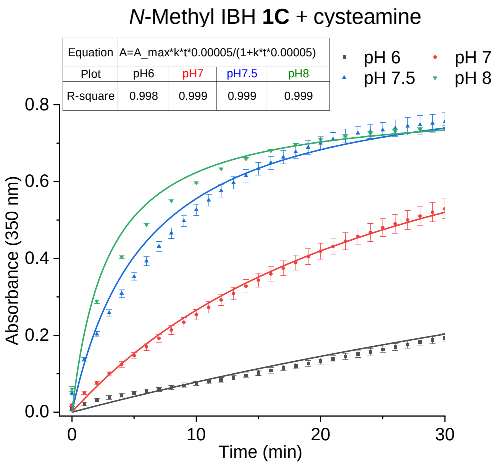

c

Table for the second-order rate constants (k $M^{-1}s^{-1}$) obtained in the reactions of **1A**–**C** (50 μM) with cysteamine (50 μM) at 25 °C in buffer of different pH values.

pH	1A	1B	1C
6	100.3 (±1.5)	87.8 (± 1.2)	5.7 (± 0.8)
7	1.7 (± 0.2) ×10^3	1.2 (± 0.07) × 10^3	16.0 (± 2.1)
7.5	3.1 (± 0.3) × 10^3	2.5 (± 0.2) × 10^3	65.3 (± 18.4)
8	7.6 (± 1.0) × 10^3	7.2 (± 0.2) × 10^3	72.5 (± 7.7)

Procedure for the conjugation reactions of IBH, CPO and CBT reagents with cysteamine in sodium phosphate buffer solutions of pH 7.

*Procedure for reaction of IBH **1A**/CPO/CBT with cysteamine:*

To a solution of IBH **1A**/CPO/CBT (25 µL, 2 mM stock in CH_3CN, final conc. 50 µM) in sodium phosphate buffer (575 µL, 50 mM, pH 7) and CH_3CN (375 µL) was added a solution of cysteamine hydrochloride (25 µL, 2 mM stock in 50 mM sodium phosphate buffer at pH 7, final conc. 50 µM) at RT. The reaction mixture was mixed by pipetting up and down. Aliquots (200 µL) from the mixture were withdrawn at different time points and quenched with 2 µL of 1M HCl solution before subjecting them to HPLC. Peaks were collected and characterized by HRMS. The HPLC traces of these experiments are provided in Supplementary Fig. 13b.

*Procedure for the competition reactions of IBH **1A** and CPO/CBT with cysteamine:*

To a solution of IBH **1A** (25 µL, 2 mM stock in CH_3CN, final conc. 50 µM) and CPO/CBT (25 µL, 2 mM stock in CH_3CN, final conc. 50 µM) in sodium phosphate buffer (575 µL, 50 mM, pH 7) and CH_3CN (350 µL) was added a solution of cysteamine hydrochloride (25 µL, 2 mM stock in 50 mM sodium phosphate buffer at pH 7, final conc. 50 µM) at RT. The reaction mixture was mixed by pipetting up and down. Aliquots (200 µL) from the mixture were withdrawn at different time points and quenched with 2 µL of 1M HCl solution before subjecting them to HPLC. Peaks were collected and characterized by HRMS. The HPLC traces of these experiments are provided in Supplementary Fig. 13b.

*Procedure for the competition reaction of IBH **1A**, CPO and CBT with cysteamine:*

To a solution of IBH **1A** (25 µL, 2 mM stock in CH_3CN, final conc. 50 µM), CPO (25 µL, 2 mM stock in CH_3CN, final conc. 50 µM) and CBT (25 µL, 2 mM stock in CH_3CN, final conc. 50 µM) in sodium phosphate buffer (575 µL, 50 mM, pH 7) and CH_3CN (325 µL) was added a solution of cysteamine hydrochloride (25 µL, 2 mM stock in 50 mM sodium phosphate buffer at pH 7, final conc. 50 µM) at RT. The reaction mixture was mixed by pipetting up and down. Aliquots (200 µL) from the mixture were withdrawn at different time points and quenched with 2 µL of 1M HCl solution before subjecting them to HPLC. Peaks were collected and characterized by HRMS. The HPLC traces of these experiments are provided in Supplementary Fig. 13b.

Supplementary Fig. 13. Comparison of the reactions of IBH, CPO and CBT 1,2-aminothiol-derivatization reagents with cysteamine. (**a**) Left: Schematic illustration of the reactions of different 1,2-aminothiol labelling reagents with cysteamine. Right: Schematic illustration of the competition reactions of IBH **1A** with cysteamine in the presence of CPO and CBT. (**b**) HPLC traces depicting the reaction progress at different time points obtained in either individual or one pot reactions of **1A**, CPO and CBT with cysteamine. All the conjugates were characterized by HRMS (ESI) analysis; **2A**, *m/z* calcd. for $C_{14}H_{14}N_2O_2S$ $[M+H]^+$ 275.0854, found 275.0852; PDT-CPO, *m/z* calcd. for $C_{13}H_{17}NOS$ $[M+H]^+$ 236.1109, found 236.1105; PDT-CBT, *m/z* calcd. for $C_{10}H_8N_2S$ $[M+H]^+$ 221.0207, found 221.0202. CPO was synthesized following protocol reported in the literature[8]. The NMR data of CPO is provided in Supplementary Fig. 92 of section 18.

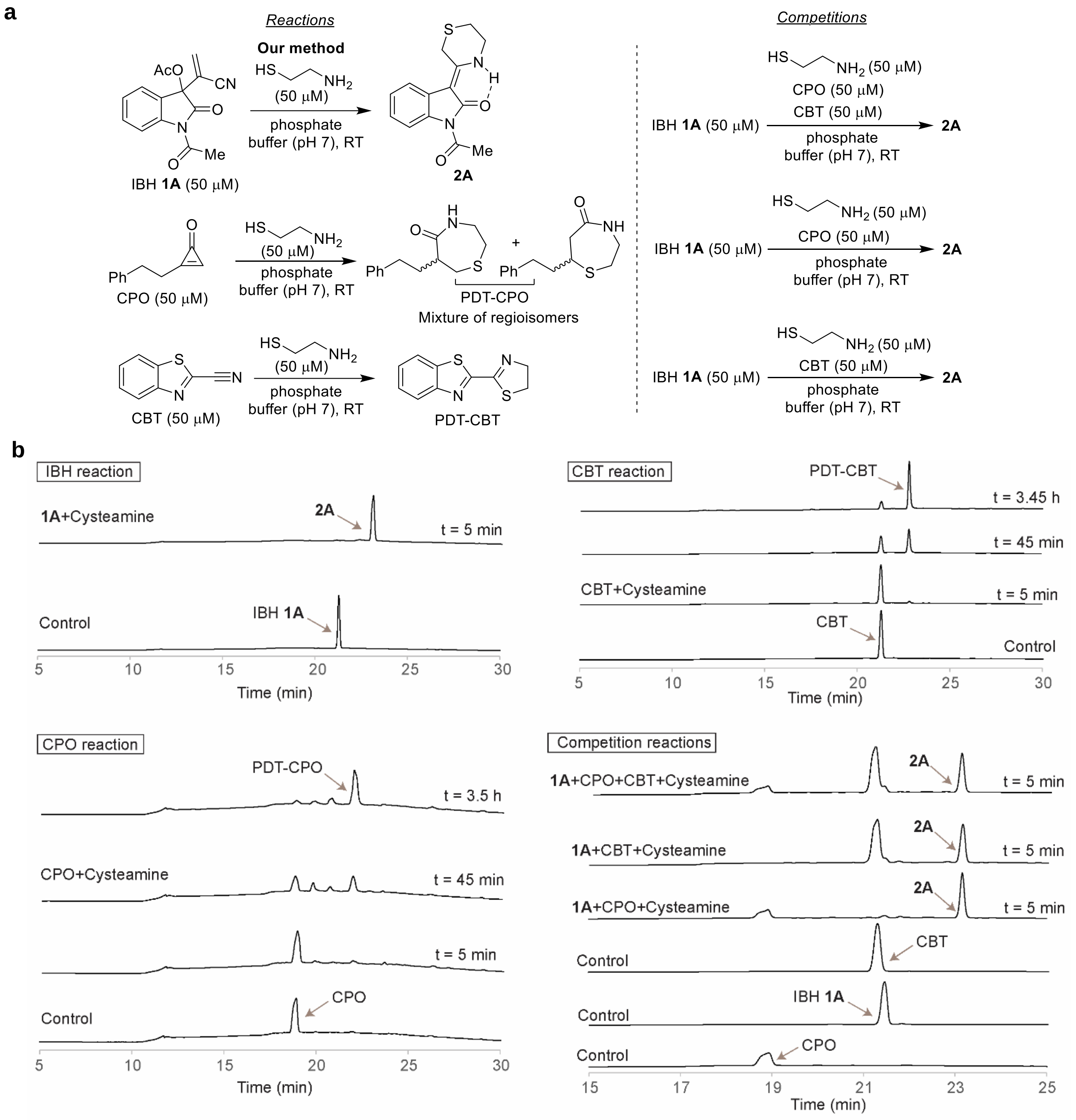

5. Stability studies of the bis-heterocyclic IBH-cysteamine conjugates

Stability at different pH:

A solution of bis-heterocyclic IBH-cysteamine conjugates, **2A** (40 µL, 50 mM in CH_3CN, final conc. 1 mM) or **2C** (40 µL, 50 mM stock in CH_3CN:DMSO 2/1, final conc. 1 mM) was added to a solution containing sodium phosphate buffer (1.2 mL, 50 mM) of different pH (4–9) and CH_3CN (760 µL). The resultant mixture was mixed by pipetting up and down, and incubated at RT for 5 d. Aliquots (300 µL) from the mixture were periodically withdrawn and subjected to HPLC. Each aliquot was spiked with 3 µL of internal standard 2-oxindole (50 mM in CH_3CN) before the HPLC injection. The area under peak for the conjugate at t = 0 was considered 100% conjugate, and this area was used for calculating the percentage of conjugate remaining at different time points. The HPLC traces of the reaction and the bar graph depicting the quantitation of conjugate remaining via HPLC are provided in Supplementary Fig. 14.

Stability in the presence of Cys:

To a solution of bis-heterocyclic IBH-cysteamine conjugates, **2A** (100 µL, 50 mM in CH_3CN, final conc. 1 mM) or **2C** (100 µL, 50 mM stock in CH_3CN:DMSO 99/1, final conc. 1 mM) in sodium phosphate buffer at pH 7 (2.0 mL, 50 mM) containing 1.9 mL of CH_3CN was added *L*-cysteine (1 mL, 50 mM stock in sodium phosphate buffer pH 7, final conc. 10 mM). The reaction mixture was mixed by pipetting up and down, and incubated at RT. Aliquots (300 µL) from the mixture were periodically withdrawn and subjected to HPLC. Each aliquot was spiked with 3 µL of internal standard 2-oxindole (50 mM in CH_3CN) before HPLC. The area under peak for the conjugate at t = 0 was considered 100% conjugate, and this area was used for calculating the percentage of conjugate remaining at different time points. The HPLC traces of the experiment are provided in Supplementary Fig. 15.

Stability in the presence of GSH:

To a solution of bis-heterocyclic IBH-cysteamine conjugates, **2A** (100 µL, 50 mM in CH_3CN, final conc. 1 mM) or **2C** (100 µL, 50 mM stock in CH_3CN:DMSO 99/1, final conc. 1 mM) in sodium phosphate buffer at pH 7 (2.9–2.0 mL, 50 mM) containing 1.9 mL of CH_3CN was added GSH (0.1–1 mL, 50 mM stock in sodium phosphate buffer pH 7, final conc. 1–10 mM). The reaction mixture was mixed by pipetting up and down, and incubated at RT for 4 d. Aliquots (300 µL) from

the mixture was periodically withdrawn and subjected to HPLC. Each aliquot was spiked with 3 µL of internal standard 2-oxindole (50 mM in CH_3CN) before HPLC. The area under peak for the conjugate at t = 0 was considered 100% conjugate, and this area was used for calculating the percentage of conjugate remaining at different time points. The HPLC traces of the reaction and the bar graph depicting the quantitation of conjugate remaining via HPLC are provided in Supplementary Fig. 16.

Supplementary Fig. 14. Stability of conjugation products at different pH. (**a**) **2A** and (**b**) **2C.** Conjugates were incubated in sodium phosphate buffer of different pH (4–8) followed by their quantitation at different time points using HPLC analysis. The HPLC traces obtained on the 2nd, 3rd and 5th day of incubation are shown on the left and the bar graphs depicting the percentage of conjugate remaining on those days on the right.

a

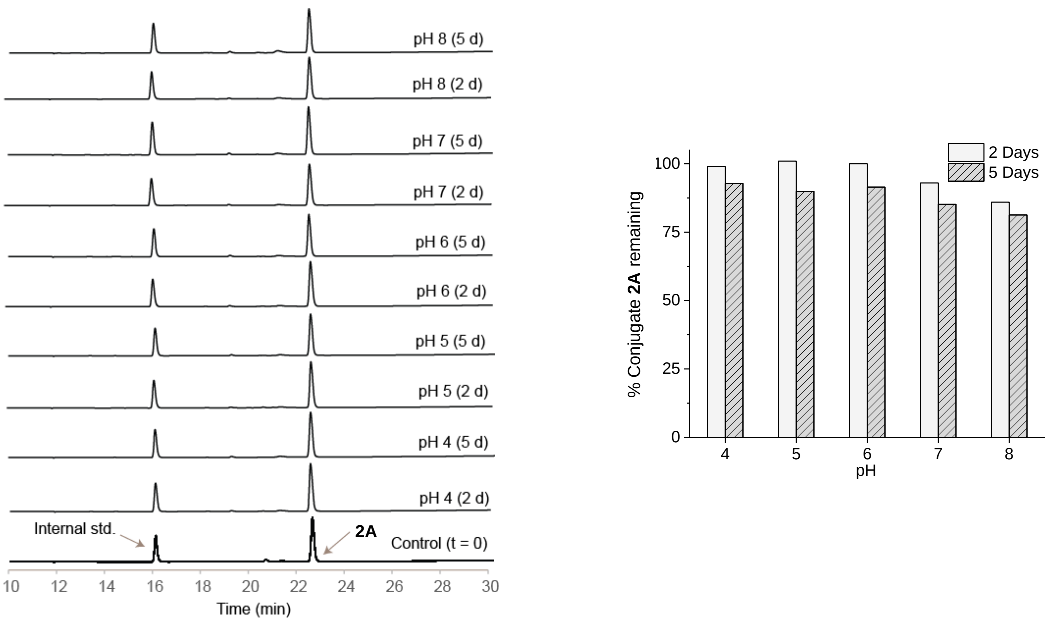

b

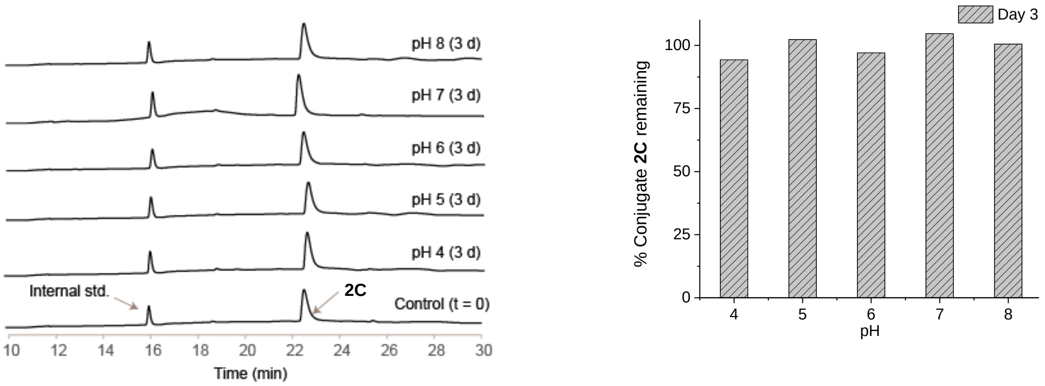

Supplementary Fig. 15. Stability of conjugation products in presence of cysteine (Cys). (**a**) **2A** and (**b**) **2C.** Conjugates were incubated in sodium phosphate buffer of pH 7 containing 10 mM Cys at RT. The stability of the conjugates was analysed by HPLC. The results showed no decomposition of conjugates **2A** and **2C** over the course of 1-2 days.

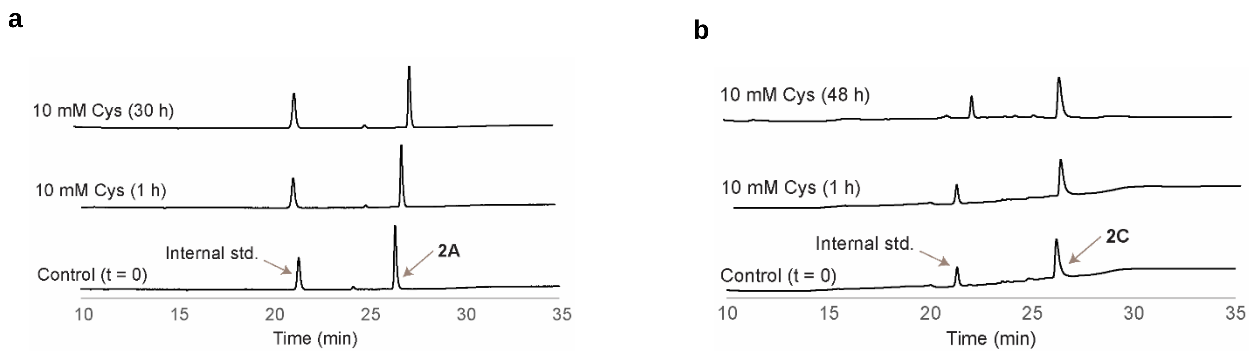

Supplementary Fig. 16. Stability of conjugation products in the presence of glutathione (GSH). (**a**) **2A** and (**b**) **2C**. Conjugates were incubated for 3 days with GSH (1–10 mM) in sodium phosphate buffer (pH 7) followed by their quantitation at different time points using HPLC analysis. The HPLC traces obtained on the 2nd and 3rd day of incubation are shown on the left and the bar graphs depicting the percentage of conjugate remaining on those days on the right.

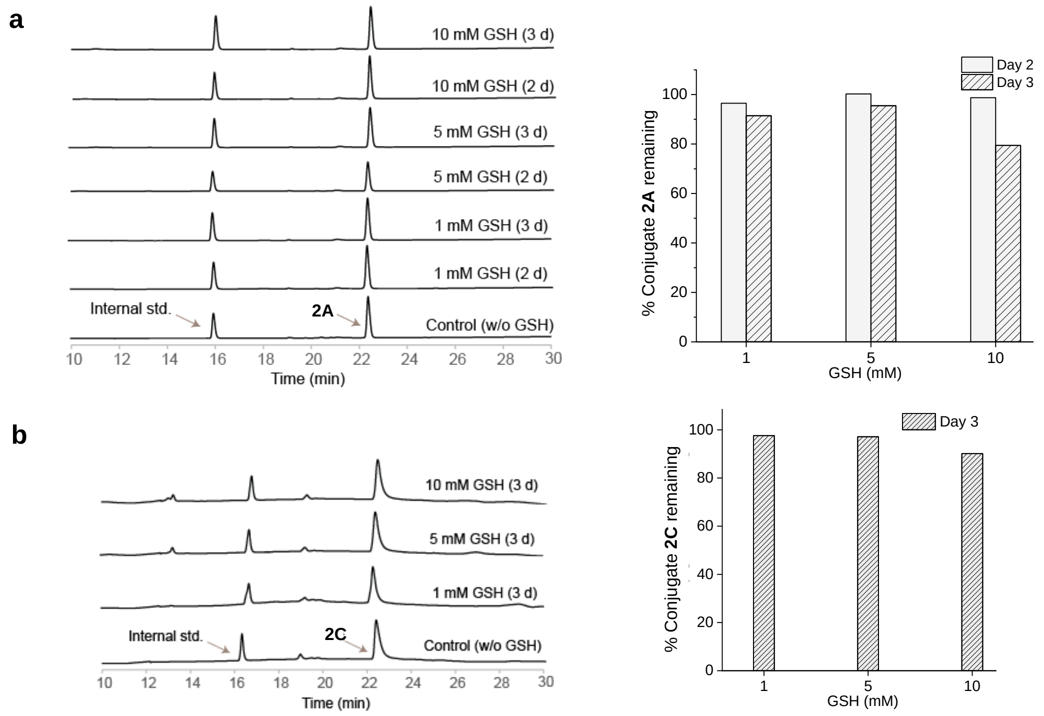

6. Recombinant production of *N*-Cys POI and His-TEV protease

Plasmid construction (cloning): Plasmids were constructed via site-directed mutagenesis by employing either substitution or insertion methods. The primers employed are summarized in Supplementary Table 1.

Supplementary Table 1. Sequence of primers employed for cloning.

Primer name	Sequence (5'-3')
EGFP-F	GAACCTGTACTTCCAGTGCTCGAGCATGGTGAG
EGFP-R	CTCACCATGCTCGAGCACTGGAAGTACAGGTTC
mCherry-F	TACTTCCAGTGCTCTATGGTGTCAAAAGG
mCherry-R	CCATAGAGCACTGGAAGTACAGGTTTTCG
MBPTEV-F	GAAAACCTGTATTTCCAGTGCTCTTCTATGAAAATCGAAGAAGGTAAACTGGTAATC
MBPTEV-R	GCACTGGAAATACAGGTTTTCACCATGGTGATGGTGATGGTGAGAAGATTTC
MBPTAA-F	CAGACTAATGGGATCGAGTAAAACCTGTACTTCCAATCCAATATTGGAAGTGG
MBPTAA-R	ACTCGATCCCATTAGTCTGCGCGTC

G33C variant of His-TEV-EGFP

The EGFP-pBAD vector containing the 6×His-T7-TEV-EGFP gene construct was purchased from Addgene (Cat. no. 54762). The glycine residue (G33) in the TEV site of this construct was substituted with the cysteine residue by site-directed mutagenesis employing SPRINP (single primer reactions in parallel) protocol[9]. Briefly, two parallel PCR reactions (25 µL) were carried out with forward (EGFP-F) and reverse (EGFP-R) primers (Table S1) in separate tubes. Each of the reaction mixtures contained 1× HF Buffer, 0.2 mM dNTPs, EGFP-F or EGFP-R (50 pmol), template plasmid DNA (~500 ng) and 0.5 U Phusion™ High-Fidelity DNA Polymerase (Thermofisher scientific, Cat. no. F530S). PCR reactions were performed according to the following protocol: 1 min at 98 °C, 30 cycles of (1 min at 98 °C, 1 min at 55 °C, 6 min at 72 °C), and a final 5 min extension at 72 °C. Next, the two PCR products were combined, heated to 95 °C and then slowly cooled to 37 °C over 10 min. Subsequently, the resultant mixture was subjected to *Dpn*I digestion (24 units, NEB, Cat no. R0176S, 20,000 units/mL) followed by transformation into *Escherichia coli* DH5α cells. The plasmid was isolated using QIAGEN miniprep kit (Cat. no. 27104), and the G33C mutation in the construct was confirmed by sequencing (Supplementary Fig. 17a).

G19C variant of His-TEV-mCherry

The pH6-TEV-mCherry vector containing a 6×His-TEV-mCherry gene construct was purchased from Addgene (Cat. no. 132430). The glycine residue (G19) in the TEV site of this construct was substituted with cysteine residue by site-directed mutagenesis using forward (mCherry-F) and reverse (mCherry-R) primers (Table S1) by performing a 25 µL-PCR reaction containing 1× HF Buffer, 0.2 mM dNTPs, mCherry-F and mCherry-R (1 µM each), template plasmid DNA (~150 ng), and 0.5 U Phusion™ High-Fidelity DNA Polymerase. PCR reactions were performed according to the following protocol: 1 min at 98 °C, 25 cycles of (1 min at 98 °C, 1 min at 55 °C, 4 min at 72 °C), and a final 5 min extension at 72 °C. The resultant PCR mixture was subjected to *Dpn*I digestion (24 units, NEB, Cat no. R0176S, 20,000 units/mL), followed by the transformation into *E. coli* DH5α cells. The plasmid was isolated using QIAGEN miniprep kit (Cat. no. 27104), and the G19C mutation in the construct was confirmed by sequencing (Supplementary Fig. 17b).

His-TEV-MBP construct

The pET His6 MBP TEV LIC cloning vector (2M-T) containing a 6×His-MBP-TEV gene construct was purchased from Addgene (Cat. no. 29708). The TEV sequence (ENLYFQC) was inserted after His-tag by site-directed mutagenesis using forward (MBPTEV-F) and reverse (MBPTEV-R) primers (Supplementary Table 1) by employing the protocol described above for the mCherry construct. TEV recognition sequence insertion in the construct (6×His-TEV-MBP-TEV) was confirmed by sequencing (Supplementary Fig. 17cc, upper panel). The resulting construct was used as a template in the next PCR reaction to introduce a stop codon (TAA) before the C-terminus TEV sequence using forward (MBPTAA-F) and reverse (MBPTAA-R) primers (Supplementary Table 1) following protocol as described above for mCherry. The final construct (His-TEV-MBP) was confirmed by sequencing data (Supplementary Fig. 17c, lower panel).

Supplementary Fig. 17. Sequence analysis of plasmid constructs. (**a**) Glycine to cysteine substitution (G33C) in His-TEV-EGFP construct. (**b**) Glycine to cysteine substitution (G19C) in His-TEV-mCherry construct. (**c**) Two-step generation of His-TEV-MBP construct via insertion of TEV-site (top panel, step 1) and stop codon (bottom panel, step 2).

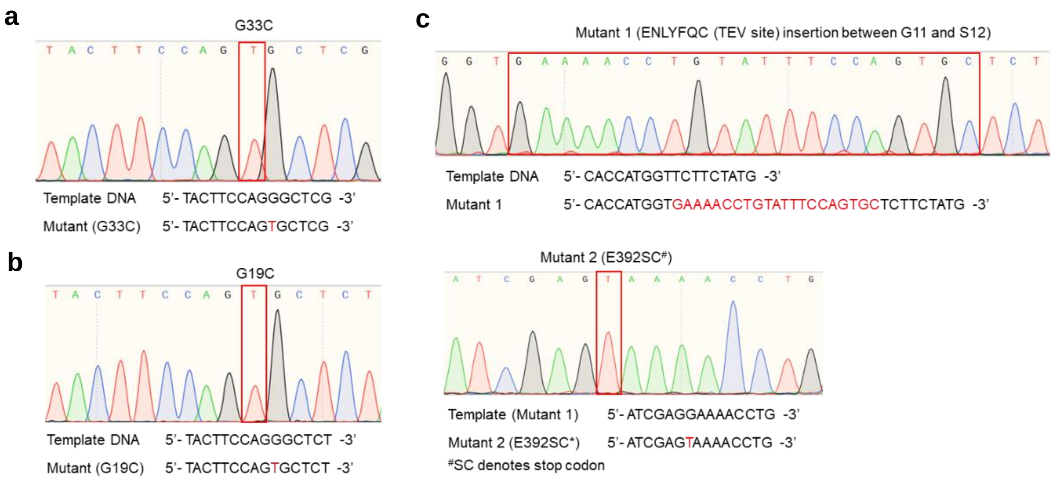

<u>Protein expression:</u>

The plasmids encoding the protein of interest were transformed into the respective competent *E. coli* cells (Supplementary Table 2). A single colony of transformed cells was inoculated into Luria Bertani (LB) media supplemented with the required antibiotic (Supplementary Table 2). The cells were cultured at 37 ºC with continuous shaking at 220 rpm. After 12 h, the cell culture was diluted with LB media (1:100, 500 mL) and the cells were grown further under the same conditions to an optical density (OD_{600}) of 0.5–0.6. Next, the protein expression was induced by adding the required concentration of the appropriate inducer (Supplementary Table 2). The cells were cultured at 25 °C, 220 rpm for 8–10 h and harvested by centrifugation (5000 g for 20 min) at 4 °C. The cell pellets were stored at -80 ºC until further purification.

Supplementary Table 2. Summary of protein expression conditions.

Protein	Plasmid used	Antibiotics (µg/mL)	*E. coli* expression strains	Inducer (conc.)
**His-TEV(G)-eGFP**	EGFP-pBAD	carbenicillin (100)	Top10	L-Arabinose (0.02%)
**His-TEV(C)-eGFP**	G33C construct of His-TEV-EGFP	carbenicillin (100)	Top10	L-Arabinose (0.02%)
**His-TEV(C)-MBP**	His-TEV-MBP construct	ampicillin (100) and chloramphenicol (35)	BL21(DE3) pLysS	IPTG (1 mM)
**His-TEV(C)-mCherry**	G19C construct of His-TEV-mCherry	kanamycin (50)	BL21(DE3)	IPTG (0.5 mM)
**His-TEV Protease**	MBP-TEVcs (ENLYFQ/G)-His6-TEVΔ (220-242)-R5	ampicillin (100)	BL21(DE3)	IPTG (1 mM)

Purification and storage of His-tagged proteins:

Cell pellet from a 500 mL culture (stored at -80 ºC), was thawed and resuspended in lysis buffer (20 mL) at pH 7.5 containing 50 mM Tris-HCl, 200 mM NaCl, 10 mM imidazole, 1 mM PMSF and 1 mM DTT. The cells were lysed by sonication for 3 min at 4 °C (20 s on, 59 s off, 40% amplitude). The cell debris were removed by centrifugation (20,000 g for 1 h) at 4 °C. The supernatant was separated and mixed with Ni-NTA agarose resin at 4 °C for 1 h to facilitate the binding of His-tagged protein. The resin was then loaded on a poly-prep chromatography column (Cat. no. 731-1550) and washed with 10 column volumes of wash buffer at pH 7.5 containing 50 mM Tris-HCl, 200 mM NaCl, 20 mM imidazole and 1 mM DTT. His-tagged proteins were eluted with buffer at pH 7.5 containing imidazole (30 → 250 mM), 50 mM Tris-HCl, 200 mM NaCl and 1 mM DTT, and the collected fractions were analyzed by SDS-PAGE for purity. The pure protein fractions were pooled and dialyzed (membrane cut off 12–14 kDa) overnight at 4 °C against storage buffer at pH 7.5 containing 50 mM Tris-HCl, 200 mM NaCl and 1 mM DTT. The concentration of the dialyzed protein was measured at A280 using nanodrop. The His-tagged proteins were either stored at -80 ºC after snap-freezing at a concentration of 2 mg/mL until cleavage with TEV protease was performed, or immediately subjected to TEV cleavage. The sequence of recombinantly expressed His-tagged proteins produced are provided in Supplementary Table 3, and the SDS-PAGE analysis of purified His-tagged proteins is depicted in Supplementary Fig. 18.

Purification of His-TEV protease:

Similar procedure was followed for the purification of His-TEV protease as described above using the following buffers: Lysis buffer at pH 8 containing 50 mM Tris-HCl, 200 mM NaCl, 25 mM imidazole, 1 mM PMSF and 10% Glycerol; wash buffer at pH 8 containing 50 mM Tris-HCl, 200 mM NaCl, 25 mM imidazole, and 10% glycerol; elution buffer at pH 8 containing (50 → 250 mM) imidazole, 50 mM Tris-HCl, 200 mM NaCl and 10% glycerol and dialysis/storage buffer at pH 8 containing 50 mM Tris-HCl, 200 mM NaCl and 10% glycerol. The protein was stored at a concentration of no greater than 1 mg/mL. SDS-PAGE analysis of the purified protease is depicted in Supplementary Fig. 18 (left panel).

TEV cleavage reaction to produce *N*-Cys POI (*N*-Cys-eGFP, *N*-Cys-MBP and *N*-Cys-mCherry) and *N*-Gly-eGFP.

The TEV protease cleavage was performed under the dialysis conditions. In a 3000 MWCO dialysis bag, His-tagged protein containing the TEV recognition site (2 mg/mL) was treated with TEV protease (protein/TEV protease molar ratio 25:1) in buffer at pH 8 containing 50 mM Tris-HCl, 200 mM NaCl, 0.5 mM EDTA and 1 mM DTT. The dialysis bag was gently stirred in 300 mL dialysate (50 mM Tris-HCl pH 8, 200 mM NaCl, 0.5 mM EDTA and 1 mM DTT) for 16 h at 4 °C. The dialyzed protein solution was mixed with Ni-NTA resin and loaded on a poly prep column to elute TEV-cleaved protein in the flowthrough. The purity of collected protein fractions was checked by SDS-PAGE analysis. Aliquots of pure protein fractions (~1 mg/mL) were snap-frozen in liquid N_2 and stored at -80 ^{o}C until their use for protein bioconjugation. The sequence of the TEV protease-cleaved proteins is provided in Supplementary Table 4. The SDS-PAGE analysis of the proteins before and after cleavage are depicted in Supplementary Fig. 18 (2nd to 5th panels from left). All the cleaved proteins were characterized by ESI-MS (Supplementary Fig. 19 for *N*-Cys-POI and Supplementary Fig. 22 for *N*-Gly-eGFP). The theoretical and the observed protein masses are provided in Supplementary Table 5.

Supplementary Table 3. Sequence of the expressed recombinant His-tagged proteins employed for TEV cleavage reactions. The TEV site is underlined and the vertical line in the site denotes the TEV protease cleavage site.

<table>
<tr><th>Protein</th><th>Sequence</th></tr>
<tr><td>His-TEV(G)-eGFP</td><td>MRGSHHHHHHGMASMTGGQQMGRDLYENLYFQ|GSSMVSKGEELFTGVVPILVELDGDVNGHKFSVS
GEGEGDATYGKLTLKFICTTGKLPVPWPTLVTTLTYGVQCFSRYPDHMKQHDFFKSAMPEGYVQERTIFFK
DDGNYKTRAEVKFEGDTLVNRIELKGIDFKEDGNILGHKLEYNYNSHNVYIMADKQKNGIKVNFKIRHNIED
GSVQLADHYQQNTPIGDGPVLLPDNHYLSTQSALSKDPNEKRDHMVLLEFVTAAGITLGMDELYK</td></tr>
<tr><td>His-TEV(C)-eGFP</td><td>MRGSHHHHHHGMASMTGGQQMGRDLYENLYFQ|CSSMVSKGEELFTGVVPILVELDGDVNGHKFSVS
GEGEGDATYGKLTLKFICTTGKLPVPWPTLVTTLTYGVQCFSRYPDHMKQHDFFKSAMPEGYVQERTIFFK
DDGNYKTRAEVKFEGDTLVNRIELKGIDFKEDGNILGHKLEYNYNSHNVYIMADKQKNGIKVNFKIRHNIED
GSVQLADHYQQNTPIGDGPVLLPDNHYLSTQSALSKDPNEKRDHMVLLEFVTAAGITLGMDELYK</td></tr>
<tr><td>His-TEV(C)-MBP</td><td>MKSSHHHHHHGENLYFQ|CSSMKIEEGKLVIWINGDKGYNGLAEVGKKFEKDTGIKVTVEHPDKLEEKFPQ
VAATGDGPDIIFWAHDRFGGYAQSGLLAEITPDKAFQDKLYPFTWDAVRYNGKLIAYPIAVEALSLIYNKDLL
PNPPKTWEEIPALDKELKAKGKSALMFNLQEPYFTWPLIAADGGYAFKYENGKYDIKDVGVDNAGAKAGLT
FLVDLIKNKHMNADTDYSIAEAAFNKGETAMTINGPWAWSNIDTSKVNYGVTVLPTFKGQPSKPFVGVLS
AGINAASPNKELAKEFLENYLLTDEGLEAVNKDKPLGAVALKSYEEELAKDPRIAATMENAQKGEIMPNIPQ
MSAFWYAVRTAVINAASGRQTVDEALKDAQTNGIE</td></tr>
<tr><td>His-TEV(C)-mCherry</td><td>MGSDKIHHHHHHENLYFQ|CSMVSKGEEDNMAIIKEFMRFKVHMEGSVNGHEFEIEGEGEGRPYEGTQT
AKLKVTKGGPLPFAWDILSPQFMYGSKAYVKHPADIPDYLKLSFPEGFKWERVMNFEDGGVVTVTQDSSL
QDGEFIYKVKLRGTNFPSDGPVMQKKTMGWEASSERMYPEDGALKGEIKQRLKLKDGGHYDAEVKTTYK
AKKPVQLPGAYNVNIKLDITSHNEDYTIVEQYERAEGRHSTGGMDELYK</td></tr>
</table>

Supplementary Table 4. Sequence of TEV cleaved proteins employed for *N*-(Cys) bioconjugation. The *N*-Gly-eGFP was employed in the control reactions.

<table>
<tr><th>Protein</th><th>Sequence</th></tr>
<tr><td>N-Gly-eGFP</td><td>GSSMVSKGEELFTGVVPILVELDGDVNGHKFSVSGEGEGDATYGKLTLKFICTTGKLPVPWPTLVTTLTYGV
QCFSRYPDHMKQHDFFKSAMPEGYVQERTIFFKDDGNYKTRAEVKFEGDTLVNRIELKGIDFKEDGNILGH
KLEYNYNSHNVYIMADKQKNGIKVNFKIRHNIEDGSVQLADHYQQNTPIGDGPVLLPDNHYLSTQSALSKD
PNEKRDHMVLLEFVTAAGITLGMDELYK</td></tr>
<tr><td>N-Cys-eGFP</td><td>CSSMVSKGEELFTGVVPILVELDGDVNGHKFSVSGEGEGDATYGKLTLKFICTTGKLPVPWPTLVTTLTYGVQ
CFSRYPDHMKQHDFFKSAMPEGYVQERTIFFKDDGNYKTRAEVKFEGDTLVNRIELKGIDFKEDGNILGHKL</td></tr>
</table>

	EYNYNSHNVYIMADKQKNGIKVNFKIRHNIEDGSVQLADHYQQNTPIGDGPVLLPDNHYLSTQSALSKDPN EKRDHMVLLEFVTAAGITLGMDELYK
***N*-Cys-MBP**	CSSMKIEEGKLVIWINGDKGYNGLAEVGKKFEKDTGIKVTVEHPDKLEEKFPQVAATGDGPDIIFWAHDRFG GYAQSGLLAEITPDKAFQDKLYPFTWDAVRYNGKLIAYPIAVEALSLIYNKDLLPNPPKTWEEIPALDKELKAK GKSALMFNLQEPYFTWPLIAADGGYAFKYENGKYDIKDVGVDNAGAKAGLTFLVDLIKNKHMNADTDYSIA EAAFNKGETAMTINGPWAWSNIDTSKVNYGVTVLPTFKGQPSKPFVGVLSAGINAASPNKELAKEFLENYL LTDEGLEAVNKDKPLGAVALKSYEEELAKDPRIAATMENAQKGEIMPNIPQMSAFWYAVRTAVINAASGR QTVDEALKDAQTNGIE
***N*-Cys-mCherry**	CSMVSKGEEDNMAIIKEFMRFKVHMEGSVNGHEFEIEGEGEGRPYEGTQTAKLKVTKGGPLPFAWDILSP QFMYGSKAYVKHPADIPDYLKLSFPEGFKWERVMNFEDGGVVTVTQDSSLQDGEFIYKVKLRGTNFPSDGP VMQKKTMGWEASSERMYPEDGALKGEIKQRLKLKDGGHYDAEVKTTYKAKKPVQLPGAYNVNIKLDITSH NEDYTIVEQYERAEGRHSTGGMDELYK

Supplementary Fig. 18. SDS-PAGE analysis of purified His-tagged and TEV-cleaved proteins. The numbers on the gel images denote the protein marker size in kDa.

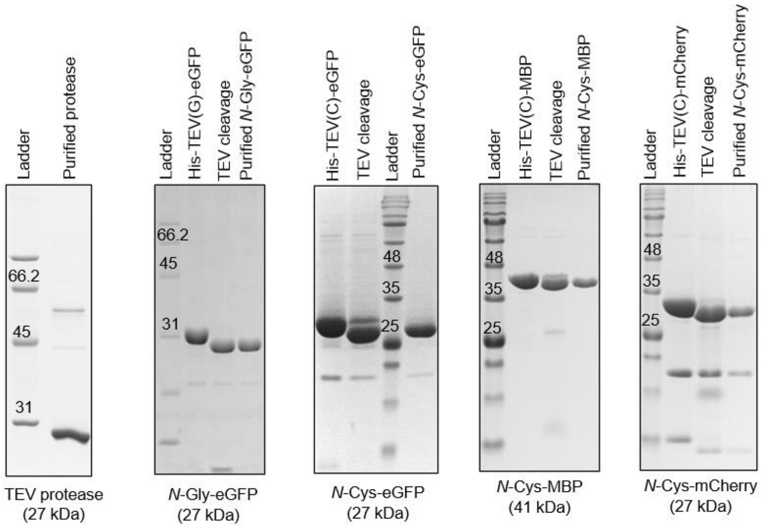

7. Labelling of *N*-Cys-POI via BHoPAL and the ESI-MS analysis of resultant protein conjugates

An aliquot (0.5 mL) of *N*-Cys-POI stock (prepared as described above) was concentrated and desalted to a final volume of ~50 µL by using an Amicon ultra-0.5 10K filter. The resultant desalted protein solution was diluted by adding sodium phosphate buffer at pH 7 (0.45 mL, 20 mM) and treated with TCEP (10 eq., 50 mM stock in water) at RT. After 1 h of incubation, the excess TCEP was removed by using an Amicon ultra-0.5 10K filter. The resultant concentrated

protein sample was diluted to the desired concentration by adding water. In general, bioconjugation reactions were set up by mixing *N*-Cys-POI (5 µL, 0.5 mM stock in water, final conc. 10 µM, 1 eq.), sodium phosphate buffer at pH 6.5 (220 µL, 50 mM), and IBH adducts **1A**/**1D**/**1E** (25 µL, 0.1 mM stock in CH_3CN, final conc. 10 µM, 1 eq.) or IBH adducts **1F**/**1G** (25 µL, 0.3 mM stock in CH_3CN, final conc. 30 µM, 3 eq.) at RT. After 1 h (for **1A**/**1D**/**1E**) or 3 h (for **1F**/**1G**), the reaction mixture was desalted using an Amicon ultra-0.5 10K filter followed by the ESI-MS characterization of the resultant desalted protein conjugates (Fig. 4 of the main text and Supplementary Fig. 19). The theoretical and the observed masses obtained for the desired cyclic *N*-Cys-IBH conjugates are provided in Supplementary Table 5. Different bioconjugation parameters (protein concentrations, reaction duration, reagent equivalence and reaction temperature) were screened as summarized below (conditions 1–6) and Table 1 of the main text.

Condition 1: *N*-Cys-POI (10 µM, 1 eq.), **1A**/**1D**/**1E** (1 eq.), 25 °C, 1 h in sodium phosphate buffer (50 mM at pH 6.5). The deconvoluted MS spectra depicting the region of unmodified and modified protein conjugates (24–32 kDa) are provided in Fig. 4, and the XIC and the entire deconvoluted spectra are provided in Supplementary Fig. 19.

Condition 2: *N*-Cys-POI (10 µM, 1 eq.), **1D**/**1E** (3 eq.), 25 °C, 10–20 min in sodium phosphate buffer (50 mM at pH 6.5). The deconvoluted MS spectra are provided given in Supplementary Fig. 20.

Condition 3: *N*-Cys-POI (10 µM, 1 eq.), **1F**/**1G** (1–3 eq.), 25 °C, 1–6 h in sodium phosphate buffer (50 mM at pH 6.5). The reactions were performed either with 1 or 3 equivalents of *N*-alkyl IBH **1F/1G** at three different time points (1 h, 3 h and 6 h). The percentage conversions observed are summarized in Supplementary Table 6 and the deconvoluted spectra acquired at these time points are provided Supplementary Fig. 21. The deconvoluted MS spectra (38–46 kDa range (for MBP) and 24–32 kDa range (for eGFP and mCherry) depicting masses of protein conjugates obtained after 6 h are provided in Fig. 4 of the main text and the XIC and the entire deconvoluted spectra are provided in Supplementary Fig. 19.

Condition 4 (control reaction): *N*-Gly-POI (10 µM, 1 eq.), **1D**/**1E** (1 eq.), 25 °C, 1 h in sodium phosphate buffer (50 mM at pH 6.5). No modification was observed in both of these reactions. The XIC and the deconvoluted MS spectra are provided in Supplementary Fig. 22.

Condition 5: *N*-Cys-POI (100 nM–5.0 µM, 1.0 eq.), **1D**/**1E** (1–6 eq.), 25 °C, 1 h in sodium phosphate buffer (50 mM at pH 6.5). The deconvoluted MS spectra of these reactions are provided in Supplementary Fig. 24.

Condition 6: *N*-Cys-POI (10 µM, 1.0 eq.), **1A**/**1D**/**1E** (1 eq.), 1 h in sodium phosphate buffer (50 mM at pH 6.5). The reactions were performed at lower temperatures (10 and 4 °C). The deconvoluted MS spectra of these reactions are provided in Supplementary Fig. 25.

Supplementary Table 5. Theoretical and observed molecular masses of the unmodified *N*-Cys-POI and their desired cyclic *N*-Cys-IBH adduct conjugates. POI denotes protein of interest.

S.No.	Protein	Theoretical mass (Da)	Observed mass (Da)
**1**	*N*-Gly-eGFP	27152.7	27150.9
**2**	*N*-Cys-eGFP	27198.8	27197.0
**3**	*N*-Cys-MBP	41030.6	41031.9
**4**	*N*-Cys-mCherry	26892.3	26890.0
**5**	*N*-Cys-eGFP-**1A**	27394.0	27394.0
**6**	*N*-Cys-eGFP-**1D**	27446.1	27446.5
**7**	*N*-Cys-eGFP-**1E**	27476.1	27476.4
**8**	*N*-Cys-eGFP-**1F**	27390.1	27390.4
**9**	*N*-Cys-eGFP-**1G**	27709.2	27710.1
**10**	*N*-Cys-MBP-**1A**	41228.9	41228.6
**11**	*N*-Cys-MBP-**1D**	41280.9	41280.8
**12**	*N*-Cys-MBP-**1E**	41311.0	41311.0
**13**	*N*-Cys-MBP-**1F**	41225.0	41225.7
**14**	*N*-Cys-MBP-**1G**	41544.1	41545.9
**15**	*N*-Cys-mCherry-**1A**	27087.0	27087.2
**16**	*N*-Cys-mCherry-**1D**	27139.1	27138.4
**17**	*N*-Cys-mCherry-**1E**	27169.1	27168.7
**18**	*N*-Cys-mCherry-**1F**	27083.1	27083.9
**19**	*N*-Cys-mCherry-**1G**	27402.2	27403.7

Supplementary Table 6. Labelling of *N*-Cys proteins with *N*-alkyl IBH adducts. (**a**) **1F** and (**b**) **1G**. The reactions were conducted by incubating 10 μM *N*-Cys POI (1 eq.) with IBH adducts (1–3 eq.) in sodium phosphate buffer (50 mM, pH 6.5) at 25 °C followed by ESI-MS analysis at different time points. **A (%)**, **B (%)** and **C (%)** represent percentage of unmodified *N*-Cys POI, uncyclized IBH-POI conjugate and cyclized IBH-POI conjugate (bis-heterocycle), respectively. All the deconvoluted ESI-MS spectra are summarized in Supplementary Fig. 21. POI = Protein of interest.

a

IBH adduct 1F (eq.)	Time (h)	eGFP A (%) | B (%) | C (%)	MBP A (%) | B (%) | C (%)	mCherry A (%) | B (%) | C (%)
**1.0**	1	0 | 38 | 62	0 | 45 | 55	n.d.
	3	0 | 24 | 76	0 | 0 | 100	n.d.
	6	0 | 0 | 100	0 | 0 | 100	n.d.
**3.0**	1	0 | 32 | 68	0 | 40 | 60	0 | 33 | 67
	3	0 | 23 | 77	0 | 0 | 100	0 | 25 | 75
	6	0 | 0 | 100	0 | 0 | 100	0 | 0 | 100

n.d. = not determined

b

IBH adduct 1G (eq.)	Time (h)	eGFP A (%) | B (%) | C (%)	MBP A (%) | B (%) | C (%)	mCherry A (%) | B (%) | C (%)
**1.0**	1	46 | 28 | 26	10 | 56 | 34	n.d.
	3	32 | 19 | 49	0 | 28 | 72	n.d.
	6	34 | 12 | 54	0 | 0 | 100	n.d.
**3.0**	1	0 | 58 | 42	0 | 44 | 56	0 | 50 | 50
	3	0 | 27 | 73	0 | 0 | 100	0 | 29 | 71
	6	0 | 0 | 100	0 | 0 | 100	0 | 0 | 100

Supplementary Fig. 19. ESI-MS spectra of *N*-Cys-POI conjugates generated via BHoPAL. Extracted ion chromatogram (XIC) is shown at the top panel and its deconvoluted mass spectrum at the bottom panel for each of the protein conjugate. The observed masses of the bis-heterocyclic *N*-Cys-POI conjugates are shown in red and their $[M+2H]^{2+}$ masses in green. The corresponding theoretical masses are shown in grey within parenthesis. The minor peaks in the mass spectra belong to impurities present in the recombinantly produced proteins. The first three entries correspond to the ESI-MS analysis of unmodified *N*-Cys POI.

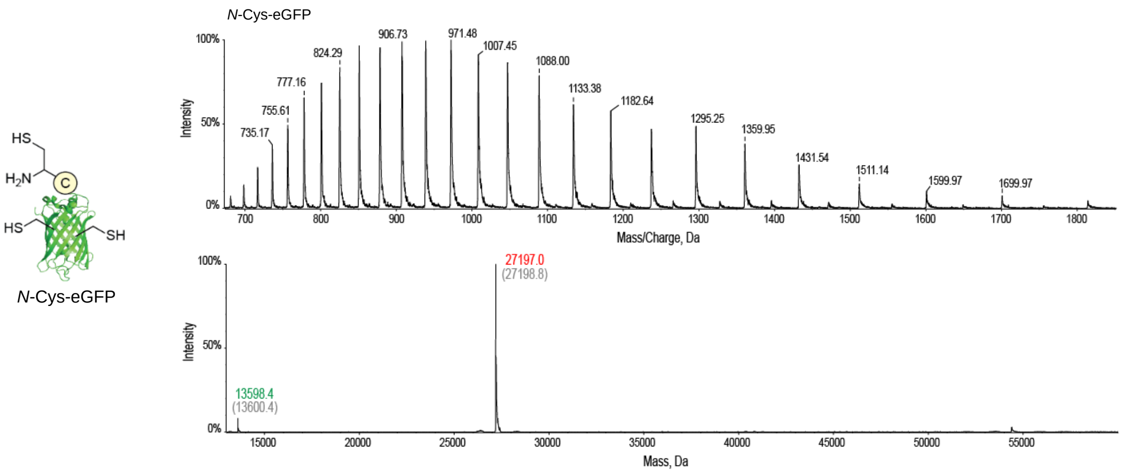

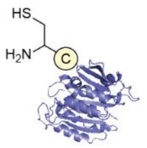

N-Cys-MBP

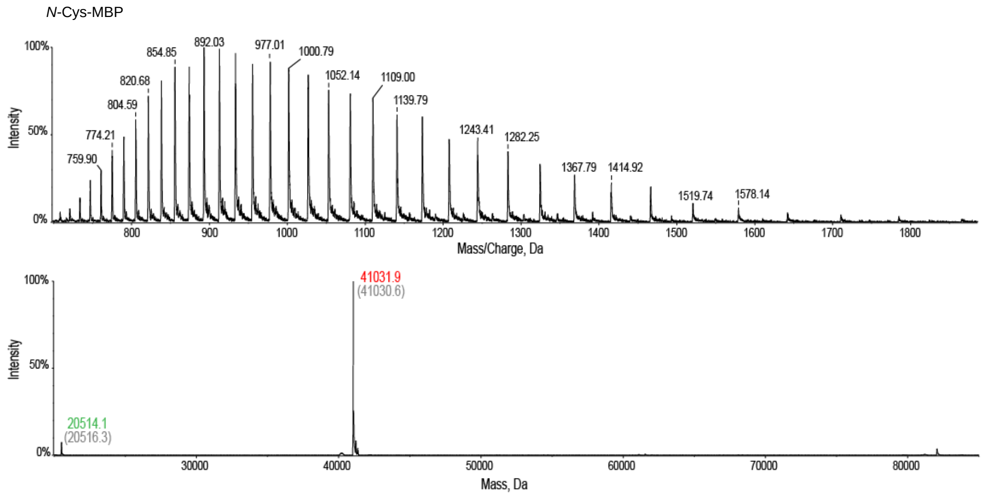

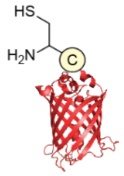

N-Cys-mCherry

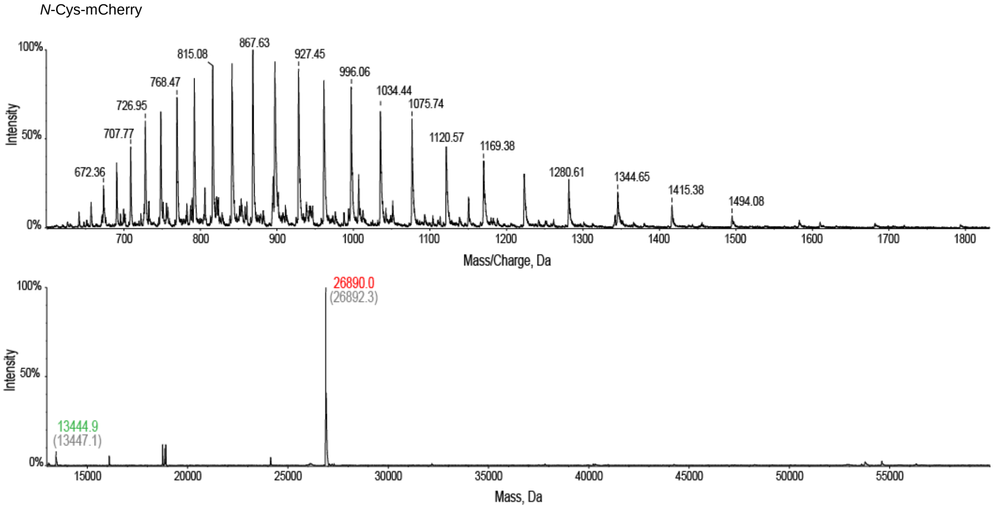

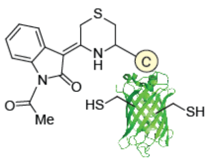

N-Cys-eGFP-**1A**

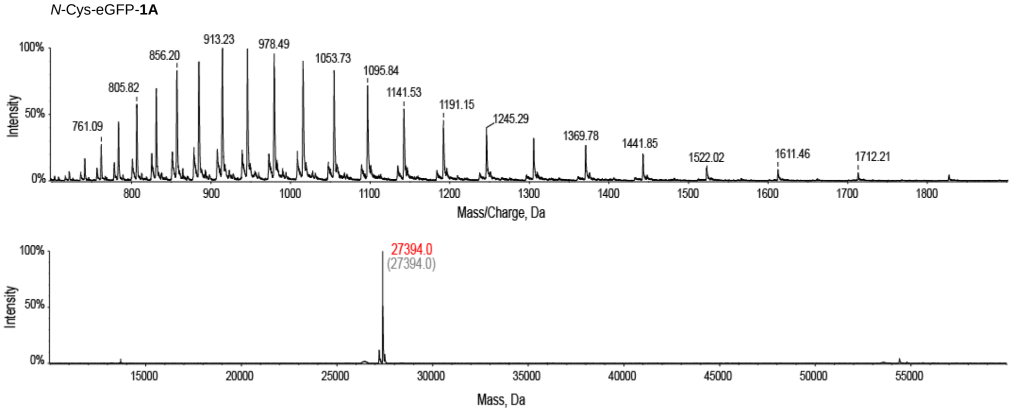

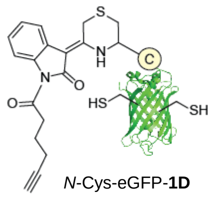

N-Cys-eGFP-**1D**

N-Cys-eGFP-**1D**

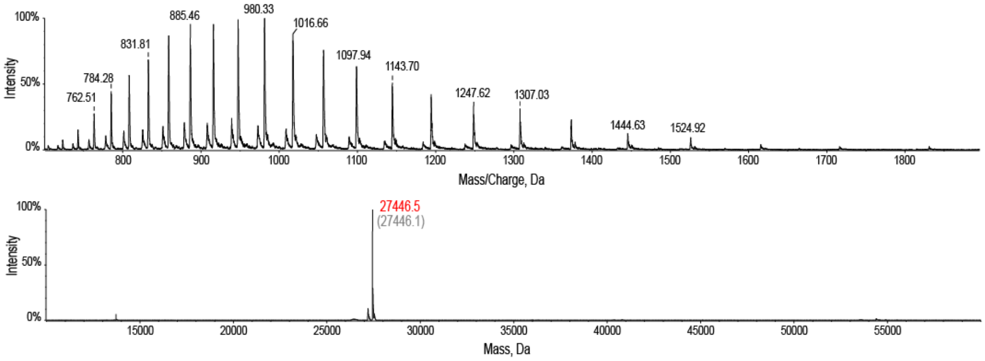

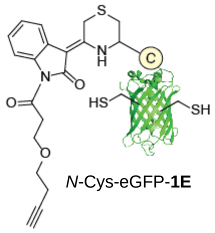

N-Cys-eGFP-**1E**

N-Cys-eGFP-**1E**

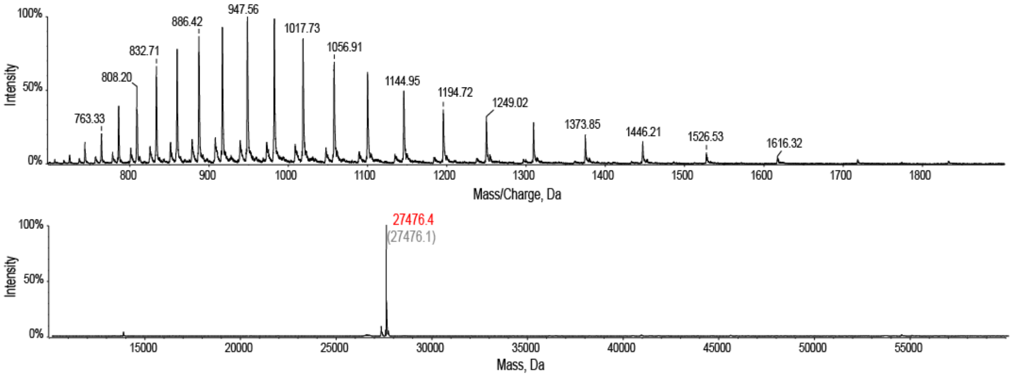

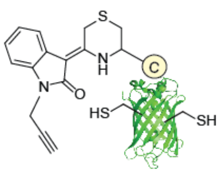

N-Cys-eGFP-**1F**

N-Cys-eGFP-**1F**

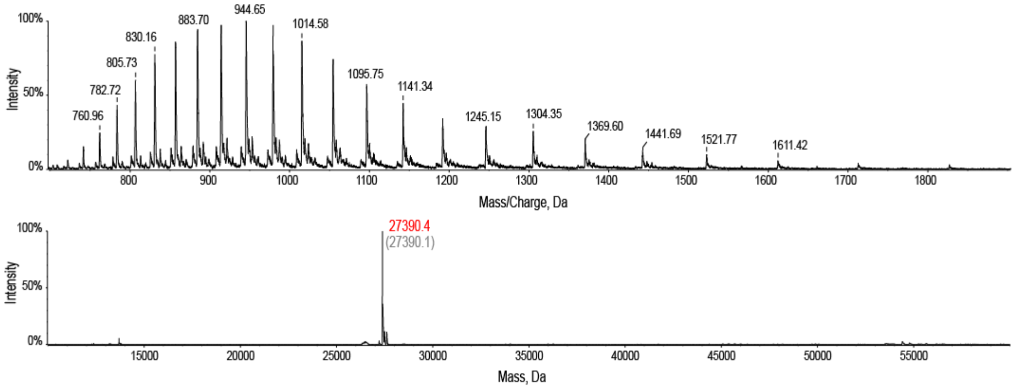

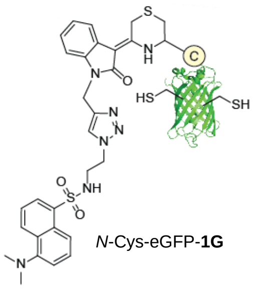

N-Cys-eGFP-**1G**

N-Cys-eGFP-**1G**

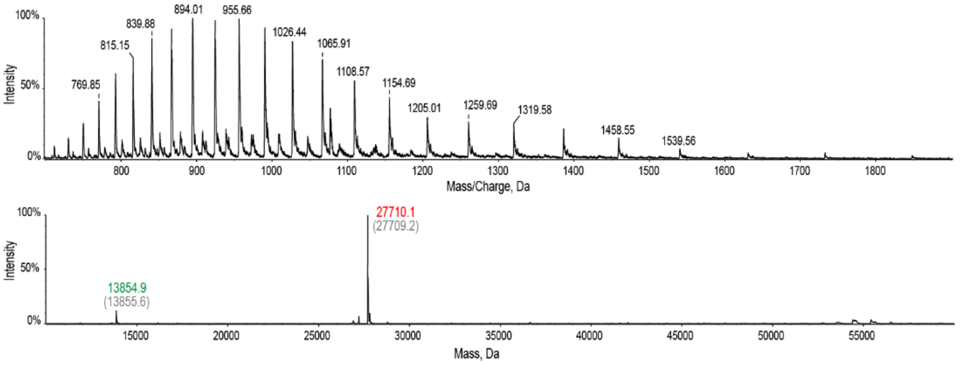

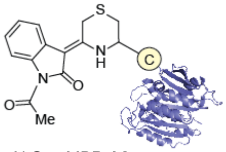

N-Cys-MBP-**1A**

N-Cys-MBP-**1A**

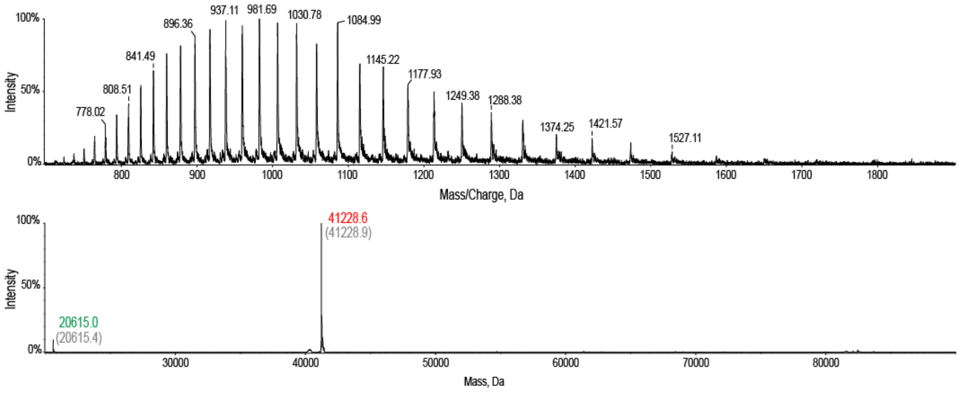

N-Cys-MBP-**1D**

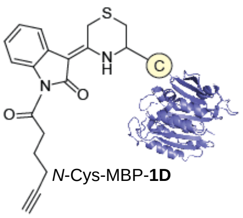

N-Cys-MBP-**1D**

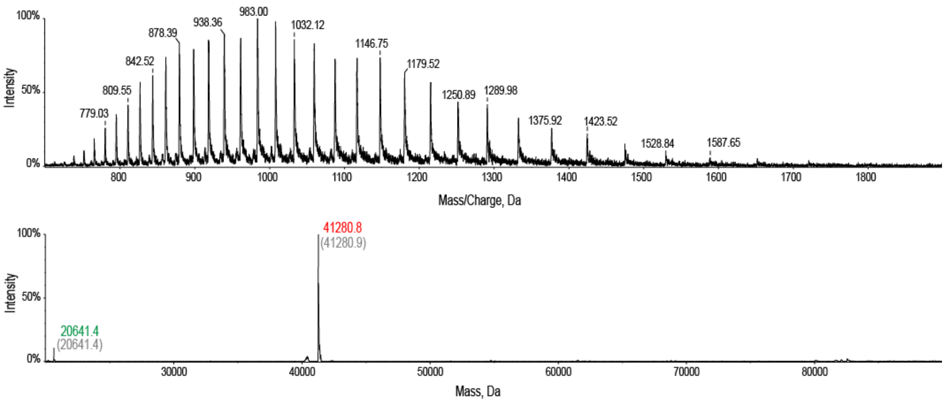

N-Cys-MBP-**1E**

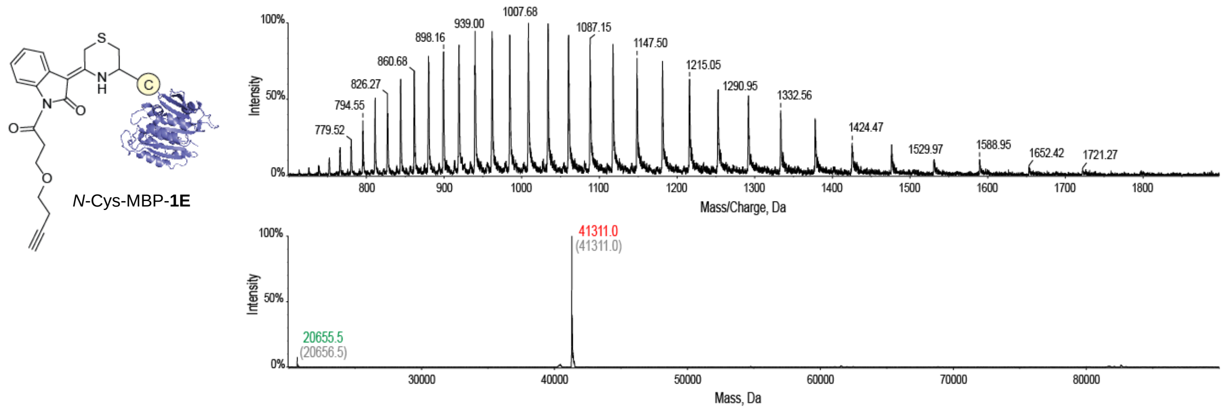

N-Cys-MBP-**1F**

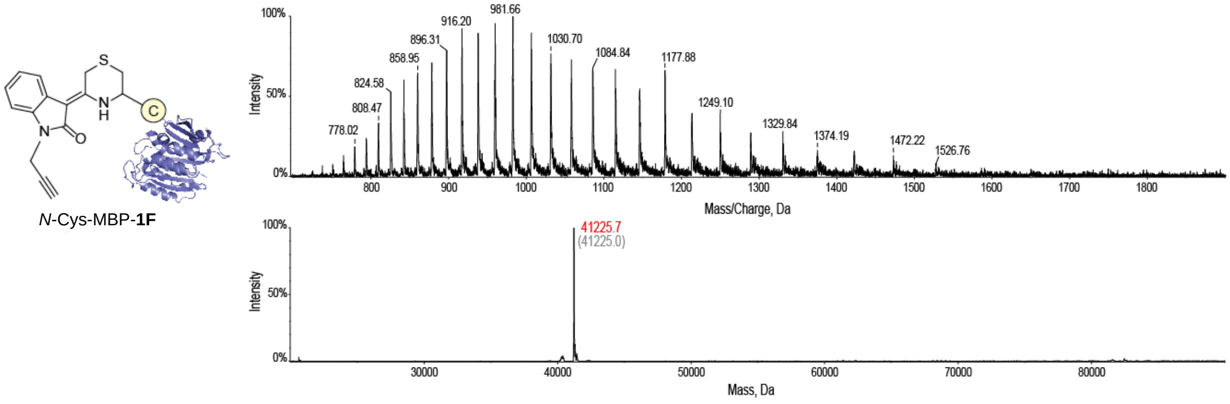

N-Cys-MBP-**1G**

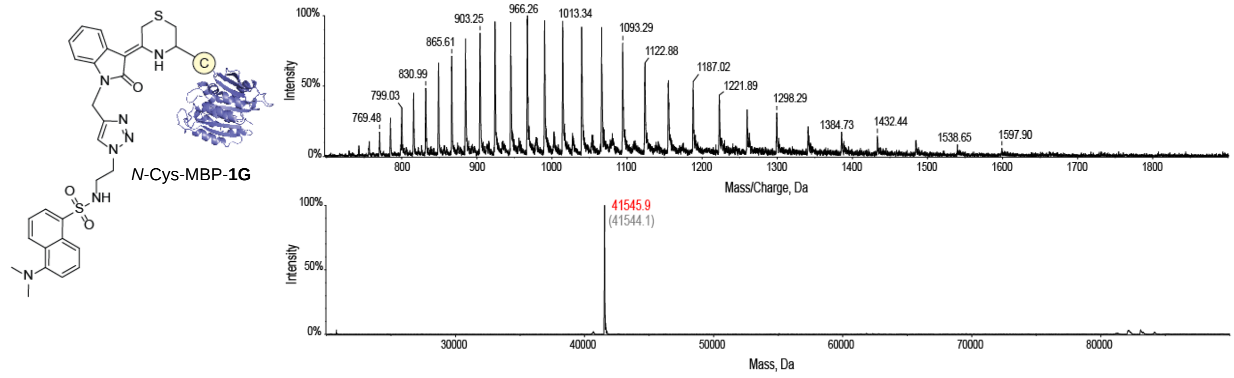

N-Cys-mCherry-**1A**

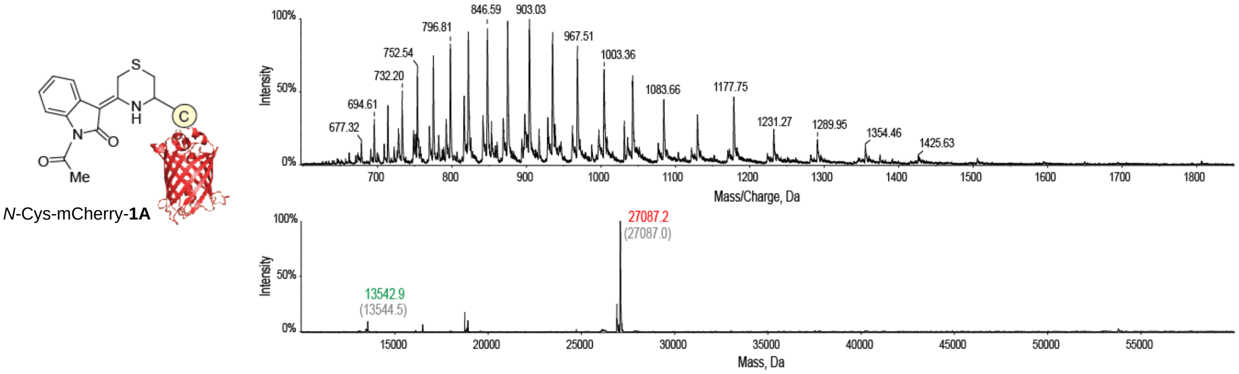

N-Cys-mCherry-**1D**

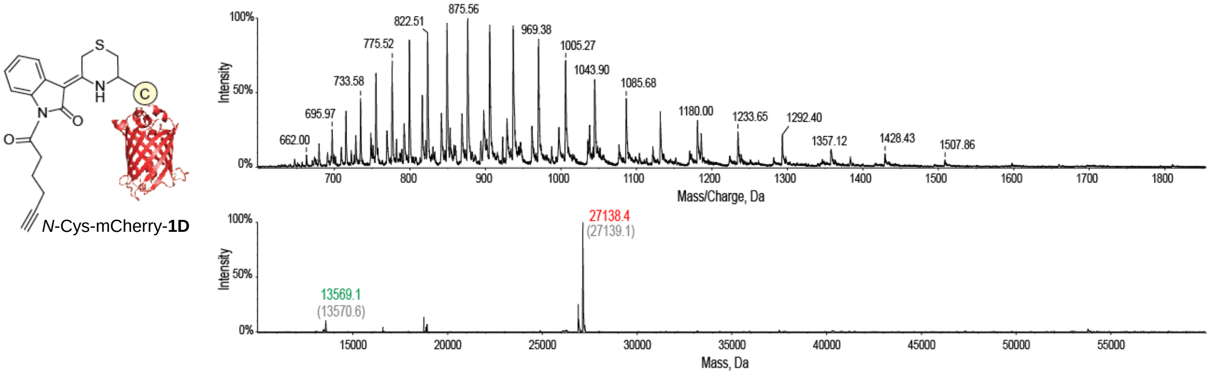

N-Cys-mCherry-**1E**

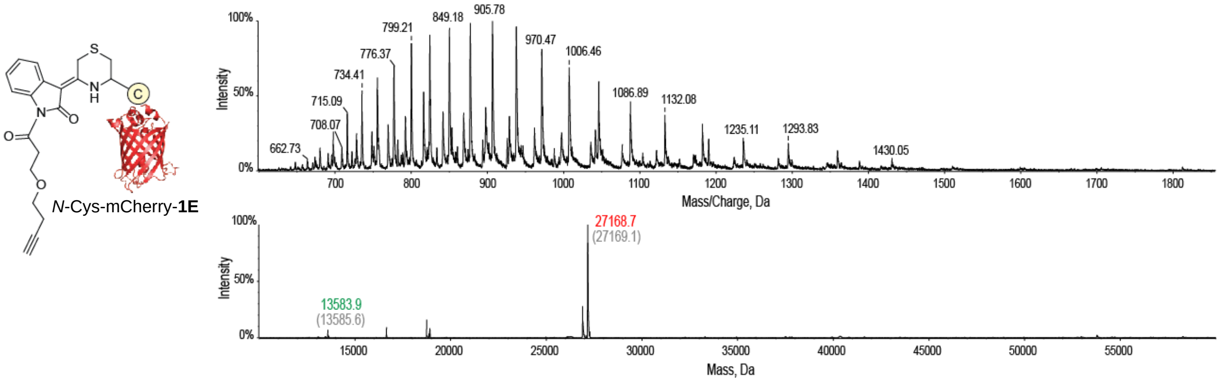

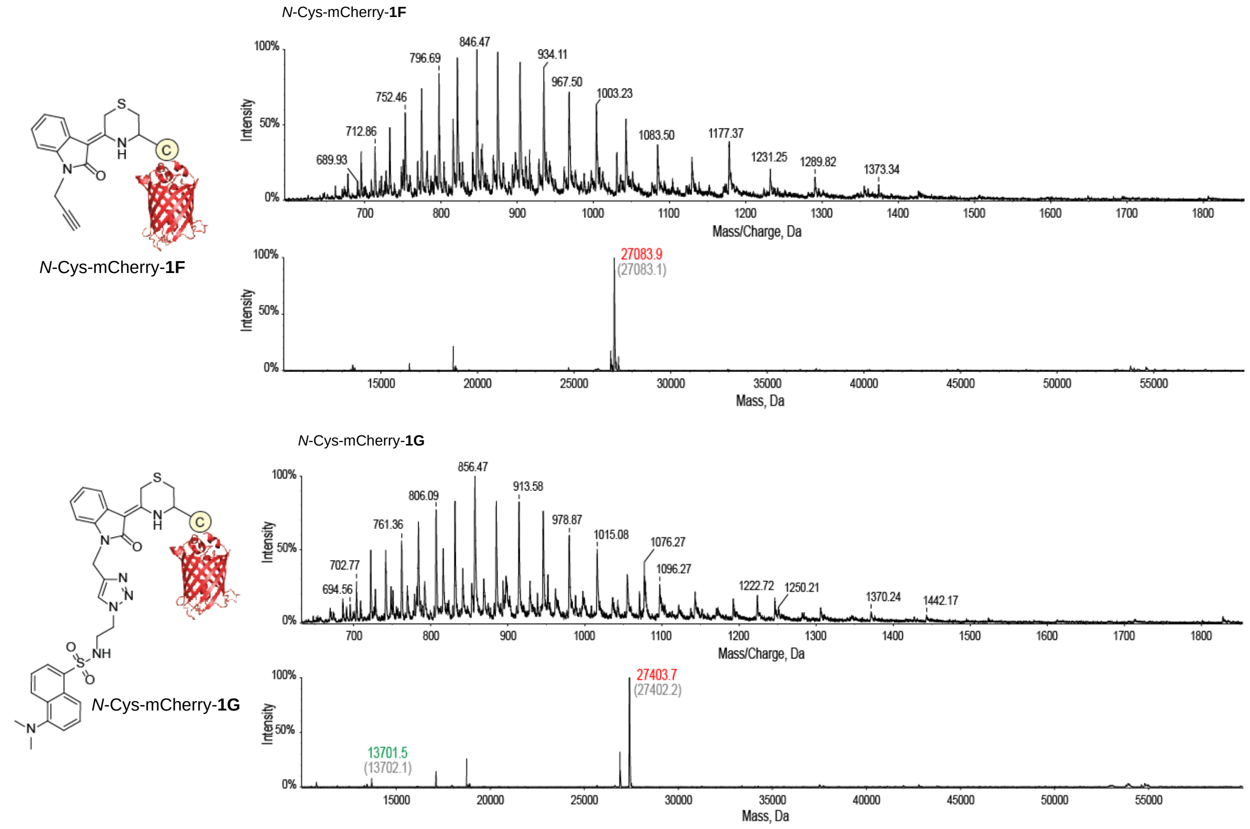

Supplementary Fig. 20. Labelling of *N*-Cys-eGFP with *N*-acyl IBH adduct at low time points. The deconvoluted mass spectra obtained upon treating *N*-Cys-eGFP (10 µM, 1 eq.) in sodium phosphate buffer (pH 6.5) at 25 °C for 10/20 min with 3 eq. of *N*-acyl IBH, (**a**) **1D** and (**b**) **1E**. The deconvoluted mass spectra obtained after 10 min of the reaction are shown at the top panel and that obtained after 20 min at the bottom panel. Quantitative conversions were obtained with both the reagents. The masses for the desired cyclic *N*-Cys-eGFP-**1D** and *N*-Cys-eGFP-**1E** conjugates are shown in red, and their respective $[M+2H]^{2+}$ masses in green. The corresponding theoretical masses are shown in grey within parenthesis.

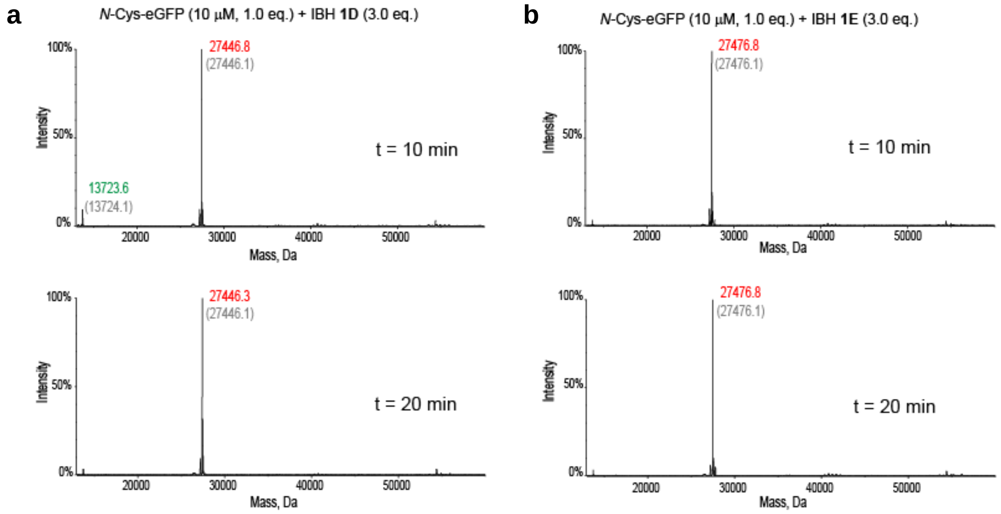

Supplementary Fig. 21. Optimization of bioconjugation conditions for labelling *N*-Cys-POI with *N*-alkyl IBH adducts. The deconvoluted MS spectra obtained under different conditions upon the treatment of IBH adducts, **1F**/**1G** with (**a**) *N*-Cys-eGFP. (**b**) *N*-Cys-MBP and (**c**) *N*-Cys-mCherry are summarized below. The reactions were conducted by incubating at 10 μM POI (1 eq.) with IBH adducts (1 or 3 eq.) in sodium phosphate buffer (50 mM, pH 6.5) at 25 °C followed by their ESI-MS analysis at different time points. A, B and C represent the masses correspond to the unmodified *N*-Cys-POI, uncyclized IBH-POI conjugate and cyclized IBH-POI conjugate (bis-heterocycle), respectively. The corresponding theoretical masses are shown in grey within parenthesis. Please refer to Supplementary Table 6 for the % conversions observed at different time points. POI = protein of interest.

a

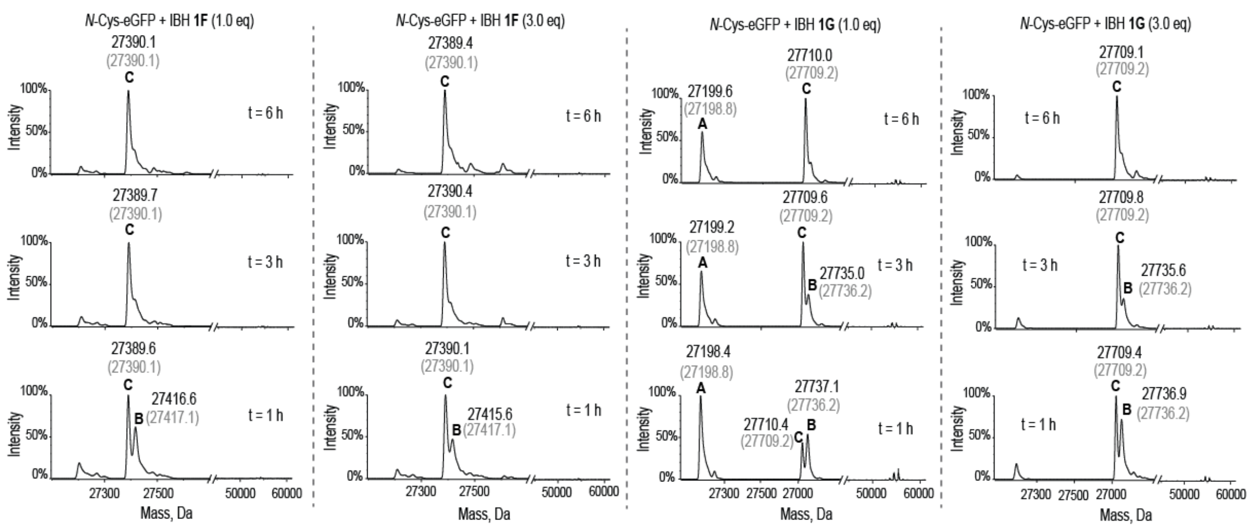

b

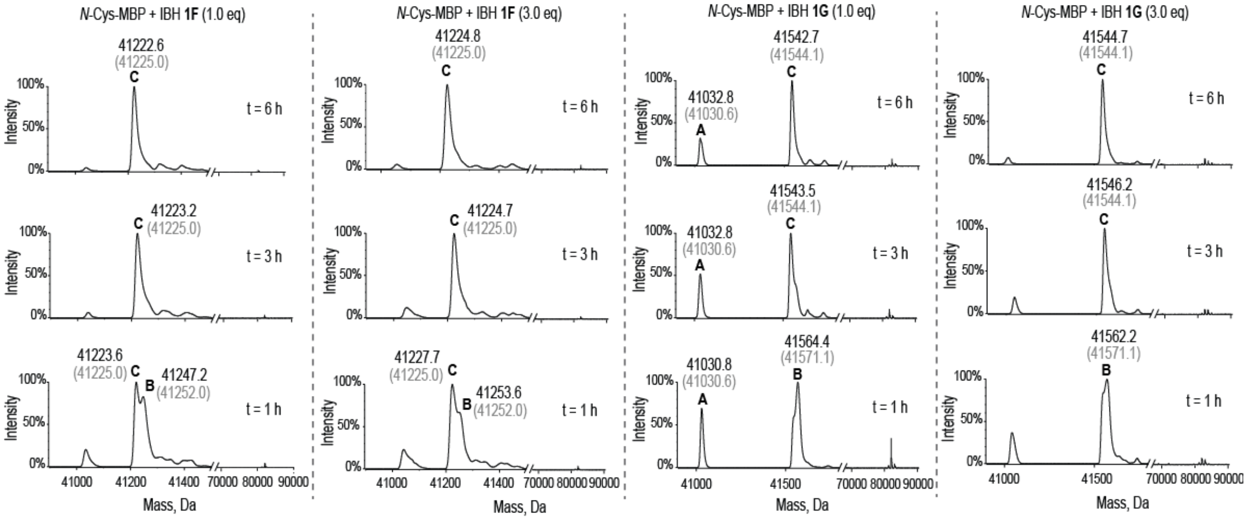

c

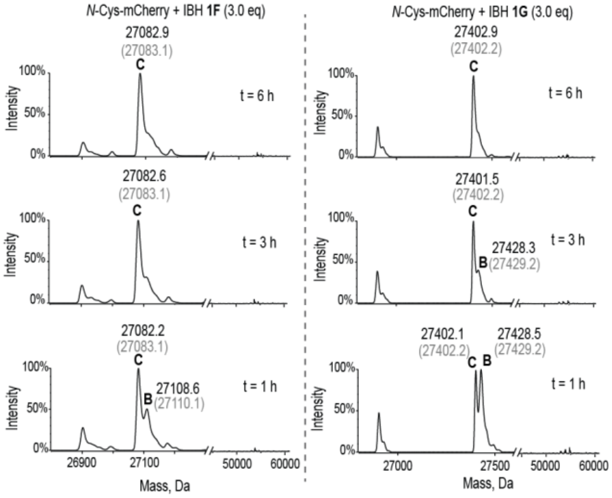

Supplementary Fig. 22. Selective labelling of *N*-Cys residue of proteins via BHoPAL. Control reaction involving treatment of *N*-Gly-eGFP (10 µM) with *N*-acyl IBH adducts **1D**/**1E** (1 eq.) in sodium phosphate buffer (pH 6.5) at 25 ºC for 1 h did not show any protein modification. XICs obtained in these reactions are shown on the left, and their deconvoluted mass spectra on the right. The observed mass is shown in black and the corresponding theoretical mass in grey within parenthesis. Top panel: no treatment, middle panel: treatment with **1D** and lower panel: treatment with **1E**.

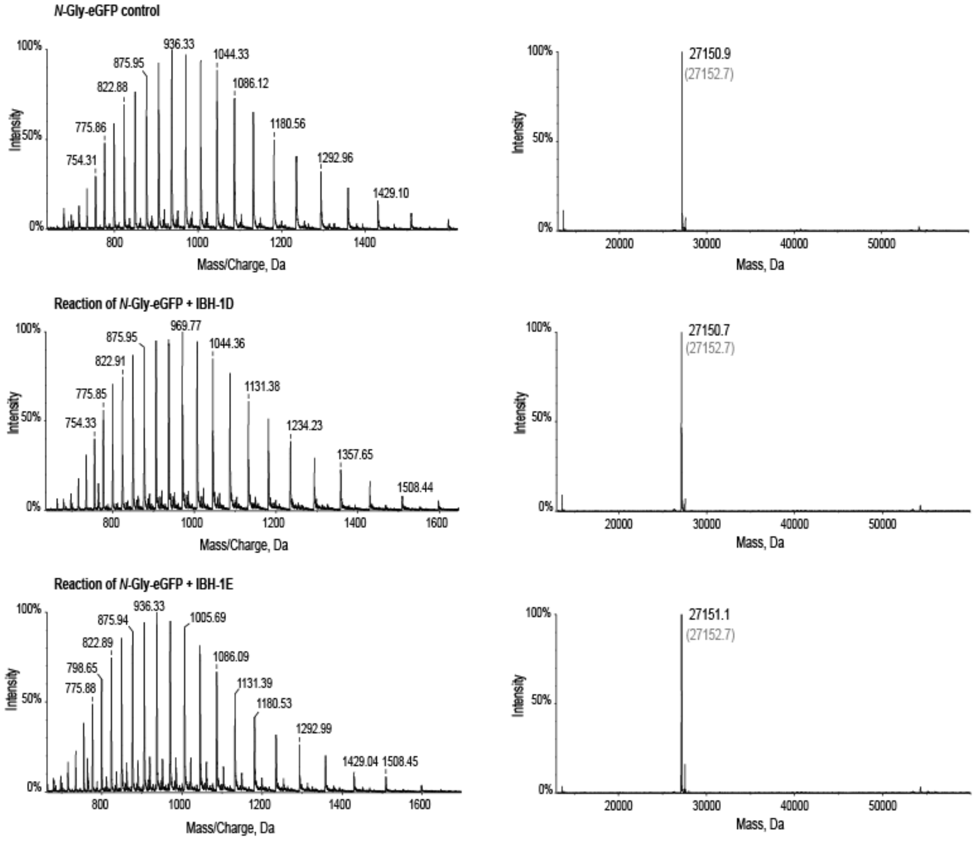

Procedure for the trypsinization of *N*-Cys labelled conjugates for the MS/MS analysis

In a 1.5 mL microfuge tube, a solution of *N*-Cys-eGFP-**1D** conjugate (15 µL, 100 µg) was treated sequentially with a solution of NH_4HCO_3 (15 µL, 0.2 M in milli-Q water), trifluoroethanol (30 µL) and DTT (3 µL, 0.2 M in milli-Q water). The resulting mixture was vortexed briefly and heated at 90 ^{o}C for 20 min. To this solution was then added a solution of iodoacetamide (12 µL, 0.2 M in milli-Q water). The mixture was vortexed briefly and incubated at 25 ^{o}C for 1 h in the dark. The excess of iodoacetamide was quenched by adding a solution of DTT (3.5 µL, 0.2 M in milli-Q water) to the mixture. After incubating at 25 ^{o}C for 1 h in the dark, the mixture was diluted with water (530 µL) and treated with a solution of NH_4HCO_3 (100 µL, 0.2 M in milli-Q water) to attain a pH value between ~7.5–8.0. To the resultant protein solution was added a solution of trypsin (5 µL, 1 µg/µL stock in 1 mM HCl, 5 µg) at 1:20 enzyme: substrate ratio. The mixture was incubated at 37 ^{o}C for 10–12 h and the digestion was quenched by the addition of acetic acid (3.5 µL, final concentration 0.5%) to achieve a final pH of the mixture below 4. Subsequently, the samples were desalted using C_{18}STAGE tips and analysed by ESI LC-MS (Supplementary Fig. 23a). Similar procedure was followed for the trypsin digestion of protein conjugates: *N*-Cys-eGFP-**1E**, *N*-Cys-eGFP-**1F**, *N*-Cys-MBP-**1D** and *N*- Cys-MBP-**1E** and their ESI mass spectra are provided in Supplementary Figs. 23b–e, respectively.

Supplementary Table 7. Sequence of labelled *N*-Cys protein bioconjugates with their trypsin cleavage sites. The cleavage sites are shown as red vertical bars and the *N*-Cys residue is highlighted in yellow circle with a modification shown as pink star.

<table>
<tr><th>POI</th><th>Sequence of protein</th></tr>
<tr><td>N-Cys labelled eGFP</td><td>★
ⒸSSMVSK|GEELFTGVVPILVELDGDVNGHK|FSVSGEGEGDATYGK|LTLK|FICTTGK|LPVPWPTLVTT
LTYGVQCFSR|YPDHMK|QHDFFK|SAMPEGYVQER|TIFFK|DDGNYK|TR|AEVK|FEGDTLVNR|IEL
K|GIDFK|EDGNILGHK|LEYNYNSHNVYIMADK|QKNGIK|VNFK|IR|HNIEDGSVQLADHYQQNTPIG
DGPVLLPDNHYLSTQSALSK|DPNEK|R|DHMVLLEFVTAAGITLGMDELYK</td></tr>
<tr><td>N-Cys labelled MBP</td><td>★
ⒸSSMK|IEEGK|LVIWINGDK|GYNGLAEVGK|K|FEK|DTGIK|VTVEHPDK|LEEK|FPQVAATGDGPD
IIFWAHDR|FGGYAQSGLLAEITPDK|AFQDK|LYPFTWDAVR|YNGK|LIAYPIAVEALSLIYNK|DLLPNP
PK|TWEEIPALDK|ELK|AK|GK|SALMFNLQEPYFTWPLIAADGGYAFK|YENGK|YDIK|DVGVDNAGA
K|AGLTFLVDLIK|NK|HMNADTDYSIAEAAFNK|GETAMTINGPWAWSNIDTSK|VNYGVTVLPTFK|G
QPSK|PFVGVLSAGINAASPNK|ELAK|EFLENYLLTDEGLEAVNK|DK|PLGAVALK|SYEEELAK|DPR|I
AATMENAQK|GEIMPNIPQMSAFWYAVR|TAVINAASGR|QTVDEALK|DAQTNGIE</td></tr>
</table>

Supplementary Fig. 23. LC-MS/MS analyses of trypsin digested IBH-labelled *N*-Cys protein conjugates. (**a**) *N*-Cys-eGFP-**1D**, (**b**) *N*-Cys-eGFP-**1E**, (**c**) *N*-Cys-eGFP-**1F**, (**d**) *N*-Cys-MBP-**1D** and (**e**) *N*- Cys-MBP-**1E**. The intact MS of the *N*-Cys labelled fragments of the conjugates are shown on the left and their MS/MS spectra on the right. The observed labelled peptide mass is shown in black and the corresponding theoretical mass in grey within parenthesis. The sequences of labelled proteins with the trypsin cleavage sites and the site of modification are given above in Supplementary Table 7.

a

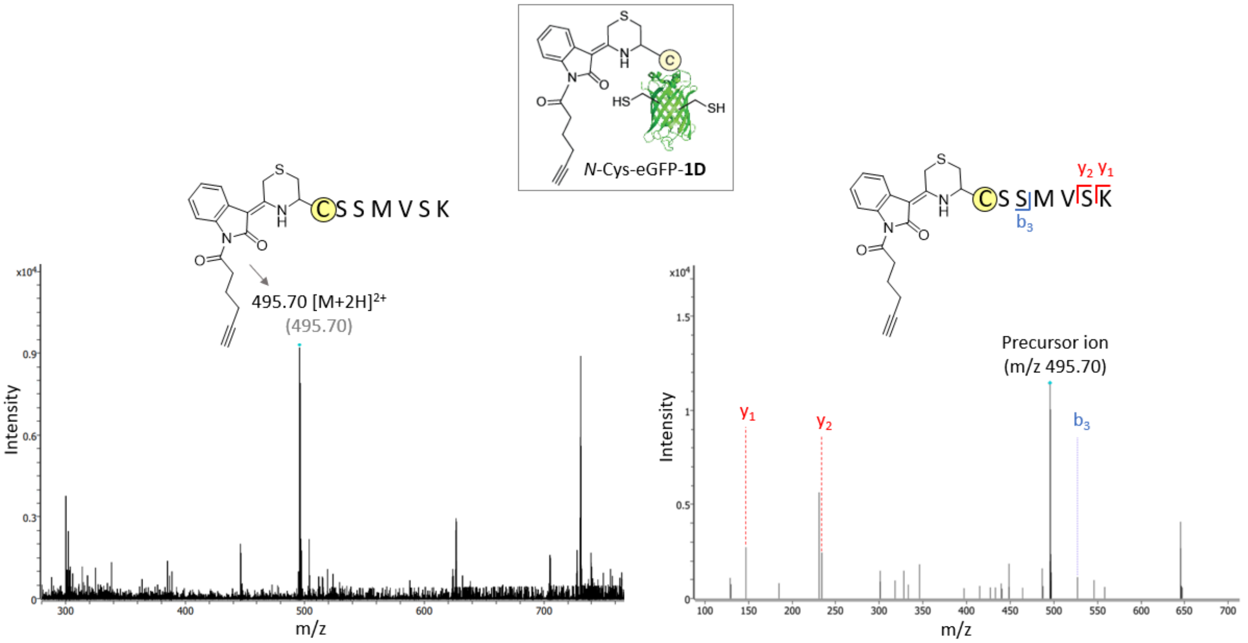

b

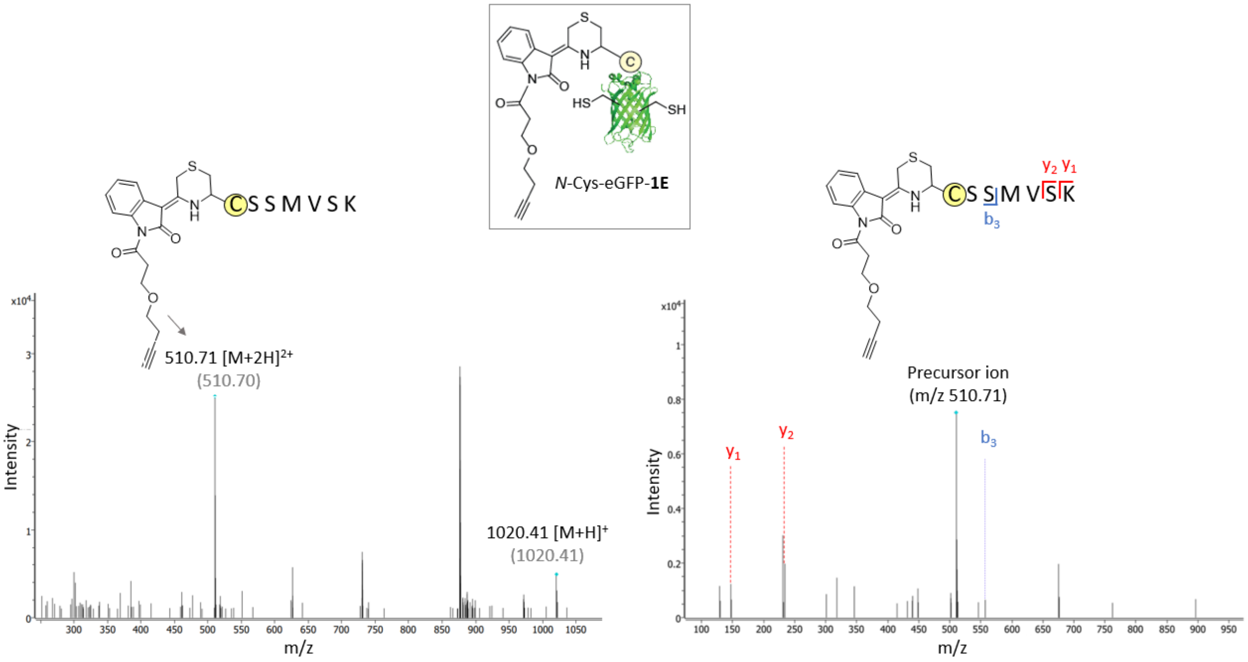

c

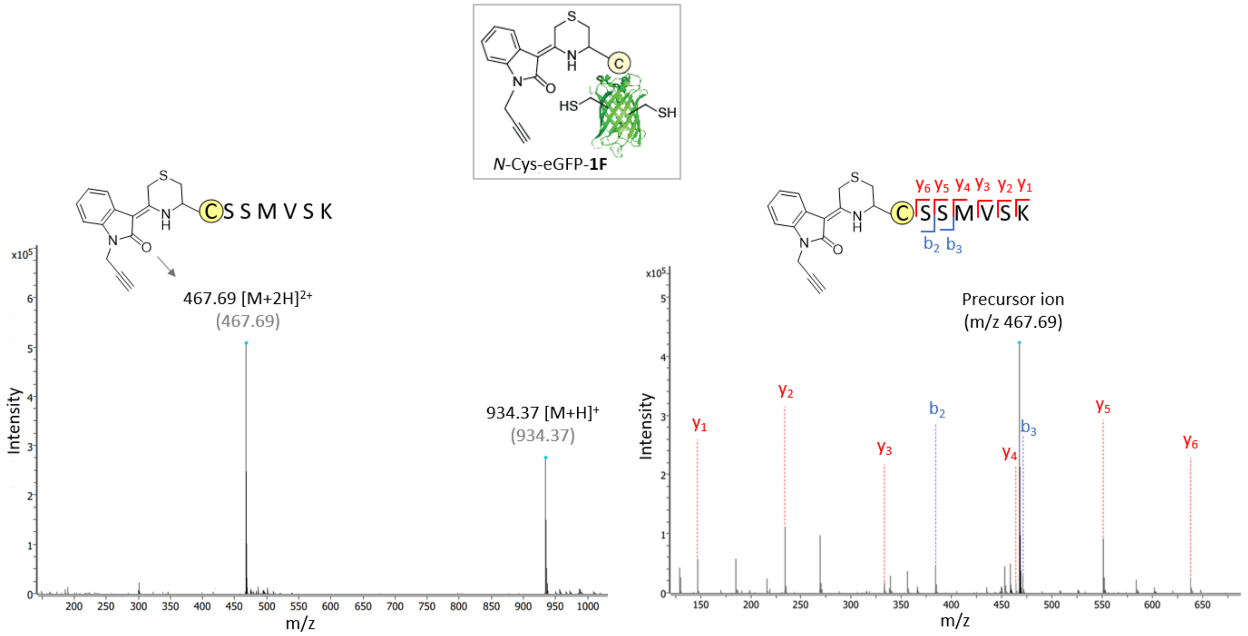

N-Cys-eGFP-1F
CSSMVSK
467.69 [M+2H]2+
(467.69)
934.37 [M+H]+
(934.37)
Intensity
m/z
Precursor ion
(m/z 467.69)

d

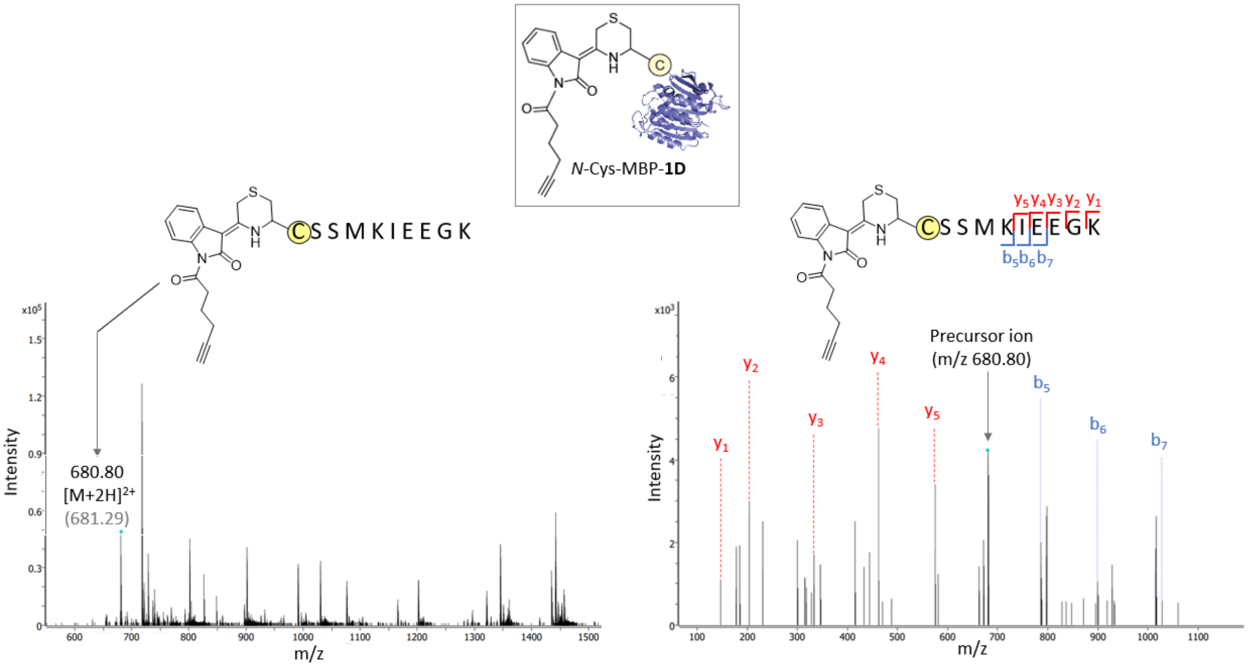

N-Cys-MBP-1D
CSSMKIEEGK
680.80
[M+2H]2+
(681.29)
Intensity
m/z
Precursor ion
(m/z 680.80)

e

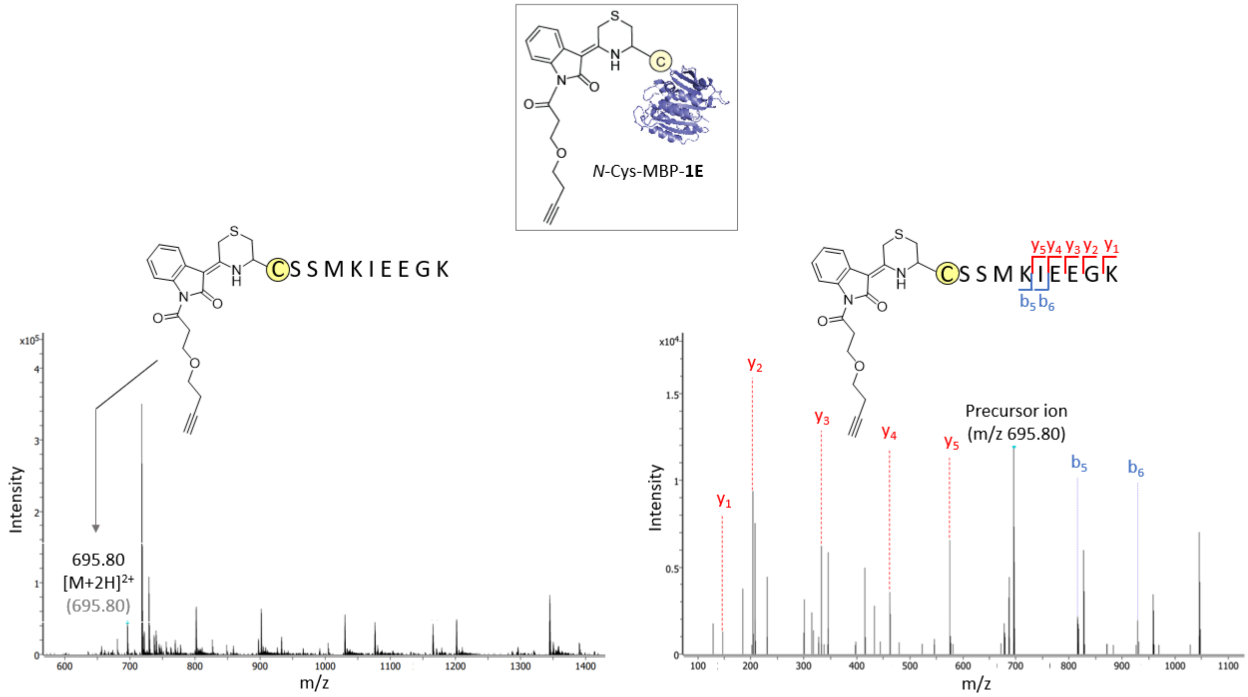

N-Cys-MBP-1E
C S S M K I E E G K
695.80
[M+2H]2+
(695.80)
Intensity
m/z
y5 y4 y3 y2 y1
b5 b6
Precursor ion
(m/z 695.80)

Supplementary Fig. 24. Labelling of *N*-Cys-POI at lower protein concentration via BHoPAL. Screening of bioconjugation reactions at low micromolar concentrations of (**a**) *N*-Cys-eGFP. (**b**) *N*-Cys-MBP and (**c**) *N*-Cys-mCherry. The reactions were performed by treating *N*-Cys-POI (100 nM–5.0 µM, 1 eq.) with *N*-acyl IBH adducts **1D**/**1E** (1–6 eq.) in sodium phosphate buffer (pH 6.5) at 25 ºC for 1 h. The observed masses of the desired bis-heterocyclic *N*-Cys protein conjugates are shown in red and their $[M+2H]^{2+}$ masses in green; and the masses for the unmodified oxidized dimer of *N*-Cys protein (POI–S–S–POI) is shown in blue. The corresponding theoretical masses are shown in grey within parenthesis. Please refer Table 1 of the main text for percentage conversions. POI = Protein of interest.

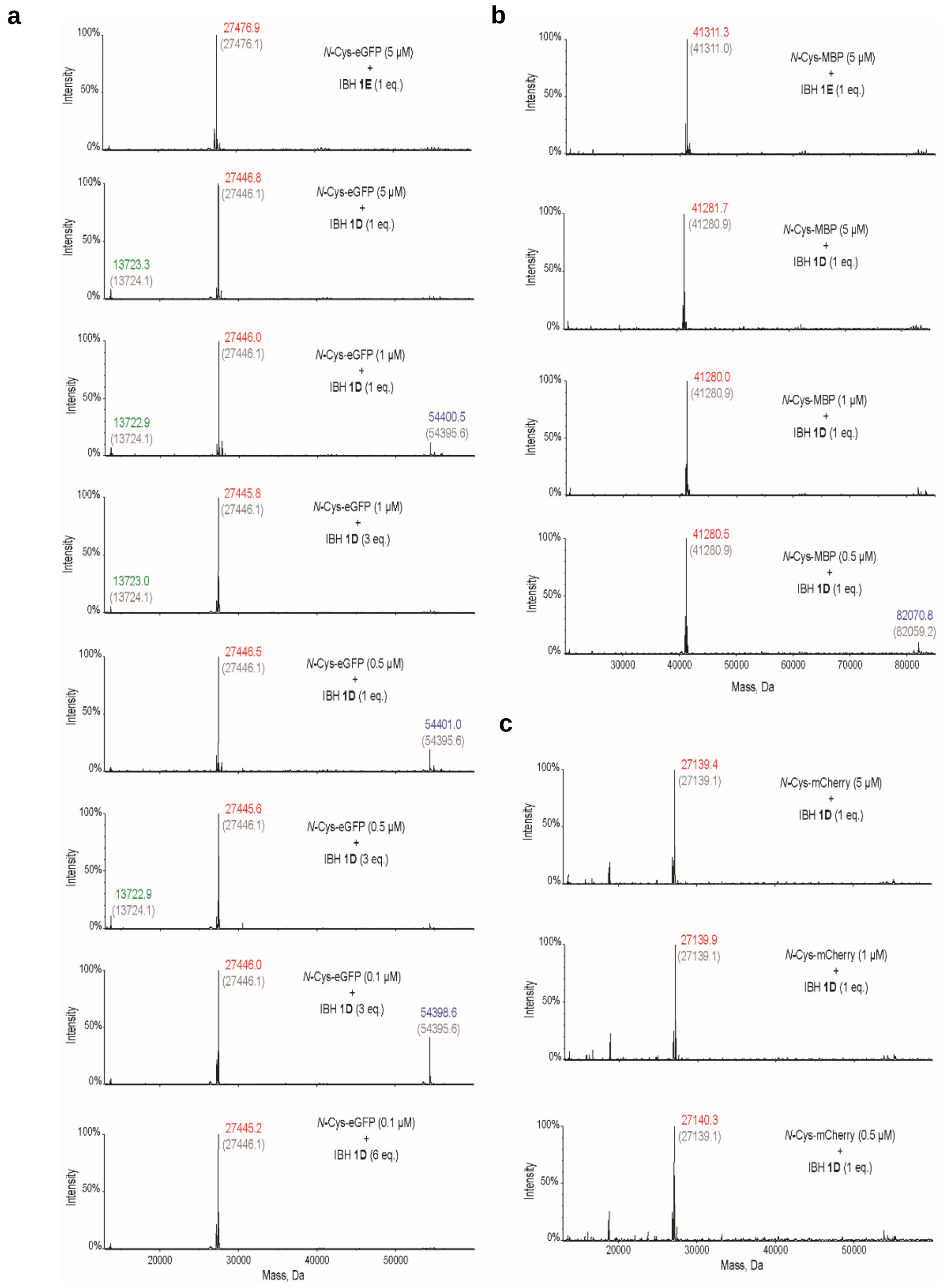

Supplementary Fig. 25. Labelling of *N*-Cys-POI with *N*-acyl IBH at low temperatures. The deconvoluted mass spectra obtained in the bioconjugation reactions performed at low temperatures with (**a**) *N*-Cys-eGFP. (**b**) *N*-Cys-MBP and (**c**) *N*-Cys-mCherry. The reactions were performed upon treating *N*-Cys-POI (10 µM, 1 eq.) with *N*-acyl IBH **1A**/**1D**/**1E** (1 eq.) in sodium phosphate buffer (pH 6.5) at 4 °C or 10 °C for 1 h. The observed masses of the desired bis-heterocyclic *N*-Cys protein conjugates are shown in red and their $[M+2H]^{2+}$ masses in green. The corresponding theoretical masses are shown in grey within parenthesis. The minor peaks in the mass spectra belong to impurities present in the recombinantly produced proteins. Please refer to Table 1 of the main text for the percentage conversions obtained in these reactions. POI = Protein of interest.

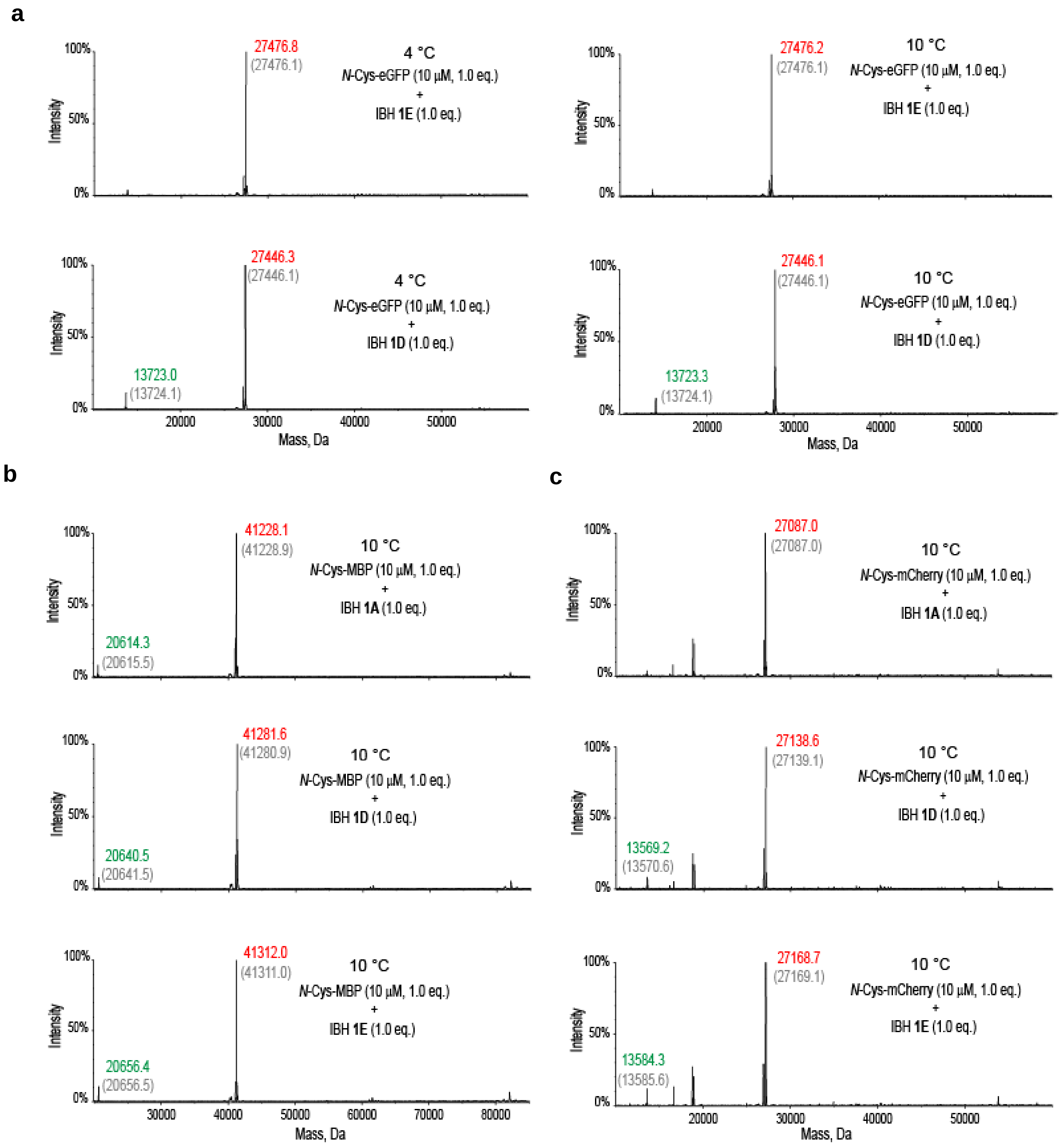

Procedure for evaluating the stability of *N*-Cys protein-IBH conjugates in the presence of GSH

N-Cys-POI conjugates (20 µL from their 0.5 mM stock, final conc. 10 µM) were incubated in sodium phosphate buffer at pH 7 (930 µL, 50 mM) containing GSH (50 µL from 100 mM stock in H_2O, final conc. 5 mM) at 37 °C for 24 h. Aliquots (250 µL) were withdrawn periodically and desalted using an Amicon ultra-0.5 10K filter before their ESI-MS analysis (Supplementary Fig. 26).

Supplementary Fig. 26. Stability of *N*-Cys-IBH protein conjugates in presence of glutathione (GSH). (**a**) *N*-Cys-eGFP-**1D**. (**b**) *N*-Cys-eGFP-**1F**. (**c**) *N*-Cys-MBP-**1D** and (**d**) *N*-Cys-MBP-**1F**. The protein conjugates (10 µM) were incubated with 5 mM GSH in sodium phosphate buffer (50 mM, pH 7) at 37 °C for 24 h. Aliquots were withdrawn at different time points and subjected to ESI-MS analysis. XIC plots acquired at different time points are shown on the left, and their deconvoluted mass spectra on the right. The observed masses of protein conjugates are shown in red and their $[M+2H]^{2+}$ masses in green. The corresponding theoretical masses are shown in grey within parenthesis.

a

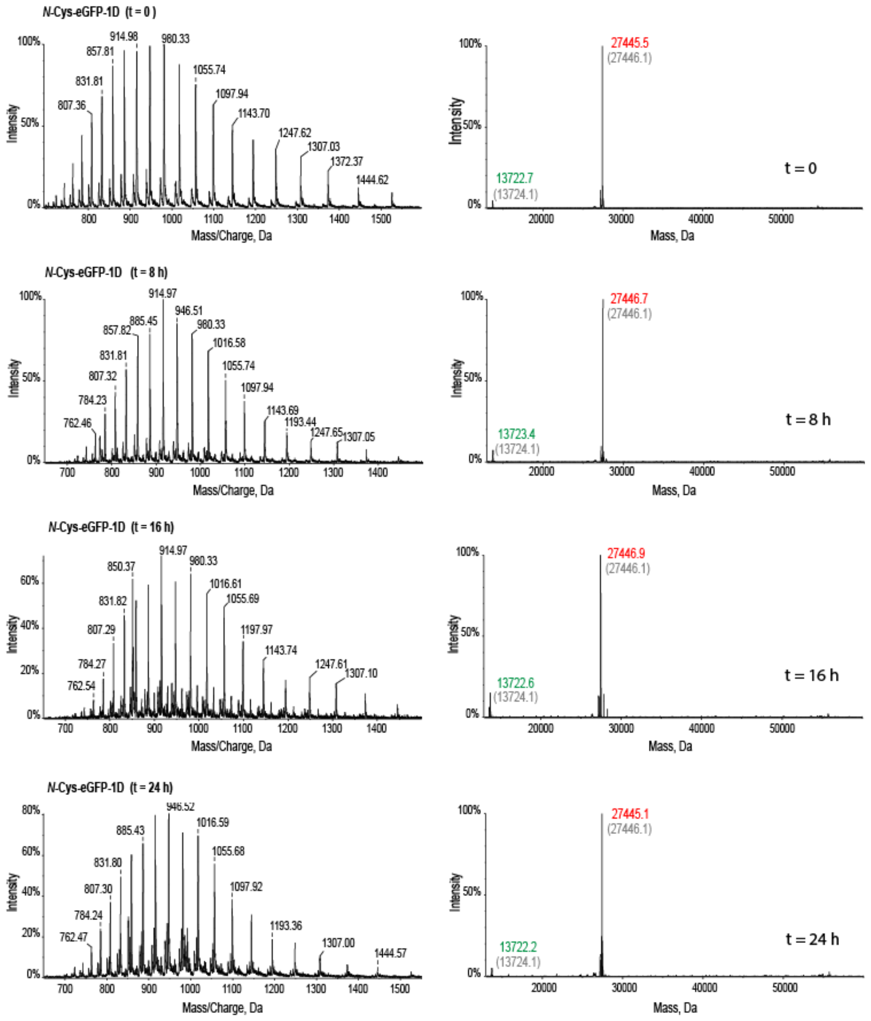

b

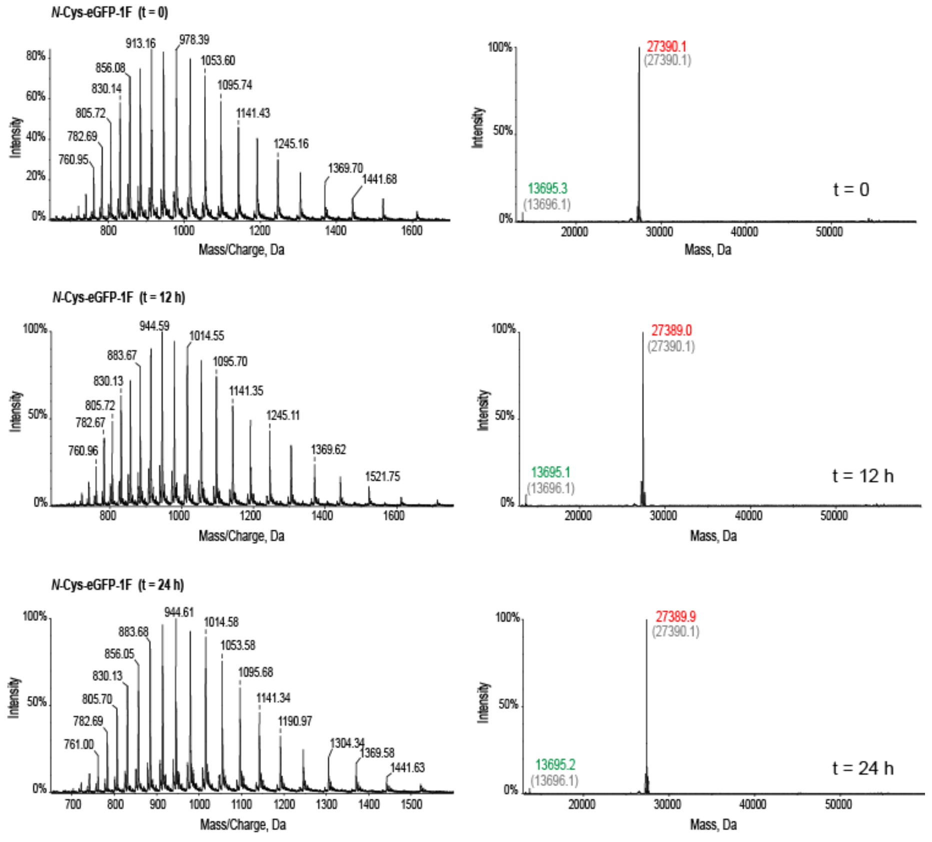
N-Cys-eGFP-1F (t = 0)
27390.1
(27390.1)
13695.3
(13696.1)
t = 0
N-Cys-eGFP-1F (t = 12 h)
27389.0
(27390.1)
13695.1
(13696.1)
t = 12 h
N-Cys-eGFP-1F (t = 24 h)
27389.9
(27390.1)
13695.2
(13696.1)
t = 24 h
Intensity
Mass/Charge, Da
Mass, Da

c

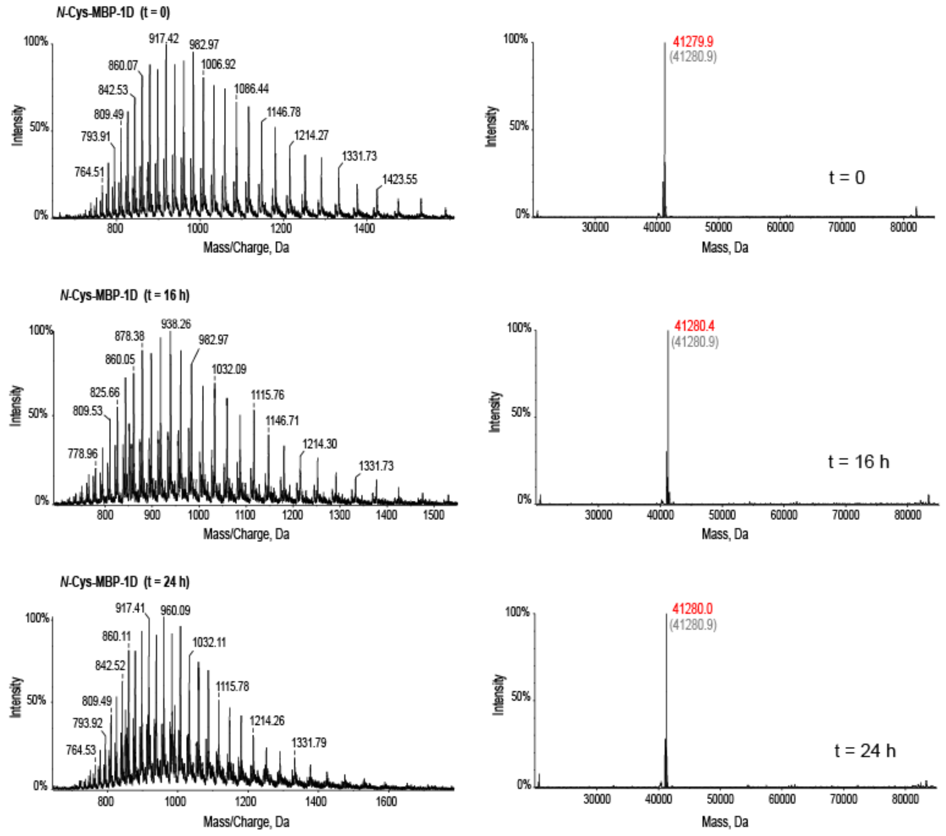
N-Cys-MBP-1D (t = 0)
41279.9
(41280.9)
t = 0
N-Cys-MBP-1D (t = 16 h)
41280.4
(41280.9)
t = 16 h
N-Cys-MBP-1D (t = 24 h)
41280.0
(41280.9)
t = 24 h
Intensity
Mass/Charge, Da
Mass, Da

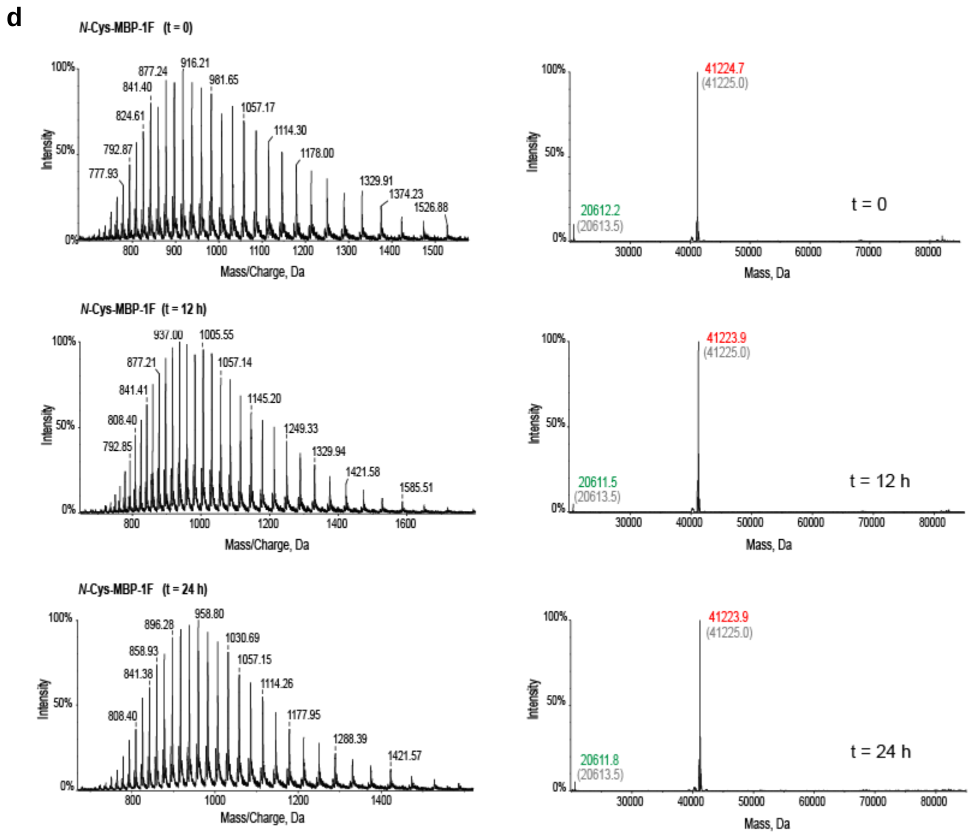

Supplementary Fig. 27. Structural integrity of IBH-POI conjugates. CD analysis of IBH-protein conjugates of (**a**) *N*-Cys-eGFP. (**b**) *N*-Cys-mCherry. (**c**) *N*-Cys-MBP and (**d**) MBP-LAP. The data was acquired using ~7 µM protein concentrations in water at 25 °C. The structural integrity of the protein remained unperturbed after their modification with IBH adducts as demonstrated by negligible changes in the CD spectra.

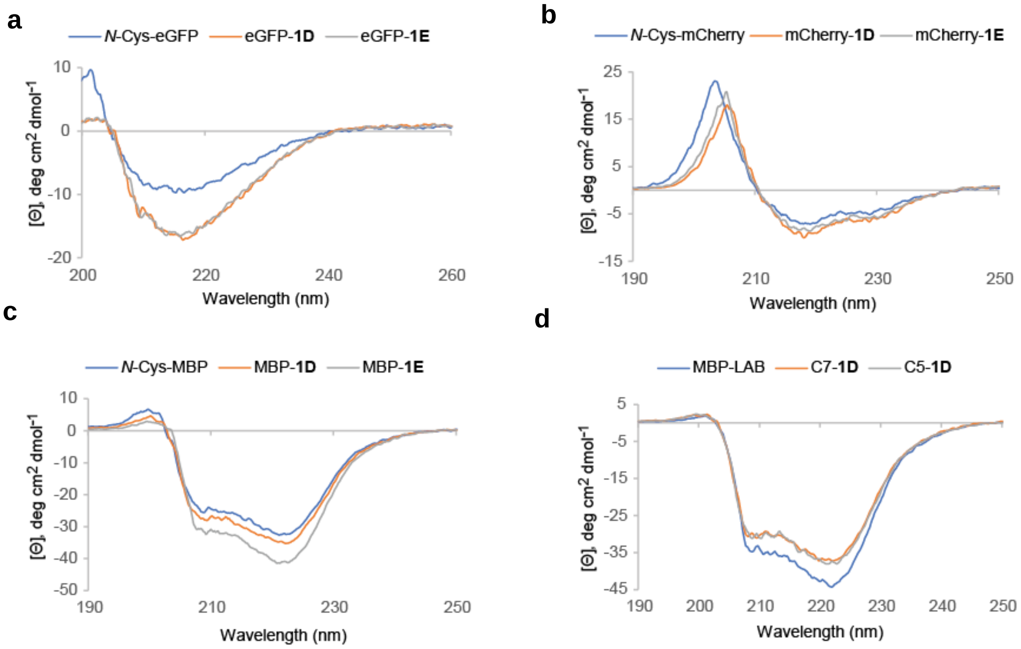

Supplementary Fig. 28. Retention of intrinsic fluorescence properties in the IBH conjugates of fluorescent proteins. Absorption (left panel) and fluorescence spectra (right panel) of (**a**) *N*-Cys-eGFP and (**b**) *N*-Cys-mCherry proteins and their IBH conjugates of **1D** and **1E**. The spectra were recorded with ~3–5 µM protein and/or conjugate concentration in water at 25 °C. λ_{ex} (eGFP) = 450 nm and λ_{ex} (mCherry) = 587 nm.

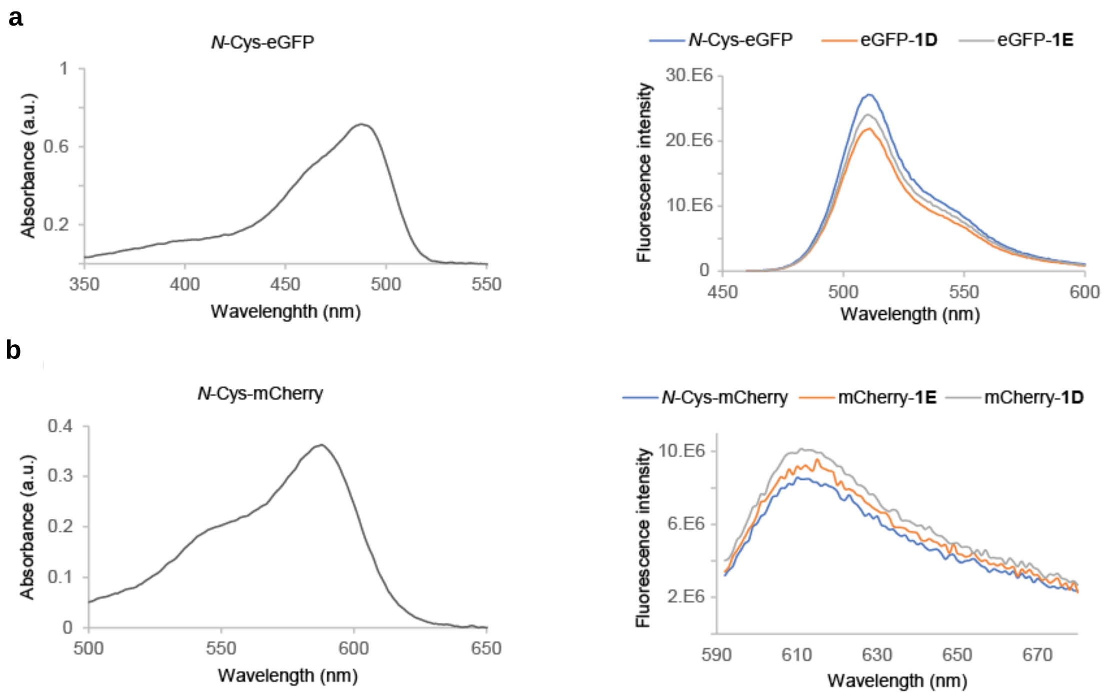

8. Fluorescent labelling of *N*-Cys-MBP via BHoPAL in a protein mixture

Prior to setting up labelling reactions, cysteine-containing proteins, *N*-Cys-MBP (containing *N*-Cys) and *N*-Gly-eGFP (containing two internal cysteines), were subjected to TCEP treatment followed by desalting using an Amicon ultra-0.5 10K filter as described above in section 7. Additionally, sodium phosphate buffer (50 mM, pH 6.5) was used in the reaction and for preparing all the proteins (*N*-Cys-MBP, *N*-Gly-eGFP, myoglobin (Mb) and lysozyme (Lyso)) stock solutions.

One-step labelling protocol: A solution of *N*-Dansyl IBH **1G** (2.5 µL, 1.5 mM in CH_3CN, final conc. 150 µM, 3.0 eq. with respect to *N*-Cys-MBP) was added to sodium phosphate buffer containing a mixture of proteins (*N*-Cys-MBP (50 µM)**,** *N*-Gly-eGFP (50 µM), Mb (60 µM) and Lyso (50 µM)) in a final volume of 25 µL at 25 °C. After 3 h of incubation, the reaction mixture was quenched by adding 2× gel loading dye (25 µL) followed by heating the mixture at 99 °C for

5 min. The resultant mixture was subjected to SDS-PAGE on a 20% polyacrylamide gel and analysed by in-gel fluorescence and Coomassie staining (Left panel of Fig. 5b of the main text). For positive control reaction, similar procedure was followed wherein **1G** was incubated in a solution containing only *N*-Cys-MBP (50 µM) in a final volume of 25 µL and for the negative control, no **1G** was used in a reaction, and an equal volume of CH_3CN was added in the reaction in place of **1G** solution. The labelling reaction was further characterized by ESI-MS analysis of the desalted reaction mixture. The MS spectra obtained are provided in Supplementary Fig. 29a.

Two-step labelling protocol: A solution of *N*-alkyne IBH **1D** (2.5 µL, 0.6 mM in CH_3CN, final conc. 60 µM, 1.2 eq. with respect to *N*-Cys-MBP) was added to sodium phosphate buffer containing a mixture of proteins (*N*-Cys-MBP (50 µM)**,** *N*-Gly-eGFP (50 µM), Mb (60 µM) and Lyso (50 µM)) in a final volume of 25 µL at 25 $^{\circ}$C. After 1 h of incubation, the reaction mixture was treated with 3 µL click reaction cocktail containing 1 mM $CuSO_4.5H_2O$ (1 µL of 30 mM stock in H_2O,) 0.5 mM TBTA (1 µL of 15 mM stock in DMSO), and 1 mM TCEP (1 µL of 30 mM stock in H_2O). Next, Dansyl azide (2 µL of 1.5 mM stock in CH_3CN, final conc. 100 µM) was added to the reaction mixture and incubated for an additional 1 h at 25 °C. The reaction was quenched by adding 2× gel loading dye (30 µL), denatured at 99 ^{o}C for 5 min, followed by in-gel fluorescence analysis and Coomassie staining (Right panel of Fig. 5b of the main text). For positive control, similar procedure was followed wherein **1D** (60 µM) was incubated in a solution containing only *N*-Cys-MBP (50 µM) in a final volume of 25 µL followed by a click reaction using Dansyl azide, and for the negative control, no Dansyl azide was used in a reaction, and an equal volume of CH_3CN was added during the click reaction in place of Dansyl azide. The labelling reaction was further characterized by ESI-MS analysis of the desalted reaction mixture. The MS spectra obtained are provided in Supplementary Fig. 29b (upper panel for step-1 and lower panel for step-2 of the labelling reaction).

Supplementary Fig. 29. Selective labelling of *N*-Cys-MBP via BHoPAL in a protein pool. ESI-MS analysis of labelling reaction via (**a**) one-step and (**b**) two-step bioconjugation. An *N*-Cys-MBP (50 µM)-spiked mixture of the eGFP (50 µM), Mb (60 µM), and Lyso (50 µM) proteins was treated with 3.0 eq. of the fluorescent *N*-Dansyl IBH adduct **1G** in sodium phosphate buffer at pH 6.5 (one-step bioconjugation), or with 1.2 eq. of the *N*-alkynyl IBH adduct **1D** followed by the fluorescent azide, $DanN_3$ (two-step bioconjugation). The detailed procedure is provided above. The TIC traces are provided in the top panel and the mass spectra depicting the deconvoluted protein masses in the bottom panel.

a

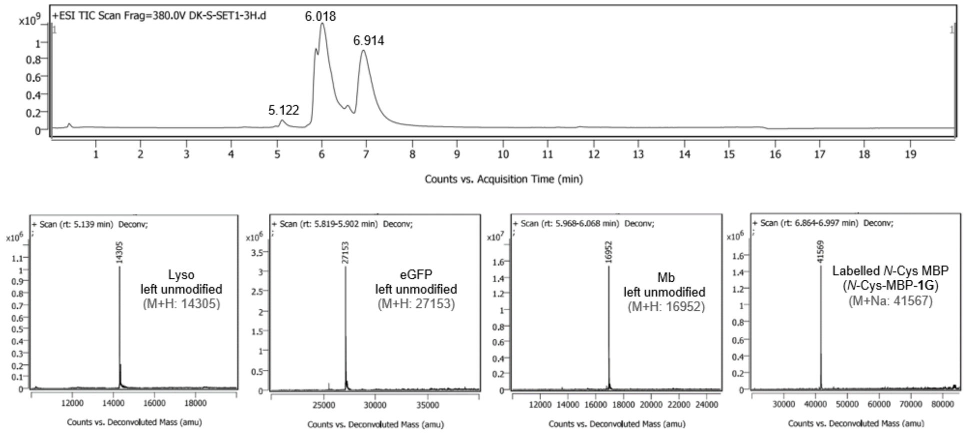

b

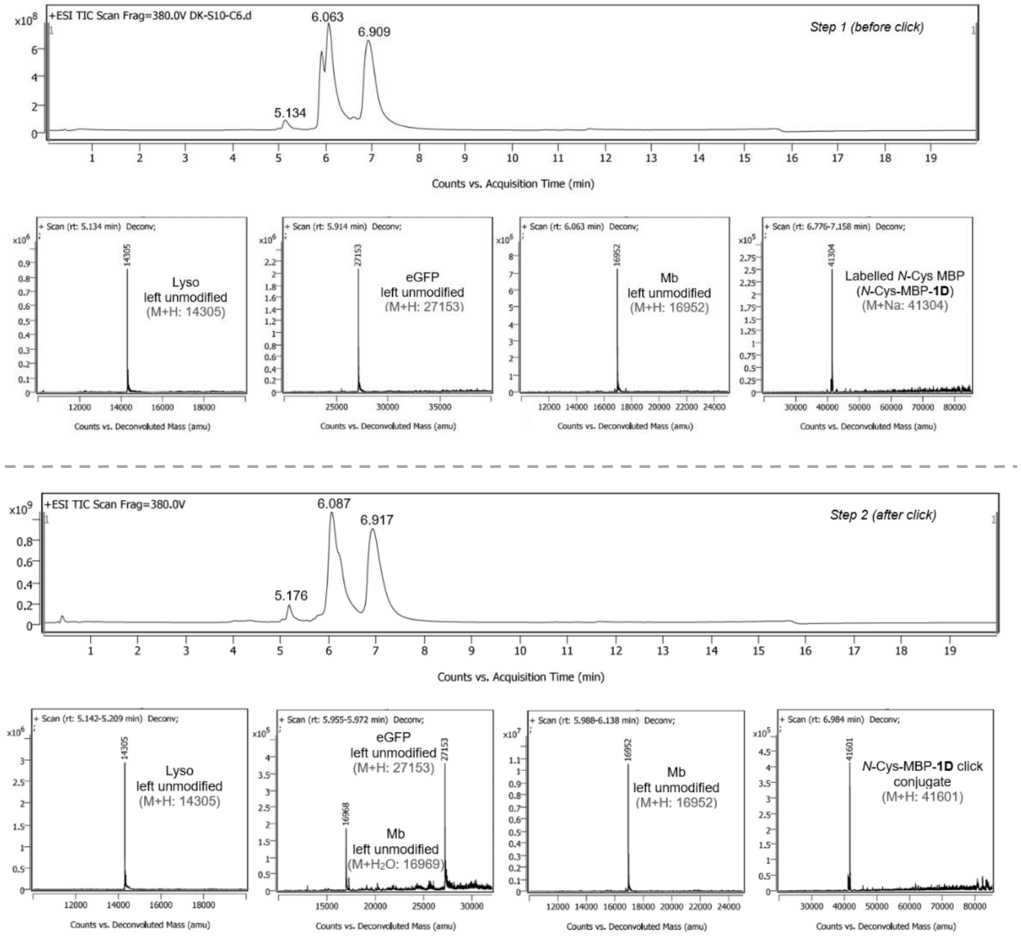

9. Fluorescent labelling of *N*-Cys of EGFR-eGFP fusion protein via BHoPAL in HEK293 cells

Transient transfection protocol: HEK293 cells were cultured in DMEM supplemented with 10% FBS and penicillin-streptomycin (100 units/mL) in T-25 culture flask under a humidified atmosphere at 37 °C containing 5% CO_2. Before cell seeding, confocal petri-dishes were coated with 0.1% poly-L-lysine. Cells at a density of 2.5×10^4 cells/mL were seeded in complete DMEM (200 μL) in 35 mm poly-L-lysine coated confocal dishes for the labelling experiment. After 24 h under standard culture conditions, cells were transfected with 150 ng of Cys-E3-EGFR-eGFP vector[10] using Lipofectamine 3000 (Lipofectamine 3000, Thermofisher scientific, Cat. no. L3000001) following manufacturer's protocol. Briefly, Lipofectamine 3000 reagent (0.375 μL) was mixed with Opti-MEM (12.5 μL) in a 0.5 mL microcentrifuge tube. In a separate tube, vector DNA (150 ng, 0.5 μL of 300 ng/μL) was mixed with the P3000 reagent (0.25 μL) in Opti-MEM (12.5 μL). After keeping both the tubes at RT for 5 min, the content of the tube containing DNA was slowly added to the tube containing Lipofectamine. The resultant transfection mix was incubated for 15 min at RT. The DMEM from the cells was replaced with Opti-MEM (174 μL), followed by the addition of a transfection mix (26 μL). After 4 h of incubation, transfection media was replaced with complete DMEM, and the cells were further allowed to grow for 48 h at 37 °C. Before imaging cells, the media was removed, and the cells were washed with 1× PBS twice, followed by the addition of nuclear staining dye (Hoechst 33342, 1 μg/mL in 1× PBS). After 5 min at RT, cells were washed with 1× PBS twice and imaged in FluoroBrite DMEM using the confocal microscope. Transfected cells were imaged in two different channels (GFP: λ_{ex} 488 nm, λ_{em} 509 nm and Hoechst 33342: λ_{ex} 350 nm, λ_{em} 460 nm).

Labelling of N-Cys of EGFR-eGFP fusion protein in cells: Before labelling, the media was removed, and the transiently transfected cells expressing EGFR-eGFP fusion protein were treated with TCEP (0.1 mM in 1× PBS, 200 μL). After 2 min at RT, TCEP was removed, followed by washing cells with 1× PBS (200 μL). To perform labelling of *N*-Cys of fusion protein, cells were incubated in 200 μL sodium phosphate buffer (50 mM, pH 6.5) containing *N*-alkyne IBH adduct **1D** (10 μL, 1 mM in DMSO, final conc. 50 μM) for 30 min at RT. The solution was removed, and the cells were subjected to CuAAC with Cy5 azide using a cell-compatible click reaction protocol[11,12]. Briefly, in this protocol, a cocktail (15 μL) was prepared by incubating $CuSO_4.5H_2O$

(5 µL, 2 mM in H_2O, final conc. 50 µM), THPTA (5 µL, 10 mM in H_2O, final conc. 0.25 mM) and sodium-L-ascorbate (5 µL, 100 mM in H_2O, final conc. 2.5 mM) for 10 min at RT and was added to 1× PBS (185 µL) containing Cy5 azide (10 µL, 0.5 mM in DMSO, final conc. 25 µM). The cells were then incubated in the resulting cocktail solution (200 µL) for 5 min at RT in the dark. The solution was removed, and the cells were washed with 1× PBS (200 µL) twice, followed by treatment with Hoechst 33342 dye (1 µg/mL in 1× PBS) for 5 min. Finally, the cells were washed with 1× PBS twice and imaged in FluoroBrite DMEM using the confocal microscope in three different channels (GFP: λ_{ex} 488 nm, λ_{em} 509 nm, Hoechst 33342: λ_{ex} 350 nm, λ_{em} 460 nm and Cy5: λ_{ex} 647 nm, λ_{em} 663 nm). The confocal fluorescence images acquired are provided Fig. 5d of the main text. Negative control was performed following a similar protocol employing an equal volume of solvent (DMSO) in place of IBH **1D** solution (Supplementary Fig. 31).

Supplementary Fig. 30. Fluorescent labelling of the *N*-Cys EGFR-eGFP fusion protein on the surface of HEK293 cells. Line intensity profile analysis for the arrowed lines depicting the green (GFP) and red (Cy5) fluorescence distribution. The overlay plot demonstrates the red fluorescence is distributed primarily at the cell periphery. Scale bar = 20 µm.

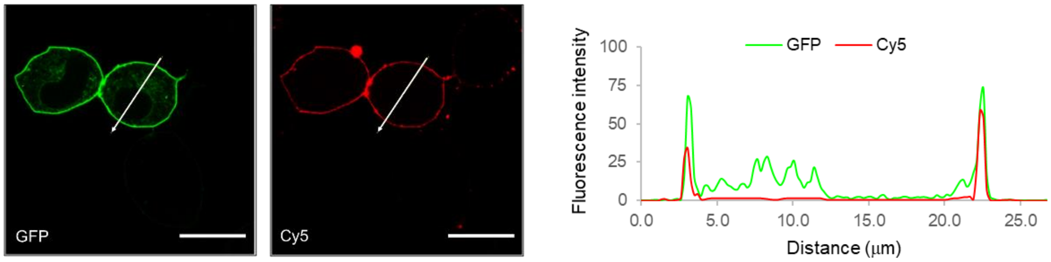

Supplementary Fig. 31. No fluorescent labelling of the *N*-Cys EGFR-eGFP upon treatment with fluorescent azide (without prior treatment with alkynyl IBH adduct, 1D). Negative control of Fig. 5c-d of the main text. HEK293 cells expressing *N*-Cys EGFR-eGFP fusion protein were treated with DMSO (no IBH **1D**) followed by treatment with click reagents in the presence of Cy5 azide. No red fluorescence due to Cy5 was observed in the cells. (GFP: λ_{ex} 488 nm, λ_{em} 509 nm, Hoechst 33342: λ_{ex} 350 nm, λ_{em} 460 nm and Cy5: λ_{ex} 647 nm, λ_{em} 663 nm).

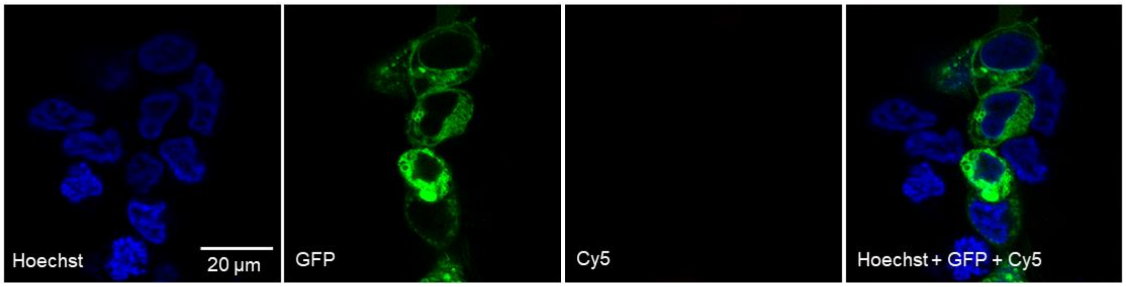

10. Docking studies on lipoic acid ligase-lipoic acid analogs interactions

The protein structure of lipoic acid (LA)-bound lipoate-protein ligase A (LplA) was downloaded from the RCSB PDB database (PDB ID: 1X2H)[13]. The W37VLplA protein was modeled by using the protein preparation wizard[14] of Schrödinger Suite[15]. The protein structure was prepared by adding hydrogen atoms, optimizing hydrogen bonds and verifying the protonation states of His, Gln and Asn. Energy minimization was carried out using default constraint of 0.3 Å RMSD and OPLS3e force field[16]. The ligand structures were optimized using the Ligand Preparation Wizard (LigPrep)[14]. The energy minimization of the ligand was performed by utilizing the OPLS3e force field. Receptor grid was generated and the TzLA analogs (C2–C7Tz) were docked to the protein using the Glide (Grid-based Ligand Docking with Energetics) docking protocol[17]. Glide module of the Extra Precision (XP) visualizer analyses the specific ligand-protein interactions. The binding free energies (ΔG) of the complexes were calculated via molecular mechanics generalized Born surface area (MMGBSA)[18]. All figures were made from Maestro[15].

In addition to the TzLA-LplA complexes described above, we also performed docking studies on the AMP-activated adducts of TzLA analogs since the LplA-catalyzed transfer of the lipoyl moiety of LA to the LAP tag proceeds via the high energy LA-AMP (lipoyl-5'-AMP) intermediate. To achieve this goal, we subjected C2–C7Tz-AMP adducts to a docking procedure similar to the one described above for TzLA analogs by employing LA-AMP bound enzyme crystal structure (PDB ID: 3A7R)[19]. The results of molecular docking on the binding of TzLA and TzLA-AMP are provided in Fig. 6b of the main text (for C7Tz), Supplementary Fig. 32 and Supplementary Table 8. These studies revealed that the binding of both TzLA and TzLA-AMP is increasingly facilitated with increasing carbon chain length, as indicated by the higher predicted Gibb's free energy (ΔG) of binding for the long-chain analogs over their short-chain analogs (Supplementary Table 8).

Supplementary Fig. 32. Molecular docking of thiazolidine-appended lipoic acid and their AMP complex to LplA. Two-dimensional (2D) interactions of the (**a**) thiazolidine appended-lipoic acid analogs (C2–C7Tz) and (**b**) their AMP adducts (C2–C7Tz-AMP), complexed with $LplA^{W37V}$ protein. The detailed information about the docking procedure is provided above.

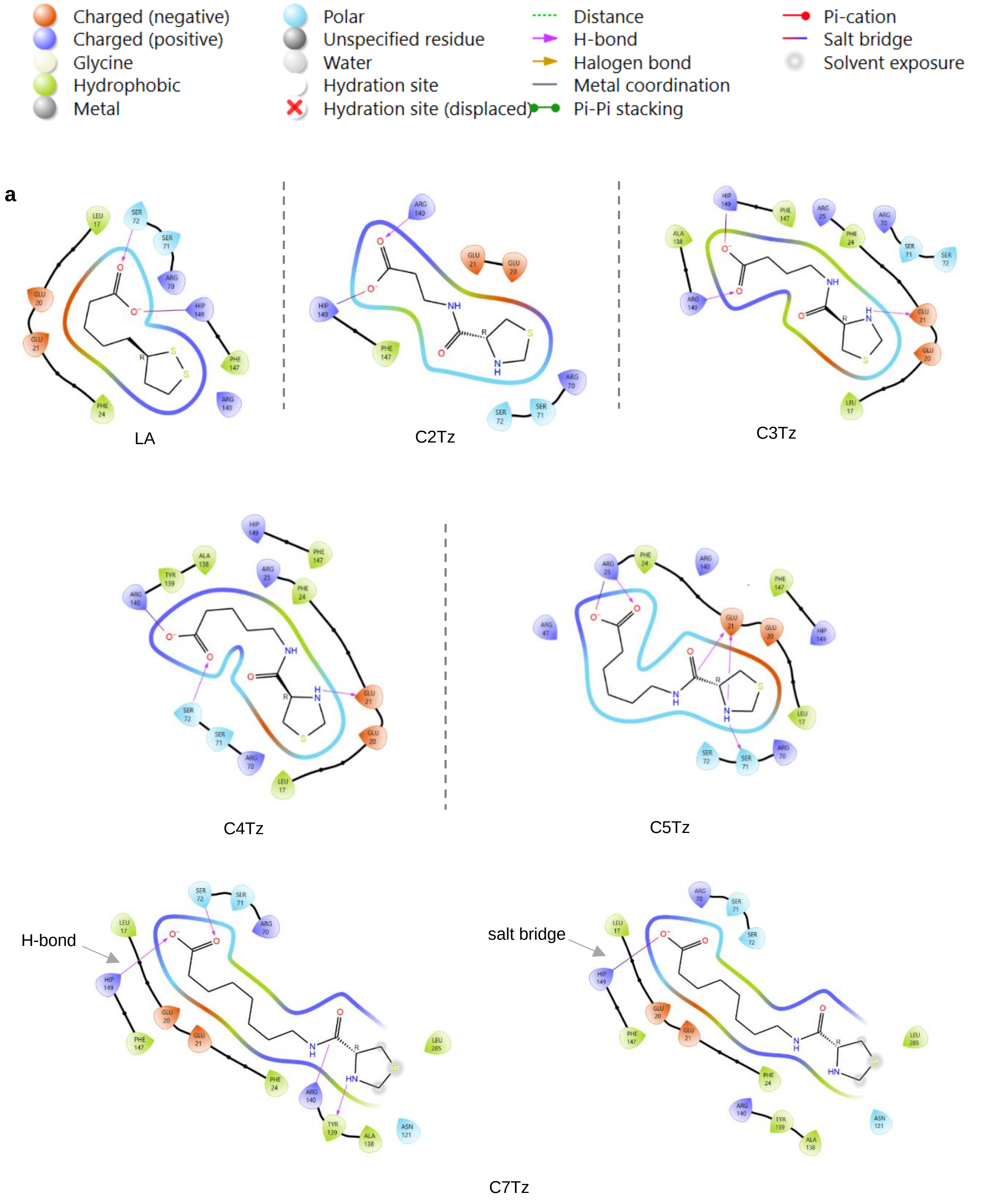

b

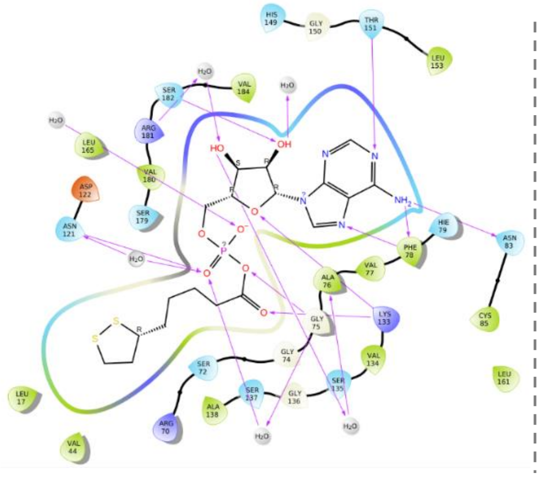

LA-AMP

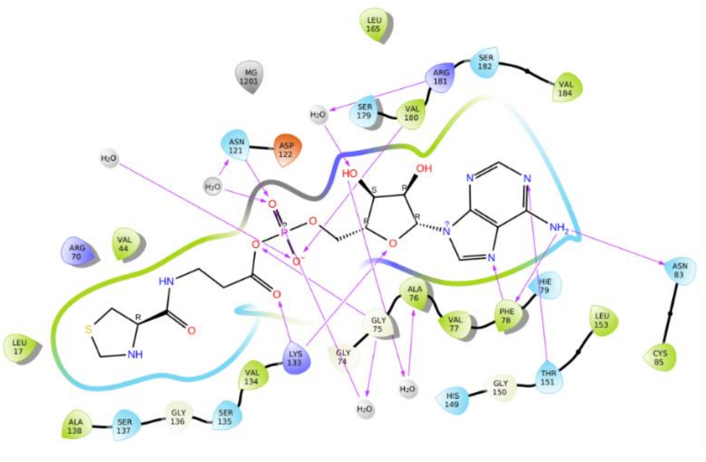

C2Tz -AMP

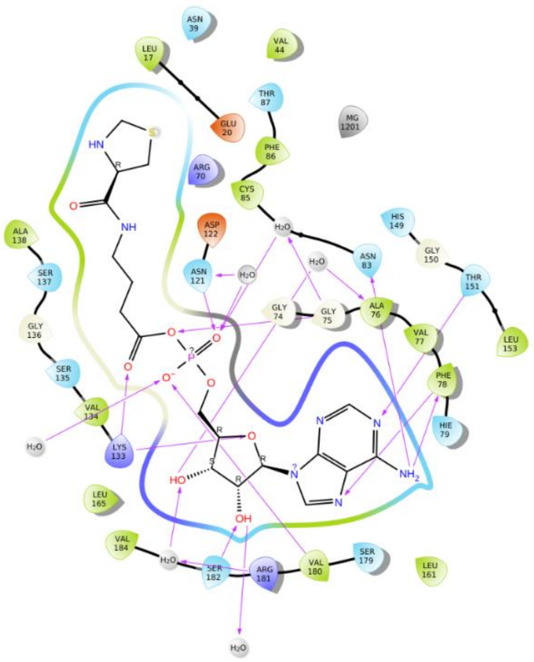

C3Tz -AMP

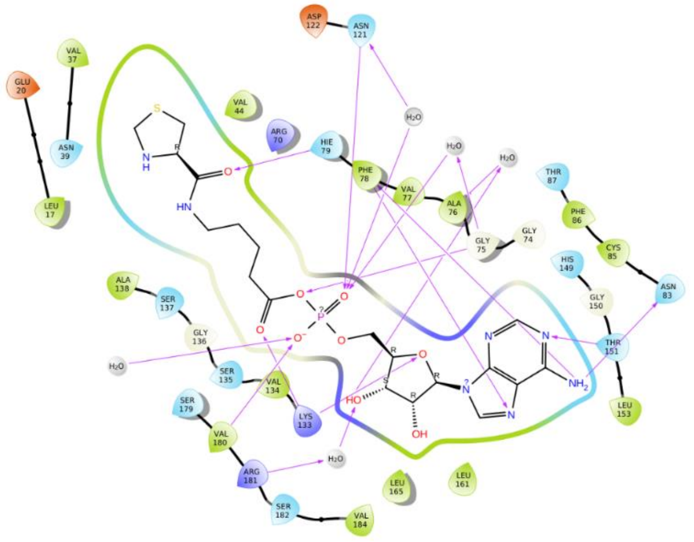

C4Tz -AMP

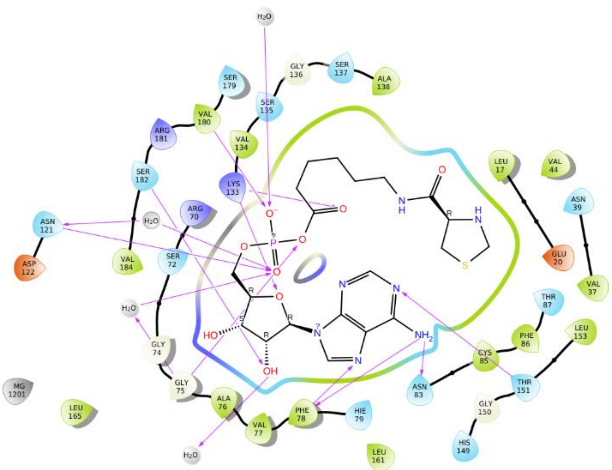

C5Tz -AMP

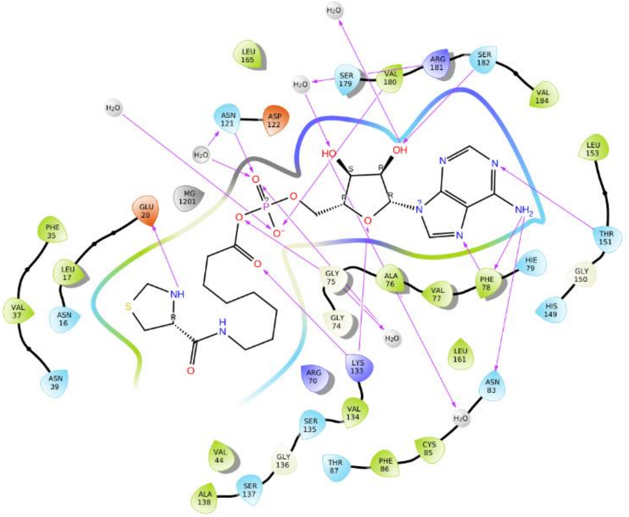

C7Tz -AMP

Supplementary Table 8. Binding energies (ΔG) of LA analogs C2–C7Tz and their AMP adducts complexed with LplA^{W37V}

Structure of LA analog	ΔG (Kcal/mol)	Structure of Lipoyl-AMP complex	ΔG (Kcal/mol)
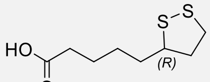 LA	-59.29	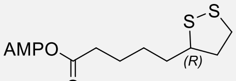 LA-AMP	-139.29
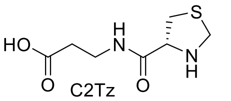 C2Tz	-45.84	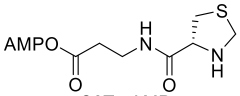 C2Tz-AMP	-91.22
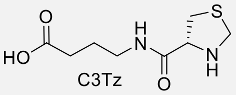 C3Tz	-49.02	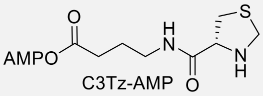 C3Tz-AMP	-125.92
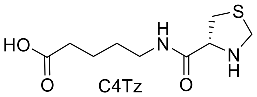 C4Tz	-61.84	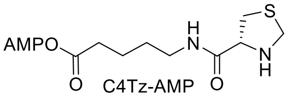 C4Tz-AMP	-122.87
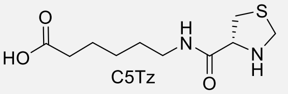 C5Tz	-58.59	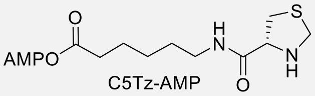 C5Tz-AMP	-123.73
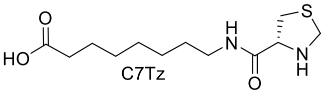 C7Tz	-67.32	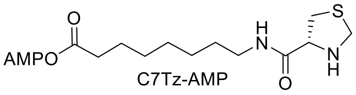 C7Tz-AMP	-151.11

11. Recombinant production of LpIA^{W37V}

The plasmid encoding LplA^{W37V} (pYFJ16-LplA(W37V); Addgene Cat. no. 34838) was transformed into competent *E. coli* cells, BL21(DE3). A single colony of transformed cells was

inoculated into Luria Bertani (LB) media supplemented with antibiotic ampicillin (100 μg/mL). The cells were cultured at 37 ºC with continuous shaking at 220 rpm. After 12 h, the cell culture was diluted with LB media (1:100, 500 mL) and the cells were grown further under the same conditions to an optical density (OD_{600}) of 0.5–0.6. Next, the protein expression was induced by adding IPTG (0.5 mM) inducer. The cells were cultured at 25 °C, 220 rpm for 10 h and harvested by centrifugation (5000 g for 20 min) at 4 °C. Cell pellet from a 500 mL culture was resuspended in lysis buffer (20 mL) at pH 7.5 containing 50 mM sodium phosphate, 300 mM NaCl, 10 mM imidazole and 1 mM PMSF. The cells were lysed by sonication for 3 min at 4 °C (20 s on, 59 s off, 40% amplitude). The cell debris were removed by centrifugation (20,000 g for 1 h) at 4 °C. The supernatant was separated and mixed with Ni-NTA agarose resin at 4 °C for 1 h to facilitate the binding of His-tagged protein. The resin was then loaded on a poly-prep chromatography column (Cat. no. 731-1550) and washed with 10 column volumes of wash buffer at pH 7.5 containing 50 mM sodium phosphate, 300 mM NaCl and 20 mM imidazole. His-tagged protein was eluted with buffer at pH 7.5 containing imidazole (30 → 250 mM), 50 mM sodium phosphate and 300 mM NaCl, and the collected fractions were analyzed by SDS-PAGE for purity (Supplementary Fig. 33). The pure protein fractions were pooled and dialyzed (membrane cut off 12–14 kDa) overnight at 4 °C against storage buffer (1× PBS). The concentration of the dialyzed protein was measured at A280 using nanodrop. The His-tagged LpIA^{W37V} was stored at -80 ºC after snap-freezing at a concentration of 2 mg/mL.

Supplementary Fig. 33. SDS-PAGE analysis of purified His-tagged LplA^{W37V}. The numbers on the gel images denote the protein marker size in kDa.

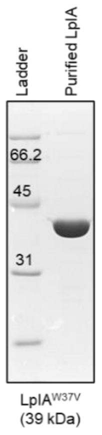

12. HPLC analysis of ligation reaction of thiazolidine-appended lipoic acid analogs on to LAP peptide

Enzyme-mediated ligation of lipoic acid (LA) on LAP peptide (control reaction):

To check the activity of expressed $LplA^{W37V}$ enzyme (section 10 above), a ligation reaction of lipoic acid (LA) on its recognition peptide (LAP peptide: GFEIDKVWYDLDA) was performed and the reaction was analyzed via HPLC. The schematic representation is provided in Fig. 6a of the main text or Supplementary Fig. 34a. The reaction was set up by adding 3 μM enzyme (5 μL from 60 μM stock in 1× PBS) to sodium phosphate buffer at pH 7 (90 μL, 25 mM) containing LAP (250 μM, 12.5 μL from 2 mM stock in reaction buffer), $Mg(OAc)_2$ (5 mM, 5 μL from 100 mM stock in water) and LA (0.5 mM, 5 μL, 10 mM stock in 50% ethanol/H_2O). The reaction was initiated by adding ATP (5 mM, 5 μL from 100 mM stock in reaction buffer) followed by incubating the reaction mixture at 37 °C with continuous shaking (300 rpm). After 45 min, the reaction was quenched by adding EDTA (50 mM, 100 μL from 0.5 M stock in water, the pH of which was adjusted to 8 with 1N NaOH to solubilize EDTA) and the formation of product was analyzed by HPLC using a reverse-phase C_{18} column (Please see section 1 for column specification and HPLC method). The chromatograms were recorded at 210 nm, and the newly formed peaks in the HPLC traces were characterized by HRMS (Supplementary Table 9). The percentage product formed in the reaction was calculated from the area under peak using the equation %Product = 100 – %LAP remaining. For negative control reactions, either ATP or LA was omitted from the reaction, and an equal volume of respective solvent was added in their place. The HPLC traces of the reaction are provided in Supplementary Fig. 34b and the % yield of the ligation product obtained in the reaction is provided in Fig. 6d of the main text.

Enzyme-mediated ligation of thiazolidine-appended LA analogs (C2–C7Tz) on LAP peptide:

To monitor the ligation reaction of thiazolidine-appended LA analogs (C2–C7Tz) on LAP peptide, the ligation reactions were set up following similar procedure as described above for the ligation of LA on LAP peptide. In these reactions, C2–C7Tz analogs were employed in place of LA. Three reaction conditions were screened with varying concentrations of enzyme and LA analogs, as mentioned below:

Condition A: Enzyme (3 μM), LA analogs (0.5 mM), LAP (250 μM), $Mg(OAc)_2$ (5 mM) and ATP (5 mM)

Condition B: Enzyme (5 μM), LA analogs (0.5 mM), LAP (250 μM), $Mg(OAc)_2$ (5 mM) and ATP (5 mM)

Condition C: Enzyme (10 μM), LA analogs (1.25 mM), LAP (250 μM), $Mg(OAc)_2$ (5 mM) and ATP (5 mM)

After setting up the reactions under different conditions (conditions A–C), aliquots (10 µL) from the reaction mixture were periodically withdrawn and injected in the HPLC to monitor the progress of ligation reaction. The newly formed peaks observed in these reactions were characterized by HRMS (Supplementary Table 9), and the percentage product was calculated from the area under peak as described above in the LA ligation reaction. The schematic representation is provided in Fig. 6a of the main text or Supplementary Fig. 34a. All the HPLC traces obtained in the reactions of C2–C7Tz with LAP under different conditions are given in Supplementary Figs. 34c–g, respectively and the % yield obtained in these reactions are provided in Fig. 6d of the main text.

Supplementary Fig. 34. HPLC traces for the screening of LplA^{W37V} mediated ligation of LA and thiazolidine-appended LA analogs (C2–C7Tz) on the LAP peptide. (**a**) General schematic of the reaction and the ligation conditions screened. (**b**) LA ligation reaction. No conjugate formation was obtained in negative controls omitting ATP and LA. (**c**) C2Tz reaction. (**d**) C3Tz reaction. (**e**) C4Tz reaction. (**f**) C5Tz reaction and (**g**) C7Tz reaction. The percentage yields observed in the ligation reactions are summarized in Fig. 6d of the main text. All the LAP-conjugates were characterized by HRMS (Supplementary Table 9). The numbers within parenthesis next to the individual peaks in the HPLC traces denote retention time. No conjugate formation was observed in the ligation reactions with C2Tz and C3Tz.

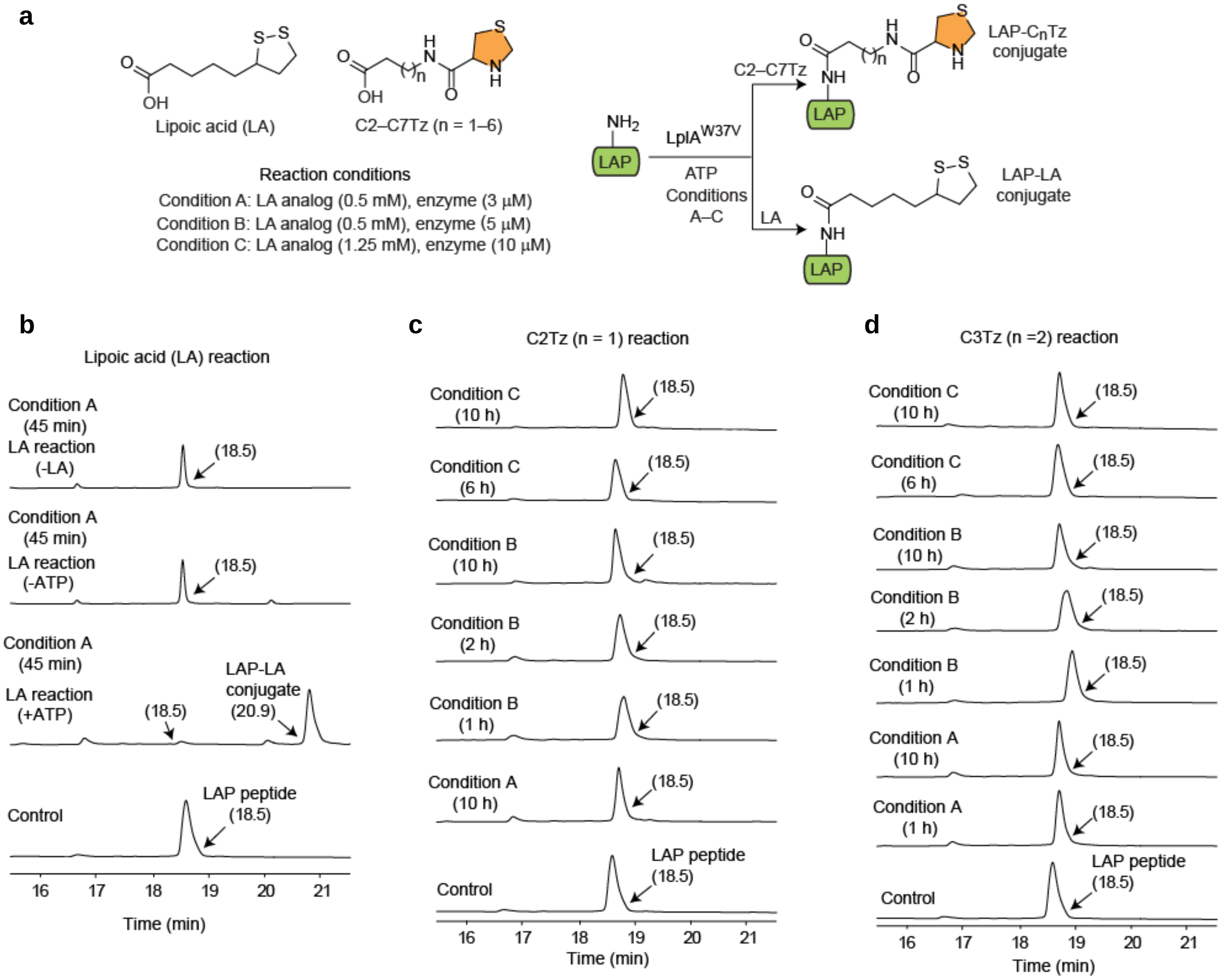

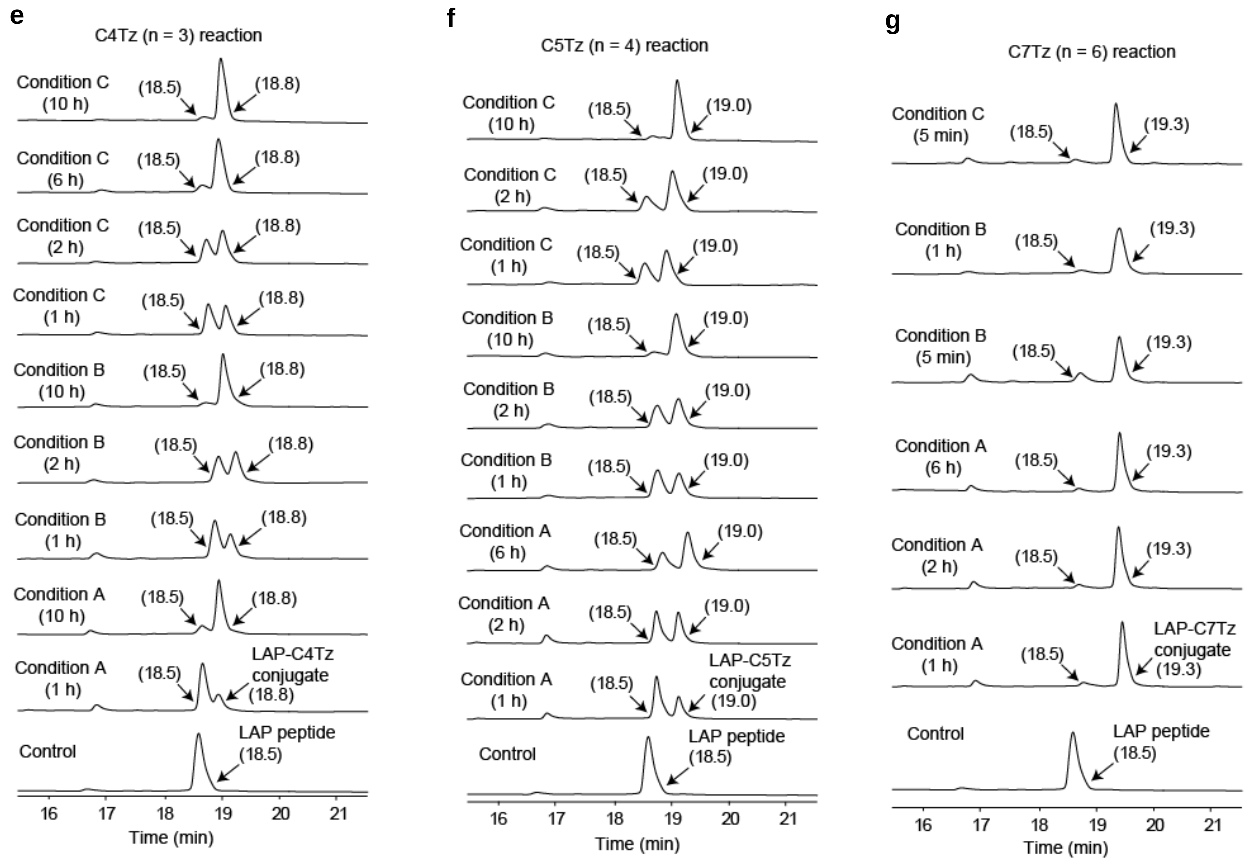

Supplementary Table 9. Theoretical and observed masses (HRMS) of LAP-conjugates

Peptide conjugate	Comp. code	Molecular formula	Calculated mass	Obtained mass
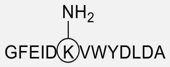	LAP	$C_{74}H_{103}N_{15}O_{23}$	785.875 $[M+2H]^{2+}$<br>1570.742 $[M+H]^{+}$	785.876 $[M+2H]^{2+}$<br>1570.745 $[M+H]^{+}$
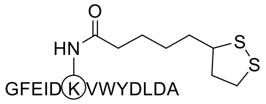	LAP-LA	$C_{82}H_{115}N_{15}O_{24}S_2$	879.891 $[M+2H]^{2+}$<br>1758.775 $[M+H]^{+}$	879.897 $[M+2H]^{2+}$<br>1758.784 $[M+H]^{+}$
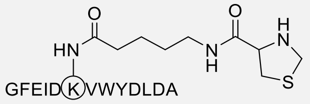	LAP-C4Tz	$C_{83}H_{117}N_{17}O_{25}S$	892.913 $[M+2H]^{2+}$<br>1784.820 $[M+H]^{+}$	892.914 $[M+2H]^{2+}$<br>1784.809 $[M+H]^{+}$
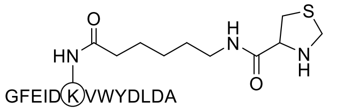	LAP-C5Tz	$C_{84}H_{119}N_{17}O_{25}S$	899.921 $[M+2H]^{2+}$<br>1798.835 $[M+H]^{+}$	899.920 $[M+2H]^{2+}$<br>1798.831 $[M+H]^{+}$
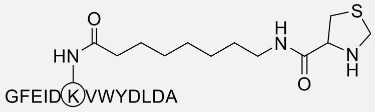	LAP-C7Tz	$C_{86}H_{123}N_{17}O_{25}S$	913.937 $[M+2H]^{2+}$<br>1826.867 $[M+H]^{+}$	913.938 $[M+2H]^{2+}$<br>1826.866 $[M+H]^{+}$

13. Recombinant production of MBP-LAP protein

Plasmid construction (cloning): Plasmids were constructed via site-directed mutagenesis by employing the insertion method. The sequences of the primers used are provided in Supplementary Table 10.

Supplementary Table 10. Sequence of primers employed for molecular cloning.

Primer name	Sequence (5'-3')
MBPLAP1-F	GCAGGTGGTGGCGGTATTGGAAGTGGATAACGGATCCGCG
MBPLAP1-R	ACCGCCACCACCTGCATTGGATTGGAAGTACAGGTTTTCCTCG
MBPLAP2-F	AGCGGTTTCGAAATTGATAAAGTTATTGGAAGTGGATAACGGATCCGCG
MBPLAP2-R	AACTTTATCAATTTCGAAACCGCTACCGCCACCACCTGCATTGGATTG
MBPLAP3-F	TGGTATGACCTGGATGCATAAATTGGAAGTGGATAACGGATCCGCG
MBPLAP3-R	TTATGCATCCAGGTCATACCAAACTTTATCAATTTCGAAACCGCTACCGCC

His-MBP-LAP construct:

The pET His6 MBP TEV LIC cloning vector containing a 6×His-MBP-TEV gene construct (2M-T) was purchased from Addgene (Cat. no. 29708). A LAP tag with flanking linker (AGGGGS-GFEIDKVWYDLDA) was inserted after the TEV site in the plasmid by site-directed mutagenesis in 3 PCR reactions following the protocol described above in section 6. The primers employed in the cloning are given in Supplementary Table 10. The product of each PCR reaction served as a template for the next PCR reaction, and all the insertions were confirmed by sequencing data (Supplementary Fig. 35).

PCR 1: Primers (forward MBPLAP1-F and reverse MBPLAP1-R) and construct generated (6×His-MBP-TEV-AGGGG)

PCR 2: Primers (forward MBPLAP2-F and reverse MBPLAP2-R) and construct generated (6×His-MBP-TEV-AGGGGS-GFEIDKV)

PCR 3: Primers (forward MBPLAP3-F and reverse MBPLAP3-R) and construct generated (6×His-MBP-TEV-AGGGGS-GFEIDKVWYDLDA)

Supplementary Fig. 35. . Sequence analysis of LAP-tag inserted MBP construct generated via three PCR steps.

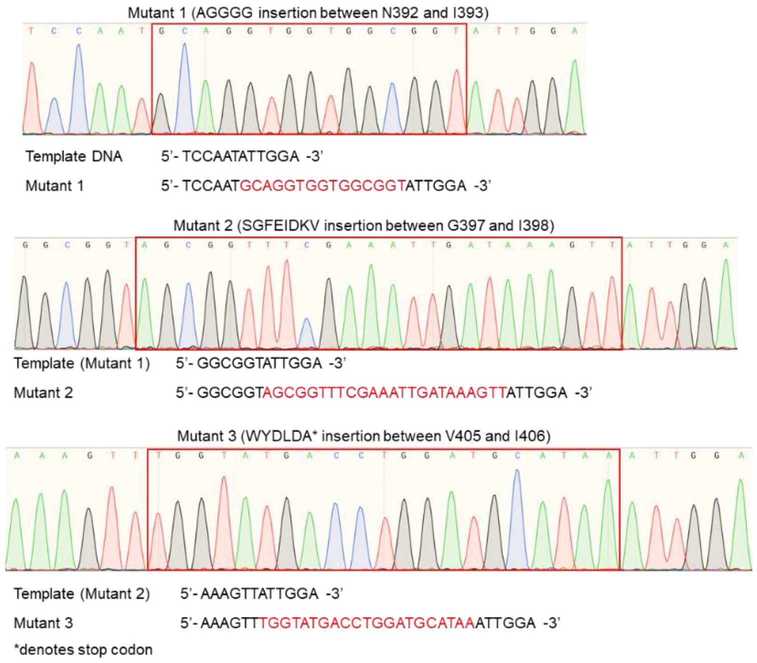

Protein expression, purification and storage:

The plasmid encoding His-MBP-LAP was transformed into competent *E. coli* cells, BL21(DE3) pLysS. A single colony of transformed cells was inoculated into Luria Bertani (LB) media supplemented with antibiotic ampicillin (100 μg/mL) and chloramphenicol (35 μg/mL). The cells were cultured at 37 ºC with continuous shaking at 220 rpm. After 12 h, the cell culture was diluted with LB media (1:100, 500 mL) and the cells were grown further under the same conditions to an optical density (OD_{600}) of 0.5–0.6. Next, the protein expression was induced by adding IPTG (1 mM) inducer. The cells were cultured at 25 °C, 220 rpm for 8–10 h and harvested by centrifugation (5000 g for 20 min) at 4 °C. Cell pellet from a 500 mL culture was resuspended in lysis buffer (20 mL) at pH 7.5 containing 50 mM sodium phosphate, 500 mM NaCl, 10 mM imidazole and 1 mM PMSF. The cells were lysed by sonication for 3 min at 4 °C (20 s on, 59 s off, 40% amplitude). The cell debris were removed by centrifugation (20,000 g for 1 h) at 4 °C. The supernatant was separated and mixed with Ni-NTA agarose resin at 4 °C for 1 h to facilitate the binding of His-tagged protein. The resin was then loaded on a poly-prep chromatography column (Cat. no. 731-1550) and washed with 10 column volumes of wash buffer at pH 7.5 containing 50 mM sodium phosphate, 500 mM NaCl and 20 mM imidazole. His-tagged

protein was eluted with buffer at pH 7.5 containing imidazole (30 → 250 mM), 50 mM sodium phosphate and 500 mM NaCl, and the collected fractions were analyzed by SDS-PAGE for purity (Supplementary Fig. 36). The pure protein fractions were pooled and dialyzed (membrane cut off 12–14 kDa) overnight at 4 °C against storage buffer (1× PBS). The concentration of the dialyzed protein was measured at A280 using nanodrop. The His-tagged MBP-LAP was stored at -80 ºC after snap-freezing at a concentration of 2 mg/mL. The sequence of the protein is provided in Supplementary Table 11.

Supplementary Fig. 36. SDS-PAGE analysis of purified MBP-LAP. The numbers on the gel images denote the protein marker size in kDa.

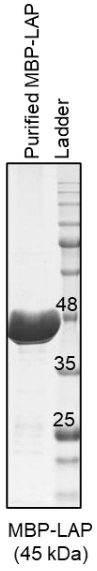

Supplementary Table 11. Sequence of expressed recombinant His-MBP-LAP used for bioconjugation. The mutations are underlined.

Protein	Sequence
**His-MBP-LAP**	MKSSHHHHHHGSSMKIEEGKLVIWINGDKGYNGLAEVGKKFEKDTGIKVTVEHPDKLEEKFPQVAATGDG<br>PDIIFWAHDRFGGYAQSGLLAEITPDKAFQDKLYPFTWDAVRYNGKLIAYPIAVEALSLIYNKDLLPNPPKTW<br>EEIPALDKELKAKGKSALMFNLQEPYFTWPLIAADGGYAFKYENGKYDIKDVGVDNAGAKAGLTFLVDLIKN<br>KHMNADTDYSIAEAAFNKGETAMTINGPWAWSNIDTSKVNYGVTVLPTFKGQPSKPFVGVLSAGINAASP<br>NKELAKEFLENYLLTDEGLEAVNKDKPLGAVALKSYEEELAKDPRIAATMENAQKGEIMPNIPQMSAFWYA<br>VRTAVINAASGRQTVDEALKDAQTNGIEENLYFQSNAGGGGSGFEIDKVWYDLDA

14. Procedure for incorporating the 1,2-aminothiol moiety within the MBP-LAP protein followed by its modification via BHoPAL

Step 1: Ligation of C5Tz/C7Tz on the LAP tag of MBP-LAP

Before the ligation reaction, the snap-frozen enzyme stocks of MBP-LAP in 1× PBS (section 13 above) were thawed and concentrated by using an Amicon ultra-0.5 10K filter.
In general, for a 250 µL reaction volume, 3 µM enzyme (10 µL from 75 µM stock in 1× PBS) was added to sodium phosphate buffer at pH 7 (190 µL, 25 mM) containing MBP-LAP (50 µM, 12.5 µL from 1 mM stock in 1× PBS), $Mg(OAc)_2$ (5 mM, 12.5 µL from 100 mM stock in reaction buffer) and C5Tz/C7Tz (250 µM, 12.5 µL from 5 mM stock in 50% $EtOH/H_2O$). After addition, ATP (5 mM, 12.5 µL from 100 mM stock in reaction buffer) was added, and the reaction mixture was incubated for 1 h at 37 ^oC with continuous shaking (300 rpm). The reaction was desalted with by using an Amicon ultra-0.5 10K filter and the MBP-LAP conjugates (C5Tz and C7Tz) were characterized by ESI-MS. The deconvoluted MS spectra (42–50 kDa range) depicting protein conjugates (C5Tz and C7Tz) are provided in Fig. 6e (panel II) of the main text and the XIC and the entire deconvoluted spectra are provided in Supplementary Fig. 37.

Step 2: Thiazolidine deprotection[20]

The C5Tz/C7Tz conjugates prepared above were subjected to thiazolidine deprotection by adding these conjugates (50 µM, 20 µL from 0.5 mM stock in water, 1 eq.) in sodium phosphate buffer at pH 7 (160 µL, 50 mM) containing $Pd[(allyl)Cl]_2$ (0.5 mM, 10 µL from 10 mM stock in MeOH, 10 eq.) and GSH (0.5 mM, 10 µL from 10 mM stock in water, 10 eq.). After the addition, the reaction was incubated at 37 ^oC with continuous shaking (150 rpm) for 1 h and then quenched by adding DTT (20 mM, 20 µL from 200 mM stock in water) at 37 ^oC. After 30 min, the reaction mixture was desalted by using an Amicon ultra-0.5 10K filter and the resultant 1,2-aminothiol-appended conjugates, C5AT and C7AT were characterized by ESI-MS. The deconvoluted MS spectra (42–50 kDa range) depicting protein conjugates (C5AT and C7AT) are provided in Fig. 6e (panel III) of the main text and the XIC and the entire deconvoluted spectra are provided in Supplementary Fig.37.

Step 3: Labelling of MBP-LAP via BHoPAL

The 1,2-aminothiol moiety of the conjugates, C5AT and C7AT, prepared above were labelled with IBH adducts by treating these conjugates (10 μM, 5 μL from 0.5 mM stock in water, 1 eq.) with IBH **1D**/**1E**/**F** (20 μM, 25 μL from 0.2 mM stock in CH_3CN, 2 eq.) in sodium phosphate buffer at pH 6.5 (220 μL, 50 mM) at 25 ^oC. After 5 h, the reaction mixtures were desalted by using an Amicon ultra-0.5 10K filter and the resultant bis-heterocyclic protein conjugates (C5-**1D**, C5-**1E**, C5-**1F**, C7-**1D**, C7-**1E**, C7-**1F**) were characterized by ESI-MS. The deconvoluted MS spectra (42–50 kDa range) depicting protein conjugates (C5-**1D**, C5-**1E**, C5-**1F**, C7-**1D**, C7-**1E**, C7-**1F**) are provided in Fig. 6e (panel IV) of the main text and the XIC and the entire deconvoluted spectra are provided in Supplementary Fig. 37.

The theoretical and the observed masses obtained for the protein conjugates obtained at each step of the workflow are provided in Supplementary Table 12.

Supplementary Table 12. Theoretical and observed molecular masses of the unmodified and modified MBP-LAP.

S.No.	Protein	Theoretical mass (Da)	Observed mass (Da)
**1**	MBP-LAP	45176.0	45175.8
**2**	C7Tz	45431.9	45431.4
**3**	C5Tz	45403.9	45403.3
**4**	C7AT	45419.9	45421.1
**5**	C5AT	45391.9	45394.3
**6**	C7-**1D**	45670.2	45670.8
**7**	C7-**1E**	45700.2	45700.6
**8**	C7-**1F**	45614.2	45616.8
**9**	C5-**1D**	45643.4	45641.4
**10**	C5-**1E**	45673.4	45671.4
**11**	C5-**1F**	45587.4	45586.1

Supplementary Fig. 37. Labelling of MBP-LAP protein. Extended ESI MS spectra of MBP-LAP protein conjugates of Fig. 6e of the main text. XICs are shown in the top panel, and their deconvoluted mass spectra are in the bottom panel of each conjugate. The observed masses of the desired protein conjugates are shown in red and their $[M+2]^{2+}$ masses in green. The corresponding theoretical masses are shown in grey within parenthesis.

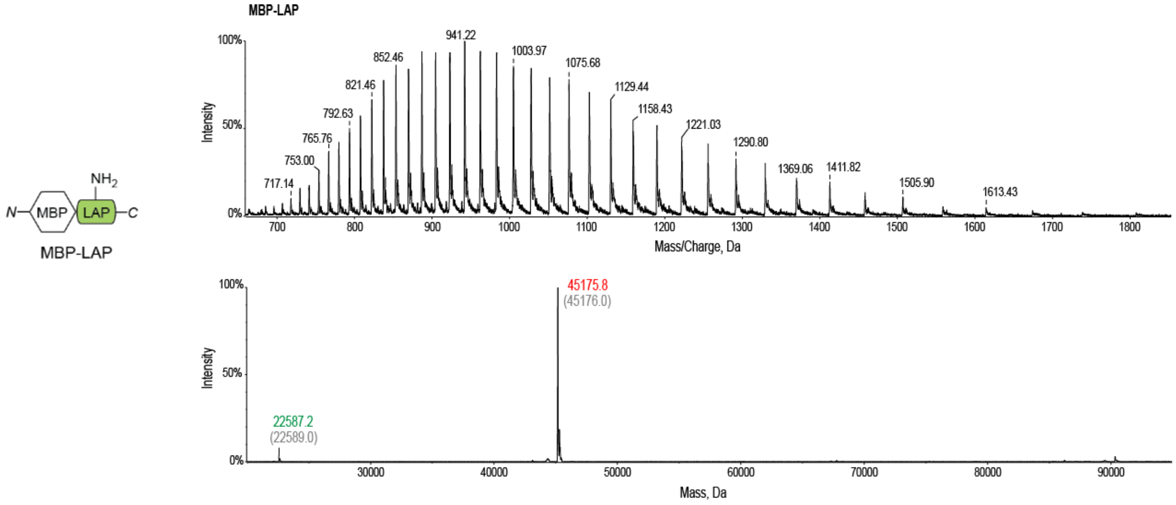

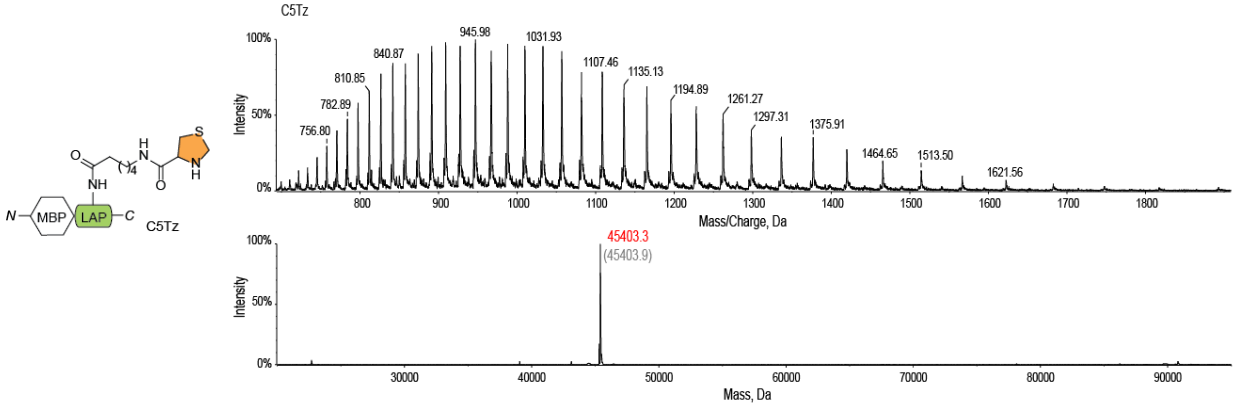

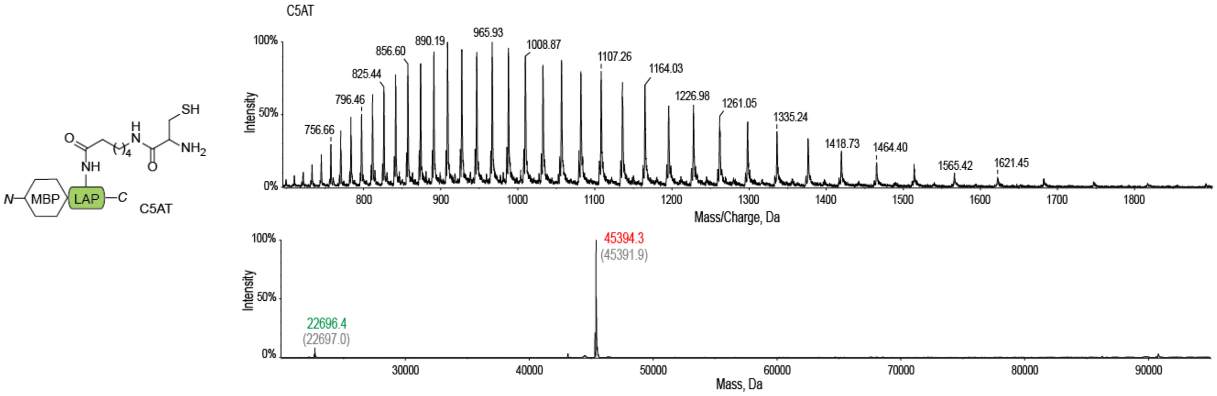

C5-**1D**

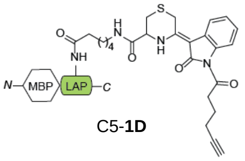

C5-**1D**

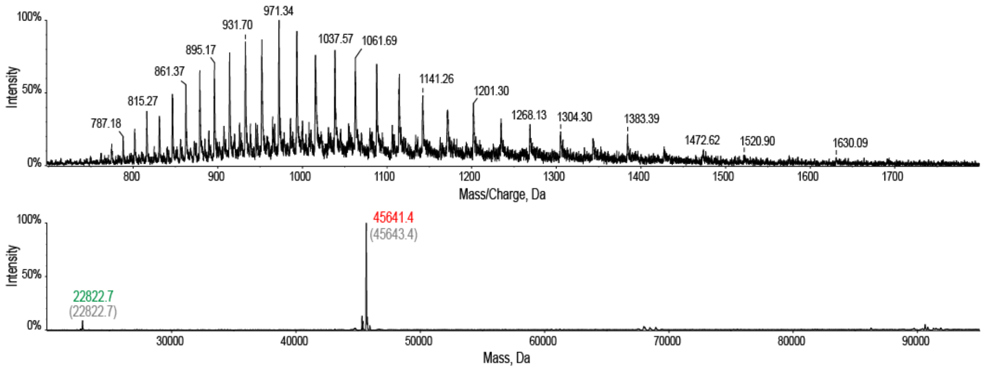

C5-**1E**

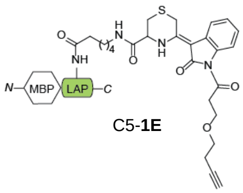

C5-**1E**

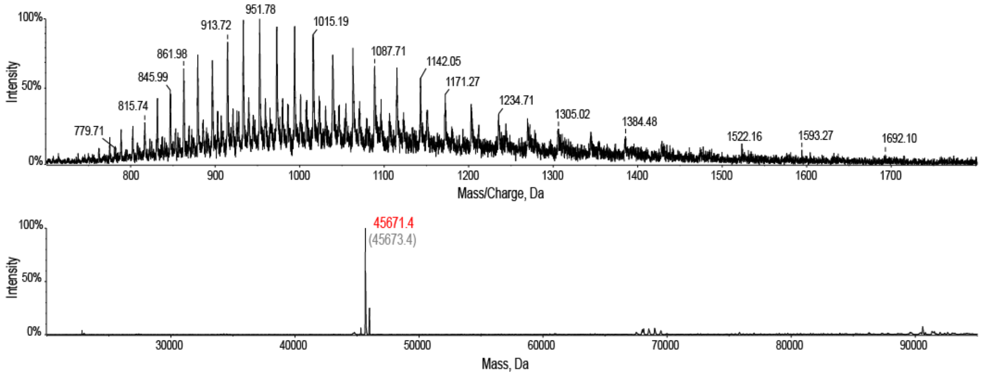

C5-**1F**

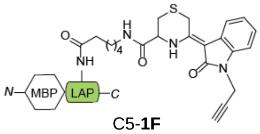

C5-**1F**

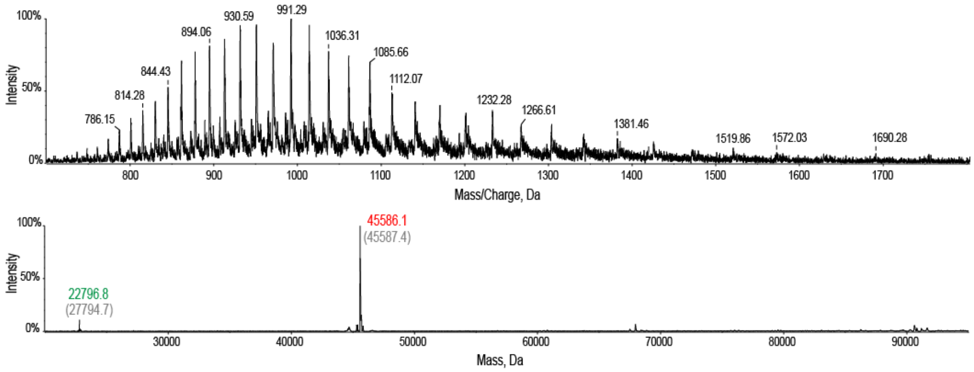

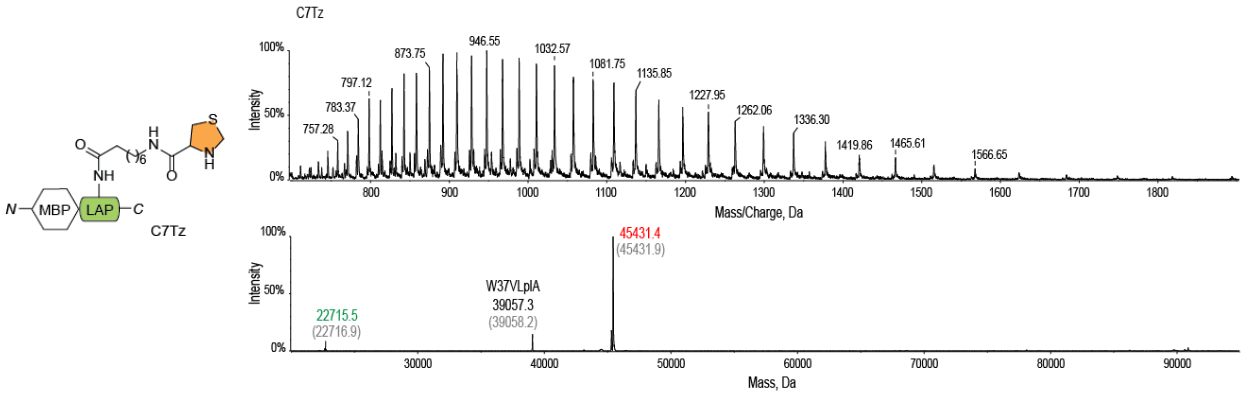
C7Tz
N MBP LAP C
C7Tz
100%
50%
0%
Intensity
757.28
783.37
797.12
873.75
946.55
1032.57
1081.75
1135.85
1227.95
1262.06
1336.30
1419.86
1465.61
1566.65
Mass/Charge, Da
45431.4
(45431.9)
W37VLplA
39057.3
(39058.2)
22715.5
(22716.9)
Mass, Da

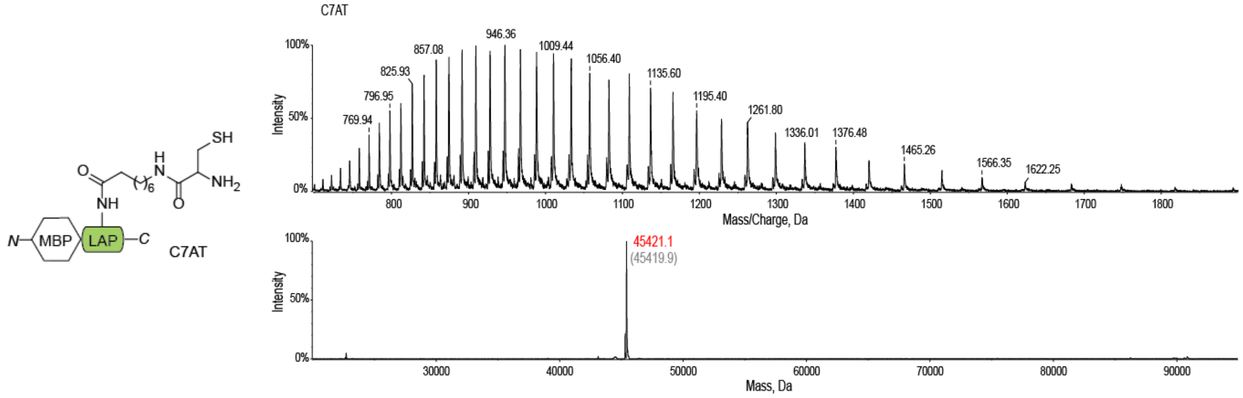
C7AT
SH
NH2
NH
N MBP LAP C
C7AT
100%
50%
0%
Intensity
769.94
796.95
825.93
857.08
946.36
1009.44
1056.40
1135.60
1195.40
1261.80
1336.01
1376.48
1465.26
1566.35
1622.25
Mass/Charge, Da
45421.1
(45419.9)
Mass, Da

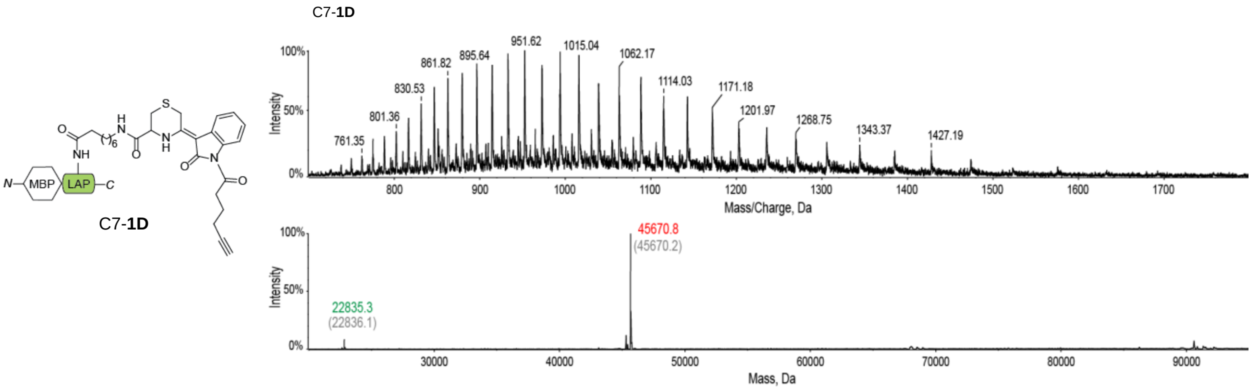
C7-1D
N MBP LAP C
C7-1D
100%
50%
0%
Intensity
761.35
801.36
830.53
861.82
895.64
951.62
1015.04
1062.17
1114.03
1171.18
1201.97
1268.75
1343.37
1427.19
Mass/Charge, Da
45670.8
(45670.2)
22835.3
(22836.1)
Mass, Da

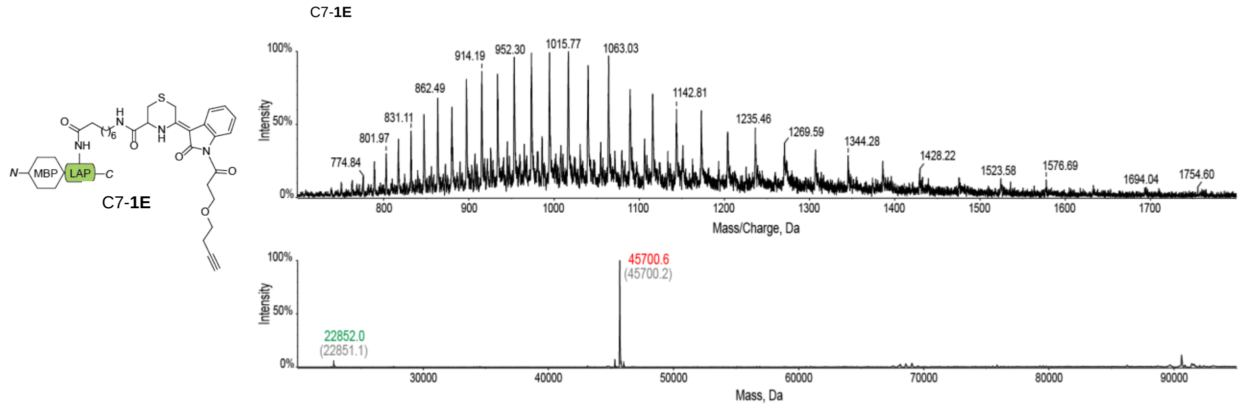

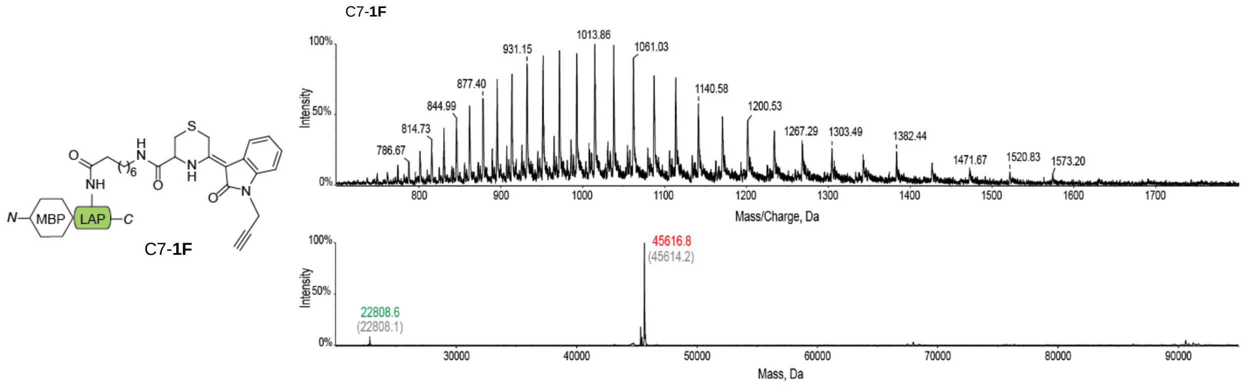

Procedure for the enzymatic digestion of MBP-LAP conjugate (C7-**1F**) for the MS/MS analysis

The C7-**1F** conjugate was digested following procedure as described for *N*-Cys-eGFP-**1D** conjugate in section 7 above, by employing chymotrypsin in place of trypsin. The sequence of labelled MBP-LAP conjugate, C7-**1F**, is given below with their chymotrypsin cleavage sites shown as red vertical bars. The LAP sequence is underlined and the labelled lysine (K) of the LAP is highlighted in yellow circle with a modification shown as pink star. The MS/MS analysis of the conjugate is depicted in Supplementary Fig 38.

MKSSHHHHHHGSSMKIEEGKLVIW|INGDKGY|NGLAEVGKKF|EKDTGIKVTVEHPDKLEEKF|PQVAATGDGPDIIF|W|AHDRF

|GGY|AQSGLLAEITPDKAF|QDKLY|PF|TW|DAVRY|NGKLIAY|PIAVEALSLIY|NKDLLPNPPKTW|EEIPALDKELKAKGKSAL

MF|NLQEPY|F|TW|PLIAADGGY|AF|KY|ENGKY|DIKDVGVDNAGAKAGLTF|LVDLIKNKHMNADTDY|SIAEAAF|NKGETA

MTINGPW|AW|SNIDTSKVNY|GVTVLPTF|KGQPSKPF|VGVLSAGINAASPNKELAKEF|LENY|LLTDEGLEAVNKDKPLGAVAL

KSY|EEELAKDPRIAATMENAQKGEIMPNIPQMSAF|W|Y|AVRTAVINAASGRQTVDEALKDAQTNGIEENLY|F|QSNAGGGGS

GF|EIDKVW|Y|DLDA

Supplementary Fig. 38. LC-MS/MS analyses of chymotrypsin-digested MBP-LAP C7-1F conjugate. The intact MS of the labelled fragment of the conjugates is shown on left and their MS/MS spectra on right. The observed labelled peptide mass is shown in black and the corresponding theoretical mass in grey within parenthesis. The sequence of the labelled protein with the chymotrypsin cleavage sites and the site of modification is given above.

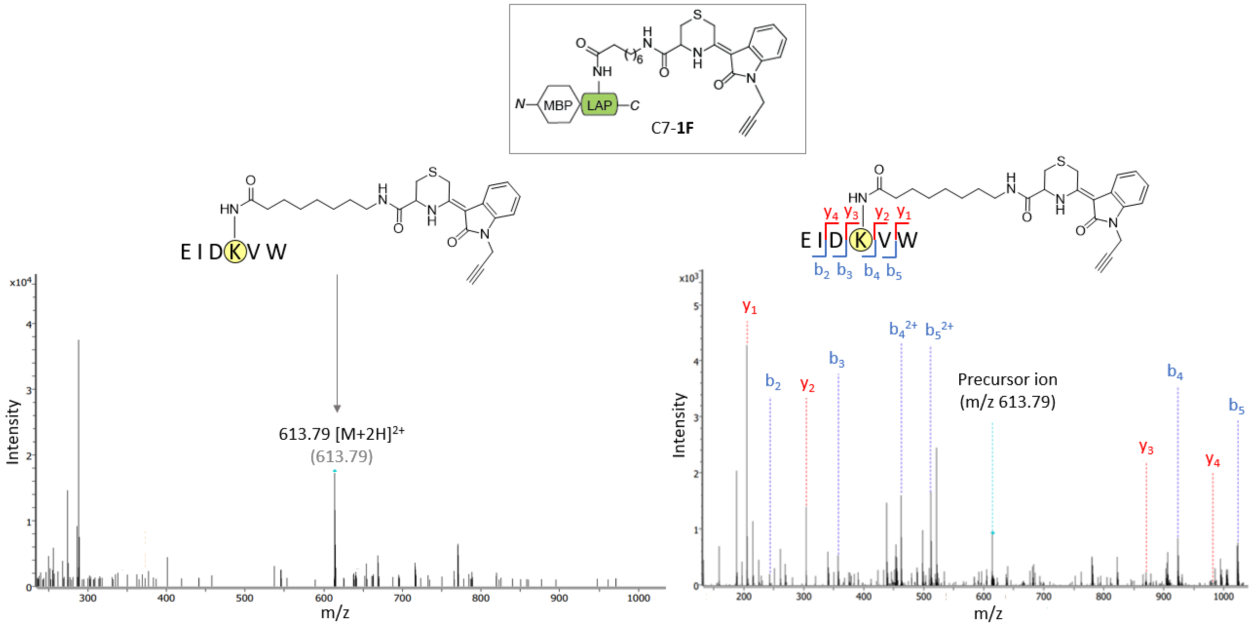

Supplementary Fig. 39. Isolated yields of a three-step protocol involved in generating C7-1D on a milligram scale. Two independent sets of experiments (Exp. 1 and 2) were performed on a milligram scale of MBP-LAP protein to generate C7-**1D** following a three-step procedure as described above in section 14. The protein conjugates at each step were desalted by using an Amicon ultra-0.5 10K filter and the isolated percentage yields obtained at each step are given in the parentheses.

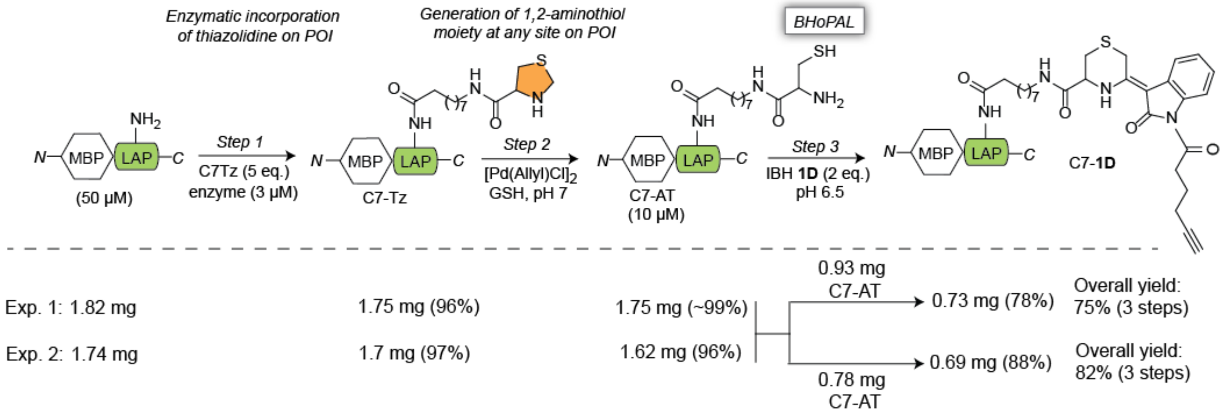

15. Recombinant production of His-TEV(C)-MBP-LAP protein and its cleavage with TEV protease

Plasmid construction (cloning): Plasmids were constructed via site-directed mutagenesis by employing the insertion method. The primers employed are summarized in Supplementary Table 13.

Supplementary Table 13. Sequence of primers employed for cloning to generate His-TEV(C)-MBP-LAP construct.

Primer name	Sequence (5'-3')
BT-MBPLAP1F	GCAGGTGGTGGCGGTGGGATCGAGGAAAACCTGTACTTCC
BT-MBPLAP1R	ACCGCCACCACCTGCATTAGTCTGCGCGTCTTTCAGGGCTTC
BT-MBPLAP2F	AGCGGTTTCGAAATTGATAAAGTTGGGATCGAGGAAAACCTGTACTTCC
BT-MBPLAP2R	AACTTTATCAATTTCGAAACCGCTACCGCCACCACCTGCATTAGTCTG
BT-MBPLAP3F	TGGTATGACCTGGATGCATAAGGGATCGAGGAAAACCTGTACTTCC
MBPLAP3-R	TTATGCATCCAGGTCATACCAAACTTTATCAATTTCGAAACCGCTACCGCC

His-TEV(C)-MBP-LAP construct:

A LAP tag with a flanking linker (AGGGGS-GFEIDKVWYDLDA) was inserted before TEV site at the *C*-terminus of 6×His-TEV-MBP-TEV construct (mutant 1 in the upper panel of Supplementary Fig. 17c) to generate His-TEV(C)-MBP-LAP construct by site-directed mutagenesis in 3 PCR reactions following similar protocol as described above section 6. The primers employed in the cloning are given in Supplementary Table 13. The product of each PCR reaction served as a template for the next PCR reaction, and all the insertions were confirmed by sequencing data (Supplementary Fig. 40).

PCR 1: Primers (forward BT-MBPLAP1F and reverse BT-MBPLAP1R) and construct generated (6×His-TEV-MBP-AGGGG)

PCR 2: Primers (forward BT-MBPLAP2F and reverse BT-MBPLAP2R) and construct generated (6×His-TEV-MBP-AGGGGS-GFEIDKV)

PCR 3: Primers (forward BT-MBPLAP3F and reverse BT-MBPLAP3R) and construct generated (6×His-TEV-MBP-AGGGGS-GFEIDKVWYDLDA*)
*Denotes stop codon.

Supplementary Fig. 40. Sequence analysis of LAP-tag inserted TEV-MBP construct generated via three PCR steps.

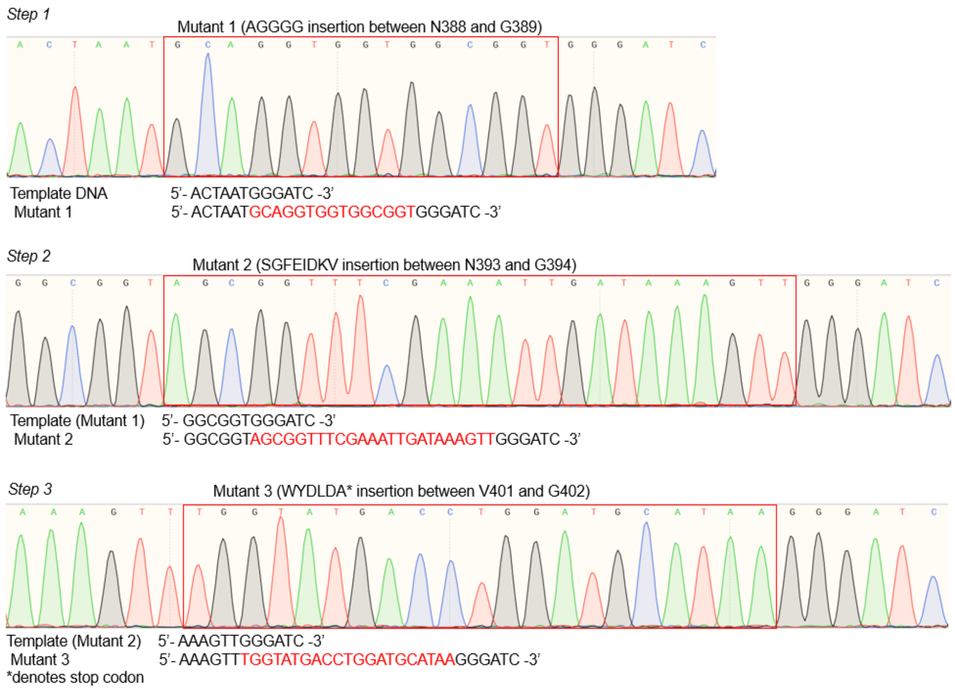

Protein expression, purification and storage:

The plasmid encoding His-TEV(C)-MBP-LAP was transformed into competent *E. coli* cells, BL21(DE3). A single colony of transformed cells was inoculated in LB media supplemented with antibiotic ampicillin (100 µg/mL). The cells were cultured at 37 ºC with continuous shaking at 220 rpm. After 12 h, the cell culture was diluted with LB media (1:100, 500 mL) and the cells were grown further under the same conditions to an optical density (OD600) of 0.5–0.6. Next, the protein expression was induced by adding IPTG (1 mM) inducer. The cells were cultured at 25 °C, 220 rpm for 8–10 h and harvested by centrifugation (5000 g for 20 min) at 4 °C. Cell pellet from a 500 mL culture was resuspended in lysis buffer (20 mL) at pH 7.5 containing 50 mM Tris-Cl, 250 mM NaCl, 10 mM imidazole, 1mM DTT and 1 mM PMSF. The cells were lysed by sonication for 5 min at 4 °C (20 s on, 59 s off, 40% amplitude). The cell debris were removed by centrifugation (20,000 g for 1 h) at 4 °C. The supernatant was separated and mixed with Ni NTA agarose resin at 4 °C for 1 h to facilitate the binding of His-tagged protein. The resin was then loaded on a poly-prep chromatography column (Cat. no. 731-1550) and washed with 10 column volumes of wash buffer at pH 7.5 containing 50 mM Tris-Cl, 250 mM NaCl, 20 mM imidazole and 1mM DTT. His-tagged protein bound to the resin was eluted with buffer at pH 7.5 containing imidazole (30

→ 250 mM), 50 mM Tris-Cl, 250 mM NaCl and 1mM DTT, and the collected fractions were analyzed by SDS-PAGE for purity (Supplementary Fig. 41, 1st Lane). The pure protein fractions were pooled and dialyzed (membrane cut off 12–14 kDa) overnight at 4 °C against storage buffer (50 mM Tris-HCl pH 8, 250 mM NaCl and 1 mM DTT). The concentration of the dialyzed protein was measured at A280 using nanodrop. The His-tagged TEV-MBP-LAP was stored at -80 ºC after snap-freezing at a concentration of 2 mg/mL. The sequence of the His-TEV(C)-MBP-LAP is provided in Supplementary Table 14.

TEV cleavage reaction to produce *N*-Cys-MBP-LAP protein

The TEV protease cleavage of His-TEV(C)-MBP-LAP was performed under the dialysis conditions following similar procedure as described for the TEV cleavage of His-tagged proteins to generate *N*-Cys-POI in section 5 above. The dialyzed protein solution was mixed with Ni-NTA resin and loaded on a poly prep column to elute TEV-cleaved protein in the flowthrough. The purity of collected protein fractions was analyzed by SDS-PAGE (Supplementary Fig. 41, 4th lane from left). Aliquots of pure protein fractions (~1 mg/mL) were snap-frozen in liquid N_2 and stored at -80 ºC until their use for protein bioconjugation. The sequence of *N*-Cys-MBP-LAP is provided in Supplementary Table 14 and the cleaved protein was characterized by ESI-MS (Supplementary Fig. 42). The theoretical and observed protein masses are provided in Supplementary Table 15.

Supplementary Fig. 41. SDS-PAGE analysis of purified His-TEV(C)-MBP-LAP and TEV-cleaved protein. The numbers on the gel images denote the protein marker size in kDa.

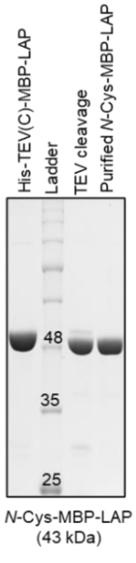

Supplementary Table 14. Sequence of recombinant His-TEV(C)-MBP-LAP and TEV cleaved *N*-Cys-MBP-LAP proteins. The mutations are underlined and the vertical line in the TEV site (ENLYFQ❙C) denotes the TEV protease cleavage site.

**Protein**	**Sequence**
**His-TEV(C) - MBP-LAP**	MKSSHHHHHHGENLYFQ❙CSSMKIEEGKLVIWINGDKGYNGLAEVGKKFEKDTGIKVTVEHPDKLEEKF<br>PQVAATGDGPDIIFWAHDRFGGYAQSGLLAEITPDKAFQDKLYPFTWDAVRYNGKLIAYPIAVEALSLIY<br>NKDLLPNPPKTWEEIPALDKELKAKGKSALMFNLQEPYFTWPLIAADGGYAFKYENGKYDIKDVGVDNA<br>GAKAGLTFLVDLIKNKHMNADTDYSIAEAAFNKGETAMTINGPWAWSNIDTSKVNYGVTVLPTFKGQP<br>SKPFVGVLSAGINAASPNKELAKEFLENYLLTDEGLEAVNKDKPLGAVALKSYEEELAKDPRIAATMENAQ<br>KGEIMPNIPQMSAFWYAVRTAVINAASGRQTVDEALKDAQTN<u>AGGGGSGFEIDKVWYDLDA</u>
***N*-Cys-MBP-LAP**	CSSMKIEEGKLVIWINGDKGYNGLAEVGKKFEKDTGIKVTVEHPDKLEEKFPQVAATGDGPDIIFWAH<br>DRFGGYAQSGLLAEITPDKAFQDKLYPFTWDAVRYNGKLIAYPIAVEALSLIYNKDLLPNPPKTWEEIPA<br>LDKELKAKGKSALMFNLQEPYFTWPLIAADGGYAFKYENGKYDIKDVGVDNAGAKAGLTFLVDLIKNK<br>HMNADTDYSIAEAAFNKGETAMTINGPWAWSNIDTSKVNYGVTVLPTFKGQPSKPFVGVLSAGINA<br>ASPNKELAKEFLENYLLTDEGLEAVNKDKPLGAVALKSYEEELAKDPRIAATMENAQKGEIMPNIPQMS<br>AFWYAVRTAVINAASGRQTVDEALKDAQTN<u>AGGGGSGFEIDKVWYDLDA</u>

16. Concomitant and tandem BHoPAL for dual labelling of proteins

One-step dual modification (Concomitant BHoPAL):

Step 1: Generation of *N*-Cys-MBP-LAP via TEV protease cleavage reaction

The procedure for the generation of *N*-Cys-MBP-LAP from His-TEV(C)-MBP-LAP employing TEV protease is described above.

Step 2: Ligation of C7Tz on the LAP tag of *N*-Cys-MBP-LAP

The ligation of C7Tz to the LAP tag of *N*-Cys-MBP-LAP was performed following similar procedure as described for the ligation of C5Tz/C7Tz to the MBP-LAP employing 3 μM enzyme in section 14 (Step 1). The desired ligated conjugate, *N*-Cys-C7Tz, was obtained in quantitative yields and was characterized by ESI-MS. The deconvoluted MS spectra (41–45 kDa range) depicting protein conjugate mass is provided in Fig. 7a (bottom panel) of the main text and the XIC and the entire deconvoluted spectra are provided in Supplementary Fig. 42.

Step 3: Thiazolidine deprotection

The thiazolidine moiety of the *N*-Cys-C7Tz was deprotected with Pd[(allyl)Cl]$_2$/GSH to generate *N*-Cys-C7AT by following similar procedure as described for the conversion of C5Tz/C7Tz to C5AT/ C7AT in section 14 (Step 2). Quantitative conversions were obtained in the reaction and the resultant *N*-Cys-C7AT conjugate appended with two 1,2-aminothiol moieties was characterized by ESI-MS. The deconvoluted MS spectra (41–45 kDa range) depicting *N*-Cys-C7AT is provided in Fig. 7a (bottom panel) of the main text and the XIC and the entire deconvoluted spectra are provided in Supplementary Fig. 42.

Step 4: Dual labelling of *N*-Cys-C7AT

The two 1,2-aminothiol moieties of the conjugate, *N*-Cys-C7AT, prepared above were labelled with IBH adduct by treating this conjugate (10 µM, 5 µL from 0.5 mM stock in water, 1 eq.) with IBH **1E** (50 µM, 25 µL from 0.5 mM stock in CH_3CN, 5 eq.) in sodium phosphate buffer at pH 6.5 (220 µL, 50 mM) at 25 ^oC. After 6 h, the reaction mixture was desalted by using an Amicon ultra-0.5 10K filter to yield the desired dually functionalized protein conjugate, **1E**-C7-**1E** in 93% yield as depicted by its ESI-MS. The deconvoluted MS spectra (42– 46 kDa range) depicting protein conjugate **1E**-C7-**1E** is provided in Fig. 7a (bottom panel) of the main text and the XIC and the entire deconvoluted spectra are provided in Supplementary Fig. 42.

Step-wise dual modification (Tandem BHoPAL):

Step 1: *N*-Cys modification of *N*-Cys-MBP-LAP via BHoPAL (Introduction of first label)

The bioconjugation reactions of *N*-Cys-MBP-LAP with IBH **1D** was set up by following a similar procedure as described in section 7. A solution of *N*-Cys-MBP-LAP (5 µL, 0.5 mM stock in water, final conc. 10 µM, 1 eq.) sodium phosphate buffer at pH 6.5 (220 µL, 50 mM) was mixed with a solution of IBH adduct **1D** (25 µL, 0.12 mM stock in CH_3CN, final conc. 12 µM, 1.2 eq.) at RT. After 1 h, the reaction mixture was desalted using an Amicon ultra-0.5 10K to yield the desired labelled protein conjugate, **1D**-MBP-LAP, in >99% yield as depicted by its ESI-MS. The deconvoluted MS spectra (41– 45 kDa range) depicting **1D**-MBP-LAP is provided in Fig. 7b of the main text and the XIC and the entire deconvoluted spectra are provided in Supplementary Fig. 42.

Step 2: Ligation of C7Tz on the LAP tag of **1D**-MBP-LAP

The ligation of C7Tz to the LAP tag of **1D**-MBP-LAP was performed following similar procedure as described for the ligation of C5Tz/C7Tz to the MBP-LAP employing 3 μM enzyme in section 14 (Step 1). The desired ligated conjugate, **1D**-C7Tz, was obtained in quantitative yields (>99%) and was characterized by ESI-MS. The deconvoluted MS spectra (41–45 kDa range) depicting **1D**-C7Tz mass is provided in Fig. 7b of the main text and the XIC and the entire deconvoluted spectra are provided in Supplementary Fig. 42.

Step 3: Thiazolidine deprotection

The thiazolidine moiety of the **1D**-C7Tz was deprotected with Pd[(allyl)Cl]$_2$/GSH to generate **ID**-C7AT by following similar procedure as described for the conversion of C5Tz/C7Tz to C5AT/ C7AT in section 14 (Step 2). Quantitative yields (>99%) were obtained in the reaction and the resultant **1D**-C7AT conjugate was characterized by ESI-MS. The deconvoluted MS spectra (41–45 kDa range) depicting **1D**-C7AT is provided in Fig. 7b of the main text and the XIC and the entire deconvoluted spectra are provided in Supplementary Fig. 42.

Step 4: Dual modification of protein via BHoPAL (introduction of second label)

The 1,2-aminothiol moiety of the **1D**-C7AT conjugate as prepared above was labelled with IBH adduct **1E** by treating this conjugate (10 μM, 5 μL from 0.5 mM stock in water, 1 eq.) with IBH **1E** (50 μM, 25 μL from 0.5 mM stock in CH_3CN, 5 eq.) in sodium phosphate buffer at pH 6.5 (220 μL, 50 mM) at 25 °C. After 6 h, the reaction mixture was desalted by using an Amicon ultra-0.5 10K filter and the resultant dually functionalized protein conjugate, **1D**-C7-**1E**) was characterized by ESI-MS. The deconvoluted MS spectra (42– 46 kDa range) depicting the mass of **1D**-C7-**1E** is provided in Fig. 7b of the main text and the XIC and the entire deconvoluted spectra are provided in Supplementary Fig. 42.

Note: The theoretical and observed masses of the protein conjugates obtained at each step are provided in Supplementary Table 15.

Supplementary Table 15. Theoretical and observed molecular masses of the TEV-MBP-LAP and *N*-Cys-MBP-LAP conjugates.

S.No.	Protein	Theoretical mass (Da)	Observed mass (Da)
**1**	TEV-MBP-LAP	44778.6	44776.7
**2**	*N*-Cys-MBP-LAP	42670.4	42668.0
**3**	*N*-Cys-C7Tz	42926.5	42925.9
**4**	*N*-Cys-C7AT	42914.5	42916.5
**5**	**1E**-C7-**1E**	43472.7	43473.1
**6**	**1D**-MBP-LAP	42919.5	42917.2
**7**	**1D**-C7Tz	43175.6	43171.8
**8**	**1D**-C7AT	43163.6	43163.5
**9**	**1D**-C7-**1E**	43442.7	43443.7

Supplementary Fig. 42. Concomitant and Tandem dual labelling of protein via BHoPAL. Extended ESI MS spectra protein conjugates of Fig. 7 of the main text. XICs are shown in the top panel, and their deconvoluted mass spectra are in the bottom panel of each conjugate. The observed masses of the desired protein conjugates are shown in red and their $[M+2]^{2+}$ masses in green. The corresponding theoretical masses are shown in grey within parenthesis.

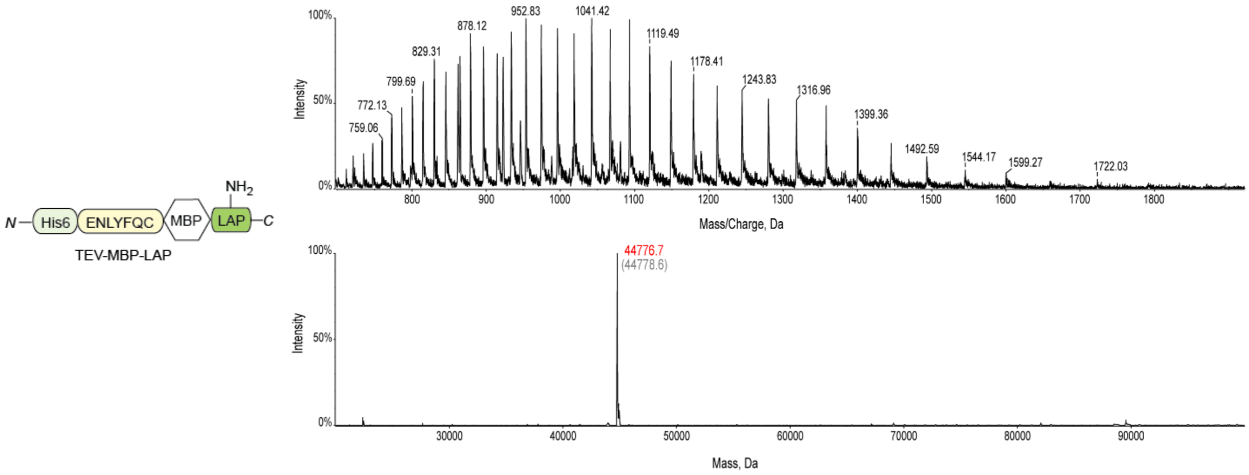

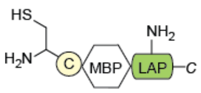

N-Cys-MBP-LAP

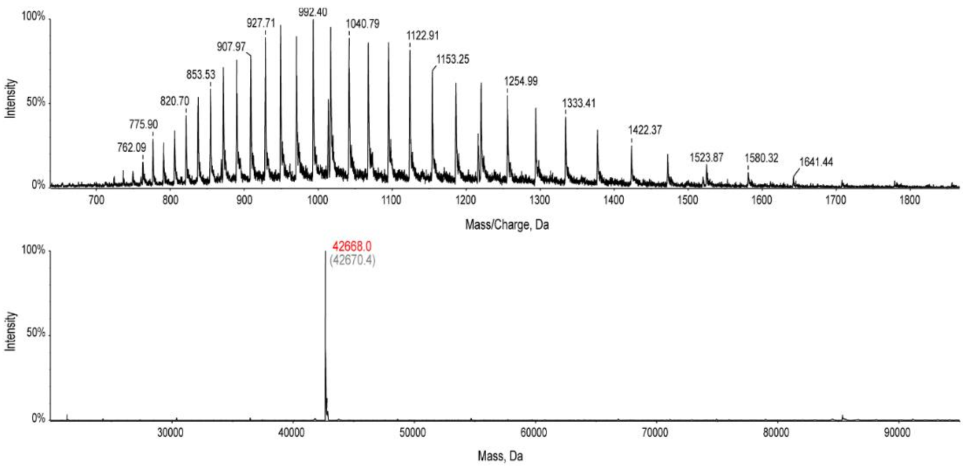

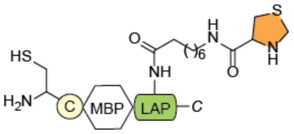

N-Cys-C7Tz

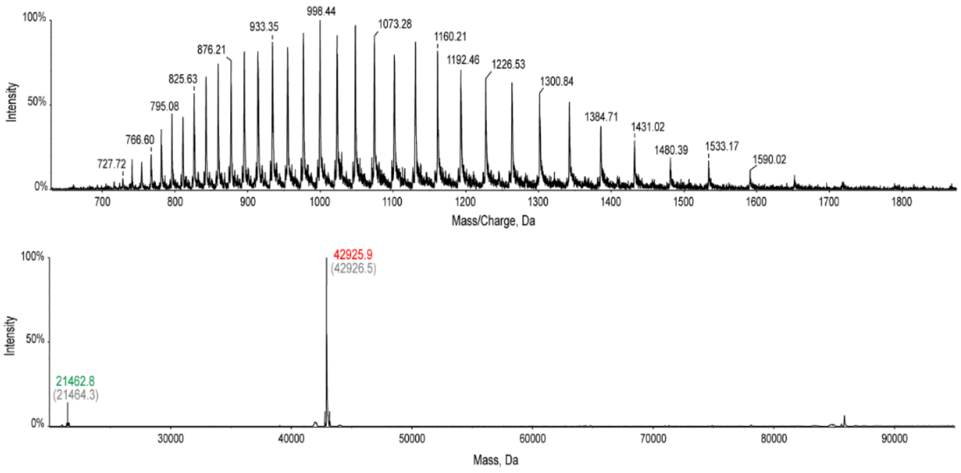

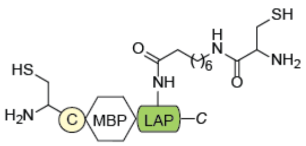

N-Cys-C7AT

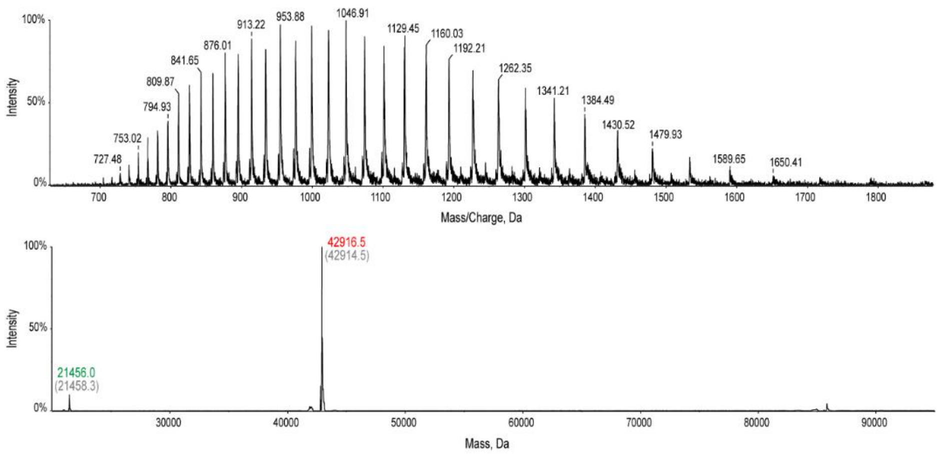

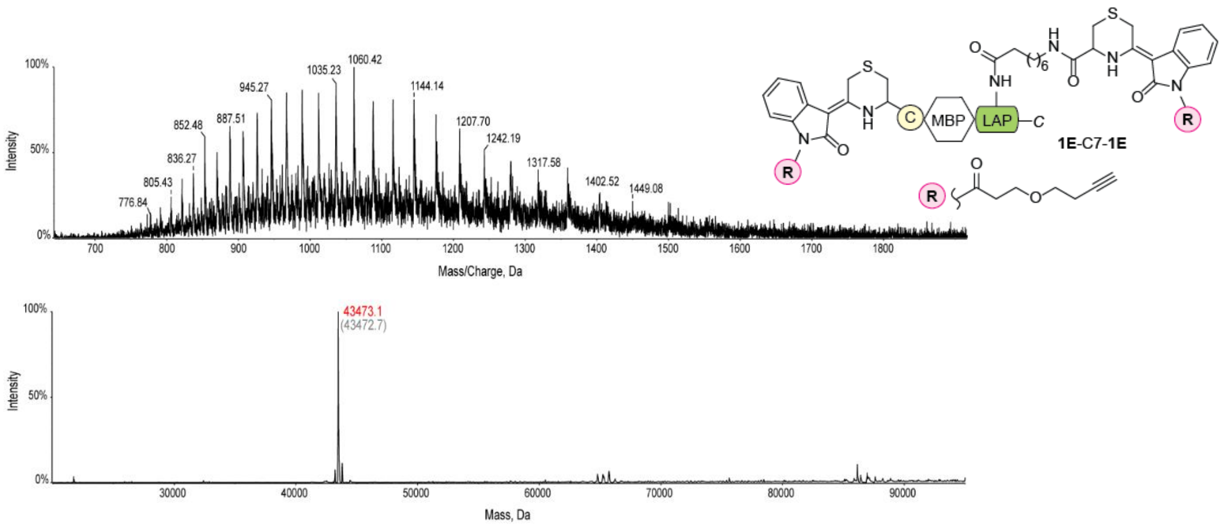
100%
50%
0%
Intensity
776.84
805.43
836.27
852.48
887.51
945.27
1035.23
1060.42
1144.14
1207.70
1242.19
1317.58
1402.52
1449.08
Mass/Charge, Da
43473.1
(43472.7)
Mass, Da
MBP
LAP
1E-C7-1E
R

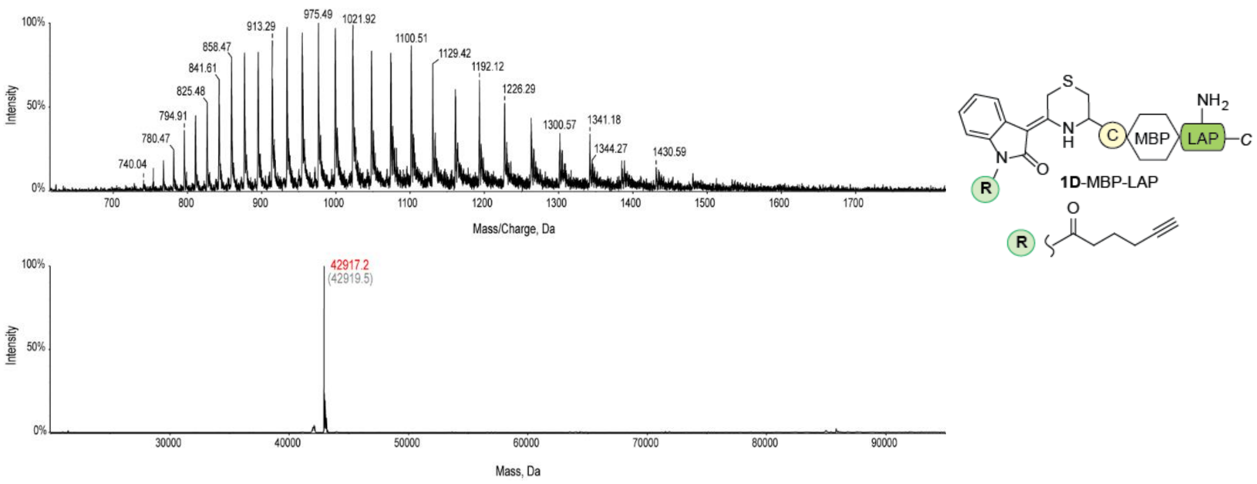
100%
50%
0%
Intensity
740.04
780.47
794.91
825.48
841.61
858.47
913.29
975.49
1021.92
1100.51
1129.42
1192.12
1226.29
1300.57
1341.18
1344.27
1430.59
Mass/Charge, Da
42917.2
(42919.5)
Mass, Da
MBP
LAP
1D-MBP-LAP
R

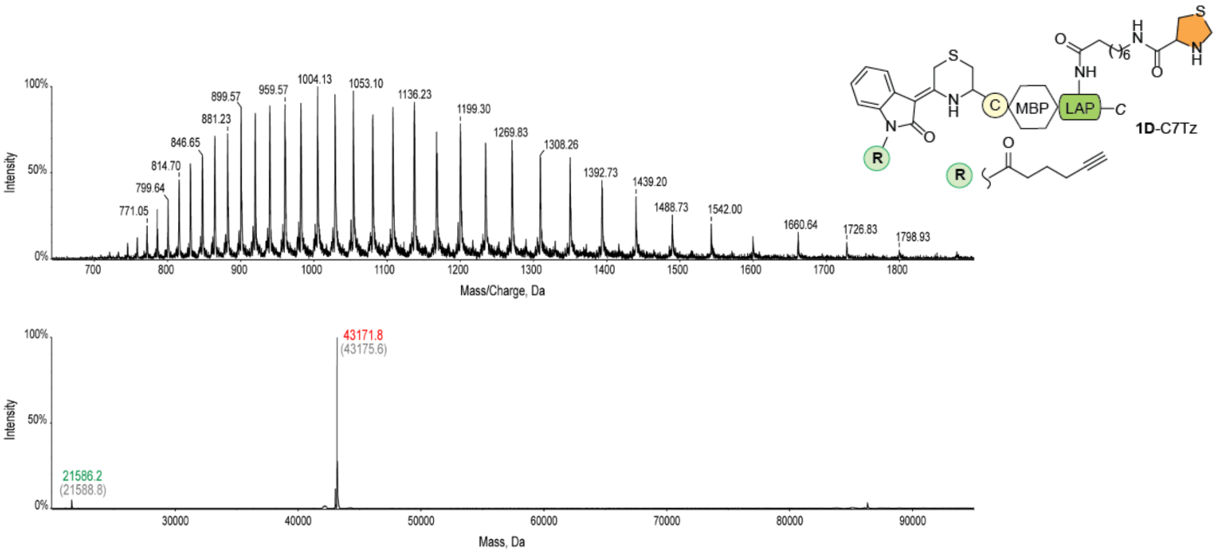
100%
50%
0%
Intensity
771.05
799.64
814.70
846.65
881.23
899.57
959.57
1004.13
1053.10
1136.23
1199.30
1269.83
1308.26
1392.73
1439.20
1488.73
1542.00
1660.64
1726.83
1798.93
Mass/Charge, Da
43171.8
(43175.6)
21586.2
(21588.8)
Mass, Da
MBP
LAP
1D-C7Tz
R

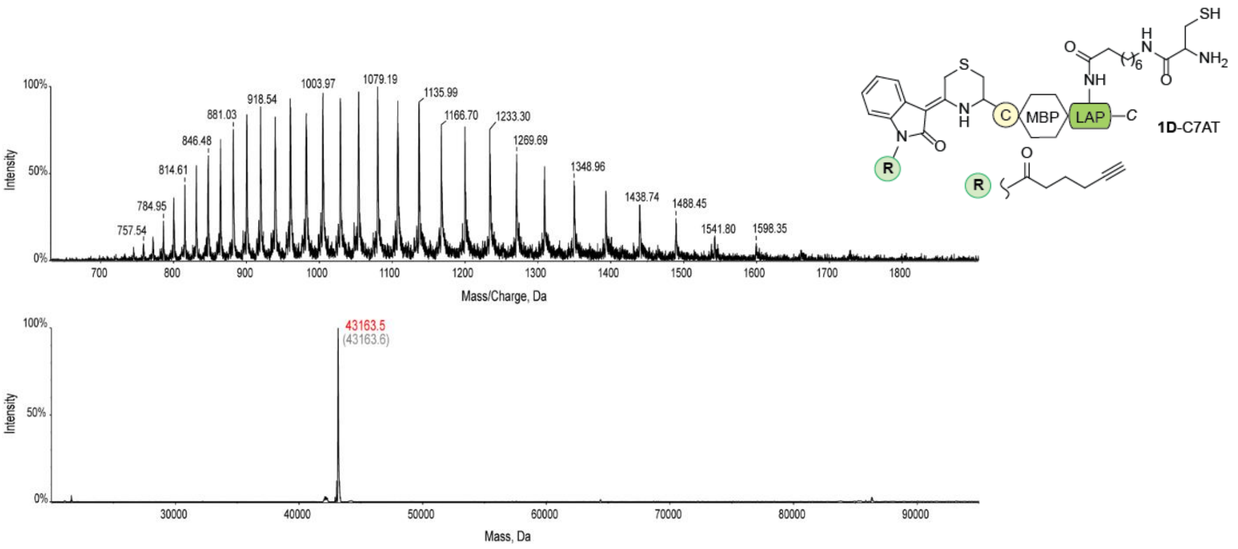

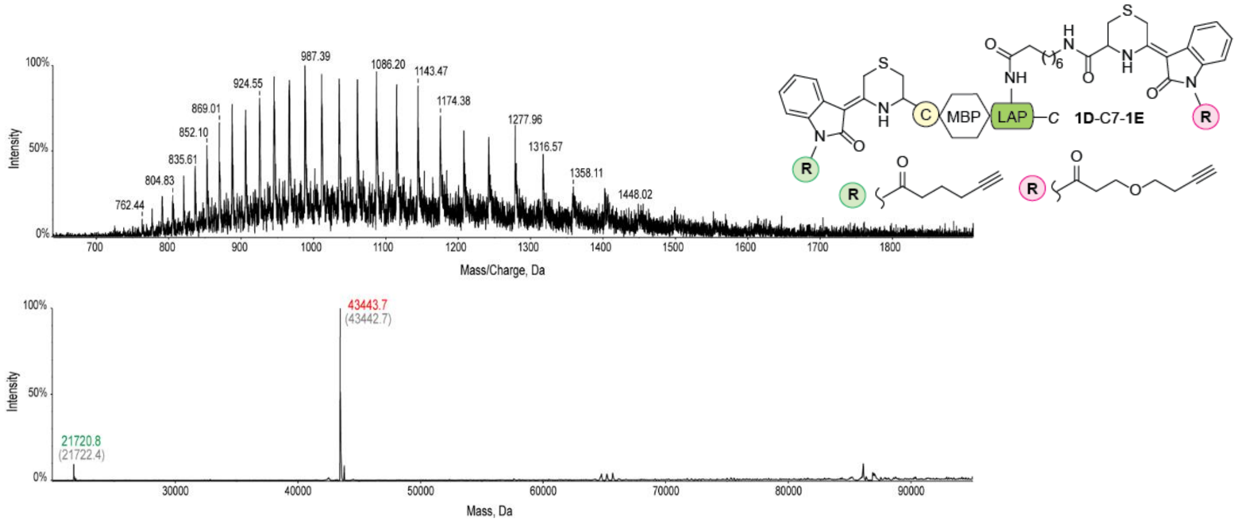

<u>Conditions screened for the one-step mono modification of *N*-Cys-C7AT</u>

The bioconjugation reactions of *N*-Cys-C7AT with IBH **1E** were set up by following a similar procedure as described in section 7. A solution of *N*-Cys-C7AT (5 µL, 0.5 mM stock in water, final conc. 10 µM, 1 eq.) sodium phosphate buffer at pH 6.5 (220 µL, 50 mM) was mixed with a solution of IBH adduct **1E** (25 µL, 0.1–0.15 mM stock in CH_3CN, final conc. 10–15 µM, 1–1.5 eq.) at different temperatures (4–25 °C). The reaction mixtures were desalted using an Amicon ultra-0.5 10K at different time intervals (5–60 min) and the resultant desalted protein conjugates were analyzed by ESI MS. The percentage conversions observed under different reaction conditions are summarized in Supplementary Table 16 and the deconvoluted MS spectra acquired under those conditions are provided below this table.

Supplementary Table 16. Reaction conditions screened for one-step mono modification of *N*-Cys-C7AT with IBH **1E.** The reactions were conducted by incubating 10 μM *N*-Cys-C7AT (1 eq.) with IBH 1E (1–1.5 eq.) in sodium phosphate buffer (50 mM, pH 6.5) in different conditions**. A (%)**, **B (%)** and **C (%)** represent percentage of unmodified *N*-Cys-C7AT, mono modified protein and dually modified protein conjugate, respectively. No preferential labelling of one of the two 1,2-aminothiols in quantitative conversions were found in these reactions. All the deconvoluted ESI-MS spectra are provided below the table. The corresponding theoretical masses are shown in grey within parenthesis.

Entry	IBH 1E (eq.)	T (°C)	Time (min)	*N*-Cys-C7AT A (%) | B (%) | C (%)
1	1	25	20	44 | 56 | 0
2	1.1	25	20	20 | 62 | 18
3	1.2	25	20	0 | 44 | 56
4	1	10	45	45 | 55 | 0
5	1.5	10	5	0 | 56 | 44
6	1.5	10	10	0 | 53 | 47
7	1	4	60	53 | 47 | 0
8	1.1	4	60	10 | 64 | 26
9	1.5	4	60	0 | 45 | 55

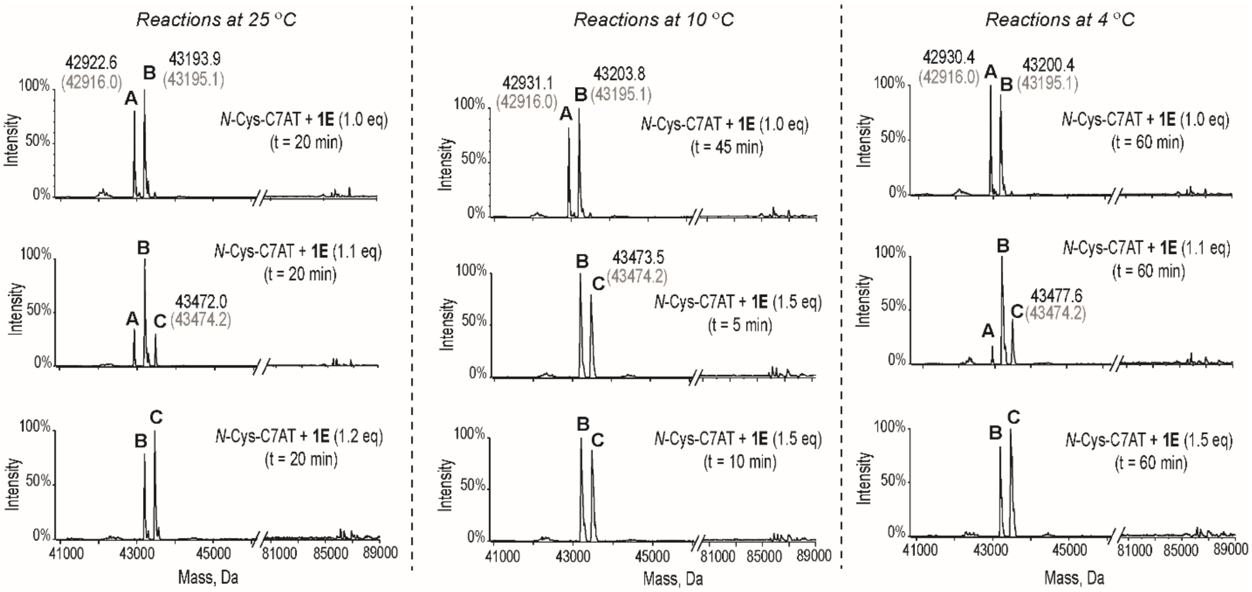

17. Binding assays of mono and dually labelled MBP conjugates

In a 1.5 mL microcentrifuge tube, 0.5 mL of amylose resin (NEB# E8021S) was equilibrated with 6 column volume (CV) of binding buffer at pH 7.5 (20 mM Tris-HCl, 0.2 M NaCl and 1 mM EDTA) by resuspending the resin into buffer (3 × 1 mL each) followed by centrifugation

(1100 g, 4 °C, 3 min). The supernatant was discarded and to the equilibrated resin was added 0.2 mg of MBP protein (unmodified MBP-LAP, mono-labelled C7-**1D** and dually-labelled **1D**-C7-**1E**) in 850 μL of binding buffer. The mixture was kept at 4 °C for 3 h with continuous mixing to facilitate protein binding. The resin was then loaded onto the poly-prep chromatography column (Cat. no. 731-1550) and the flow through was collected by gravity flow. The resin was then washed with 1.5 CVs of binding buffer to remove the unbound protein. Next, the bound protein to the resin was eluted with binding buffer containing 10 mM maltose and collected in aliquots of 150 μL until the A280 of the eluted solution was ≥ 0.05. Finally, 10 μL of each collected fractions (flowthrough, wash and eluate) were mixed with 10 μL of 2× gel loading buffer, heated at 95 °C for 5 min and analysed by SDS-PAGE (20% Tris-Glycine gel, 120 V) (Supplementary Fig. 43). The concentration of protein in each of the collected fractions was measuring the absorbance at 280 nm. The % recovery was calculated using the following formula:

$$\% \boldsymbol{Recovery} = \frac{\boldsymbol{Total\ protein\ in\ eluate}\ (\mu \boldsymbol{g})}{\boldsymbol{Total\ protein}\ (\mu \boldsymbol{g})} \times \mathbf{100}$$

Supplementary Fig. 43. Binding studies on MBP-LAP conjugates. Top: Schematic illustration of amylose resin binding experiment with MBP conjugates. Bottom: SDS-PAGE analysis of eluted protein conjugates (MBP-LAP, C7-**1D** and **1D**-C7-**1E**, fractions in amylose binding assay and their observed % recovery.

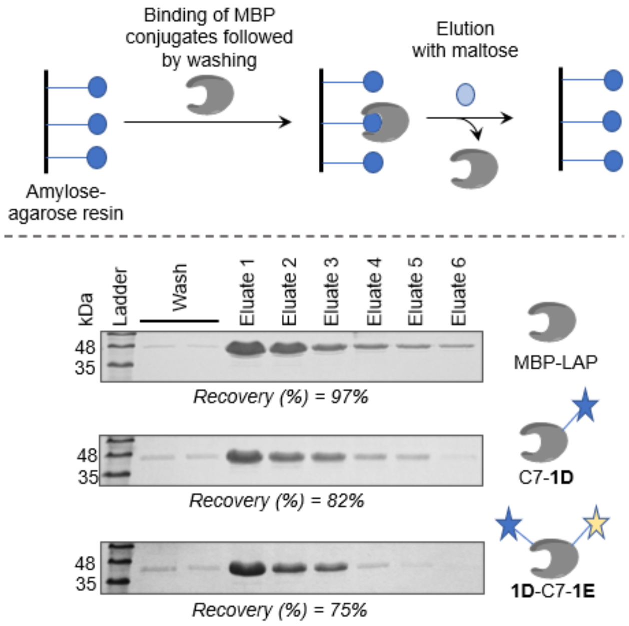

18. NMR spectral data

Supplementary Fig. 44. NMR of S1A

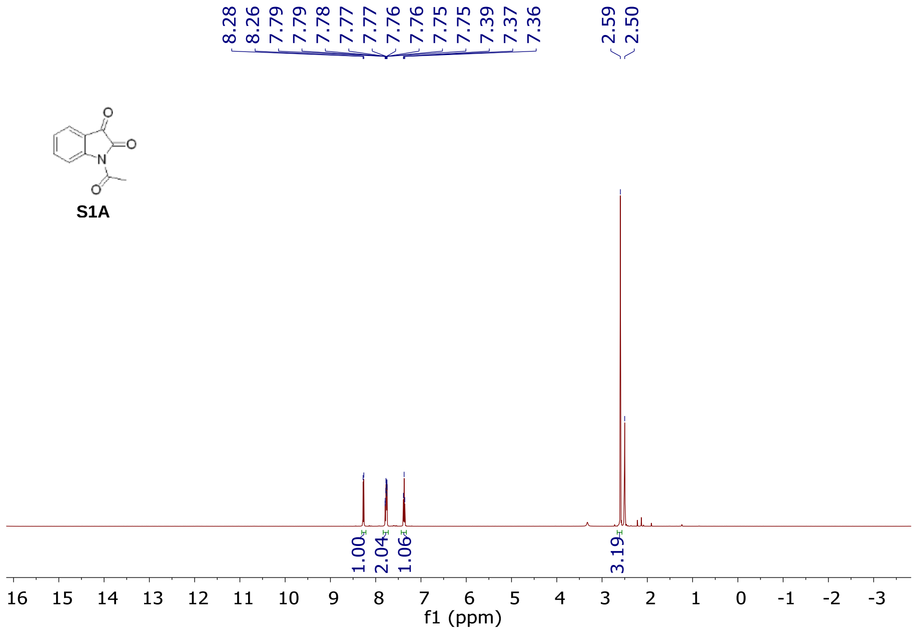

^{1}H NMR of S1A

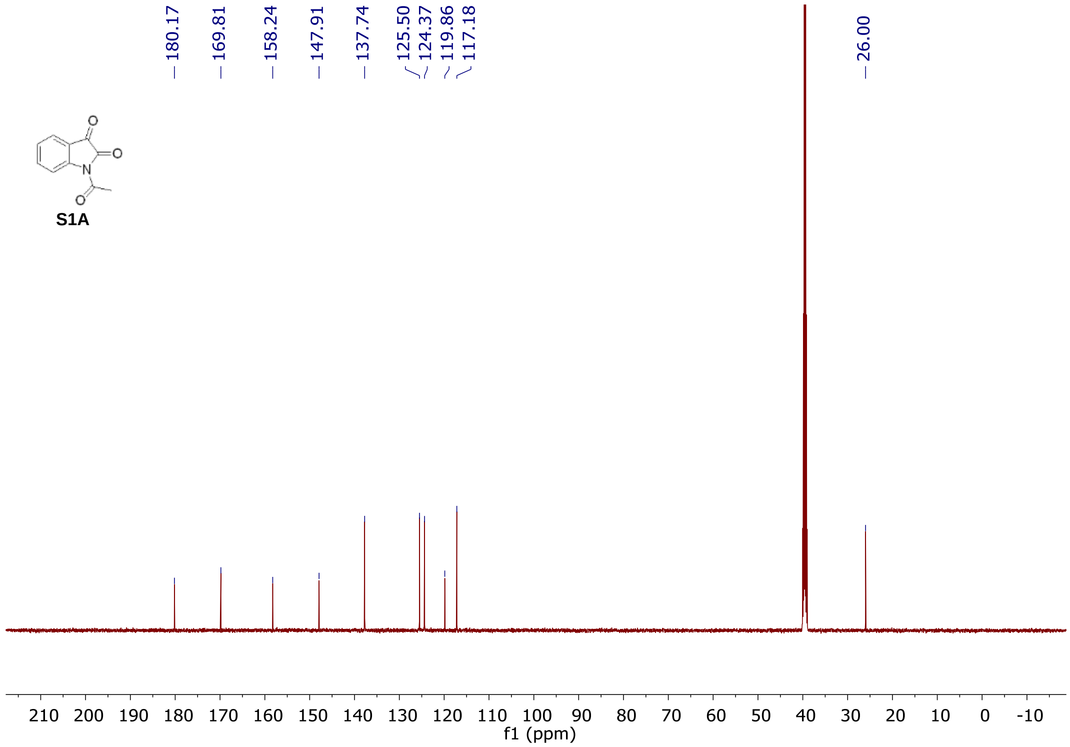

^{13}C NMR of S1A

Supplementary Fig. 45. NMR of S1B

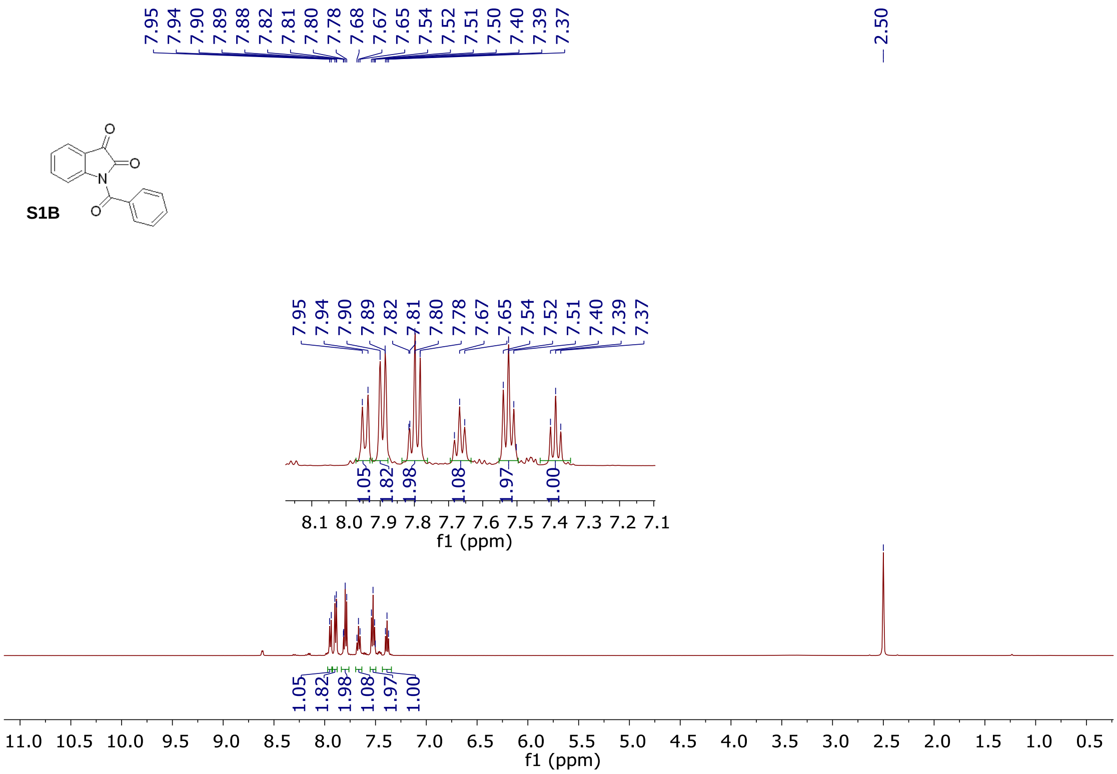

^{1}H NMR of S1B

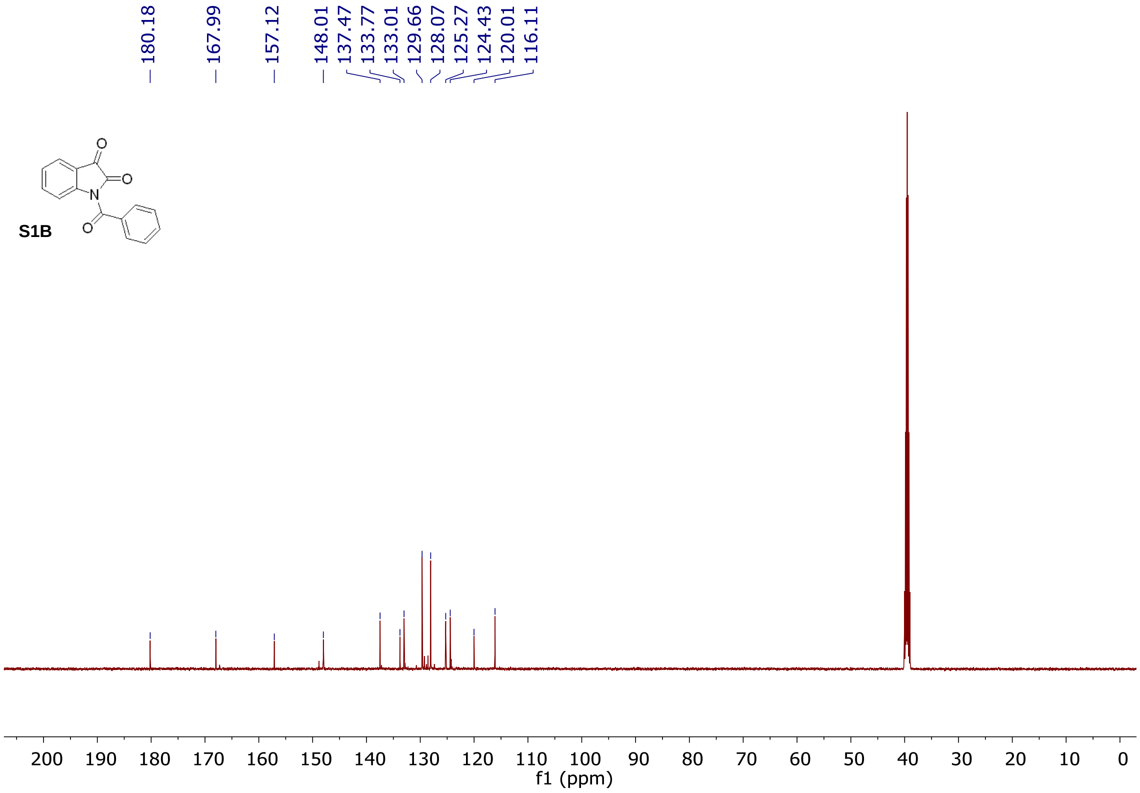

^{13}C NMR of S1B

Supplementary Fig. 46. NMR of S1C

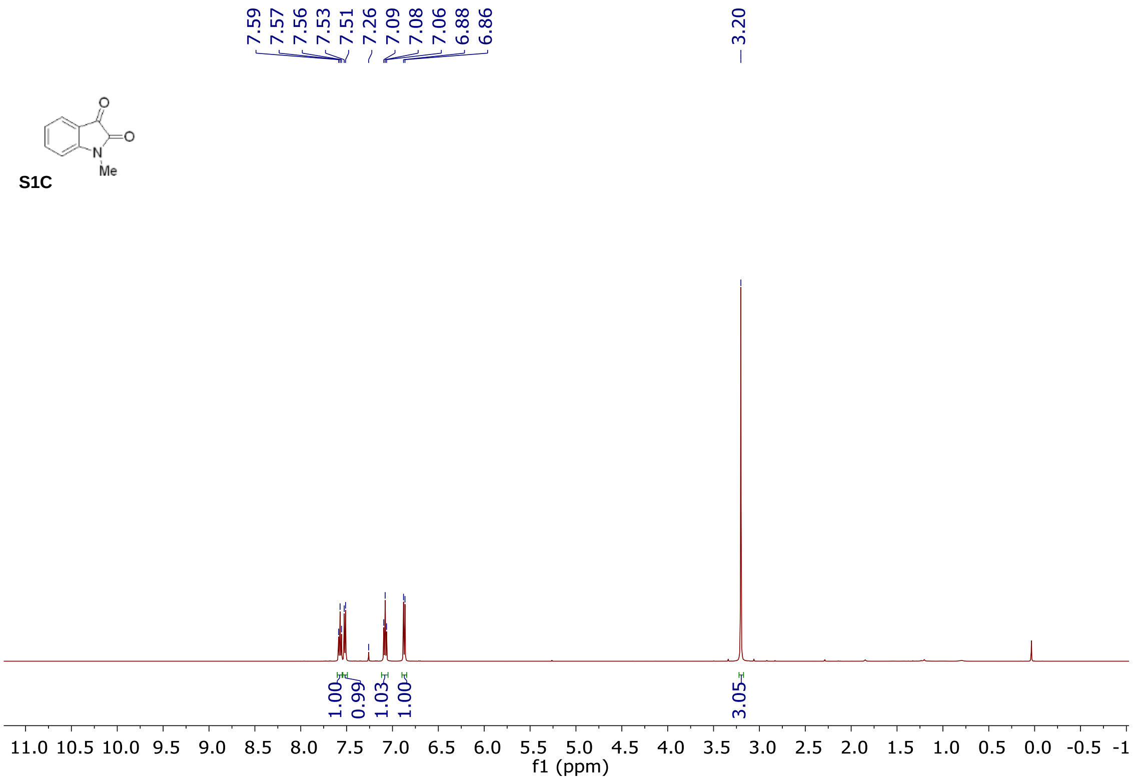

^{1}H NMR of S1C

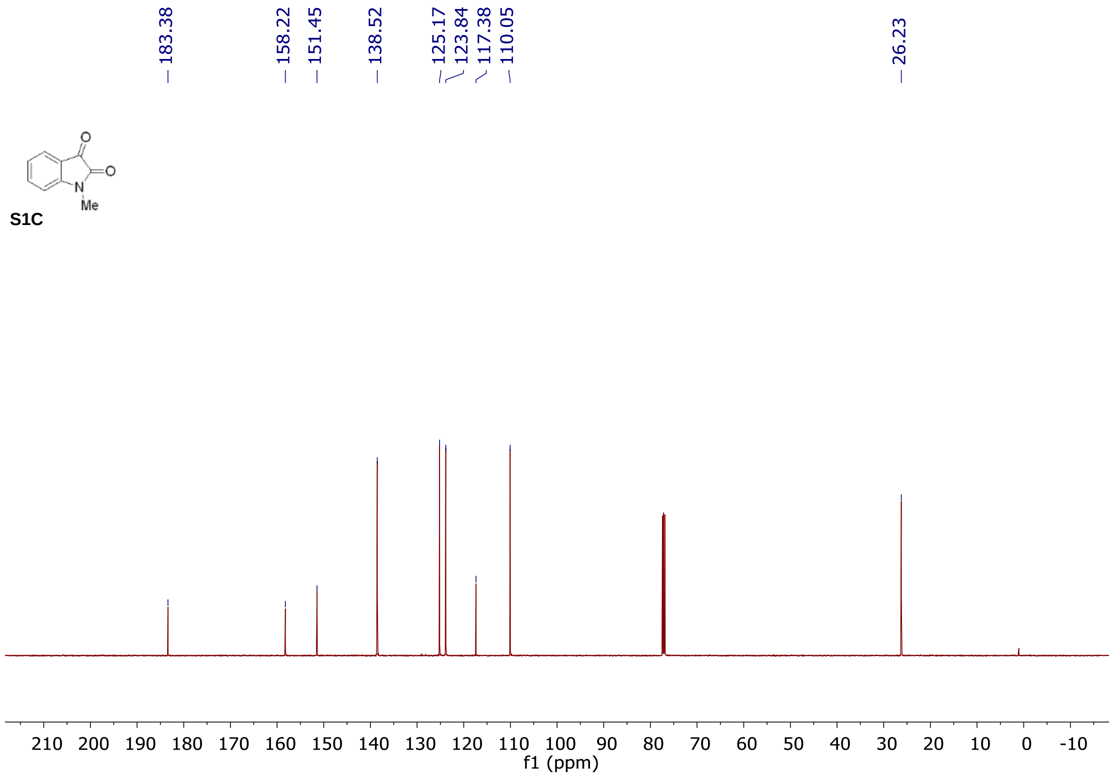

^{13}C NMR of S1C

Supplementary Fig. 47. NMR of S1F

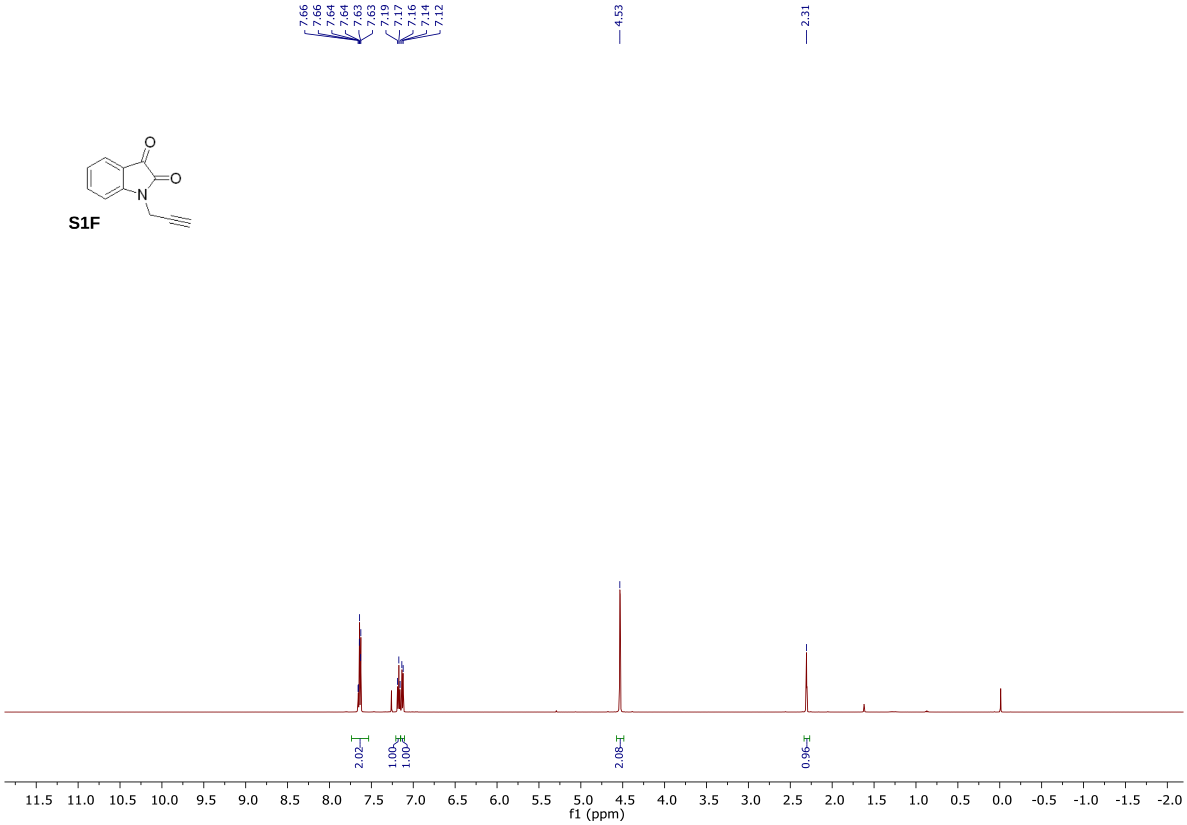

^{1}H NMR of S1F

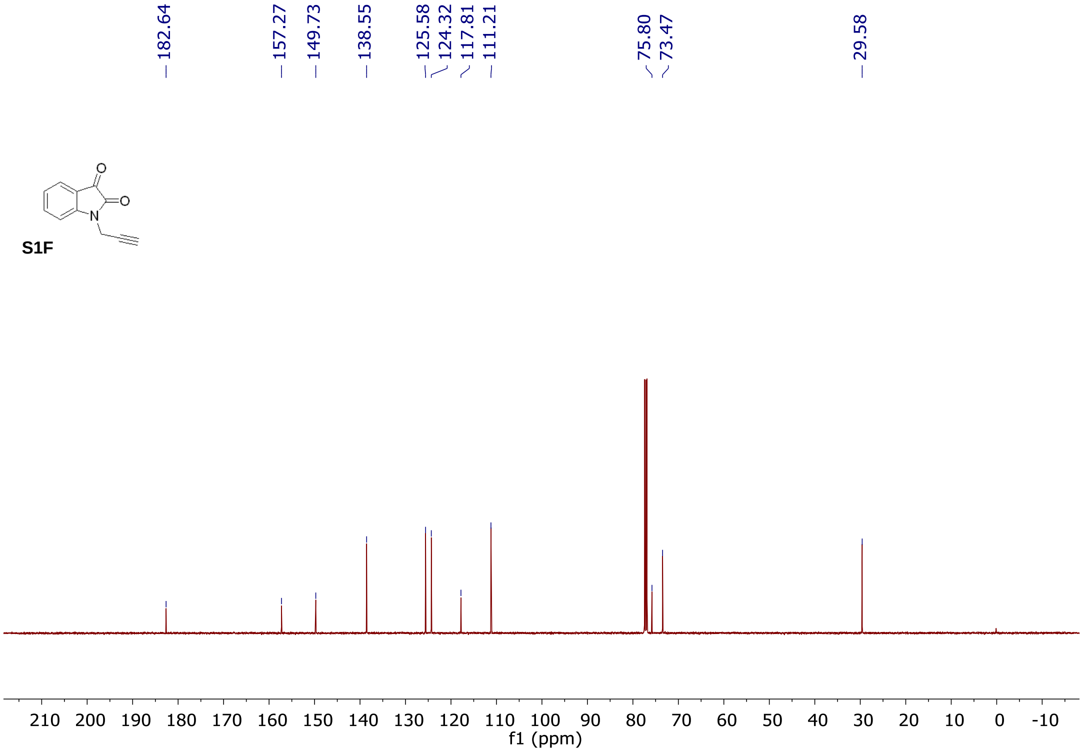

^{13}C NMR of S1F

Supplementary Fig. 48. NMR of S1D

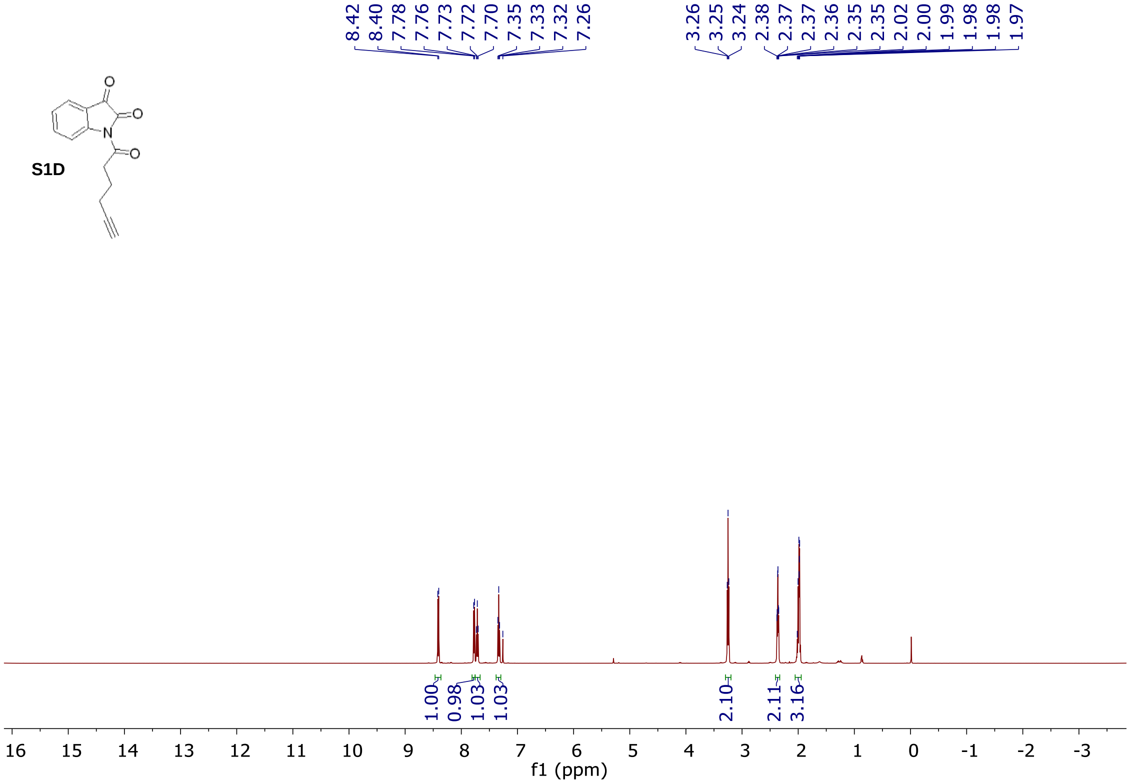

^{1}H NMR of S1D

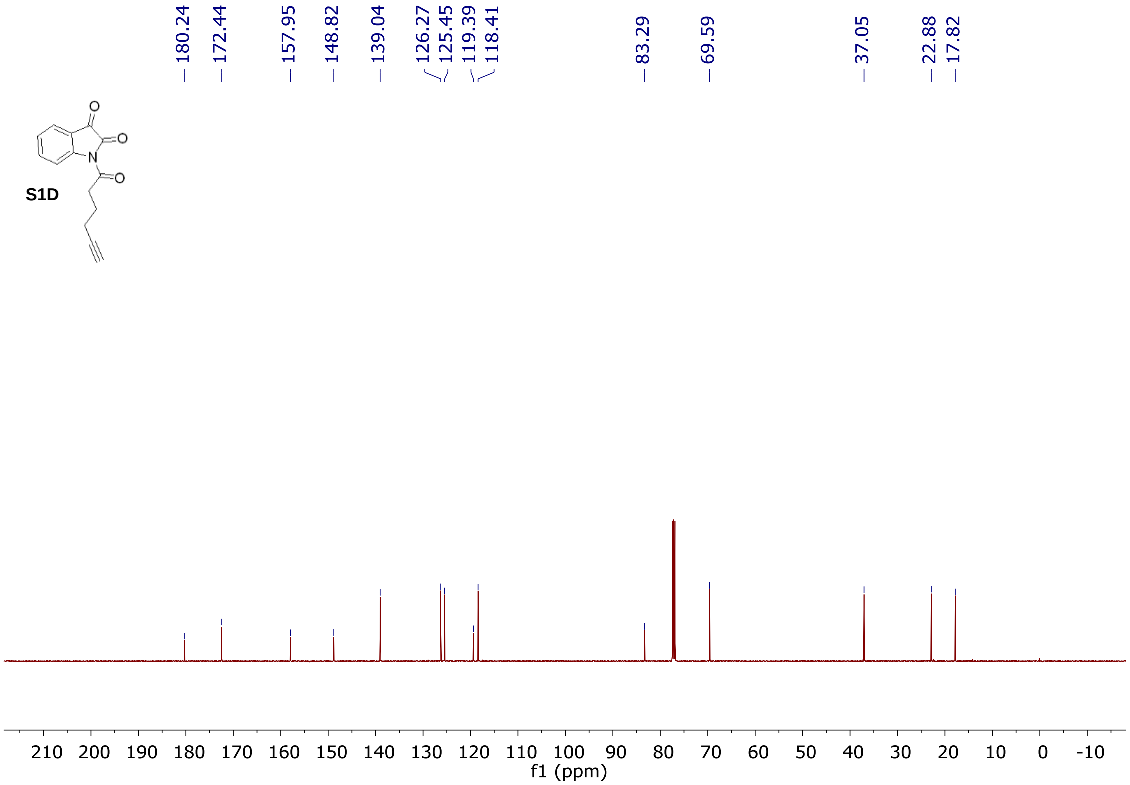

^{13}C NMR of S1D

Supplementary Fig. 49. NMR of S1E

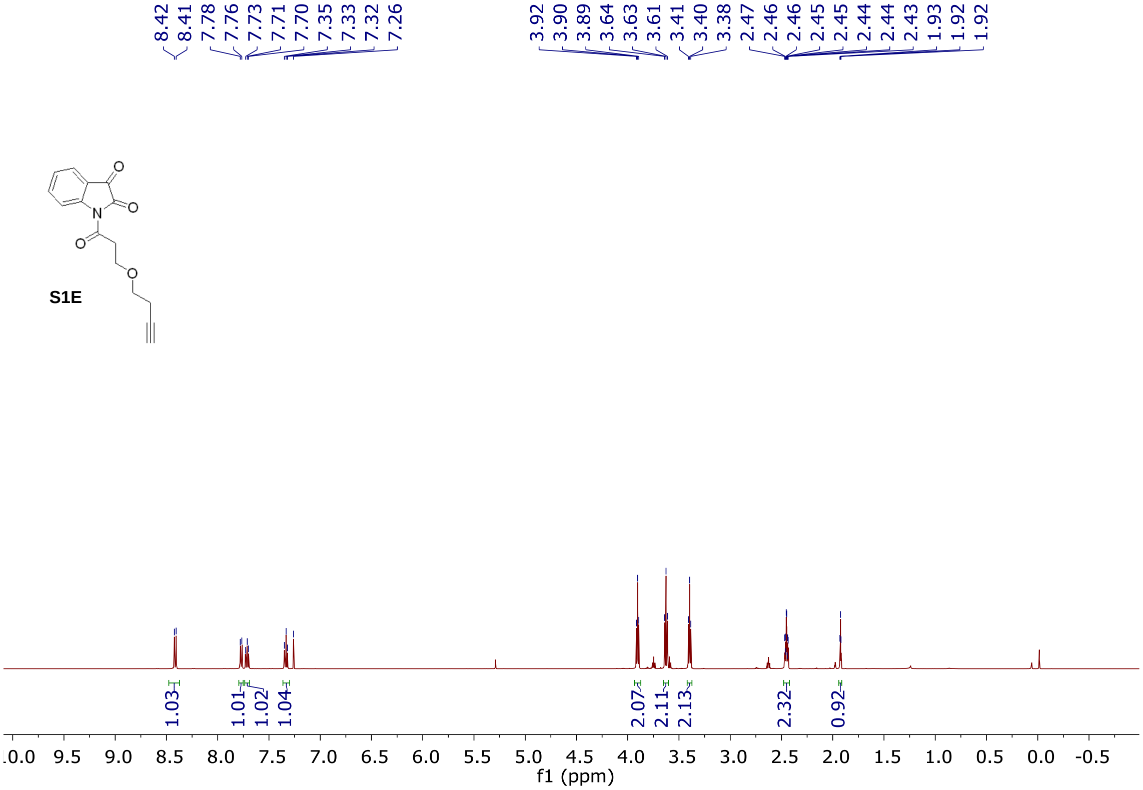

^{1}H NMR of S1E

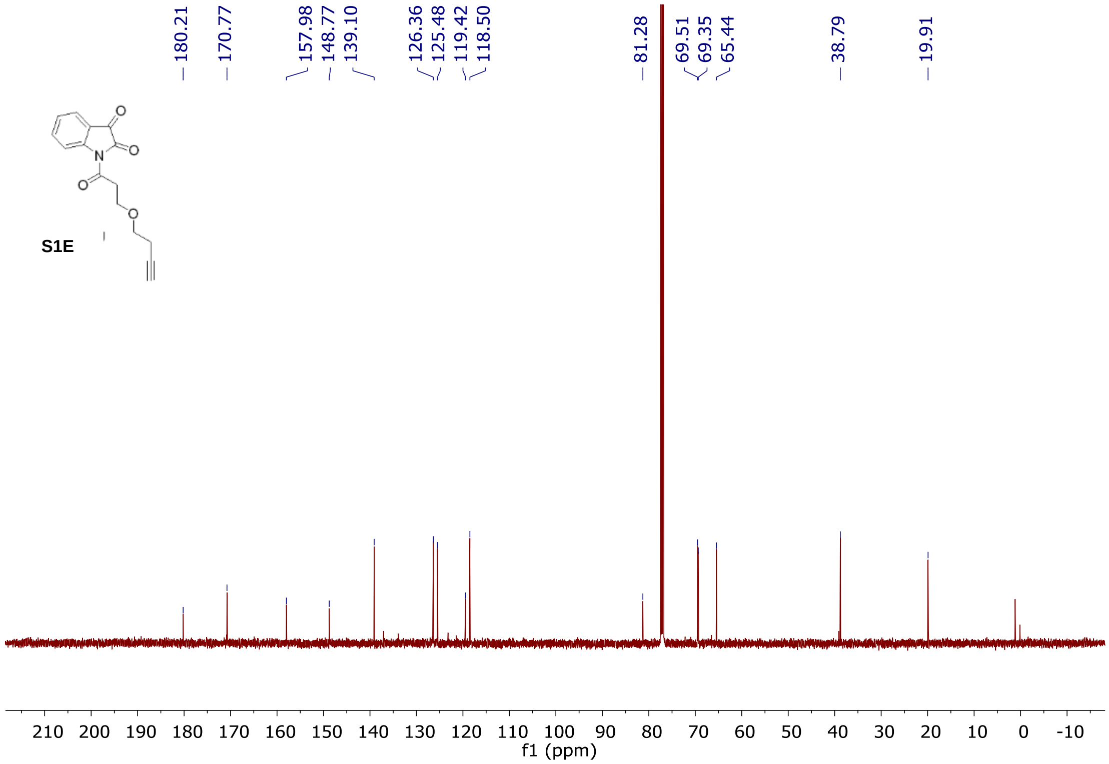

^{13}C NMR of S1E

Supplementary Fig. 50. NMR of S4

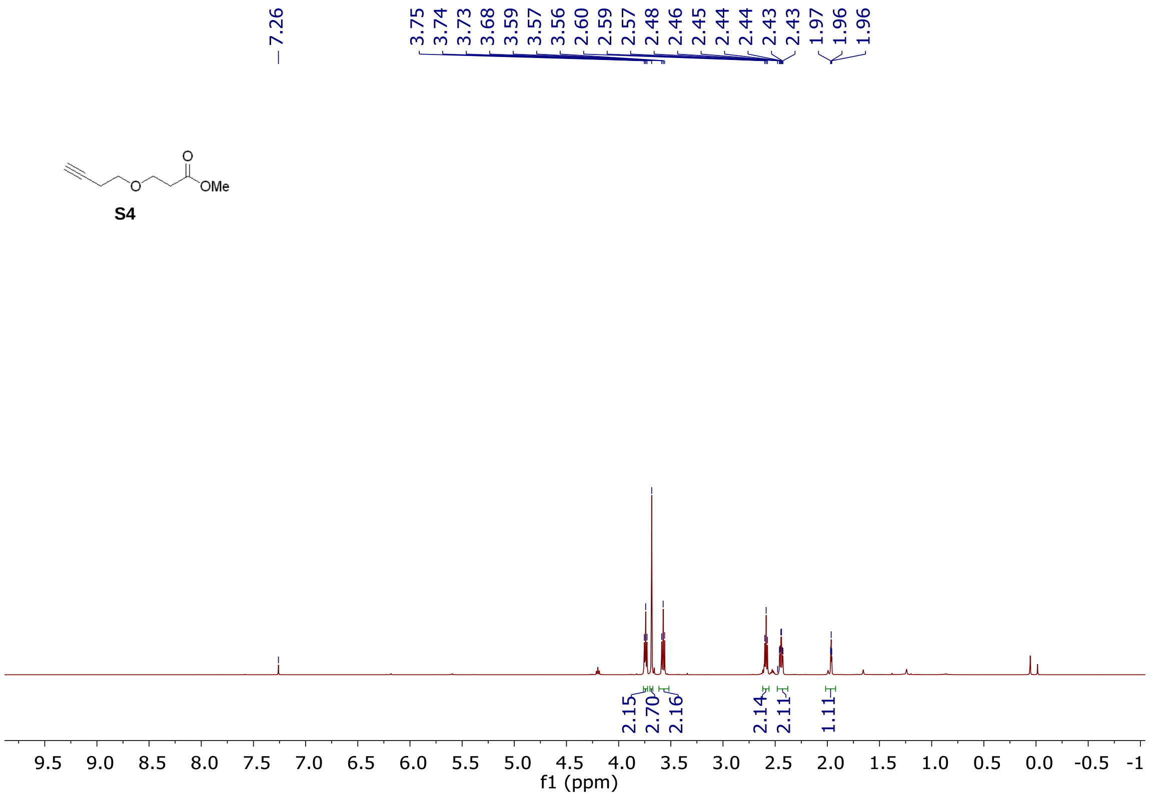

^{1}H NMR of S4

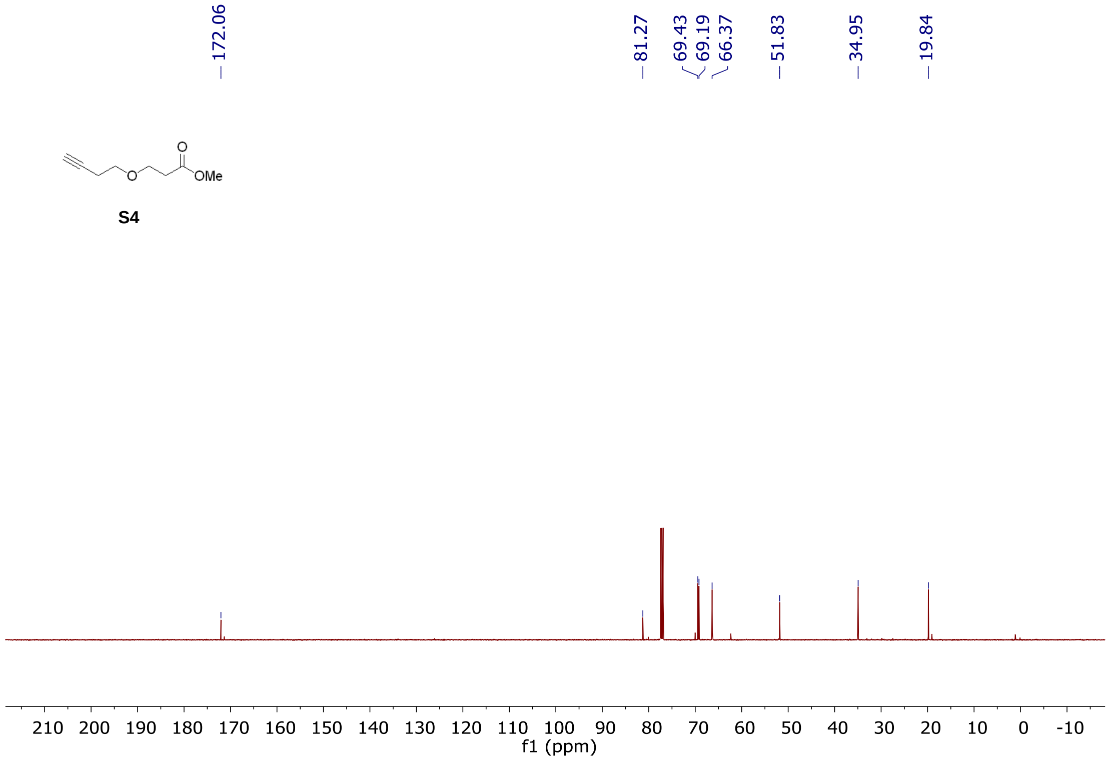

^{13}C NMR of S4

Supplementary Fig. 51. NMR of S5

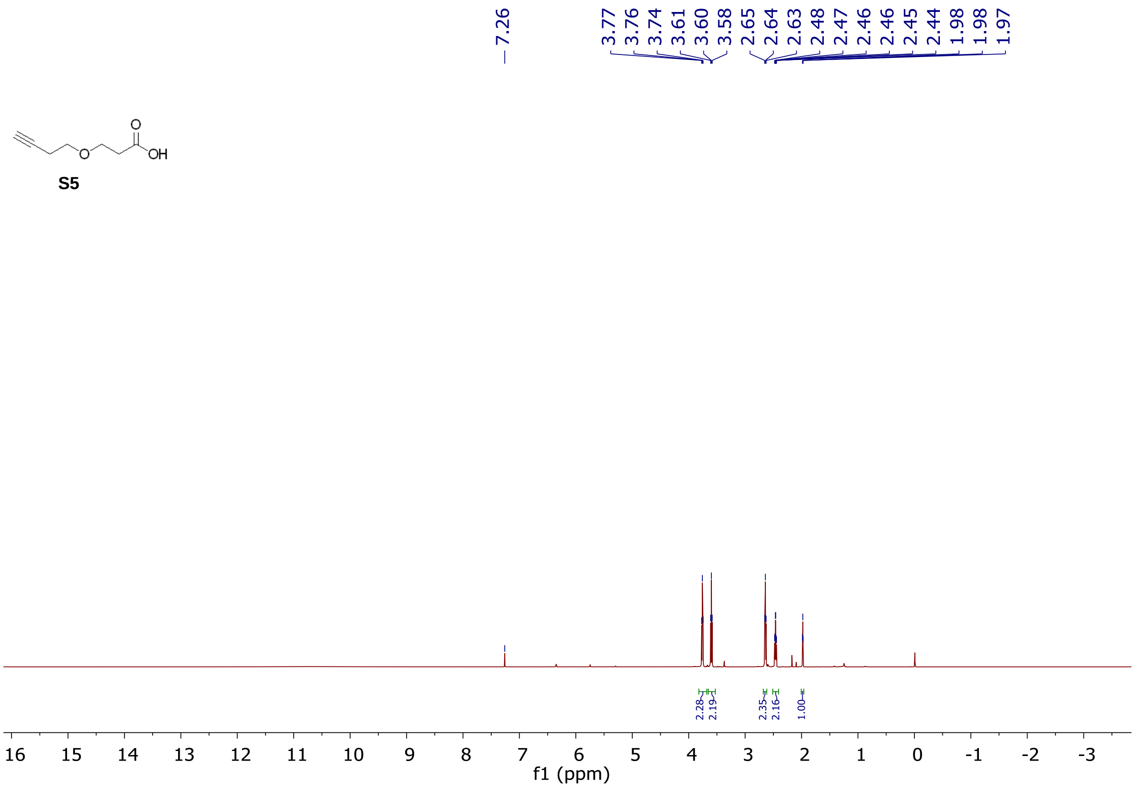

^{1}H NMR of S5

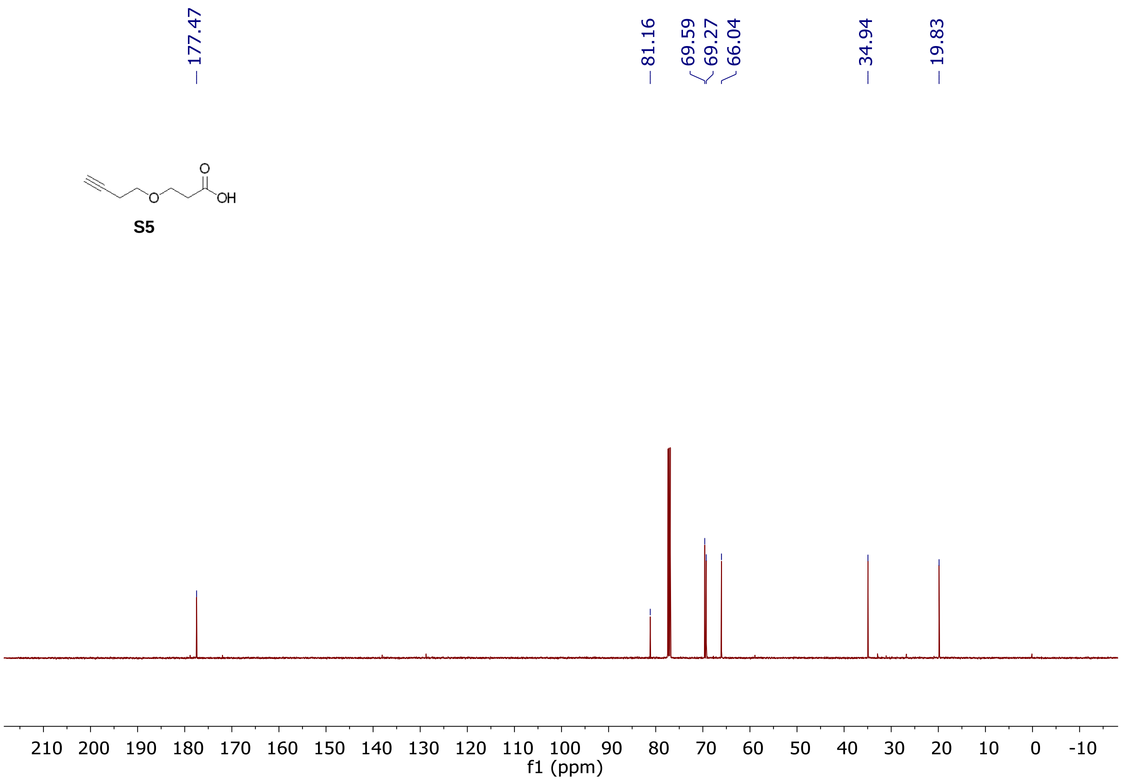

^{13}C NMR of S5

Supplementary Fig. 52. NMR of S2A

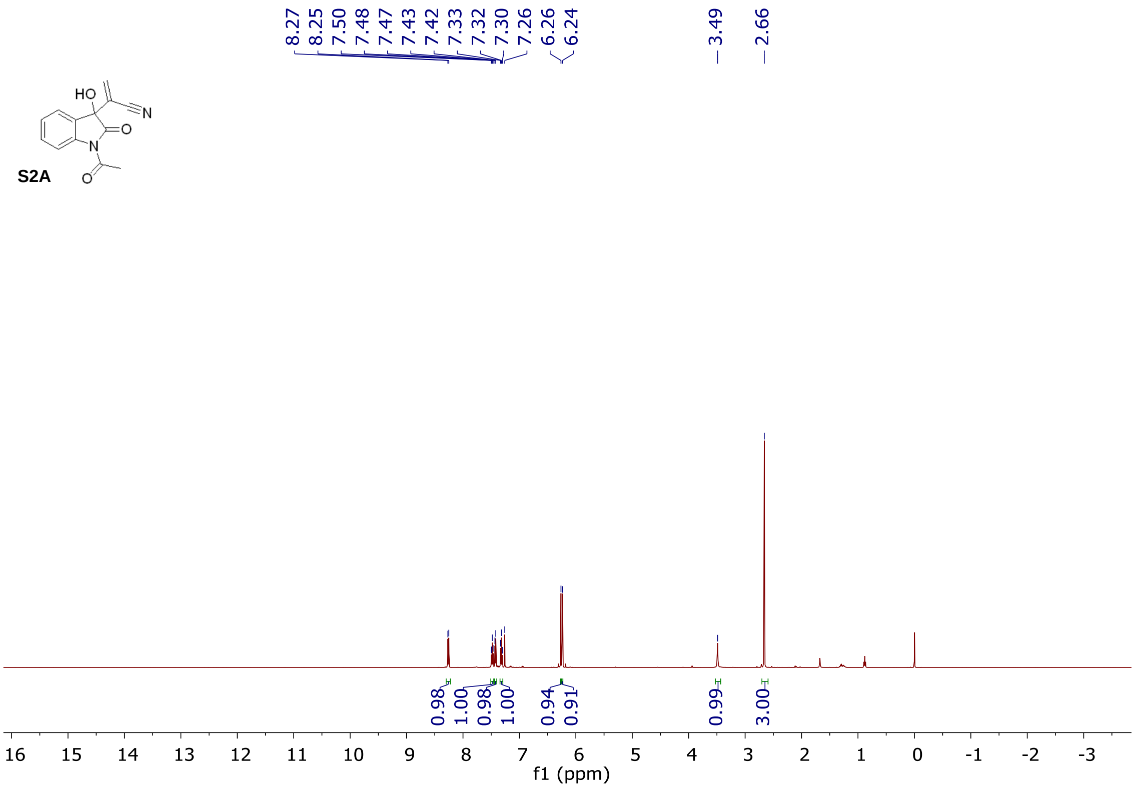

^{1}H NMR of S2A

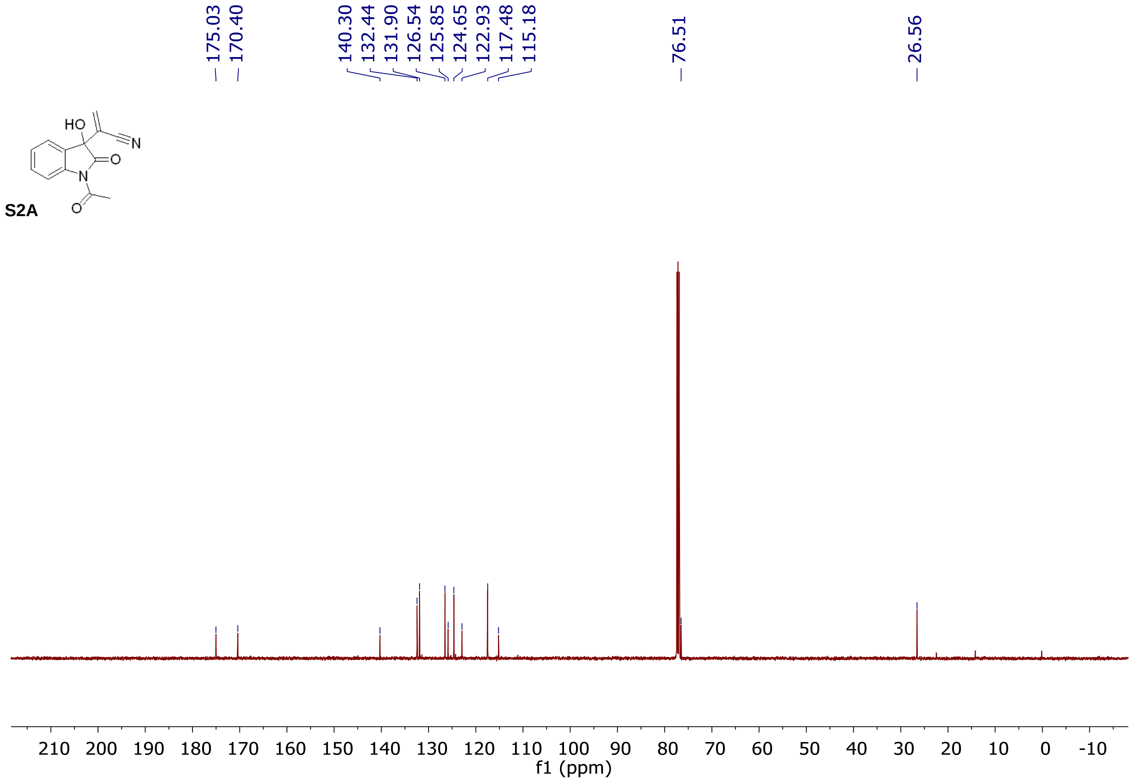

^{13}C NMR of S2A

Supplementary Fig. 53. NMR of S2B

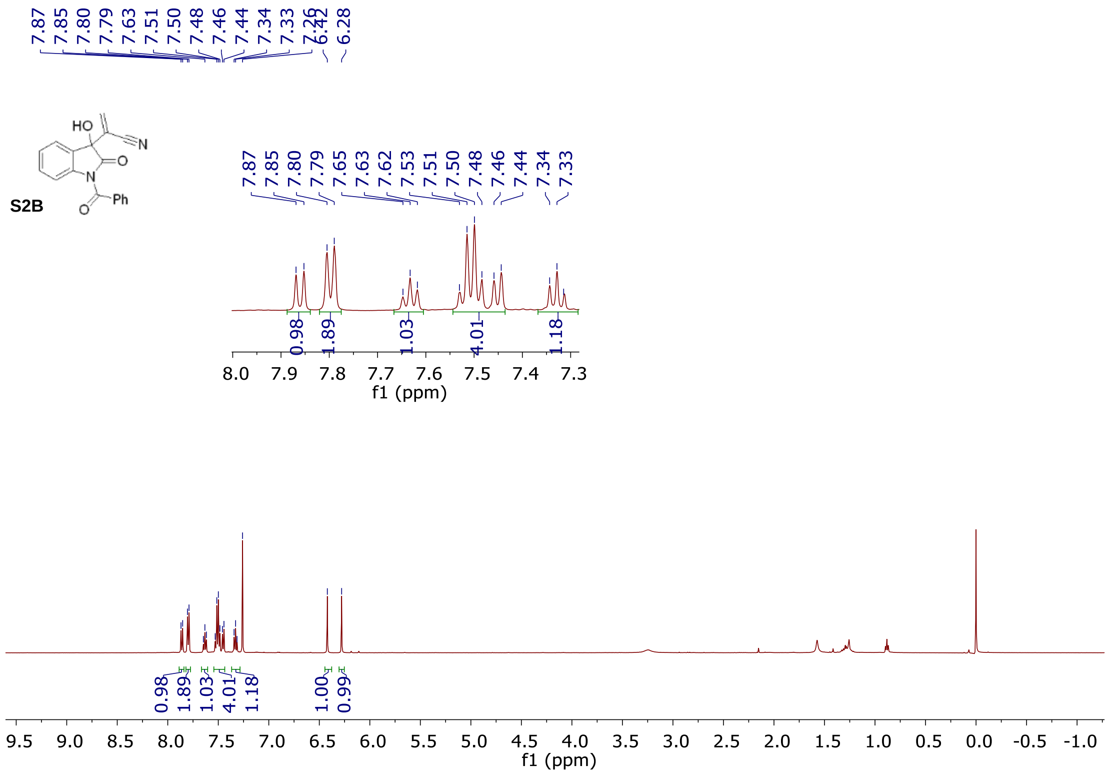

^{1}H NMR of S2B

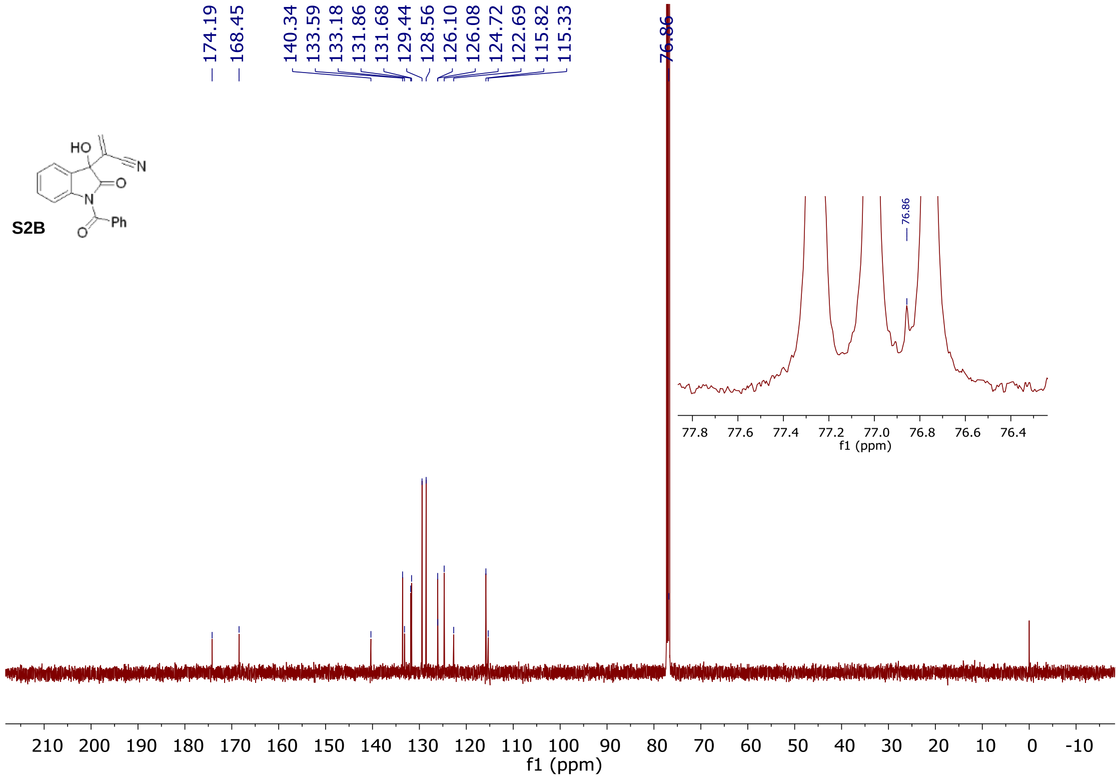

^{13}C NMR of S2B

Supplementary Fig. 54. NMR of S2C

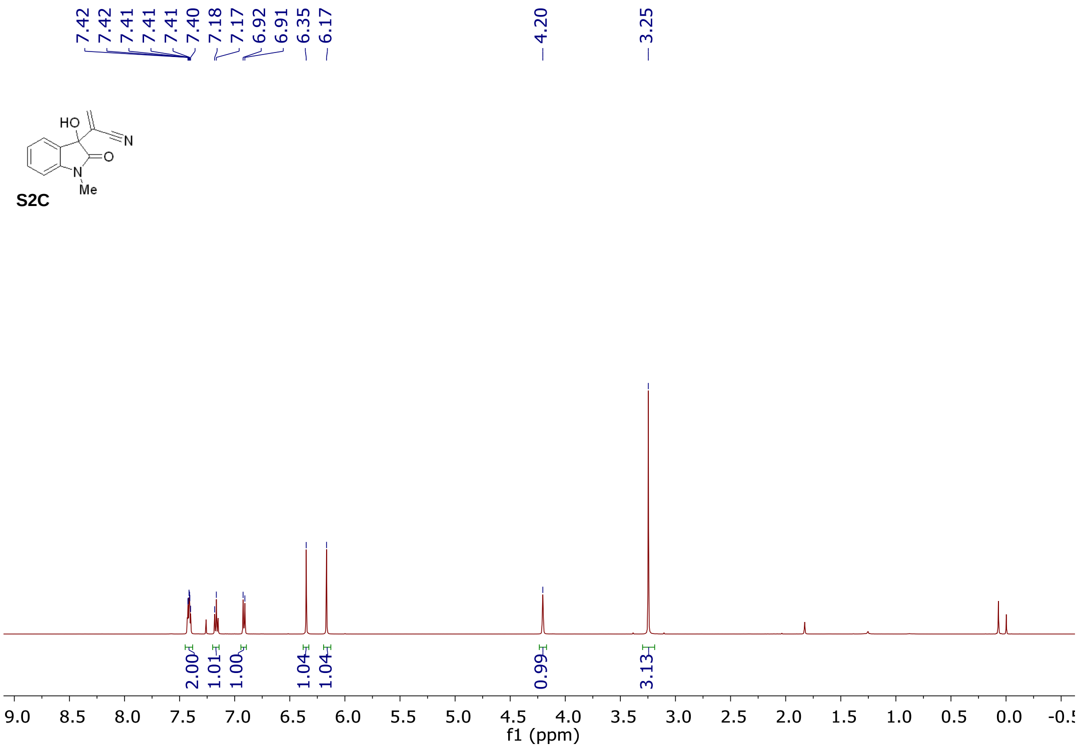

^{1}H NMR of S2C

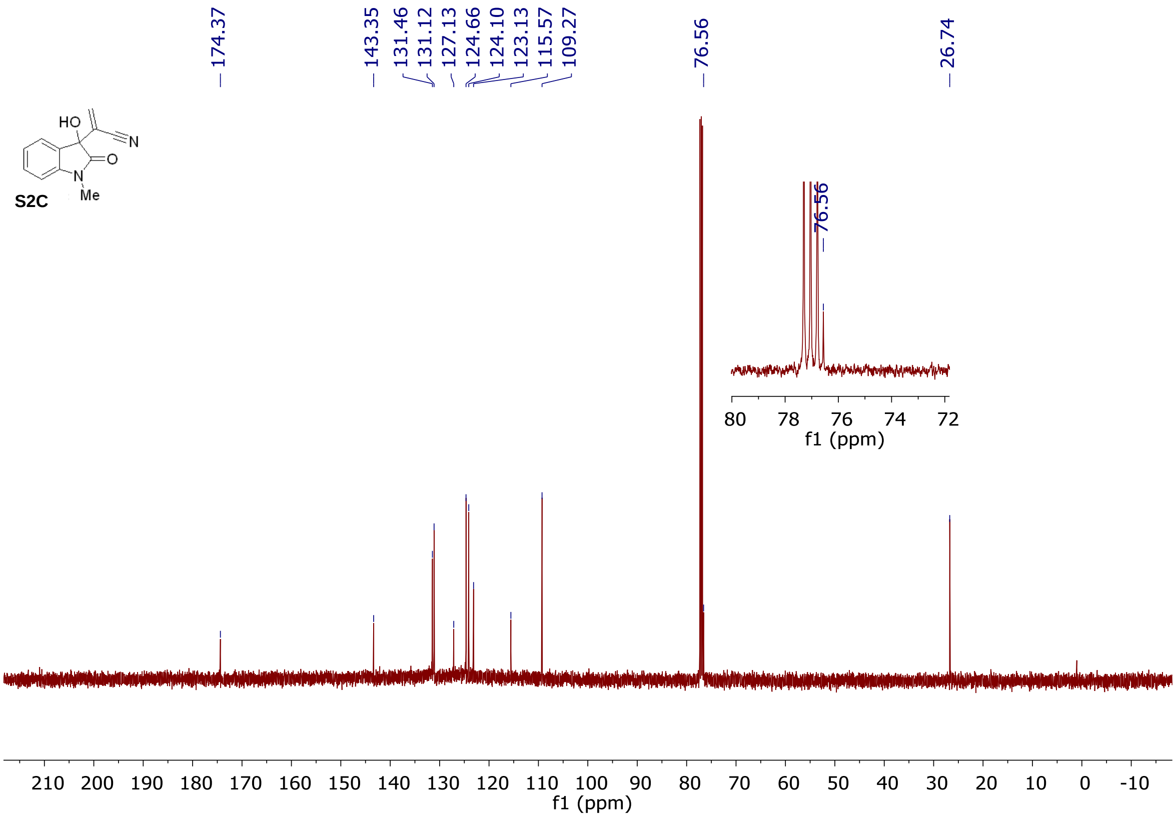

^{13}C NMR of S2C

Supplementary Fig. 55. NMR of S2F

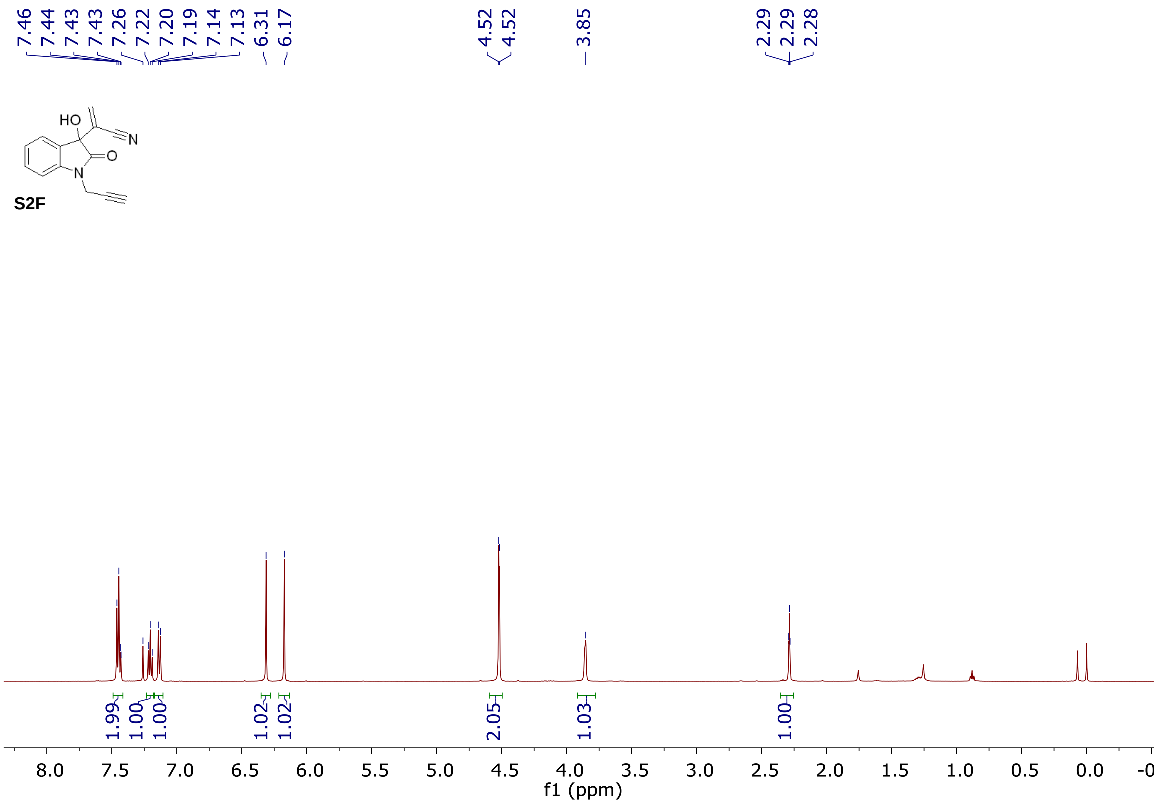

^{1}H NMR of S2F

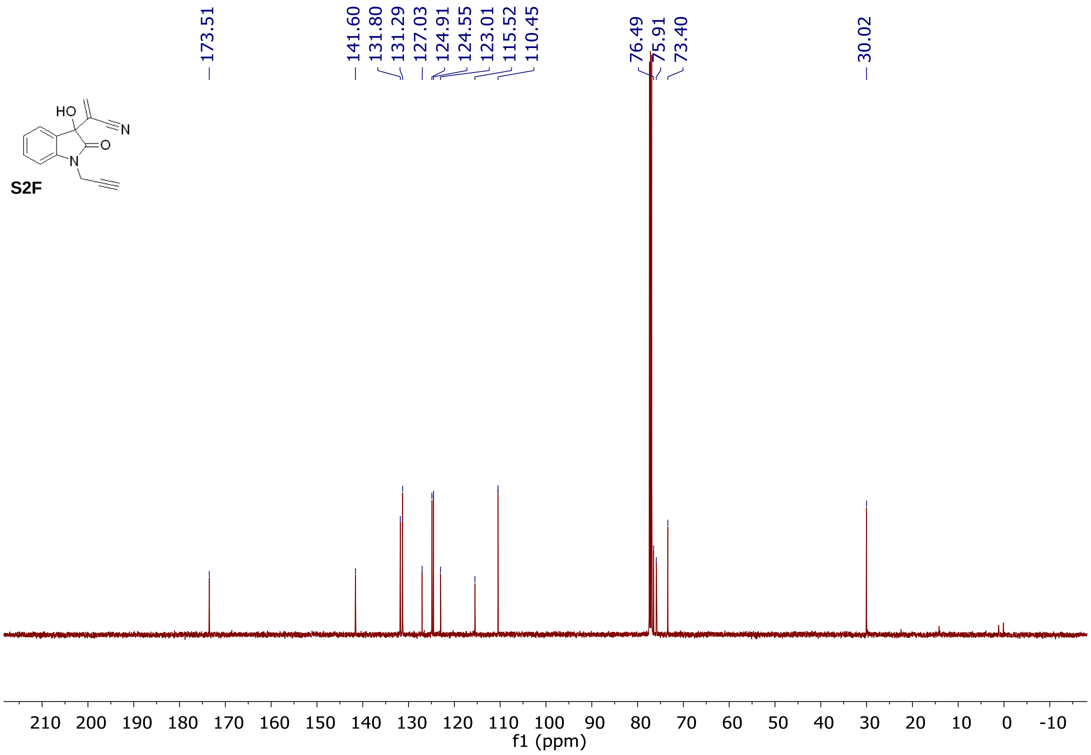

^{13}C NMR of S2F

Supplementary Fig. 56. NMR of S2D

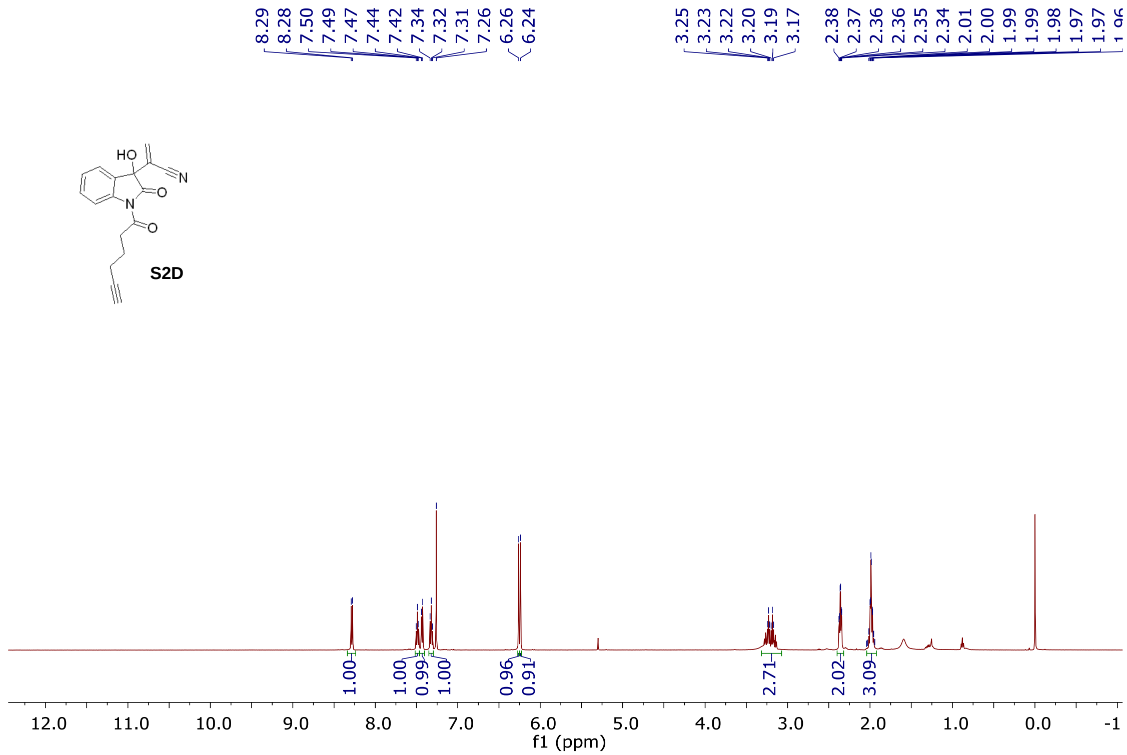

^{1}H NMR of S2D

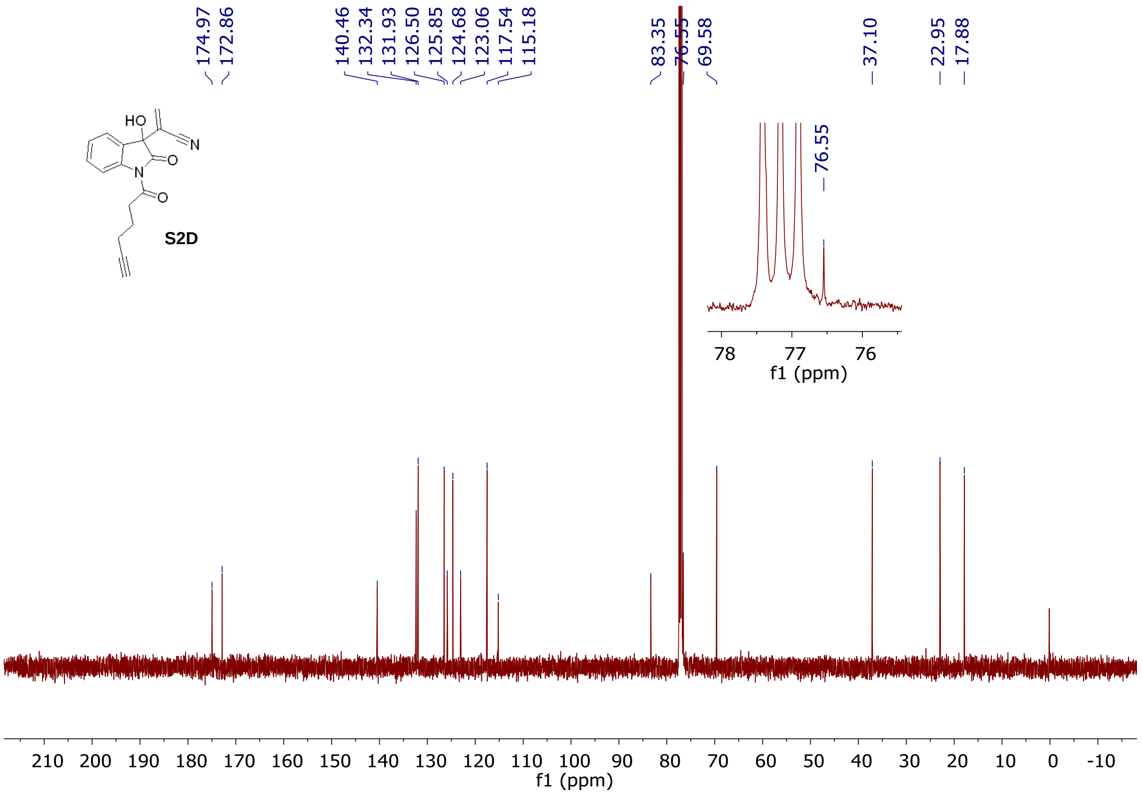

^{13}C NMR of S2D

Supplementary Fig. 57. NMR of S2E

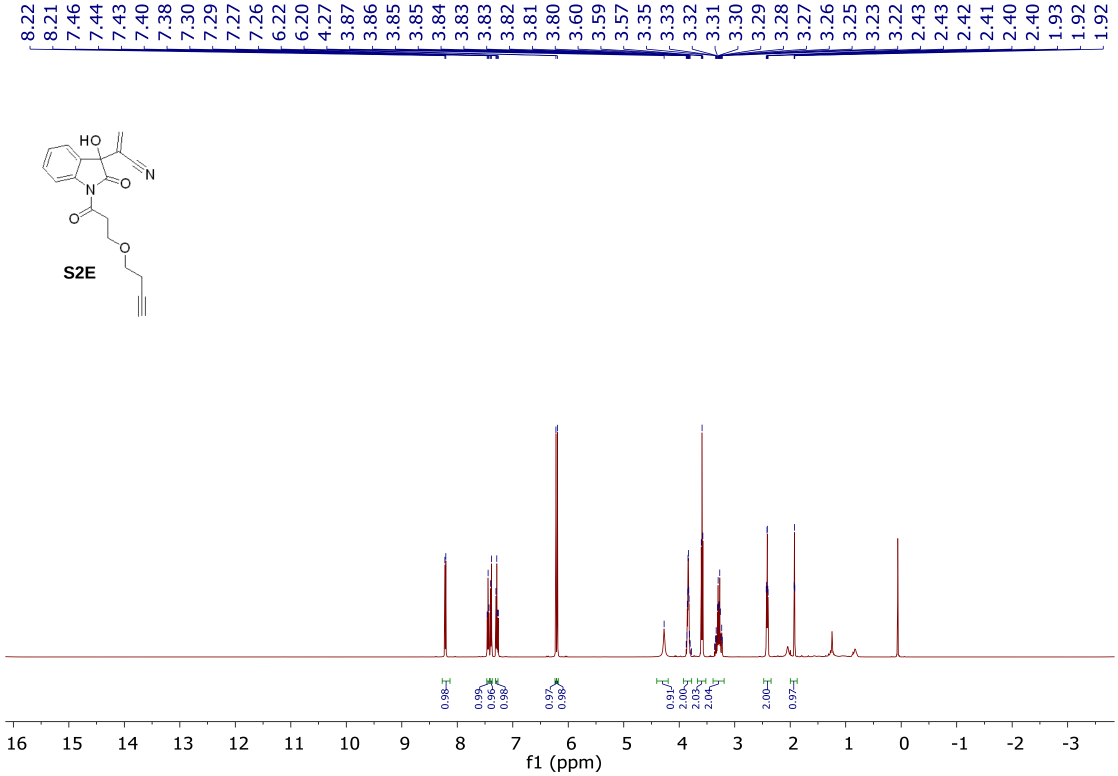

^{1}H NMR of S2E

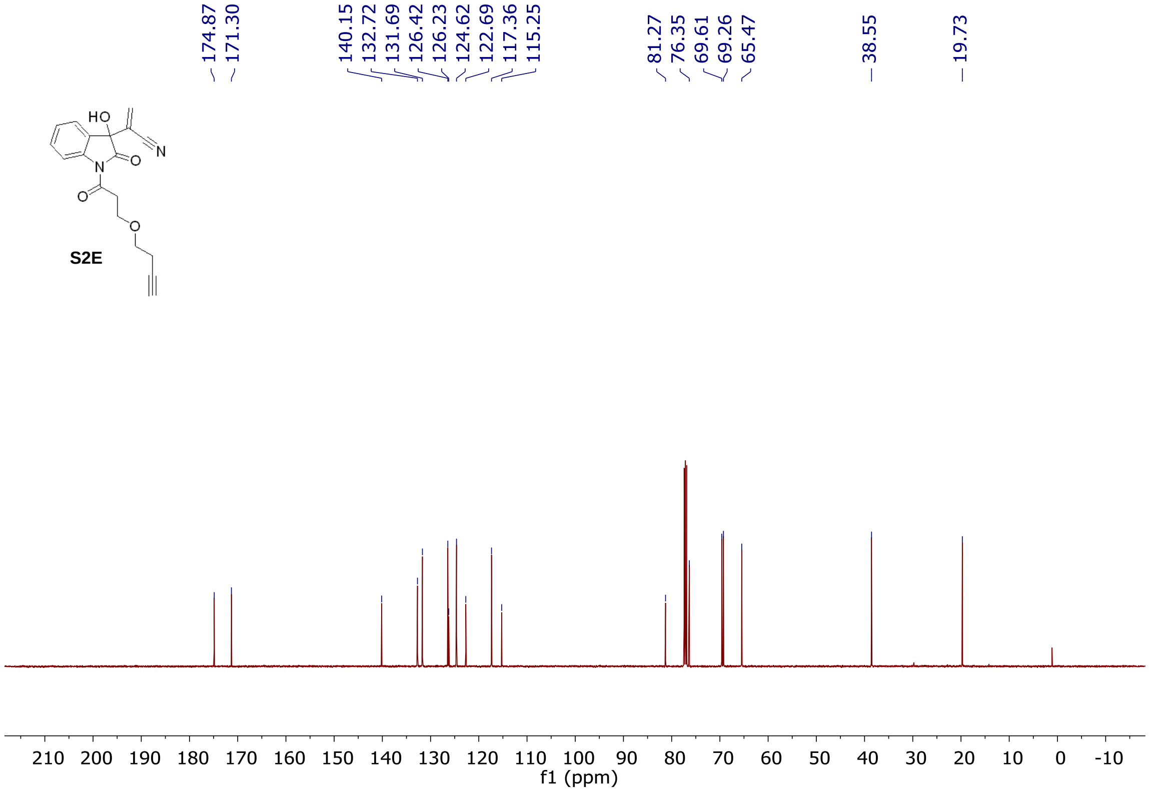

^{13}C NMR of S2E

Supplementary Fig. 58. NMR of 1A

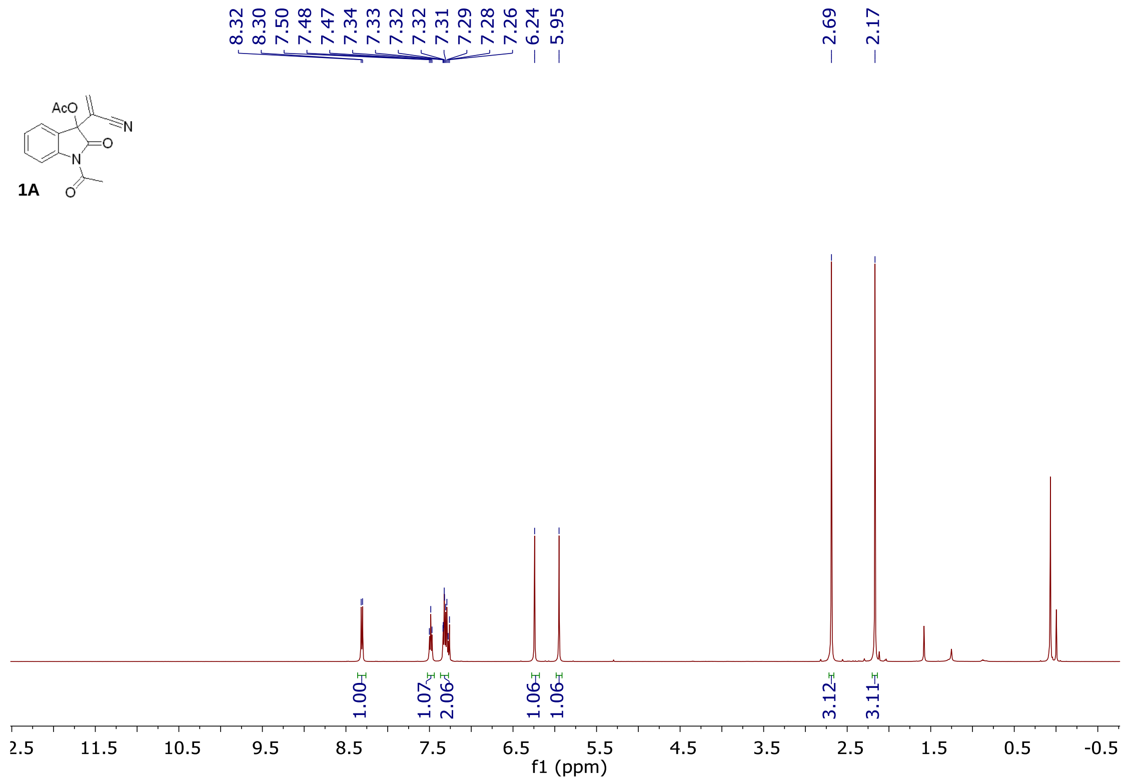

^{1}H NMR of 1A

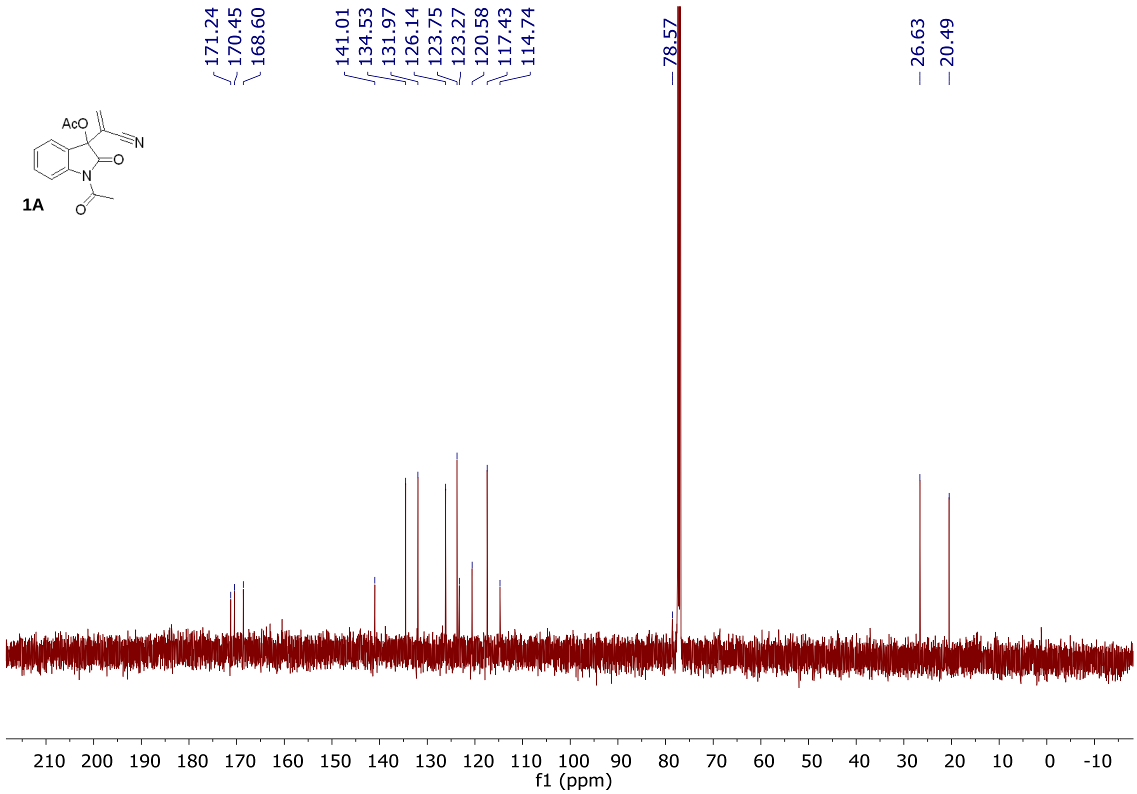

^{13}C NMR of 1A

Supplementary Fig. 59. NMR of 1B

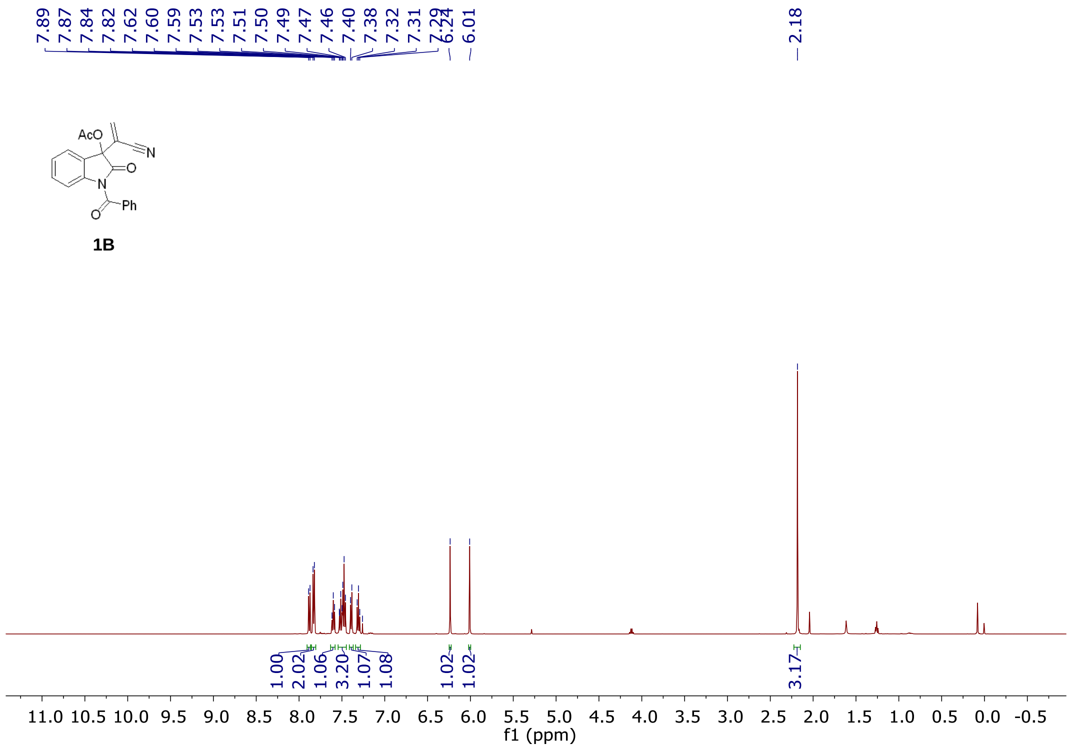

^{1}H NMR of 1B

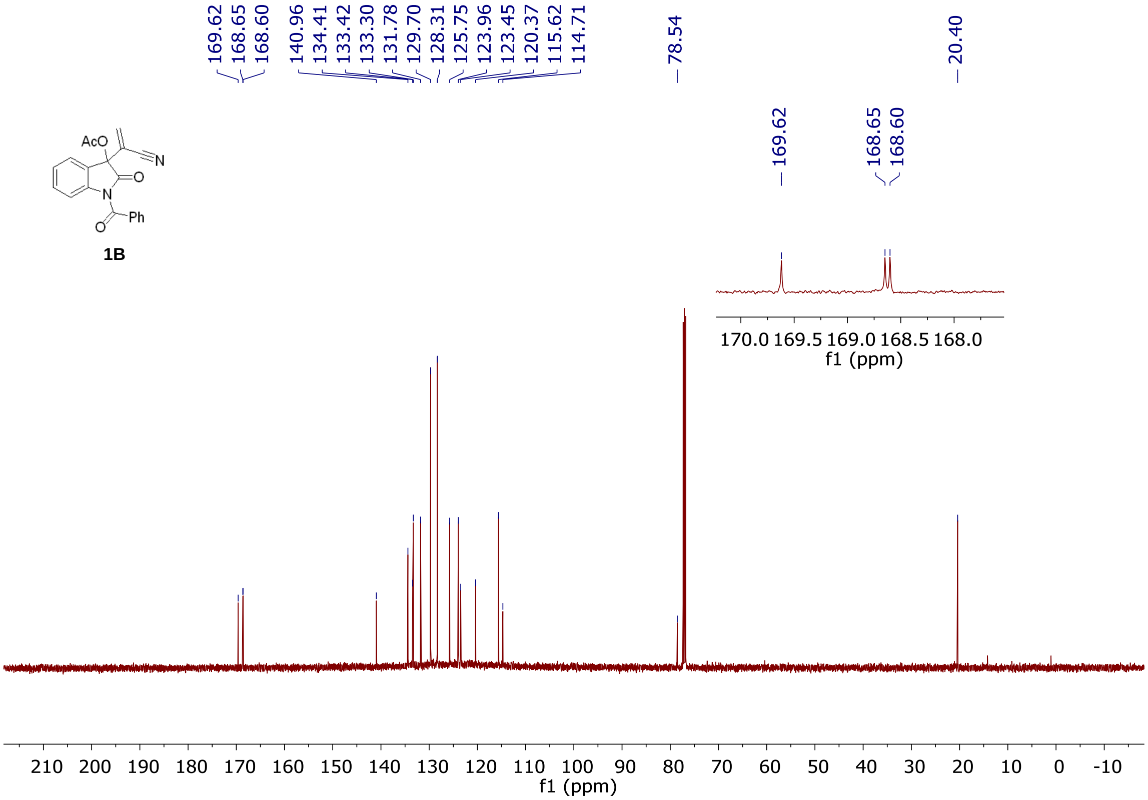

^{13}C NMR of 1B

Supplementary Fig. 60. NMR of 1C

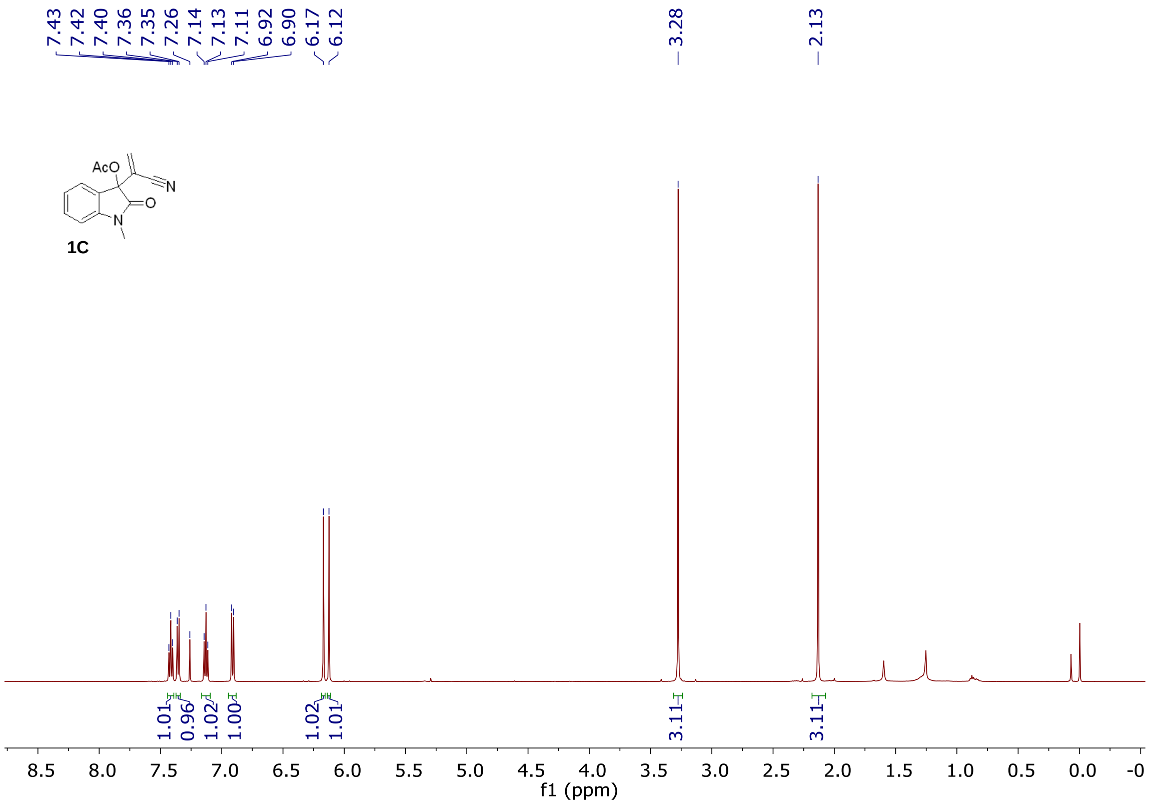

^{1}H NMR of 1C

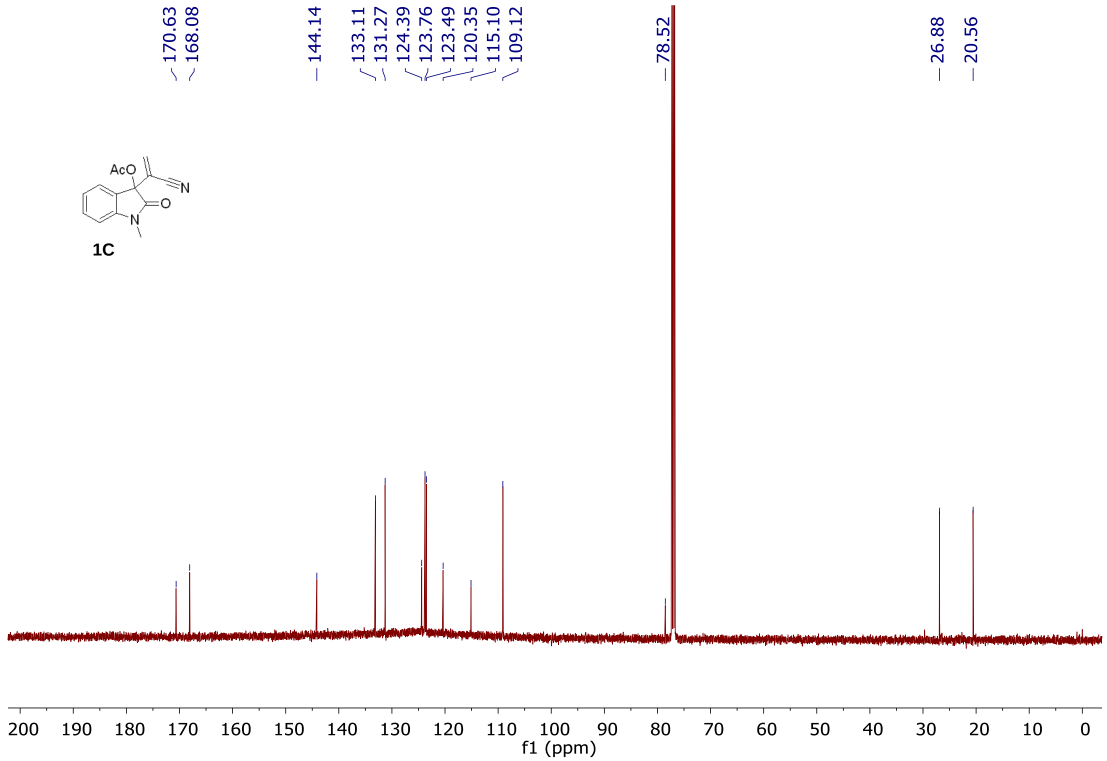

^{13}C NMR of 1C

Supplementary Fig. 61. NMR of 1F

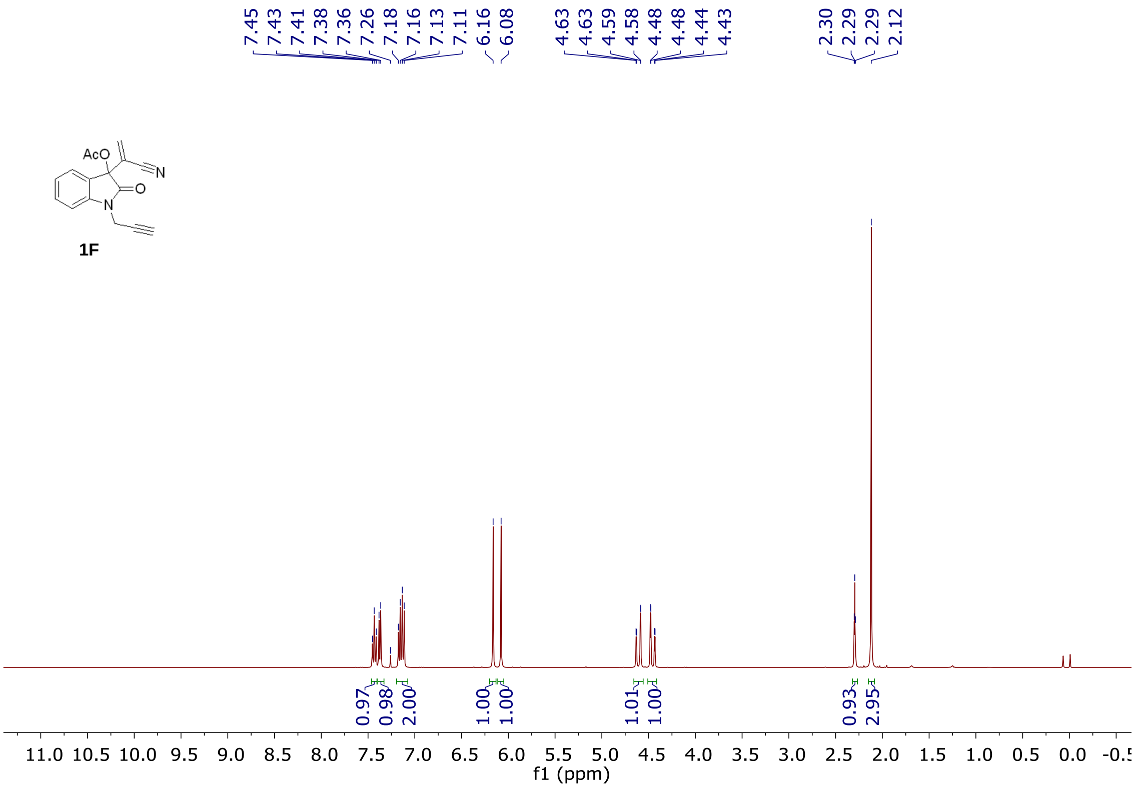

^{1}H NMR of 1F

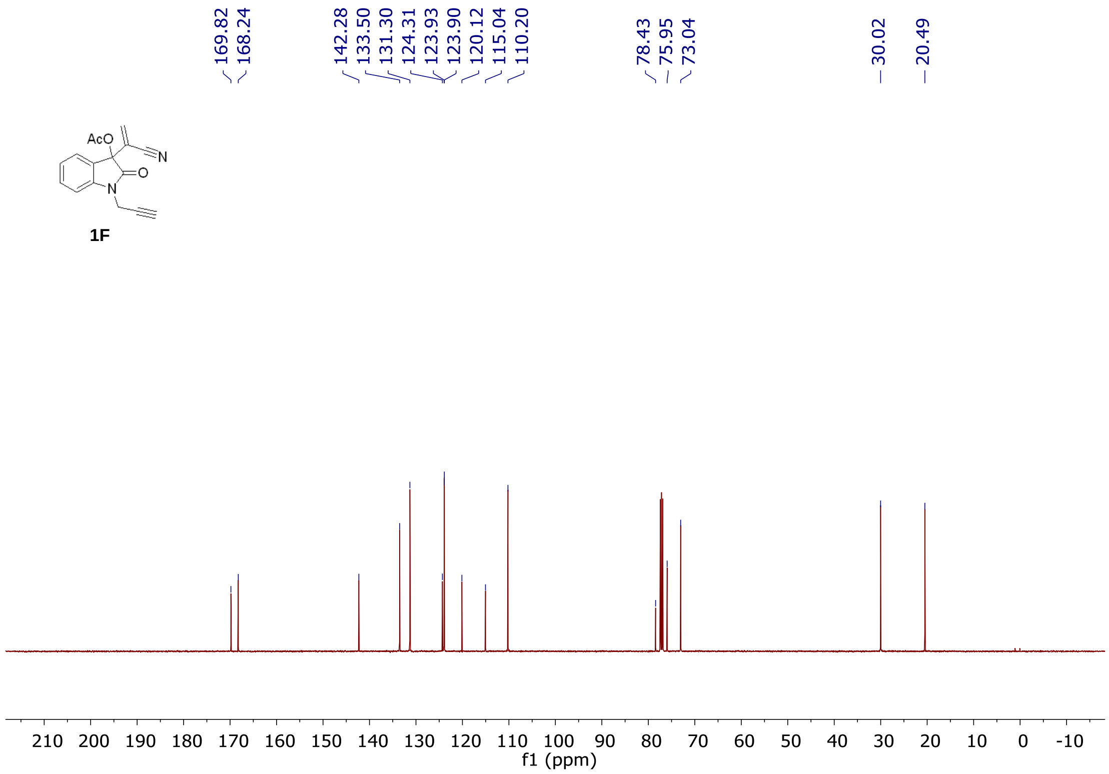

^{13}C NMR of 1F

Supplementary Fig. 62. NMR of 1D

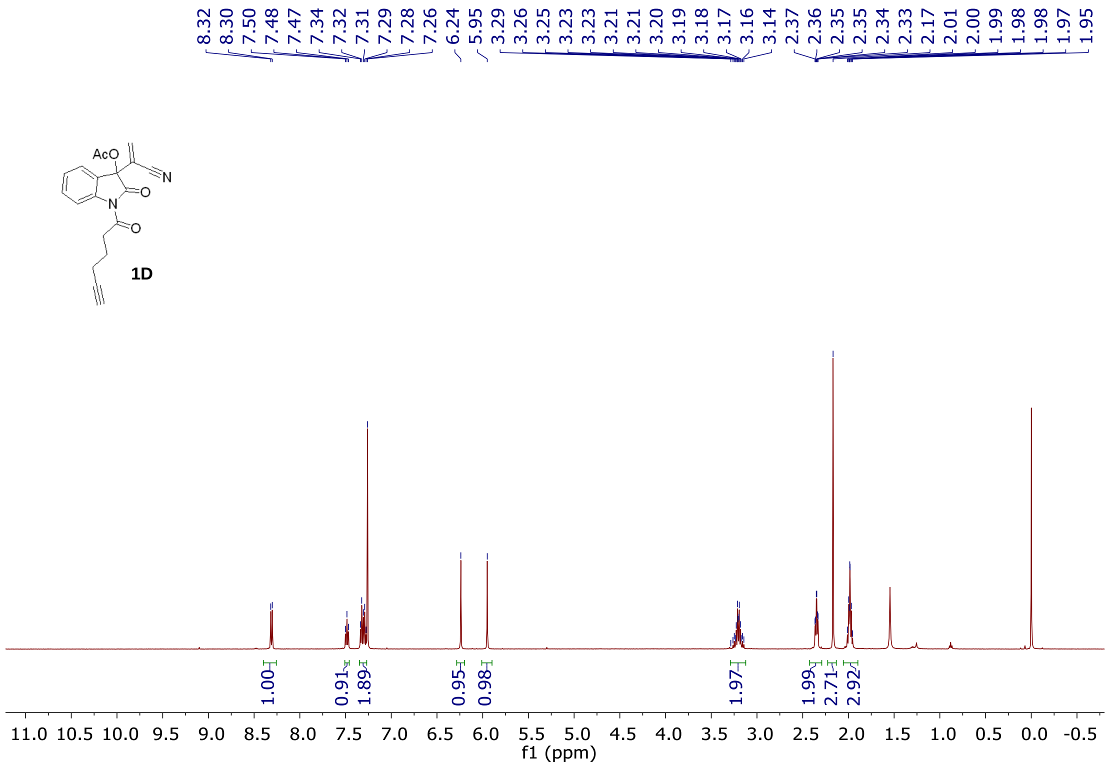

^{1}H NMR of 1D

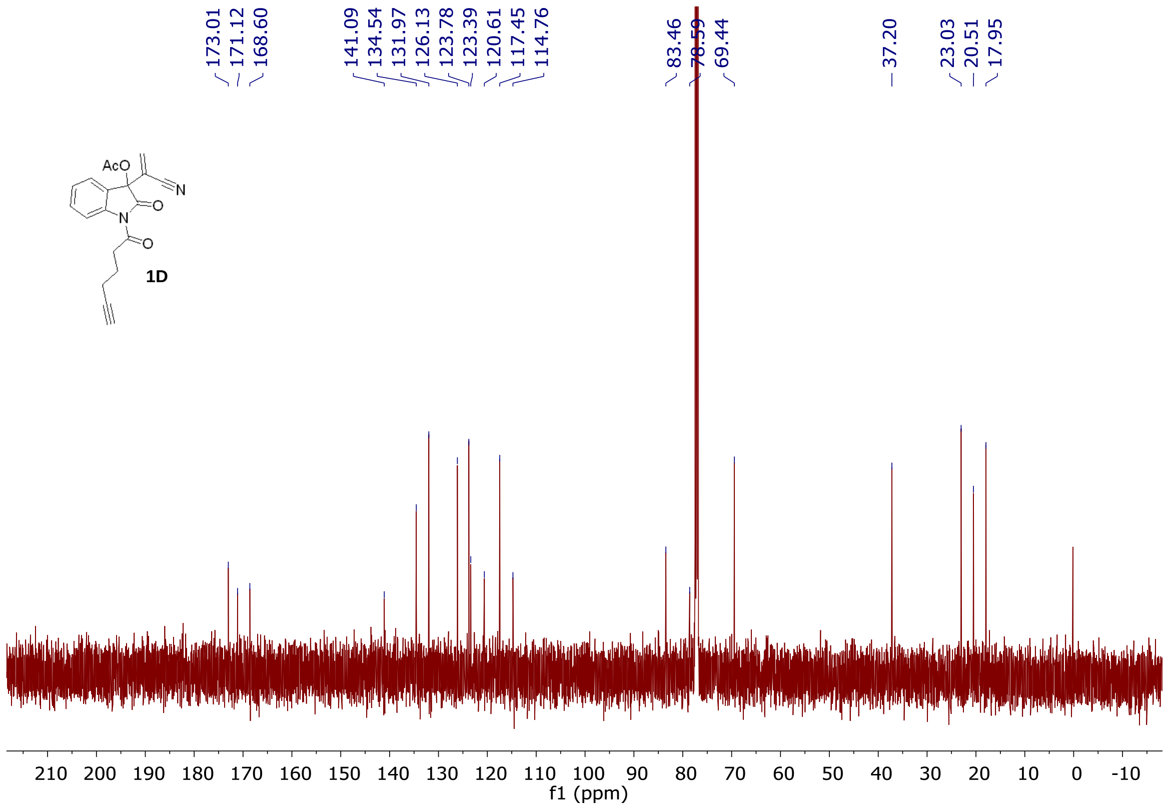

^{13}C NMR of 1D

Supplementary Fig. 63. NMR of 1E

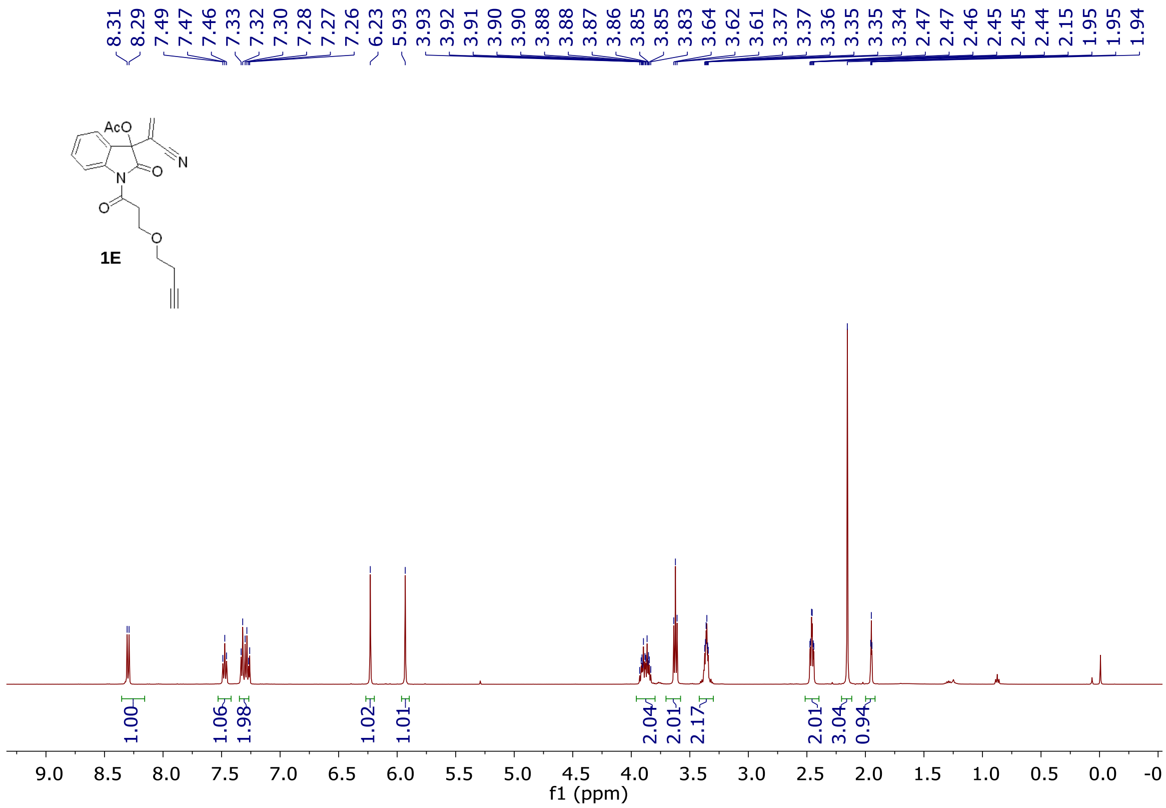

^{1}H NMR of 1E

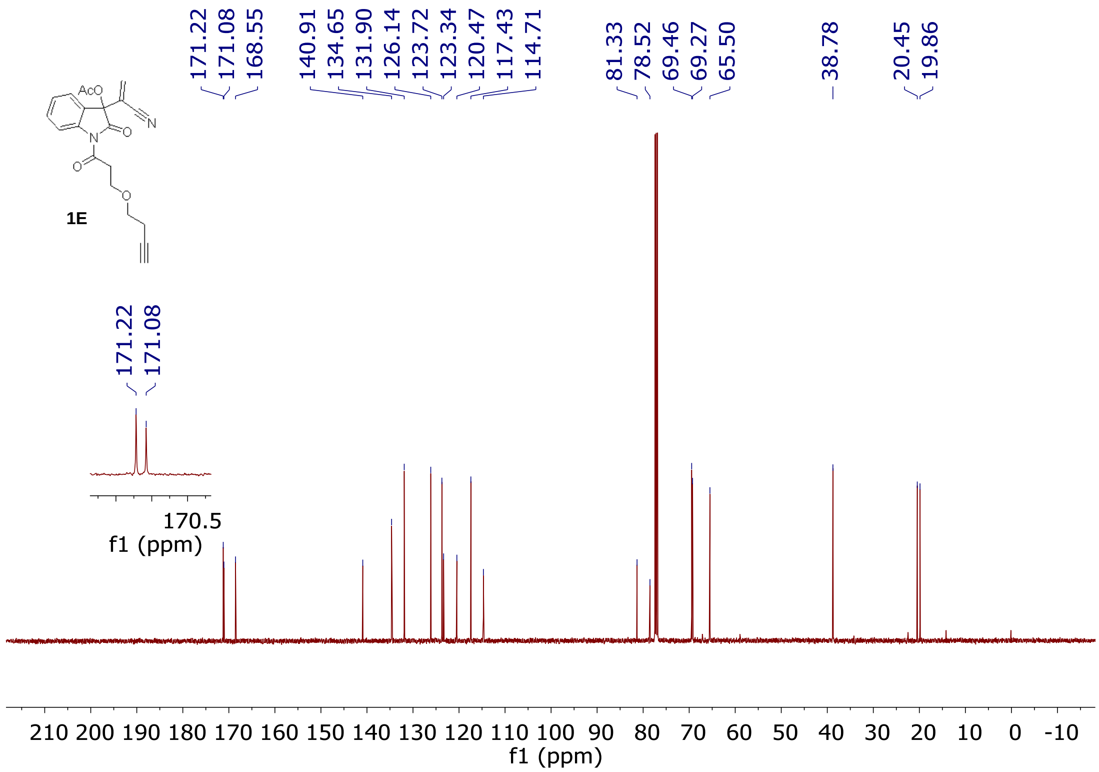

^{13}C NMR of 1E

Supplementary Fig. 64. NMR of DanN$_3$

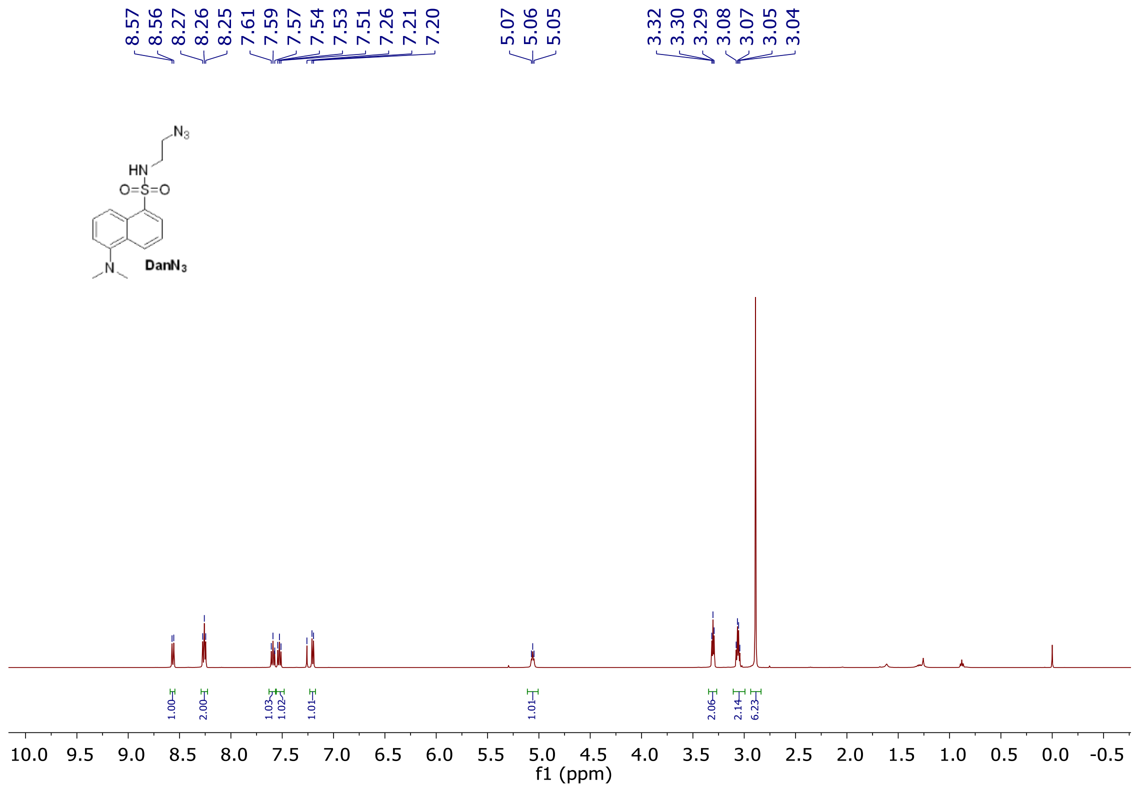

^{1}H NMR of DanN$_3$

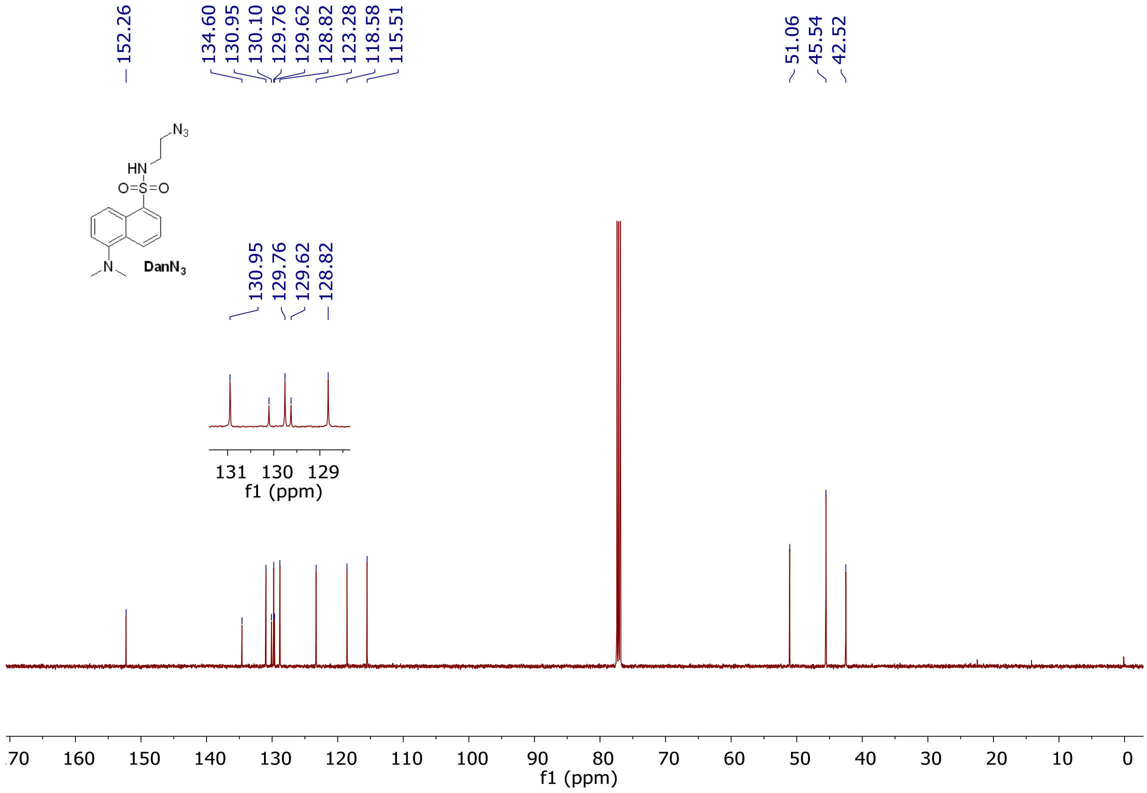

^{13}C NMR of DanN$_3$

Supplementary Fig. 65. NMR of 1G

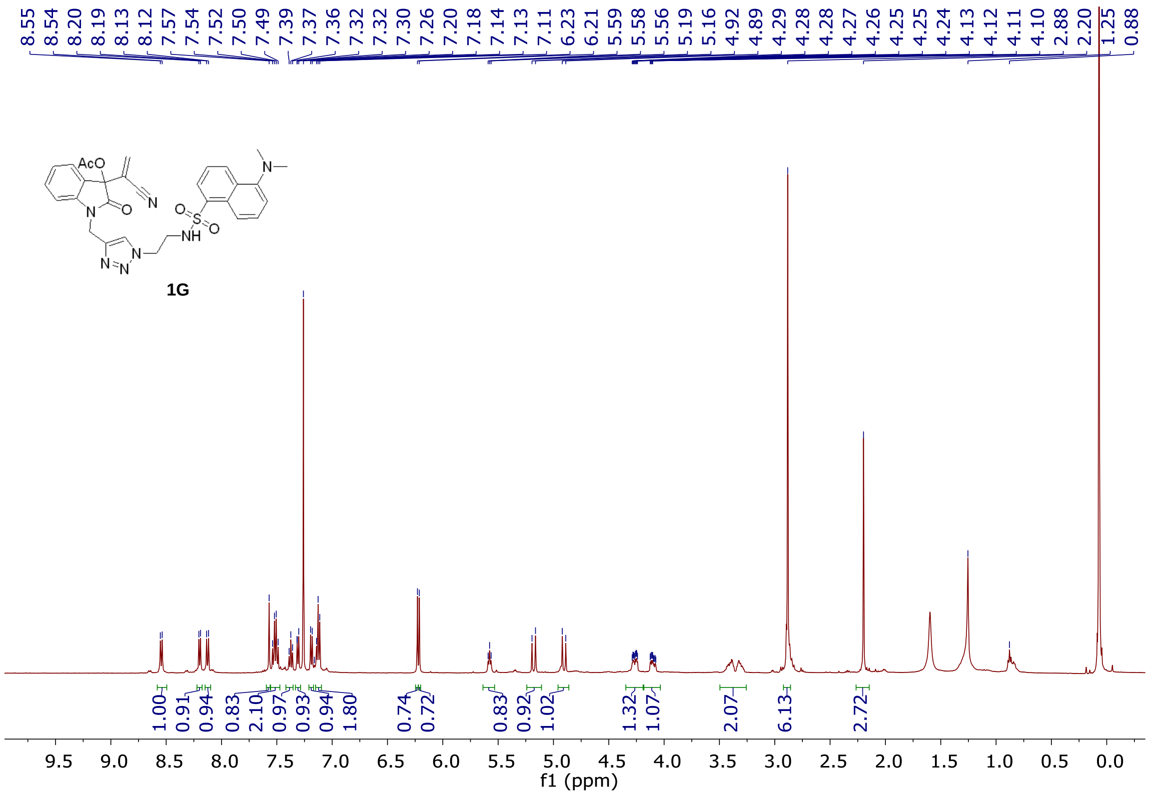

^{1}H NMR of 1G

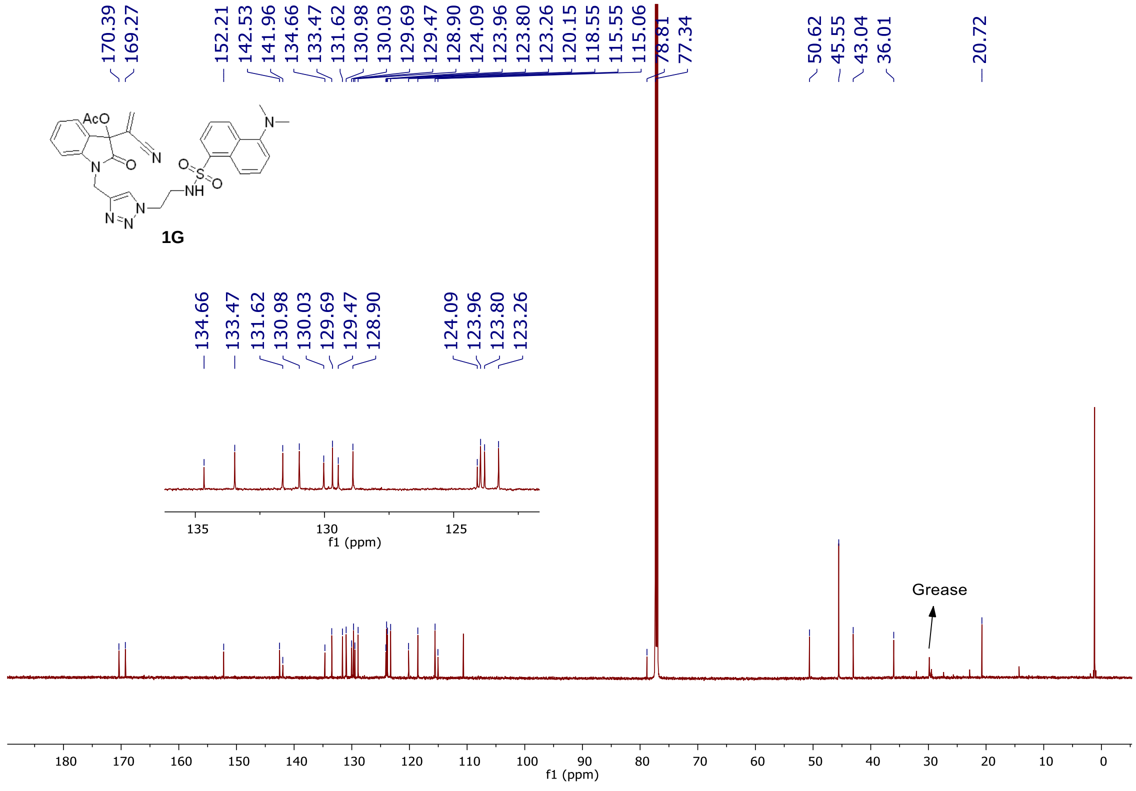

^{13}C NMR of 1G

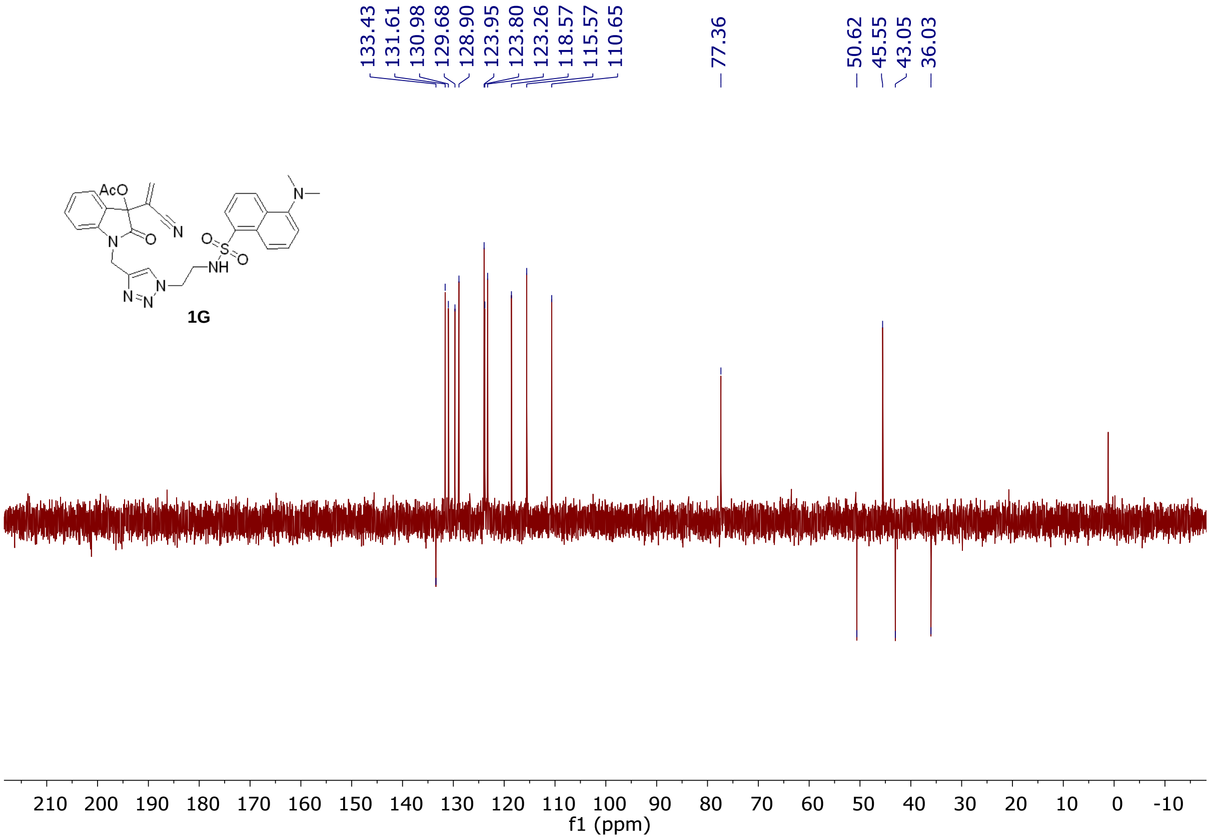

^{13}C DEPT-135 NMR of 1G

Supplementary Fig. 66. 1D and 2D-NMR of 2A

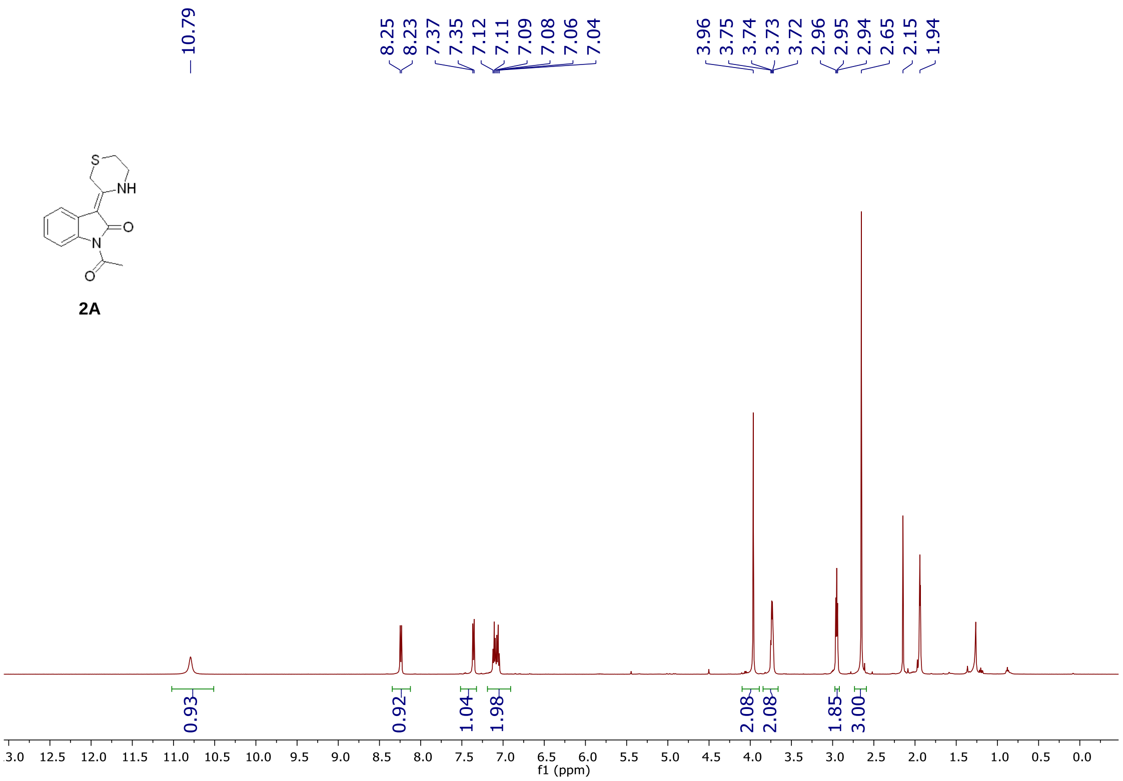

^{1}H NMR of 2A

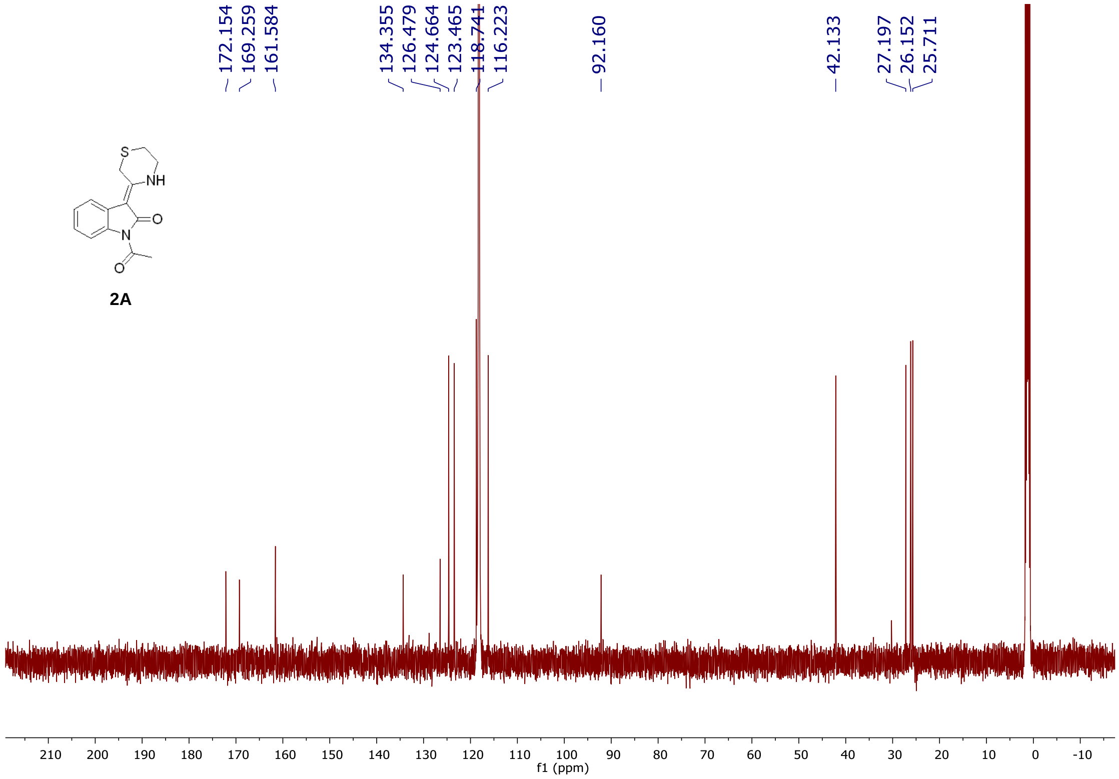

^{13}C NMR of 2A

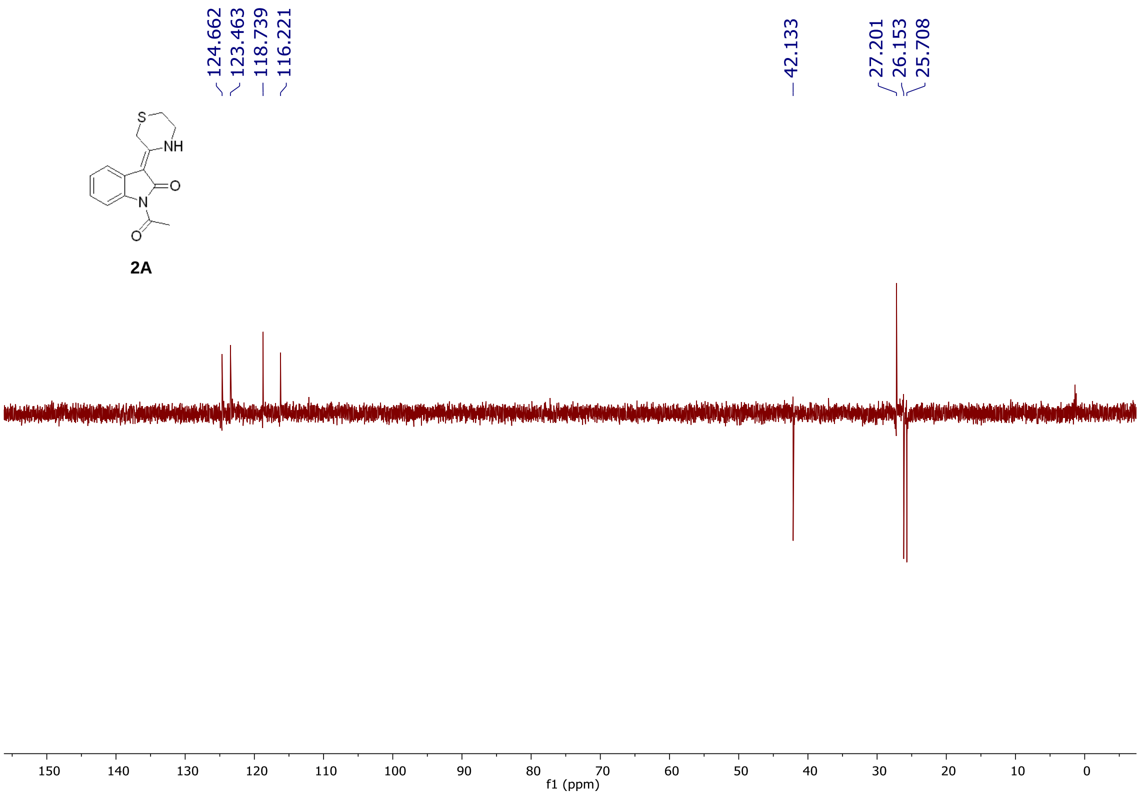

^{13}C DEPT-135 NMR of 2A

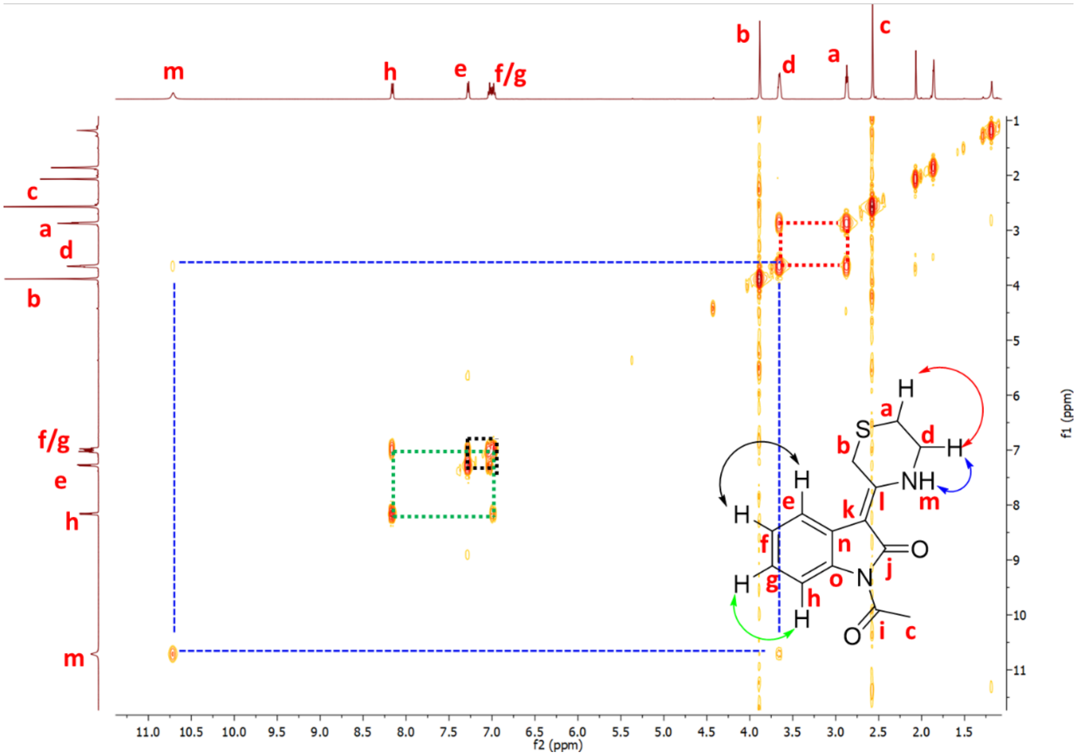

(^{1}H, ^{1}H) COSY of 2A

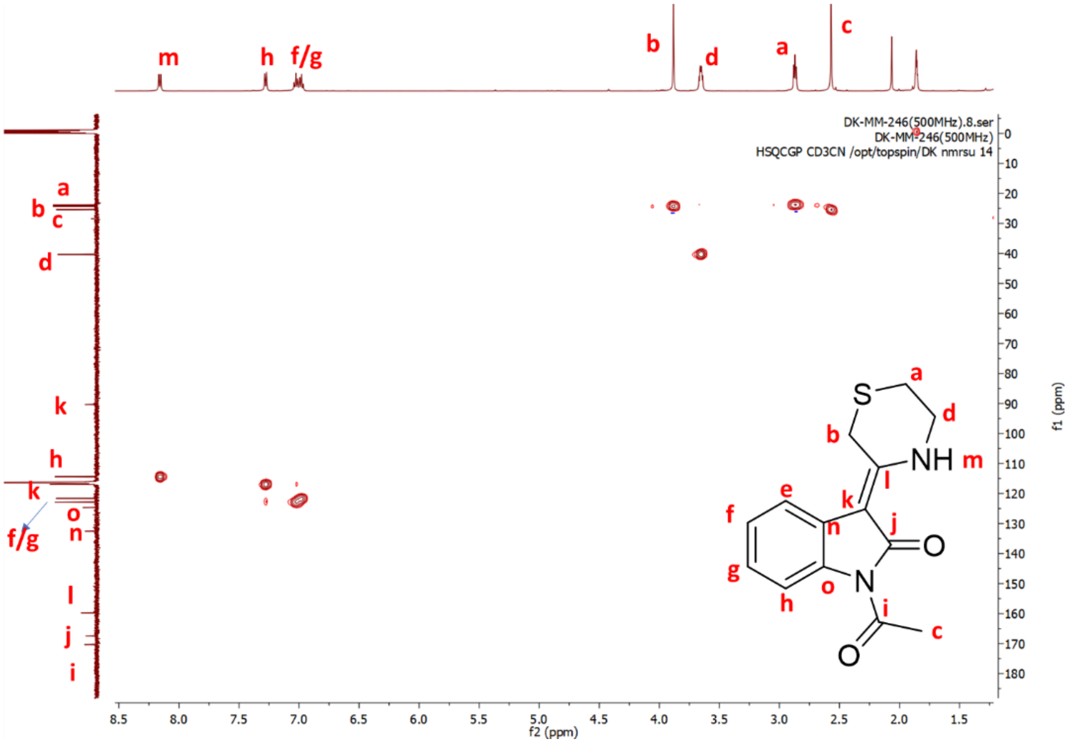

(^{1}H, ^{13}C) HSQC of 2A (δ_H 2.5–8.5)

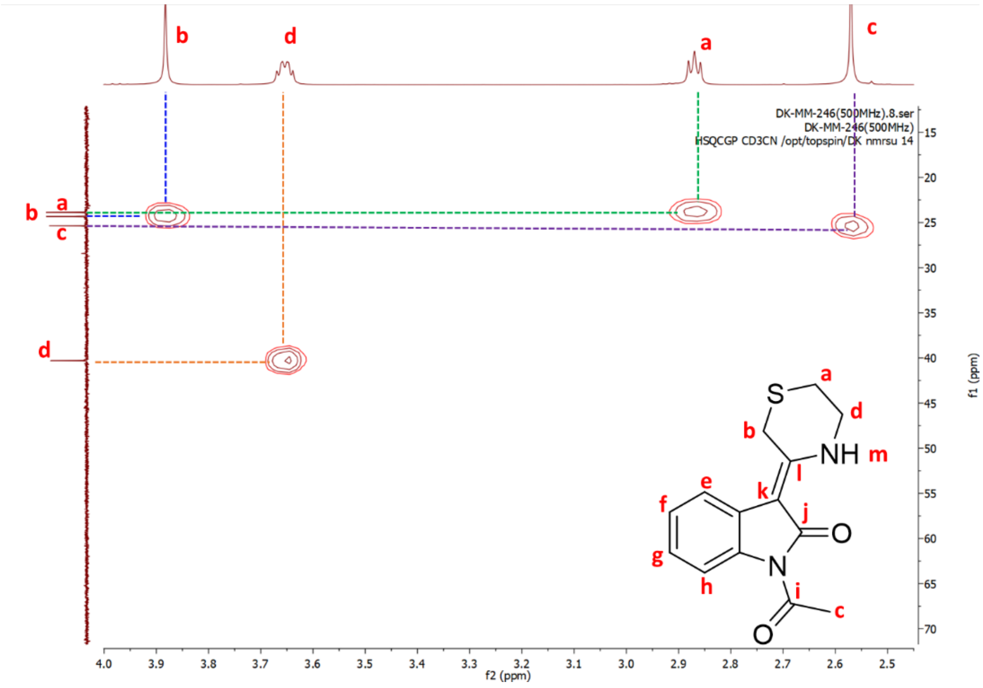

(^{1}H, ^{13}C) HSQC of 2A (δ_H 2.5–4.0)

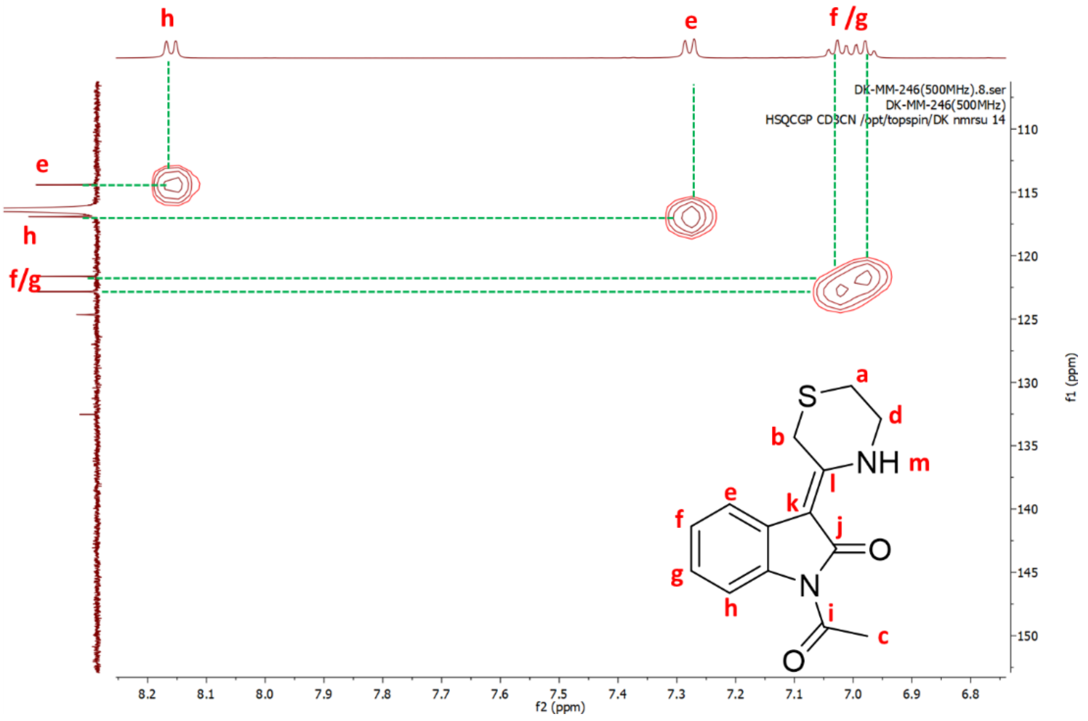

(^{1}H, ^{13}C) HSQC of 2A (δ_H 6.8–8.2)

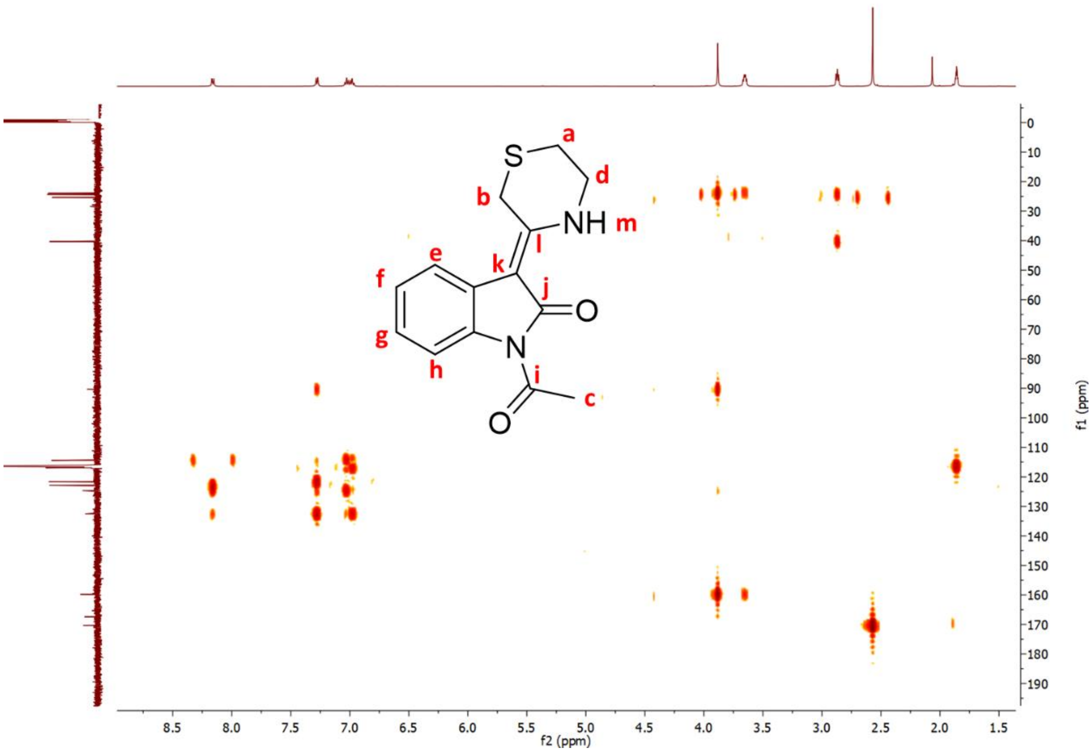

(^{1}H, ^{13}C) HMBC of 2A (δ_H 1.5–8.5)

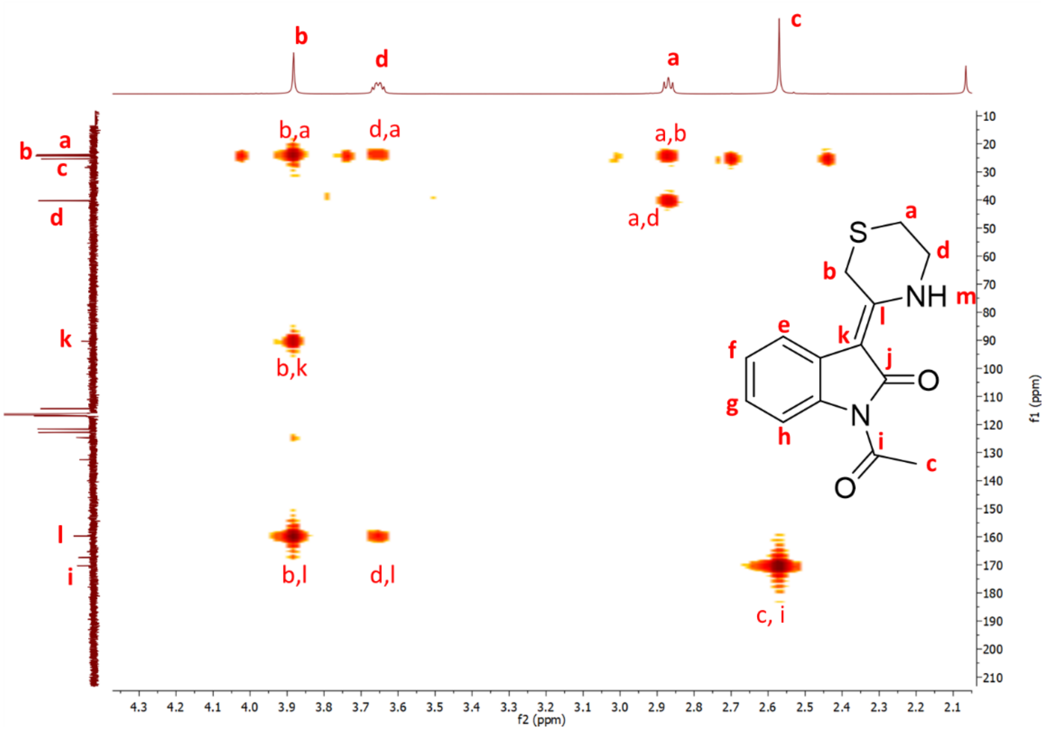

(^{1}H, ^{13}C) HMBC of 2A (δ_H 2.1–4.3)

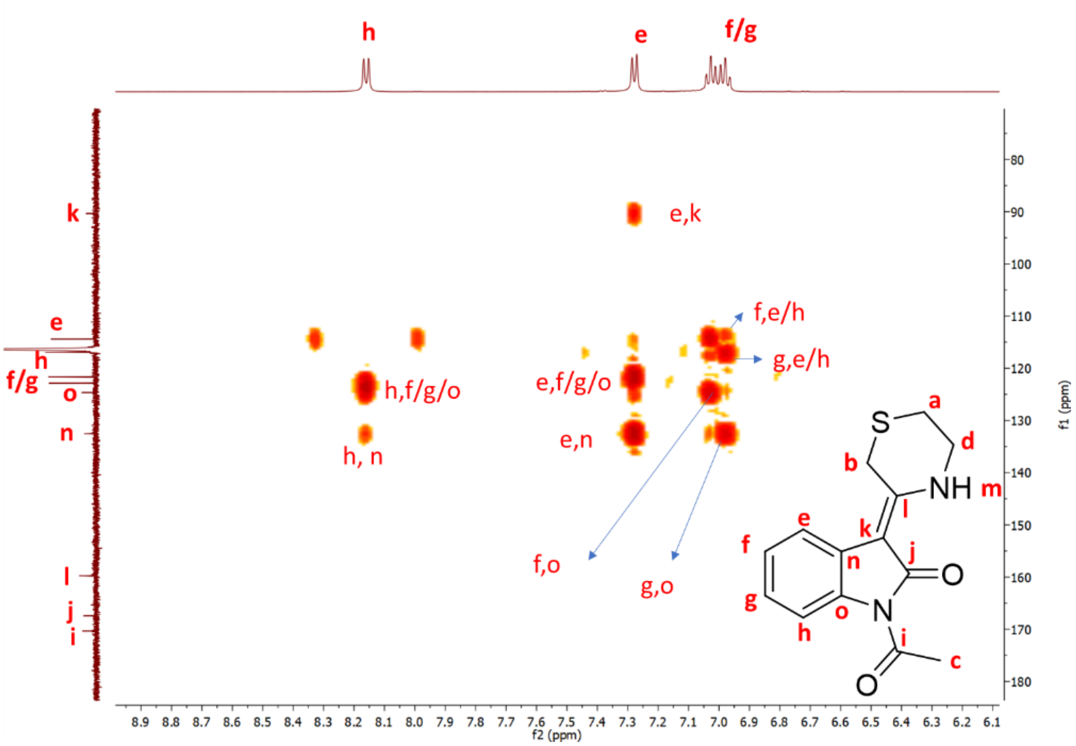

(^{1}H, ^{13}C) HMBC of 2A (δ_H 6.1–8.9)

Supplementary Fig. 67. 1D and 2D-NMR of 2B

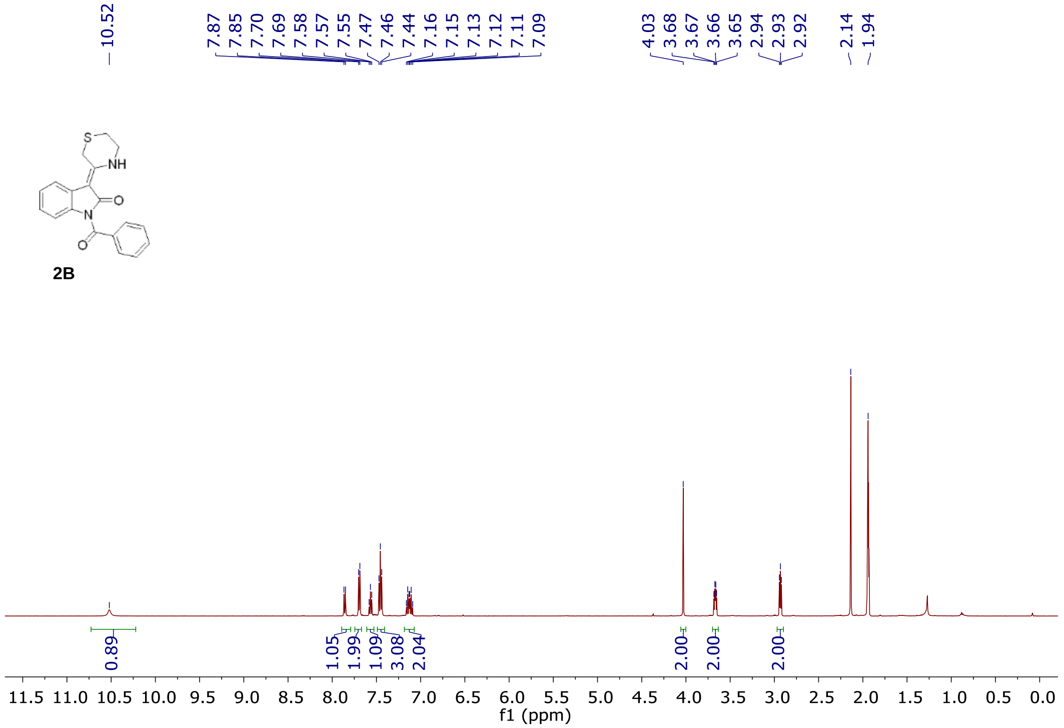

^{1}H NMR of 2B

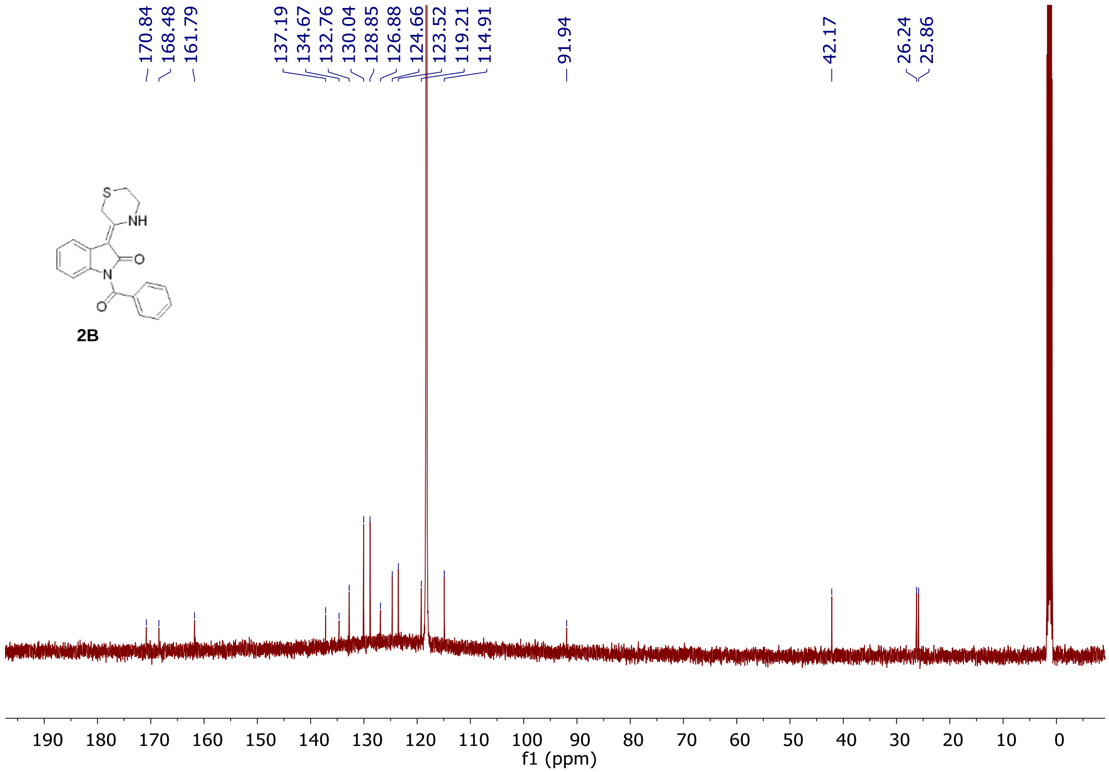

^{13}C NMR of 2B

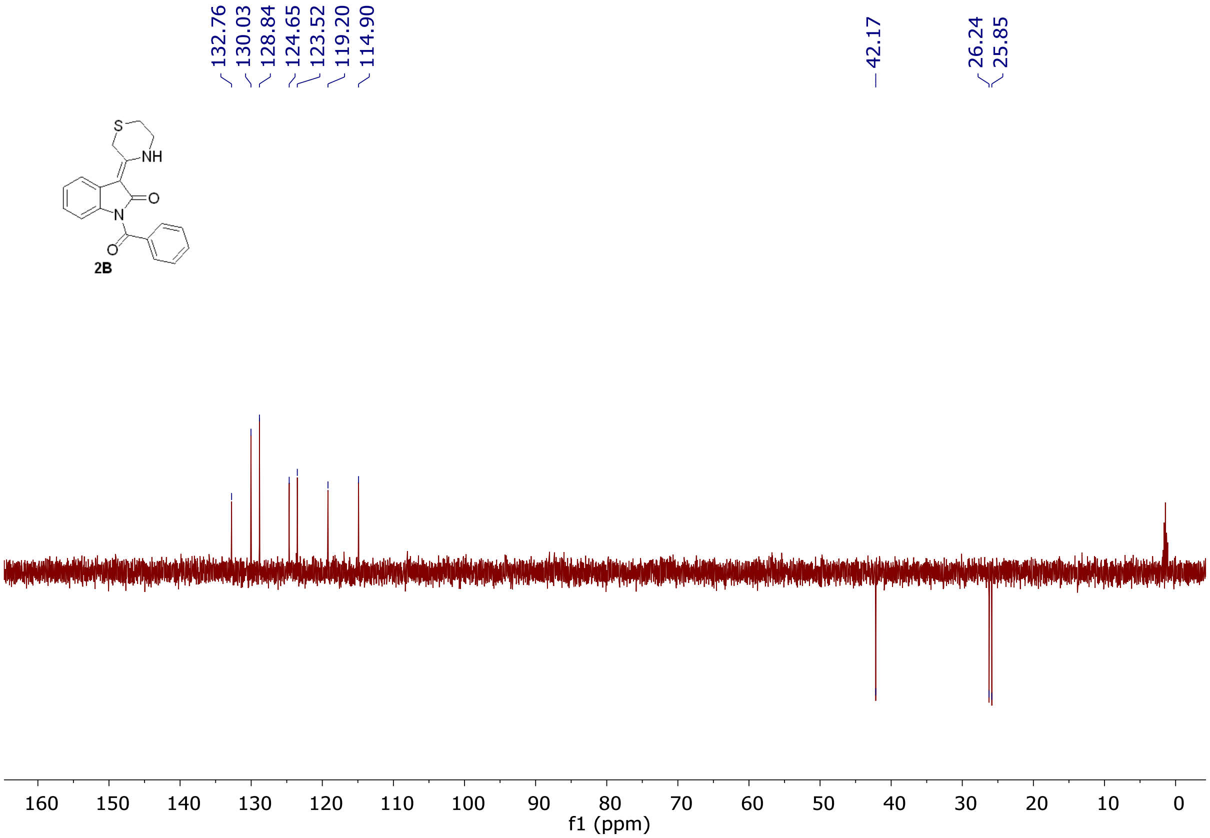

^{13}C DEPT-135 NMR of 2B

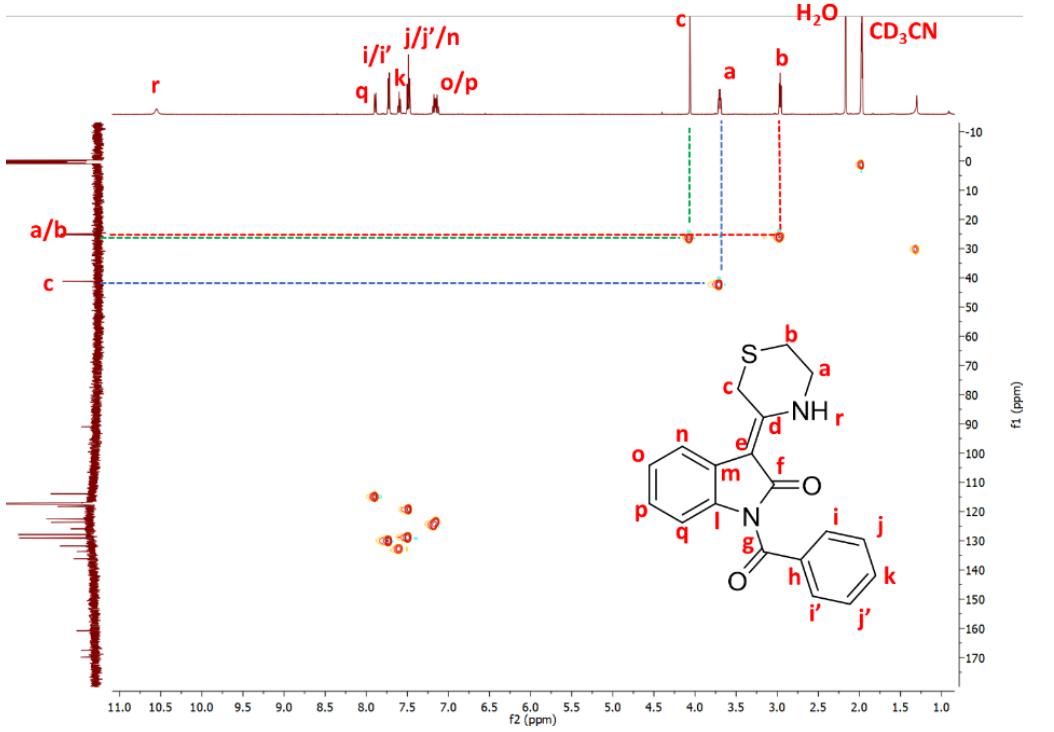

(^1H, ^{13}C) HSQC of 2B (δ_H 1.0–11.0)

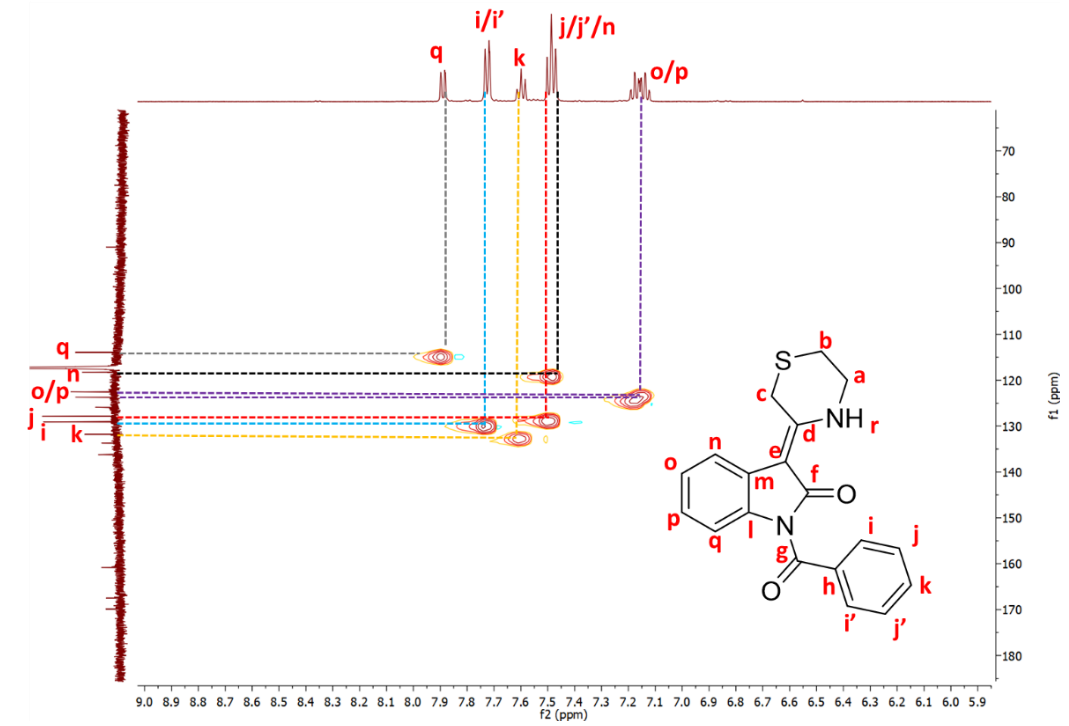

(^{1}H, ^{13}C) HSQC of 2B (δ_H 5.9–9.0)

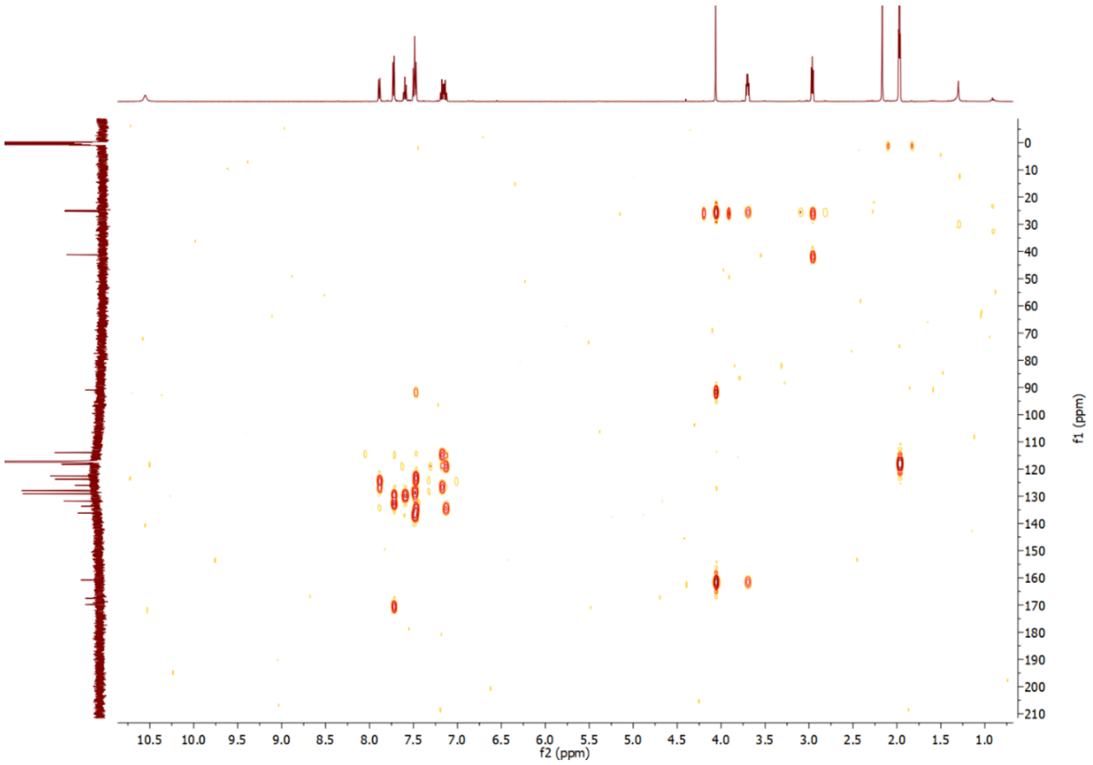

(^{1}H, ^{13}C) HMBC of 2B (δ_H 1.0–10.5)

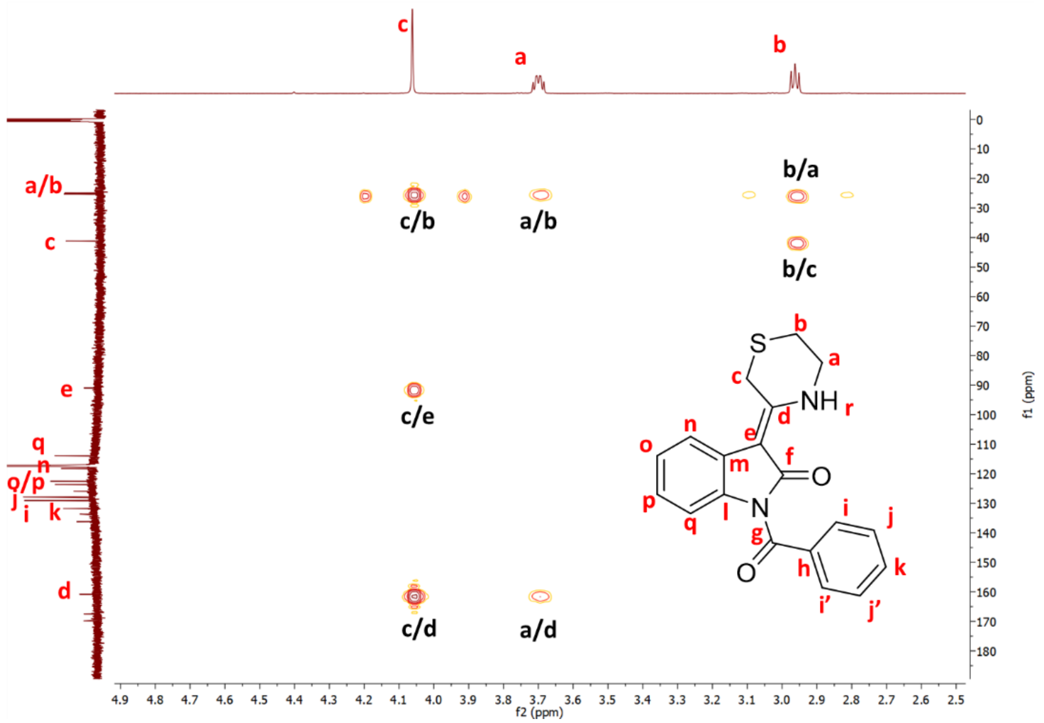

(^{1}H, ^{13}C) HMBC of 2B (δ_H 2.5–4.9)

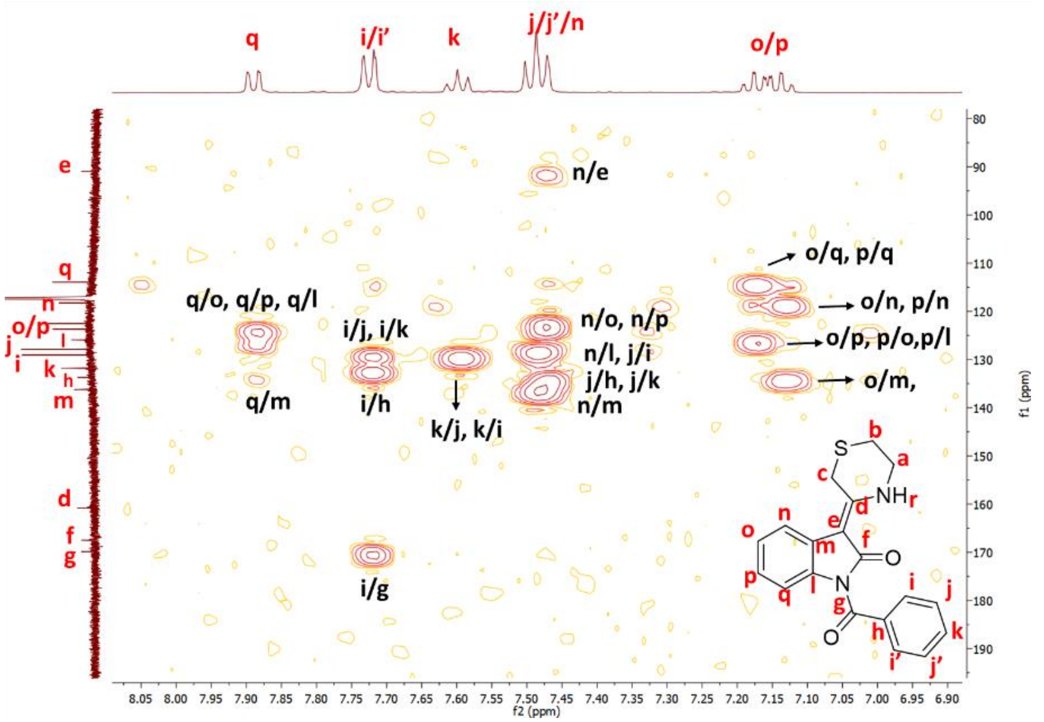

(^{1}H, ^{13}C) HMBC of 2B (δ_H 6.9–8.0)

Supplementary Fig. 68. 1D and 2D-NMR of 2C

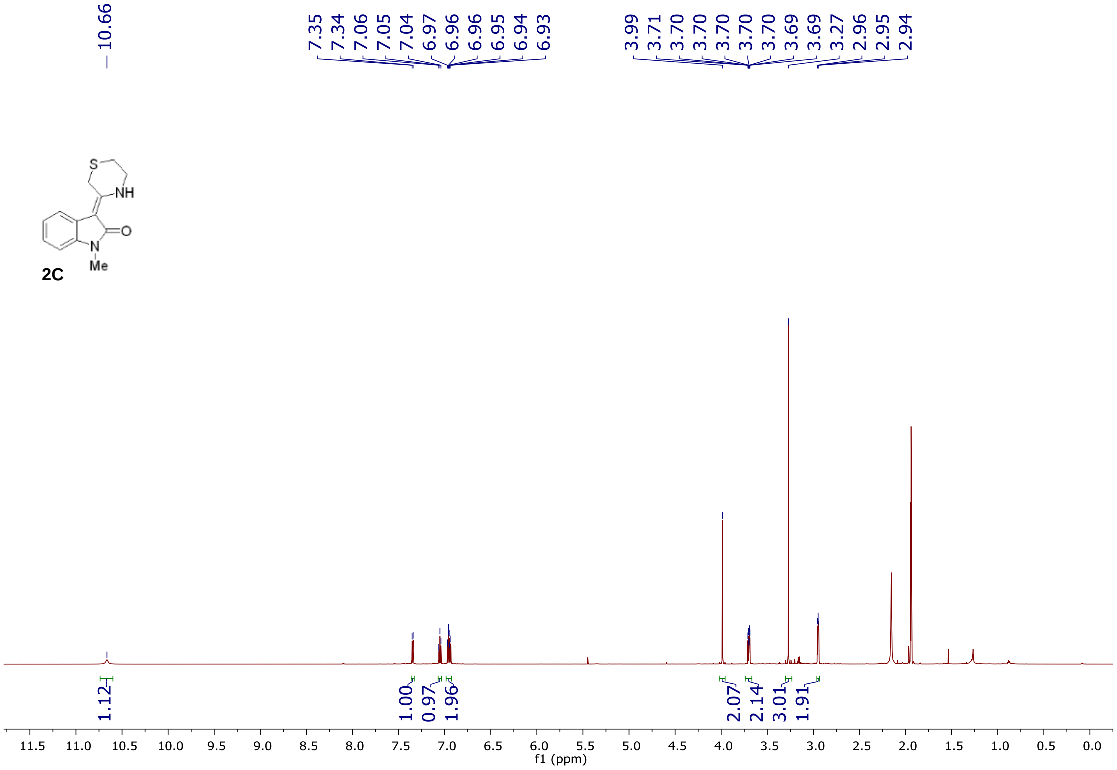

^{1}H NMR of 2C

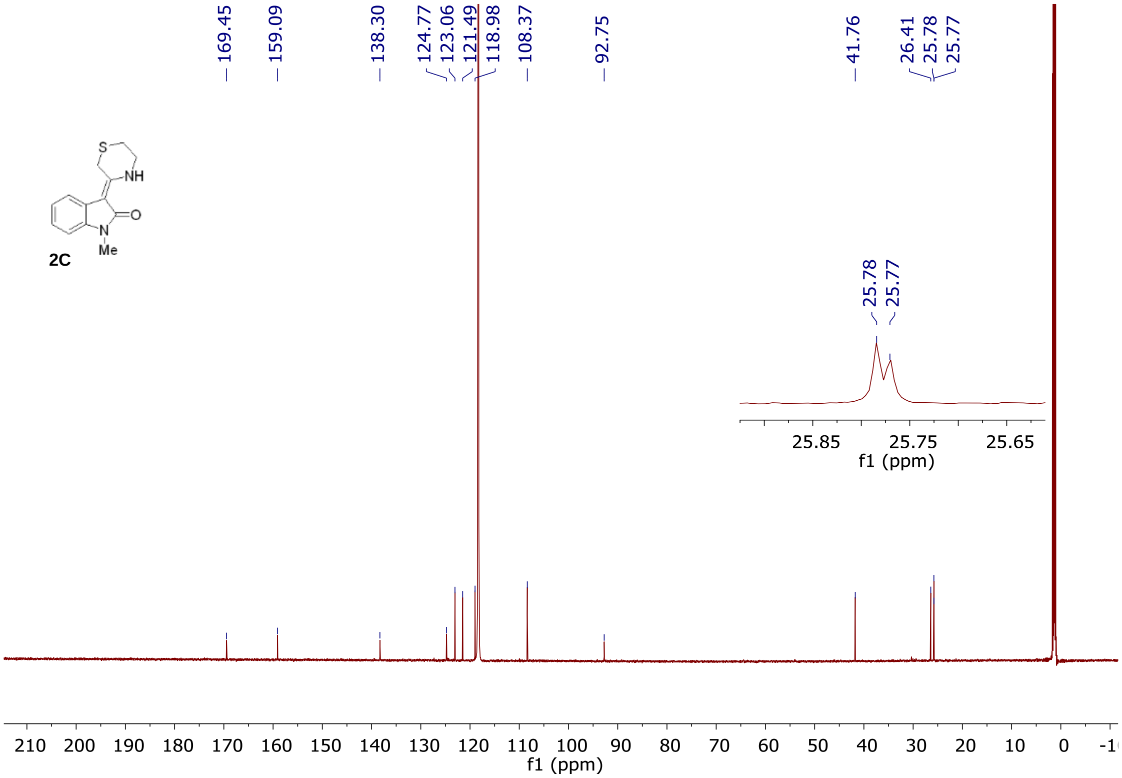

^{13}C NMR of 2C

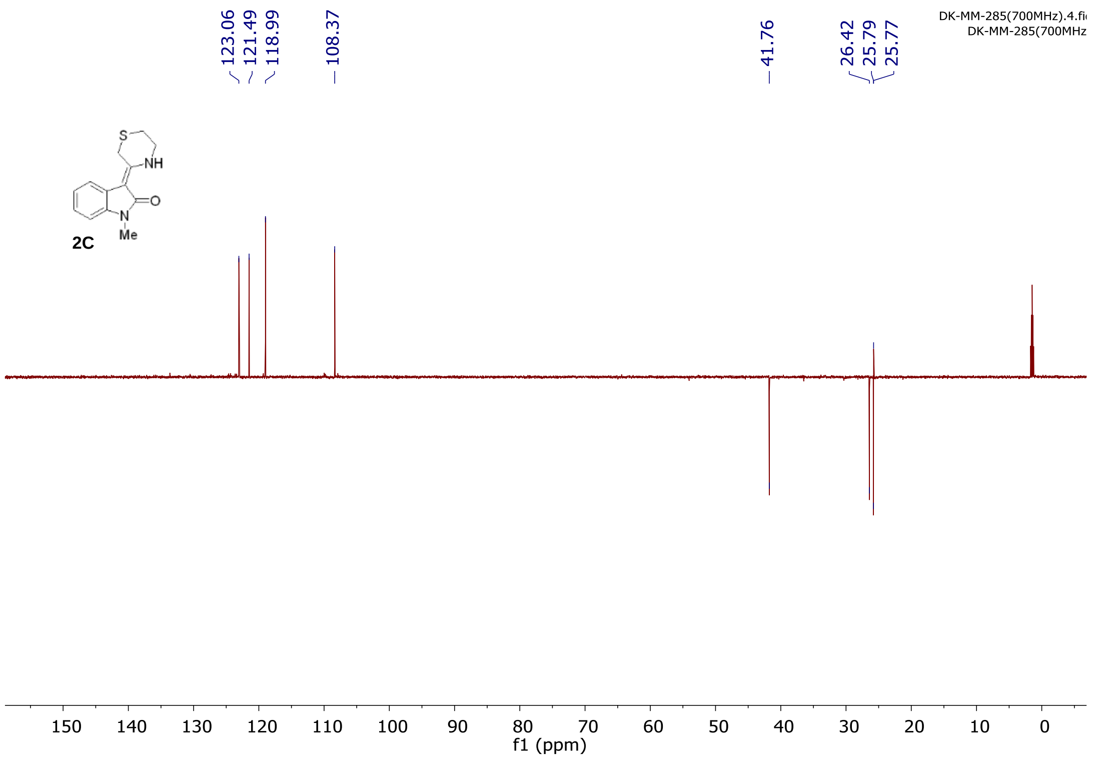

^{13}C DEPT-135 NMR of 2C

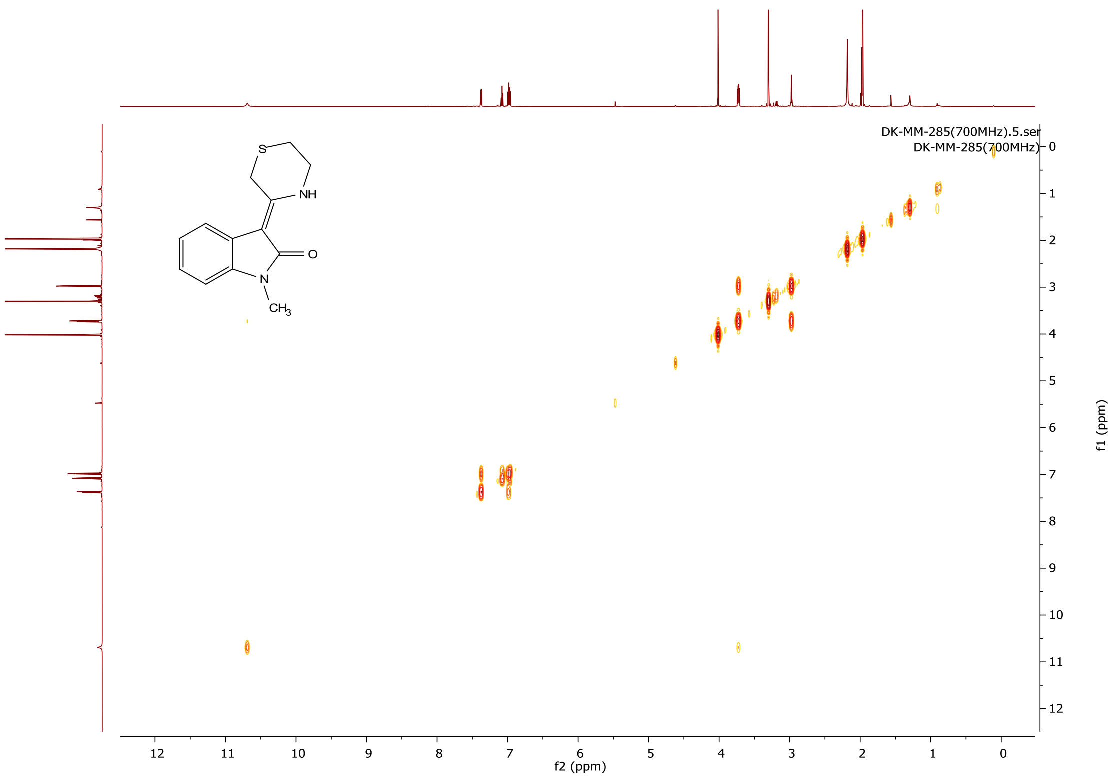

(^{1}H, ^{1}H) COSY of 2C

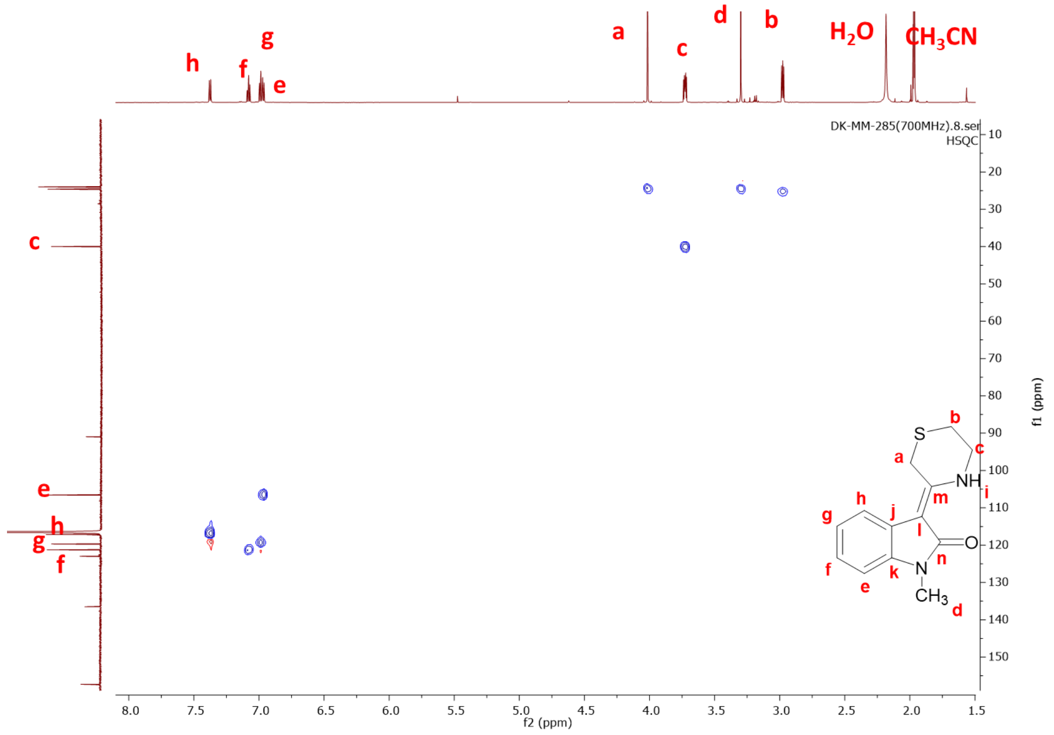

(^{1}H, ^{13}C) HSQC of 2C

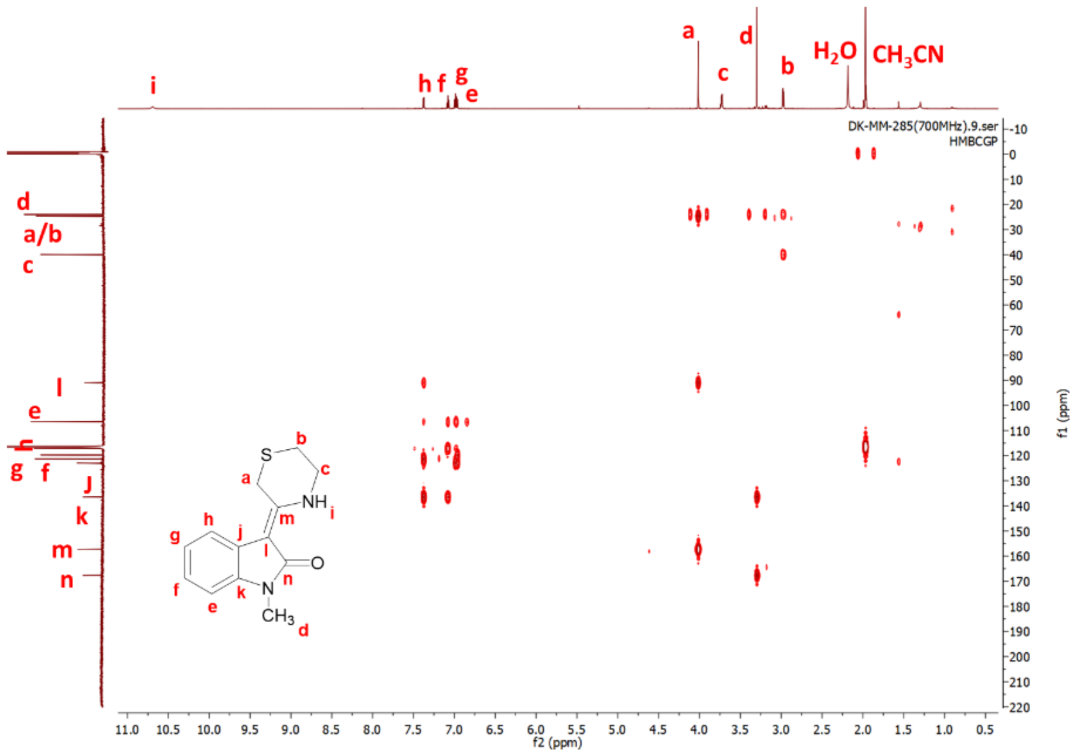

(^{1}H, ^{13}C) HMBC of 2C (δ_H 0.5–11.0)

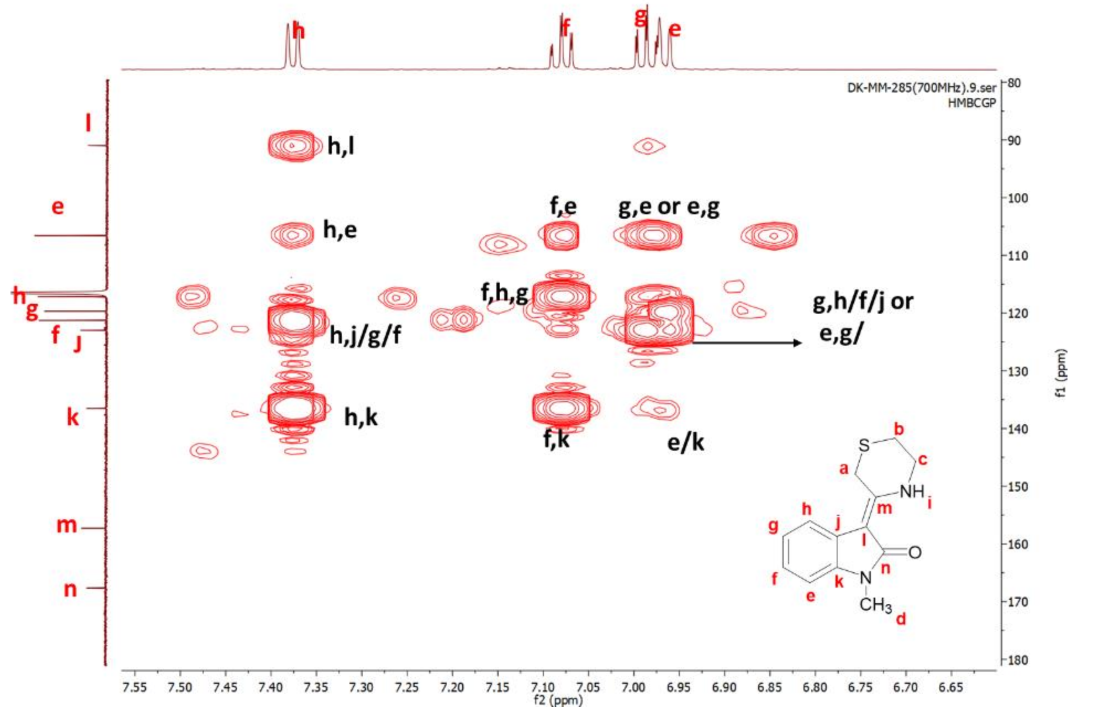

(^{1}H, ^{13}C) HMBC of 2C (δ_H 6.6–7.5)

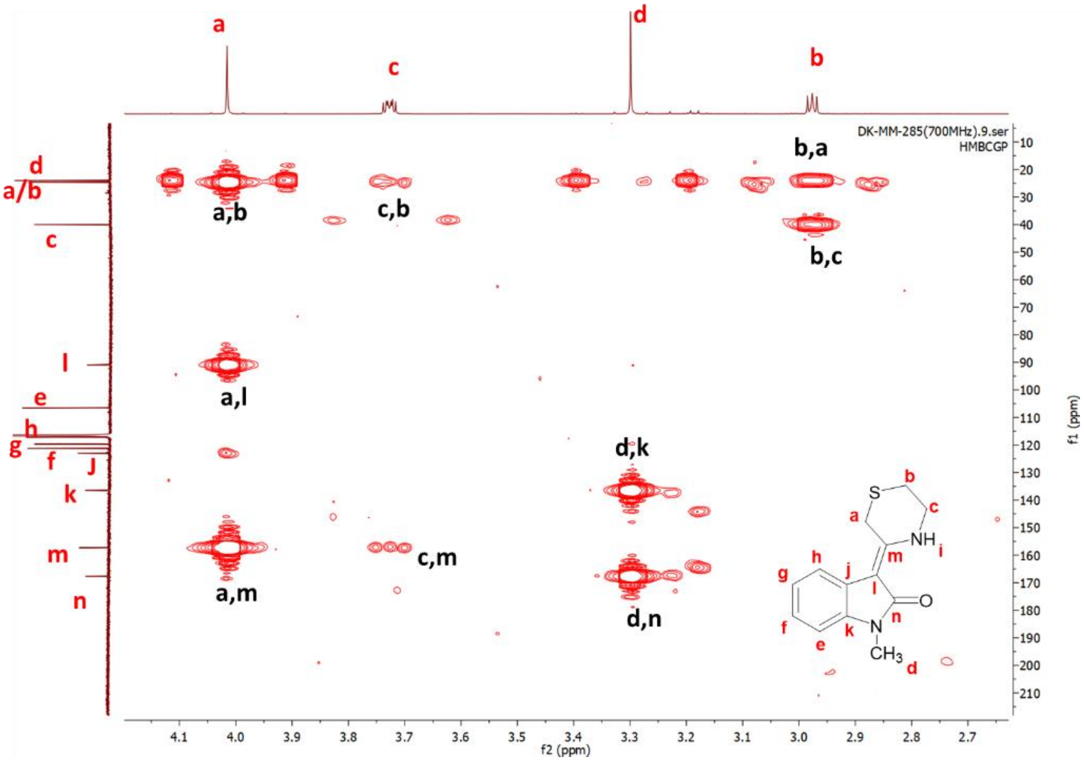

(^{1}H, ^{13}C) HMBC of 2C (δ_H 2.7–4.1)

Supplementary Fig. 69. NMR of S2A-Cys

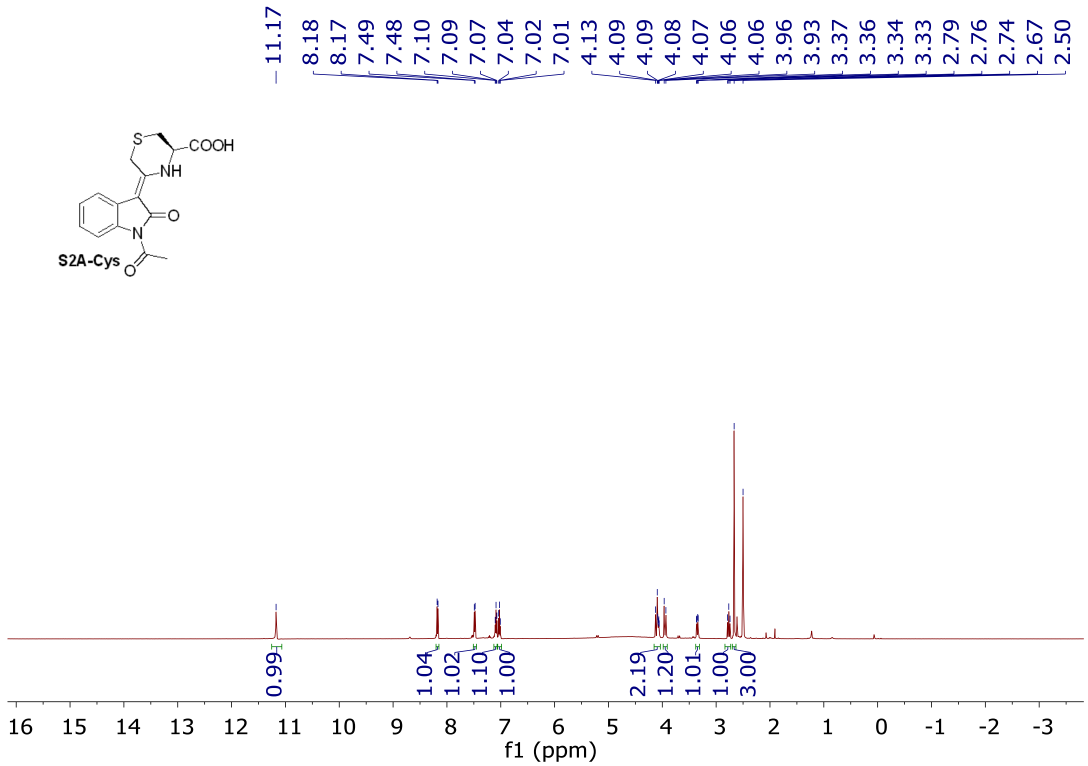

^{1}H NMR of S2A-Cys

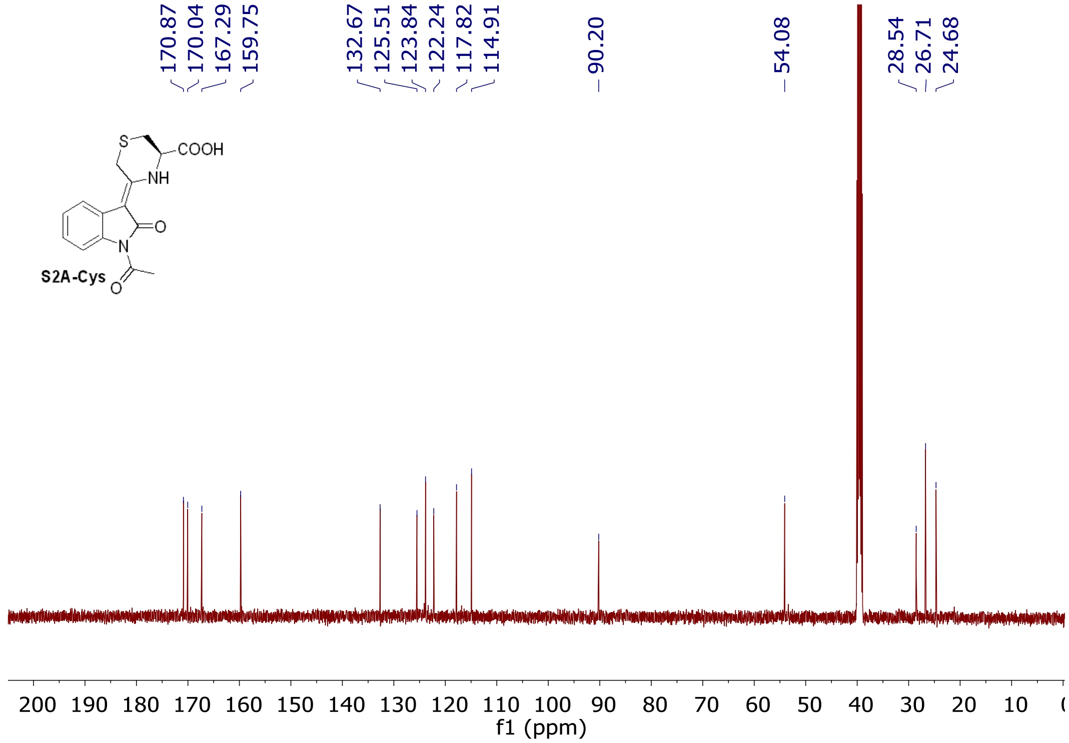

^{13}C NMR of S2A-Cys

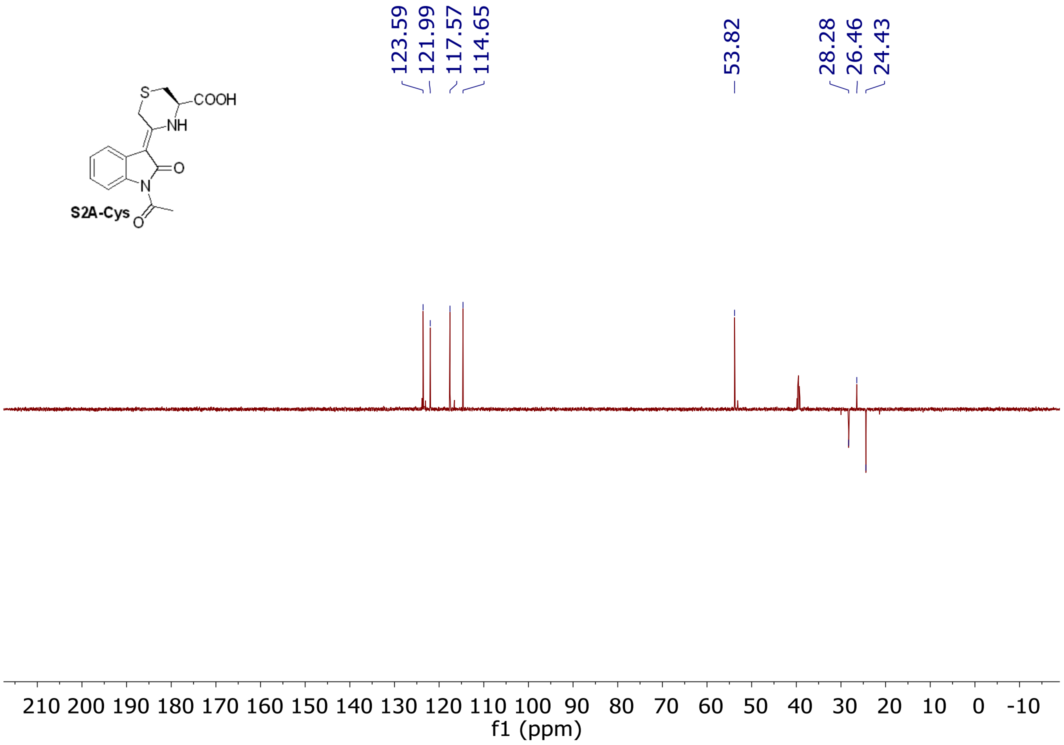

^{13}C DEPT-135 NMR of S2A-Cys

Supplementary Fig. 70. NMR of S7

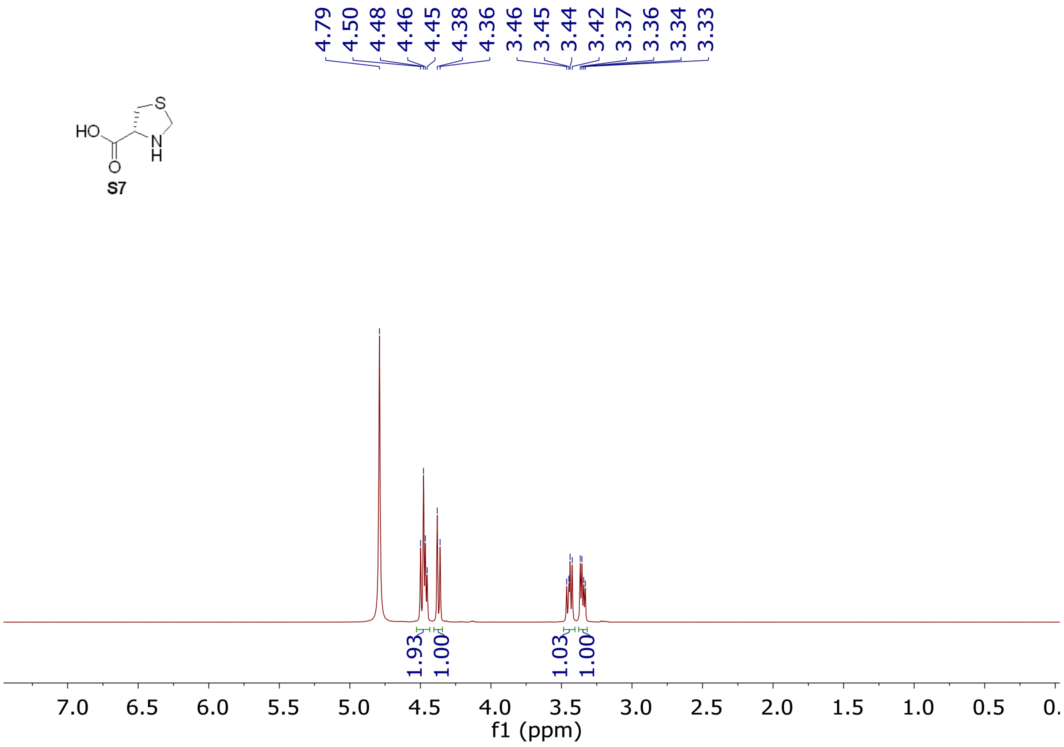

^{1}H NMR of S7

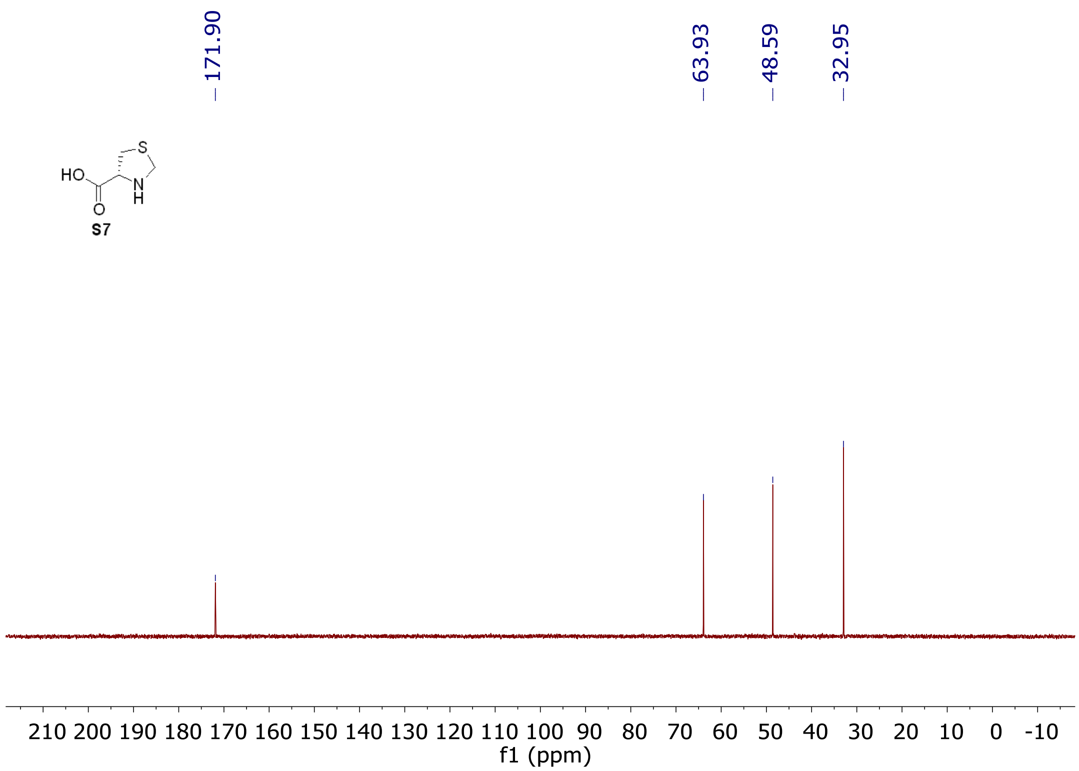

^{13}C NMR of S7

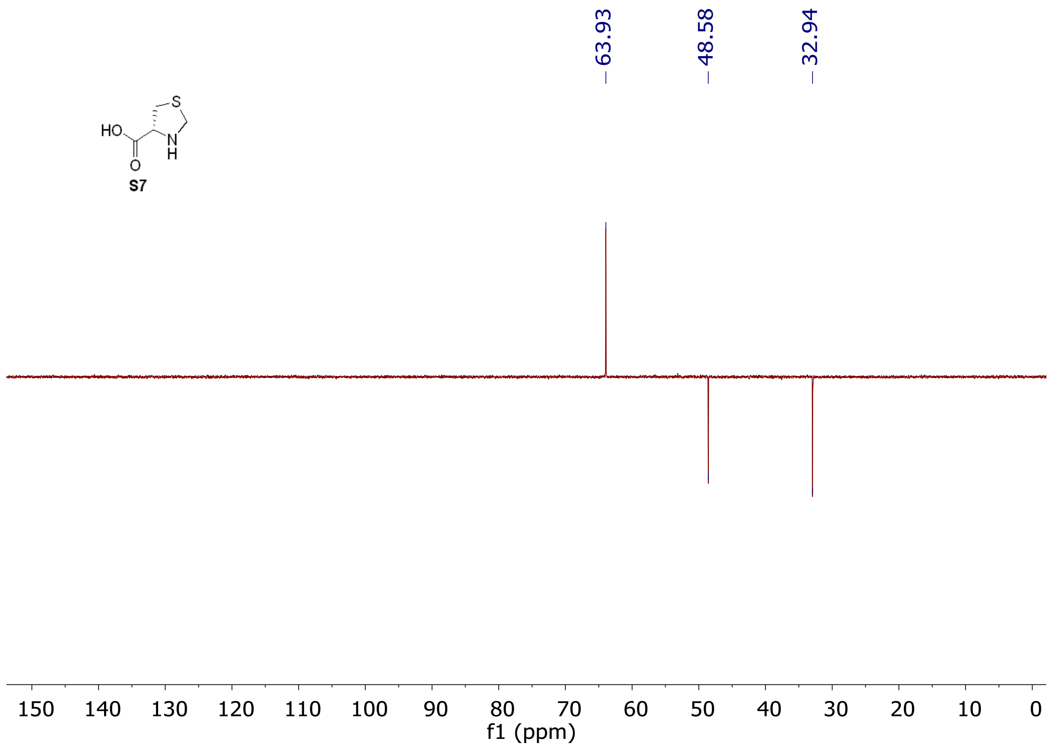

^{13}C DEPT-135 NMR of S7

Supplementary Fig. 71. NMR of *N*-boc thiazolidine acid

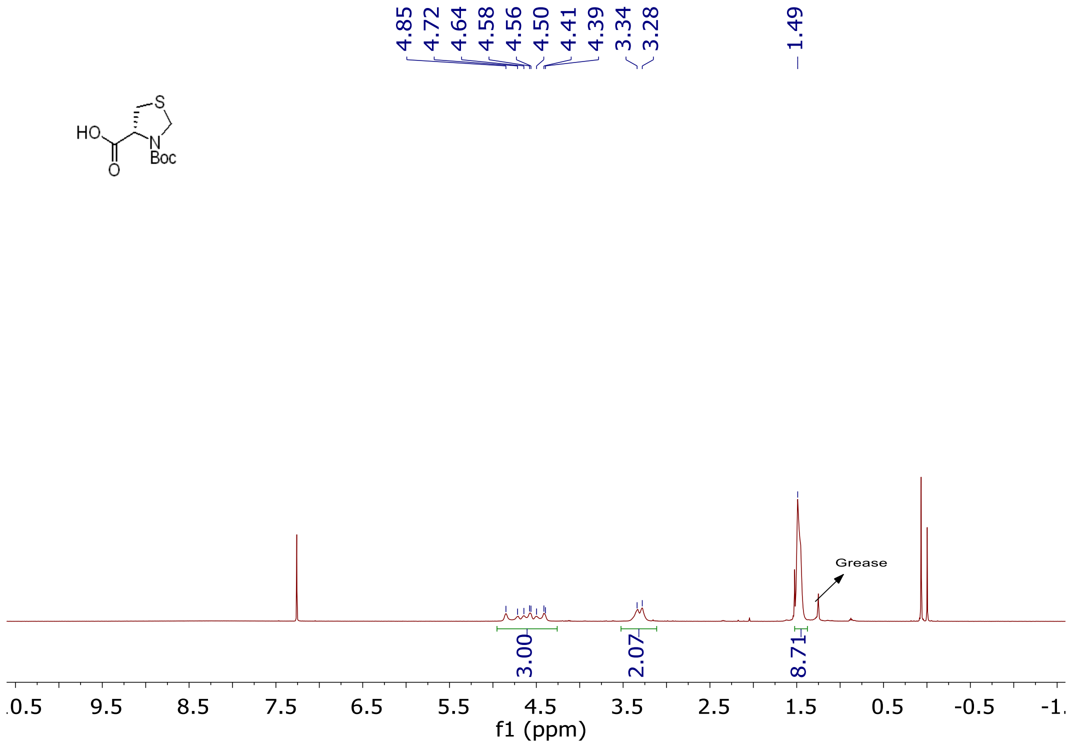

^{1}H NMR of *N*-boc thiazolidine acid

Supplementary Fig. 72. NMR of S8

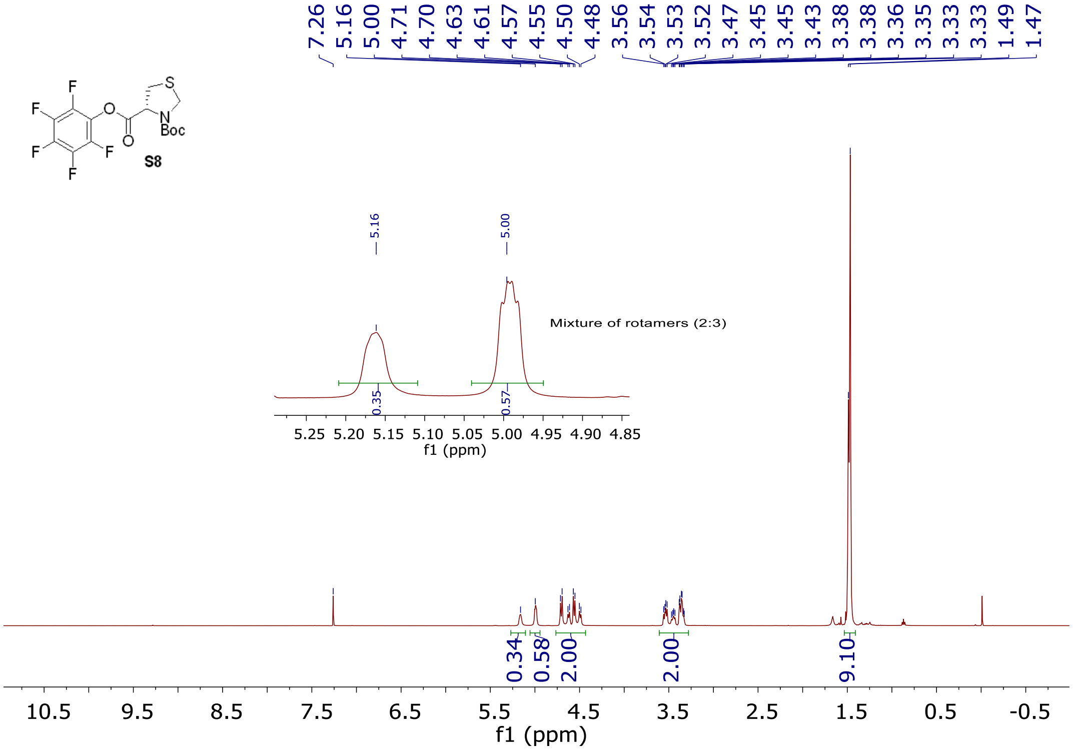

^{1}H NMR of S8

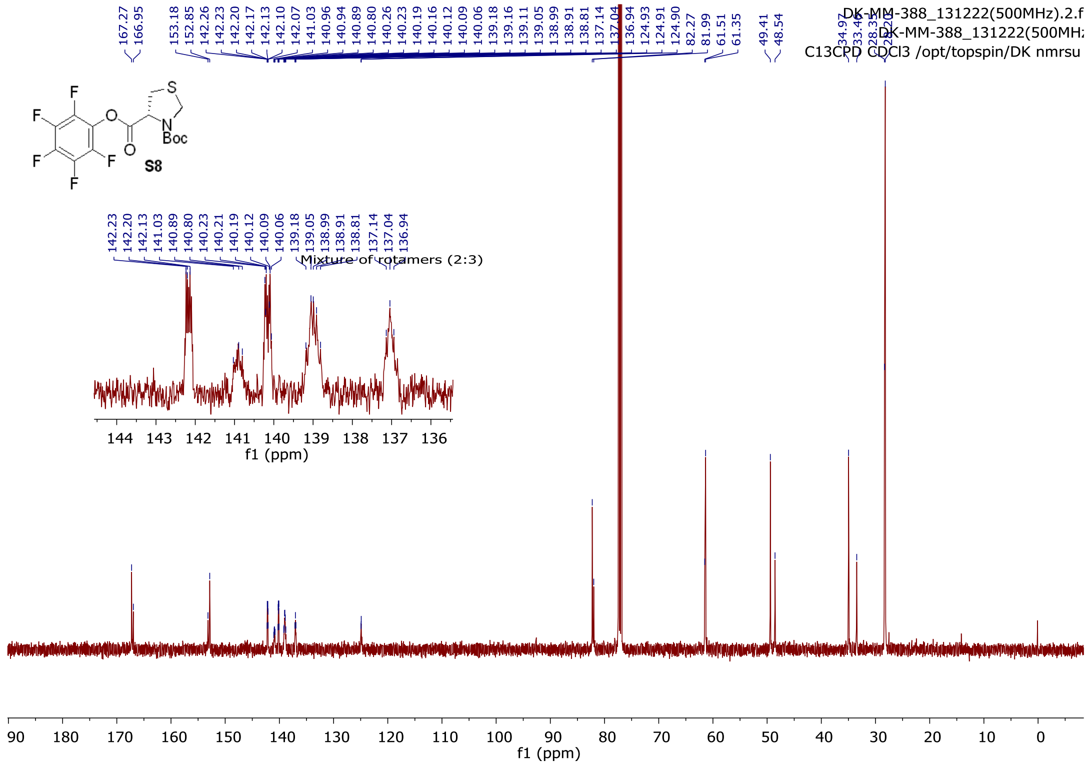

^{13}C NMR of S8

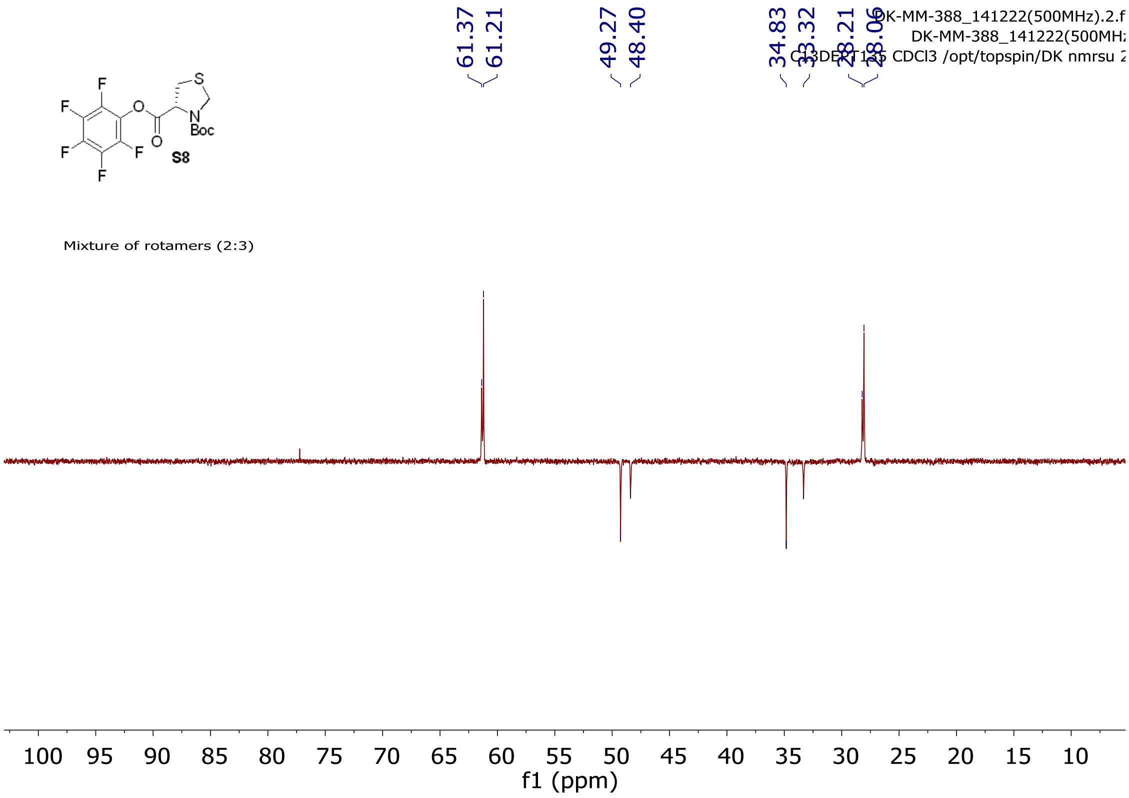

^{13}C DEPT-135 of NMR of S8

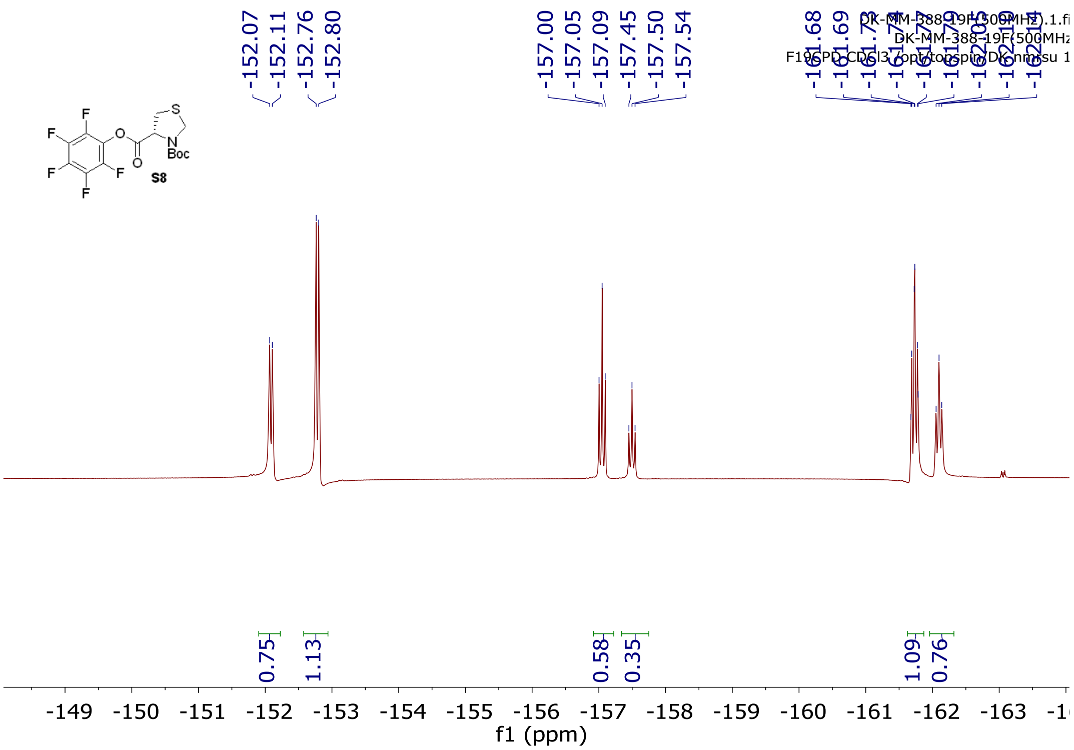

^{19}F NMR of S8

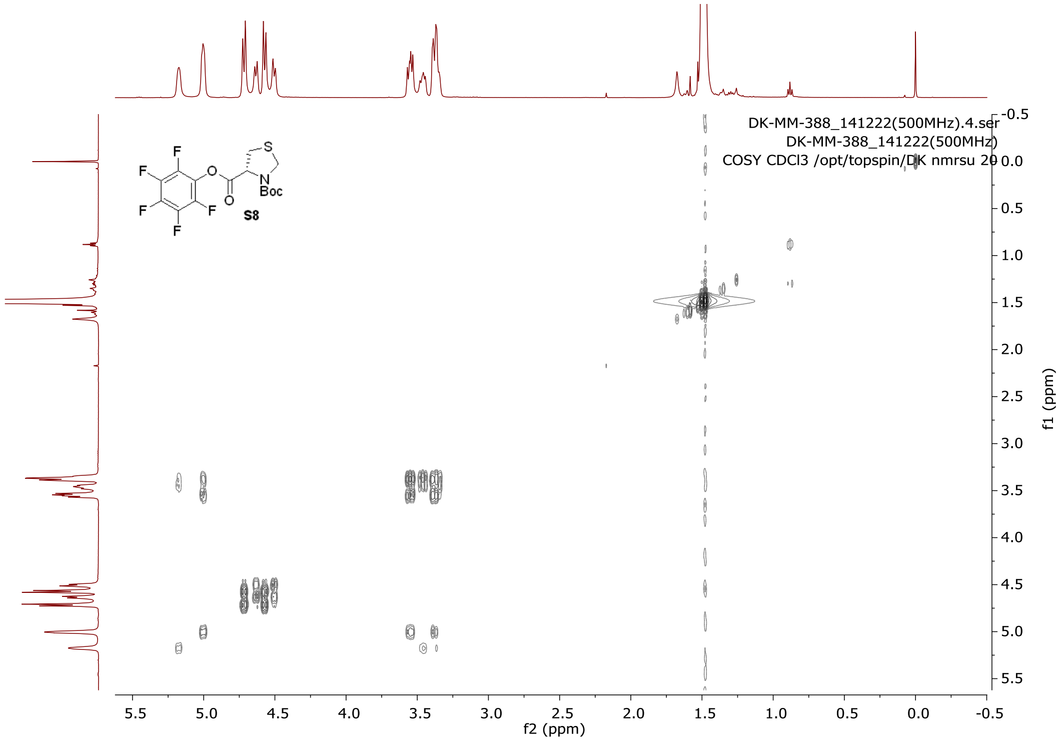

(^{1}H, ^{1}H) COSY of S8

Supplementary Fig. 73. ^{1}H-NMR of S9

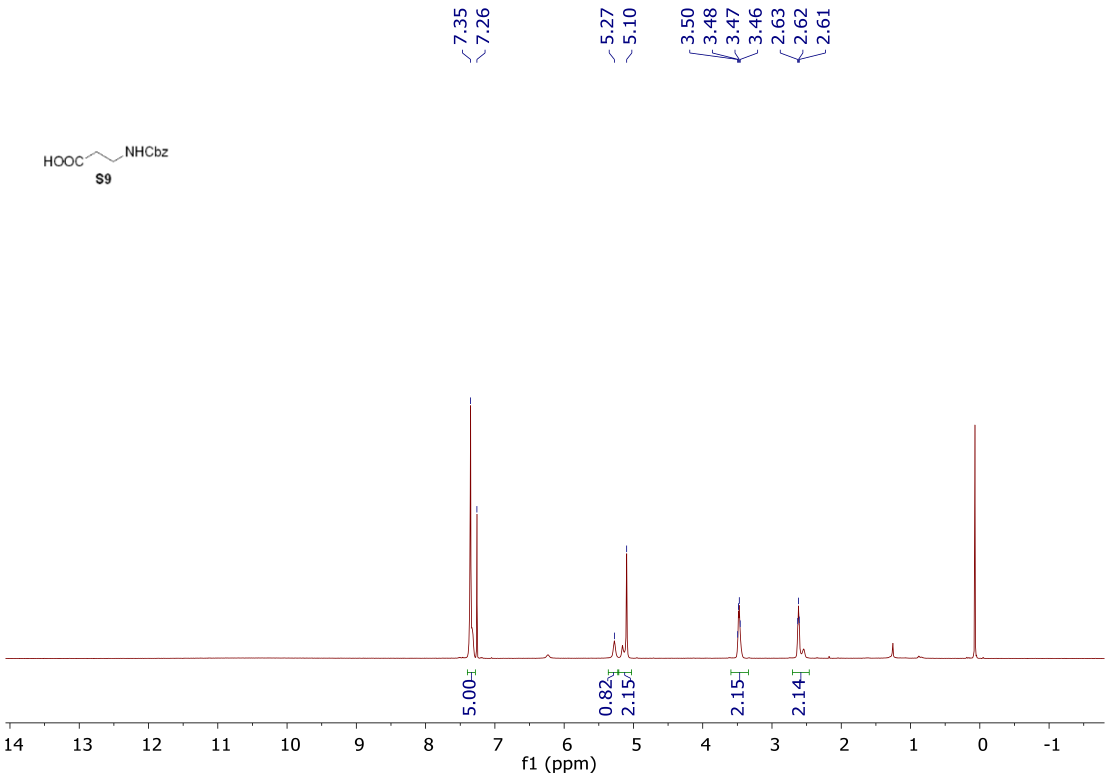

Supplementary Fig. 74. ^{1}H-NMR of S10

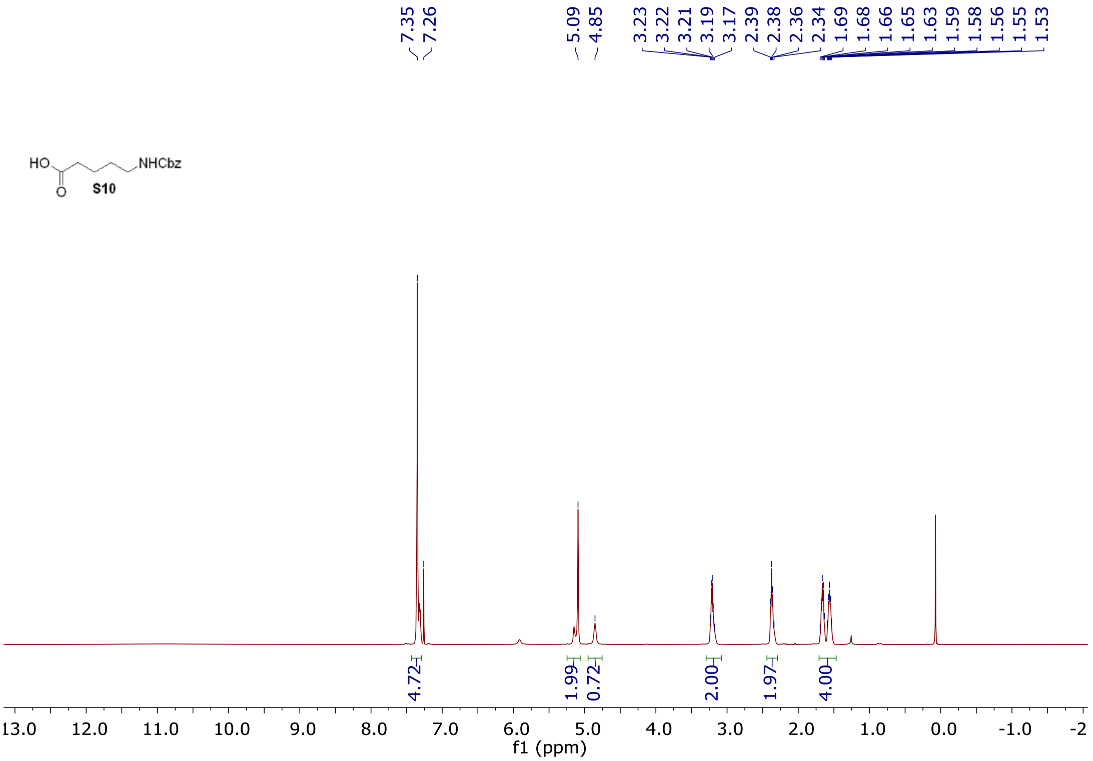

Supplementary Fig. 75. ^{1}H-NMR of S11

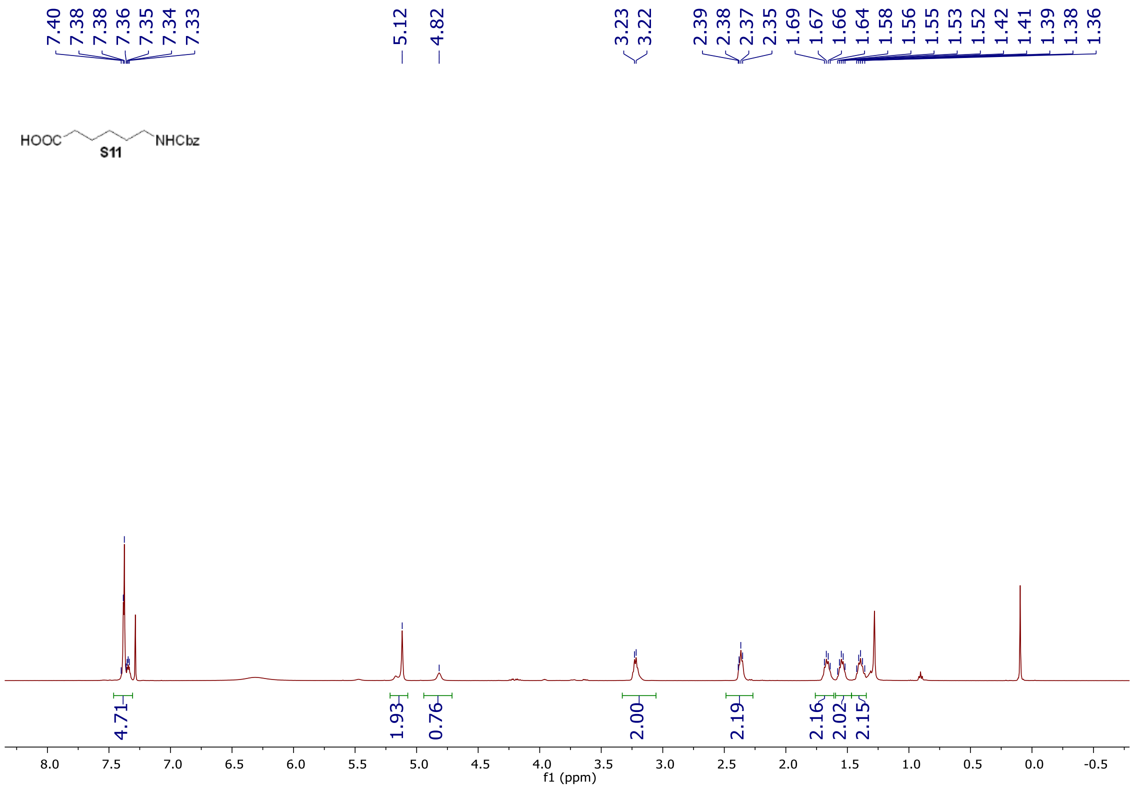

Supplementary Fig. 76. NMR of S12

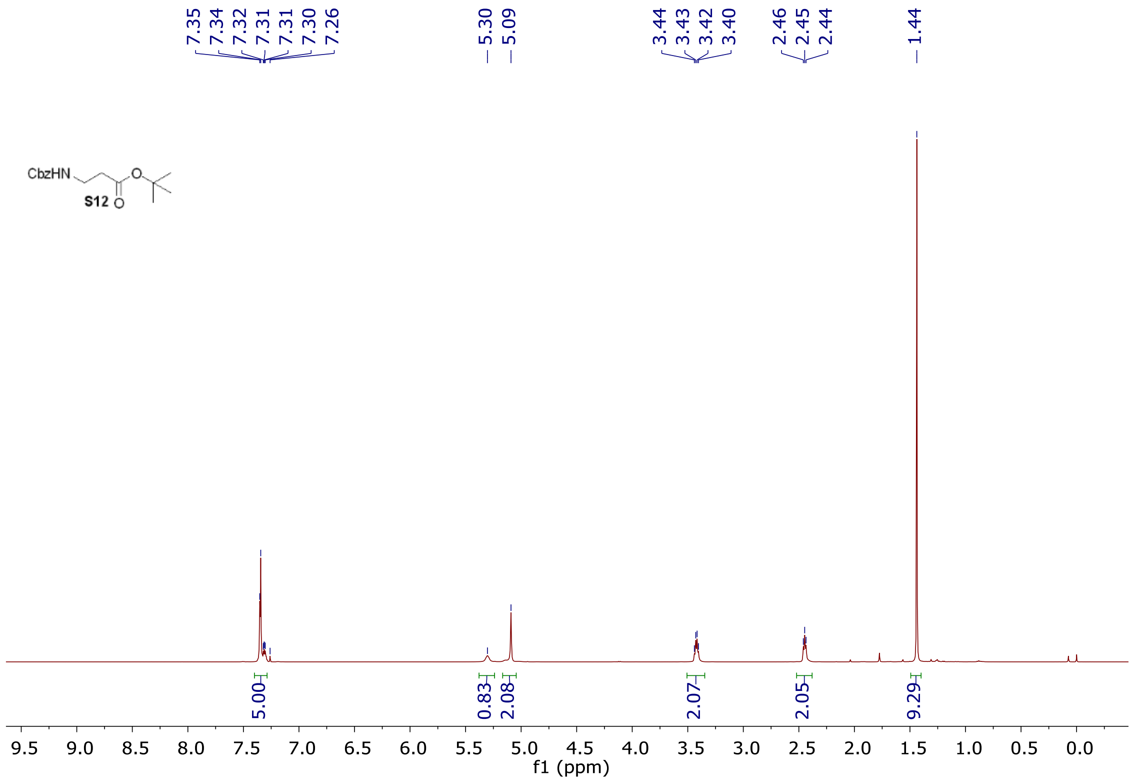

^{1}H NMR of S12

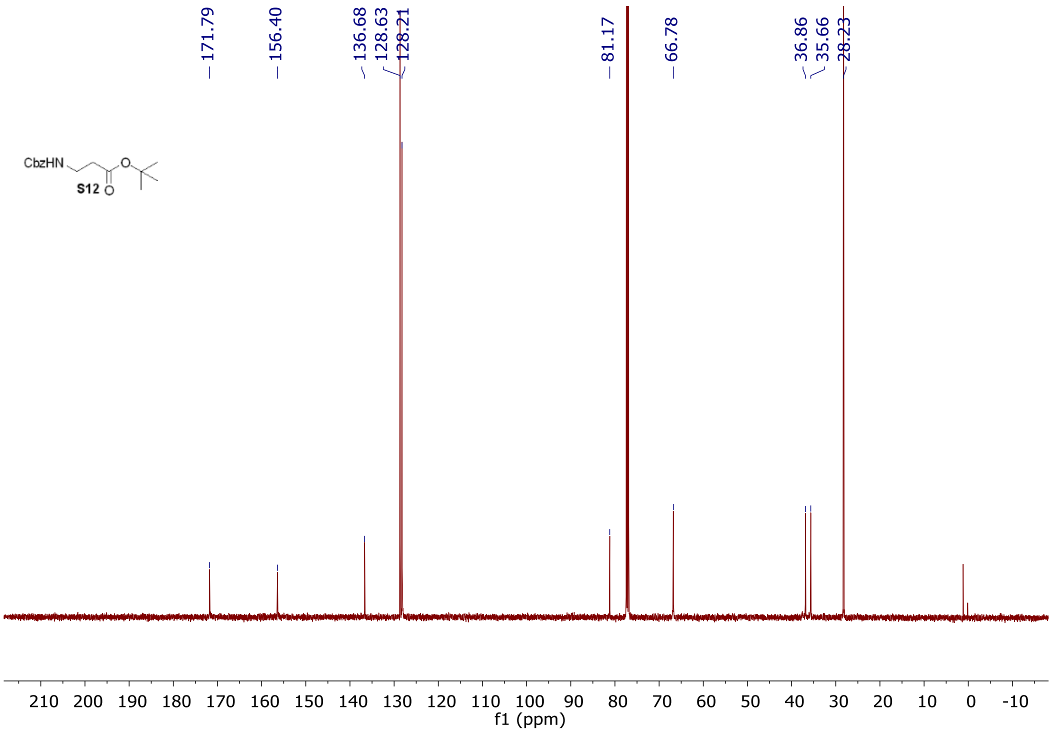

^{13}C NMR of S12

Supplementary Fig. 77. NMR of S13

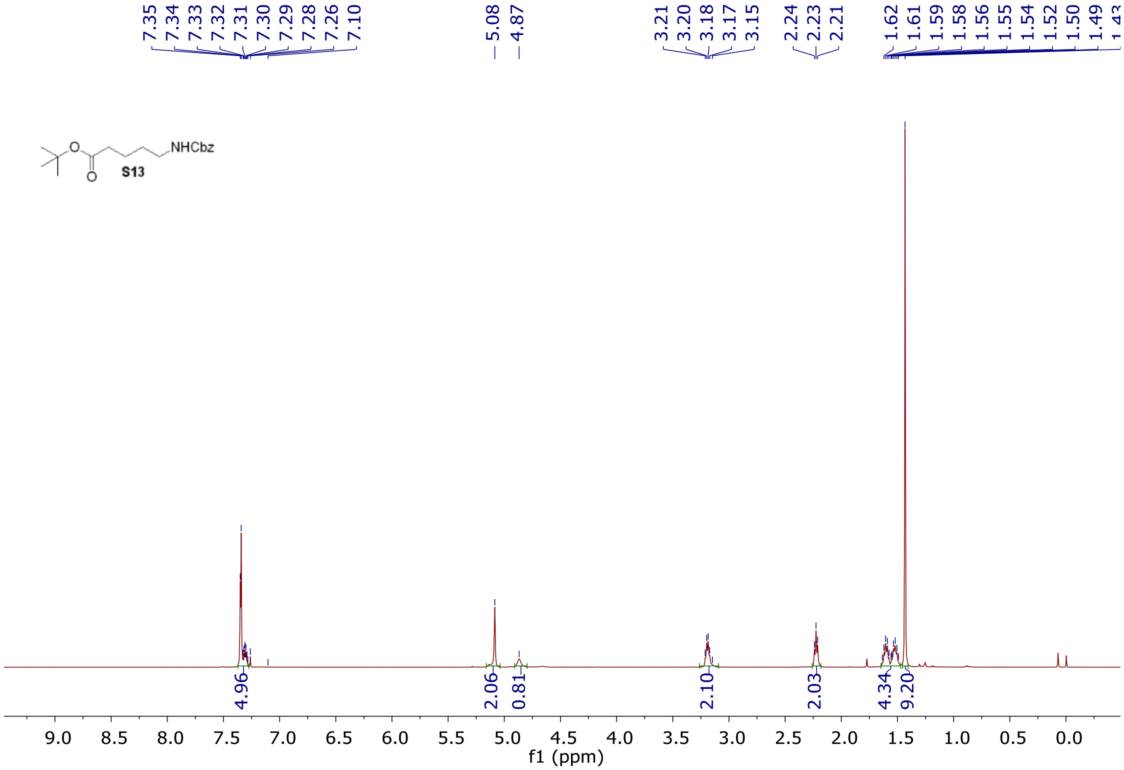

^{1}H NMR of S13

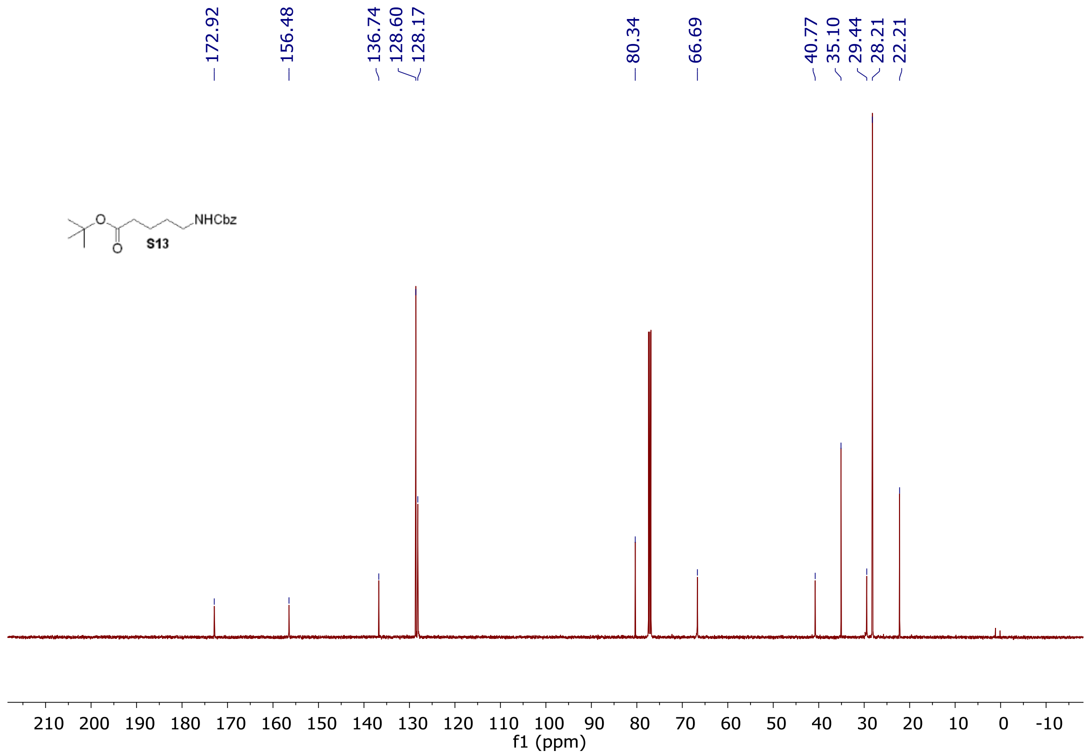

^{13}C NMR of S13

Supplementary Fig. 78. NMR of S14

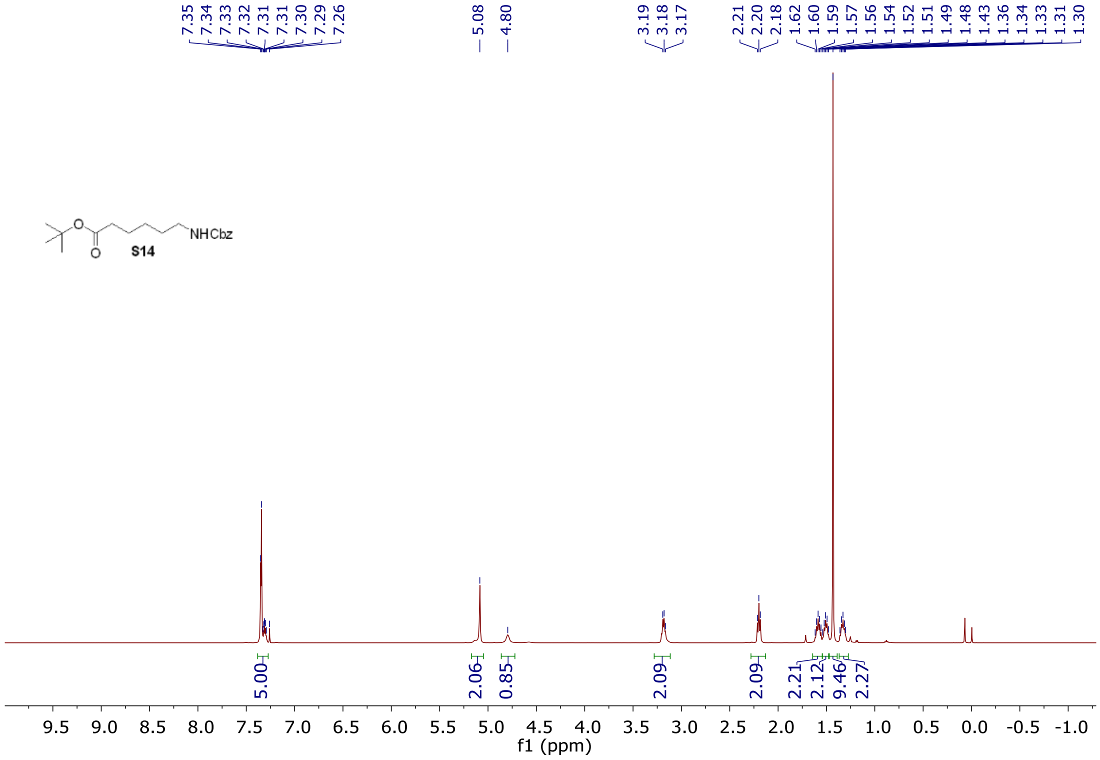

^{1}H NMR of S14

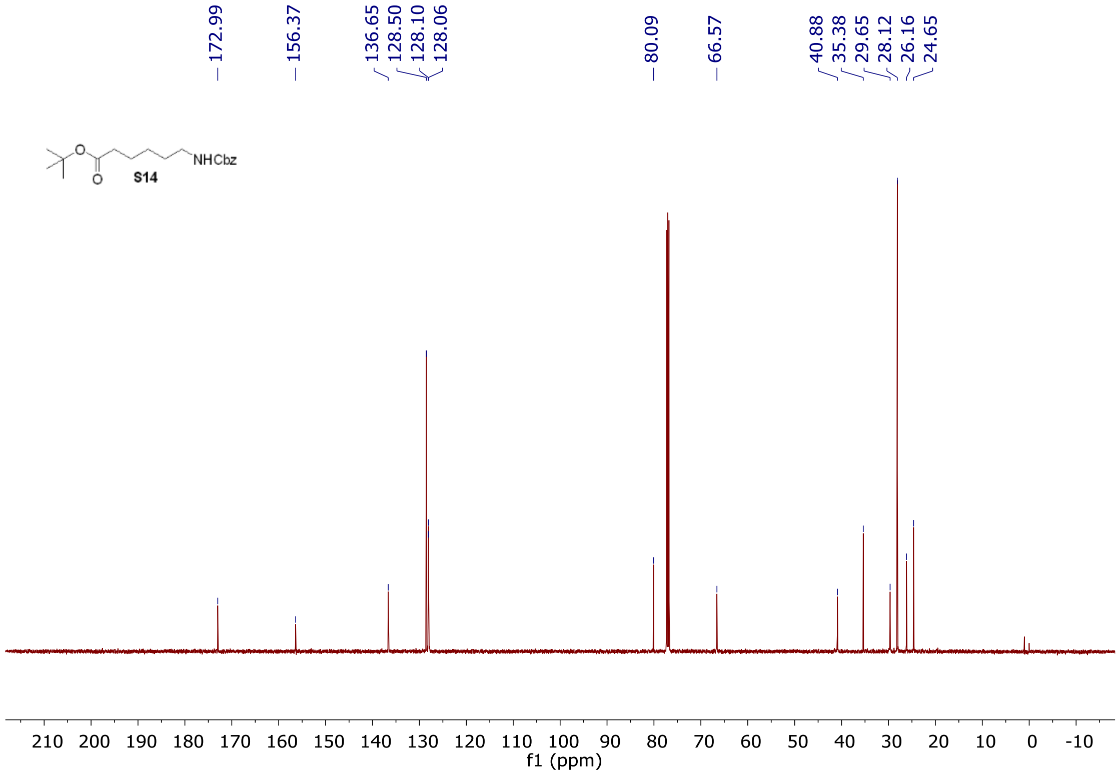

^{13}C NMR of S14

Supplementary Fig. 79. NMR of S15

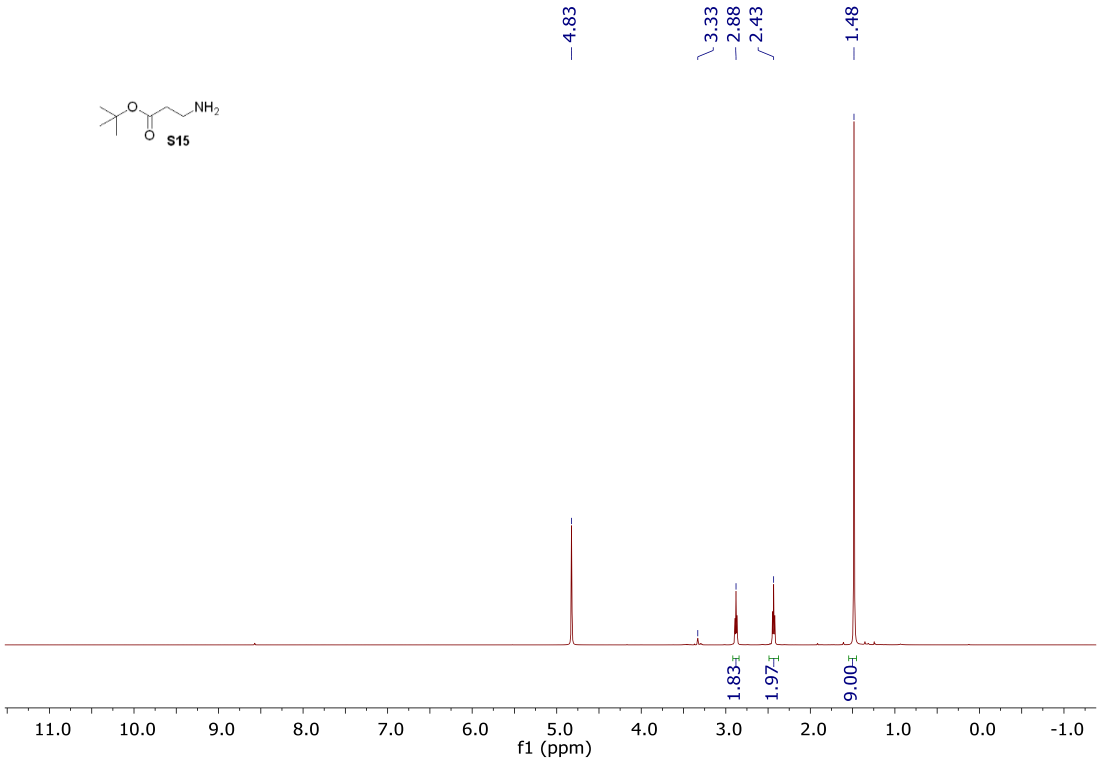

^{1}H NMR of S15

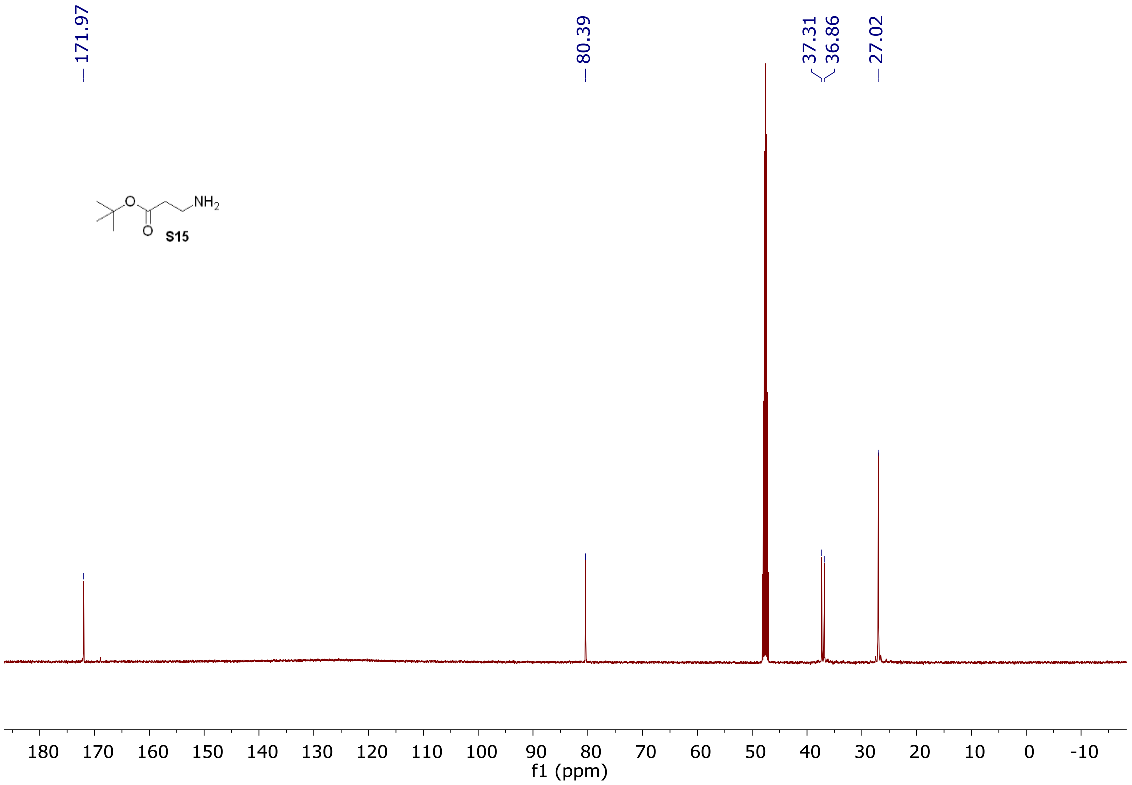

^{13}C NMR of S15

Supplementary Fig. 80. NMR of S16

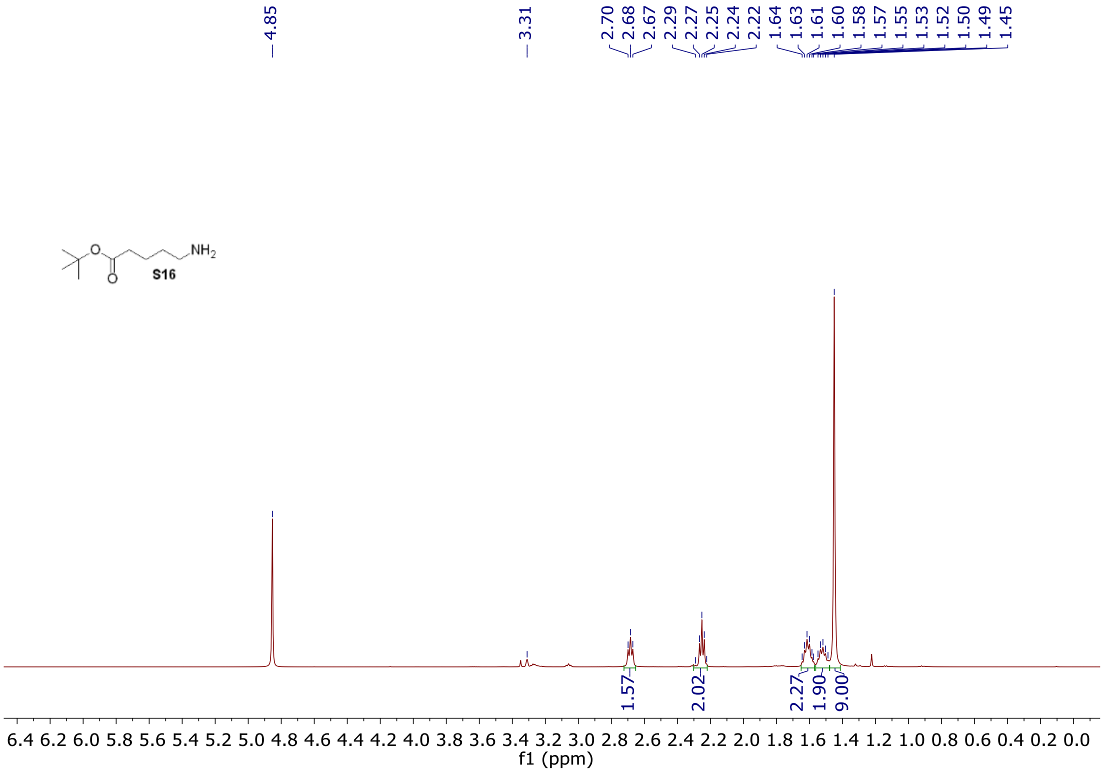

^{1}H NMR of S16

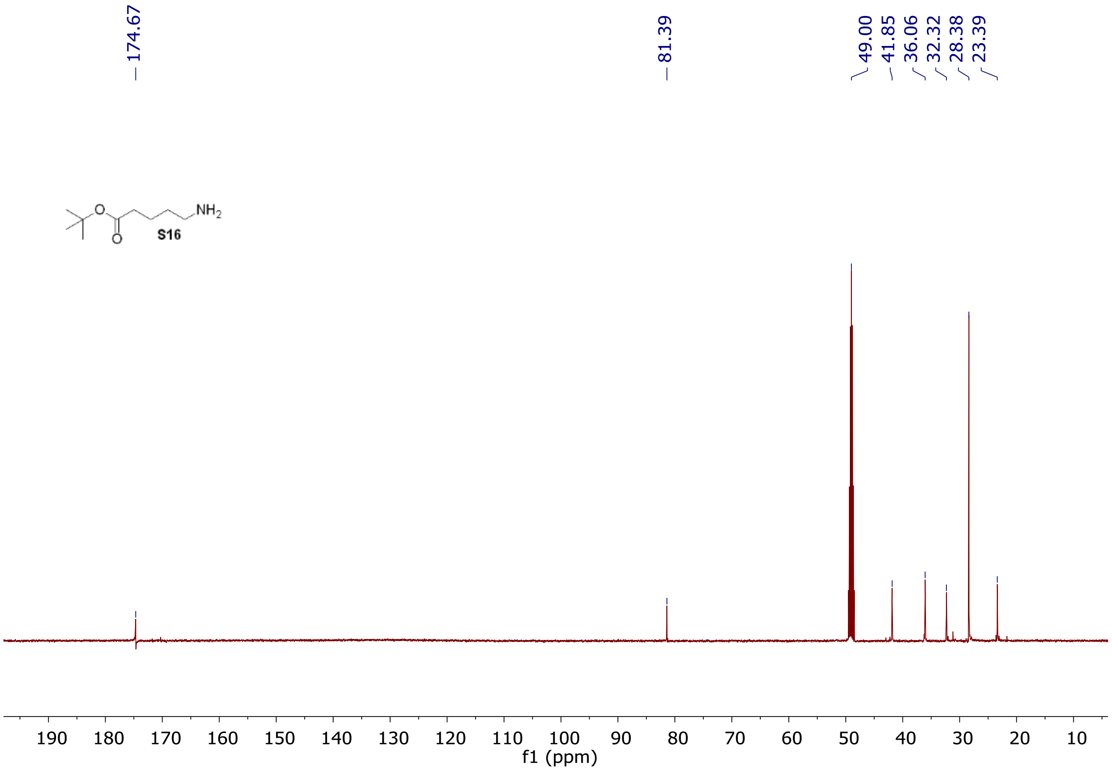

^{13}C NMR of S16

Supplementary Fig. 81. NMR of S17

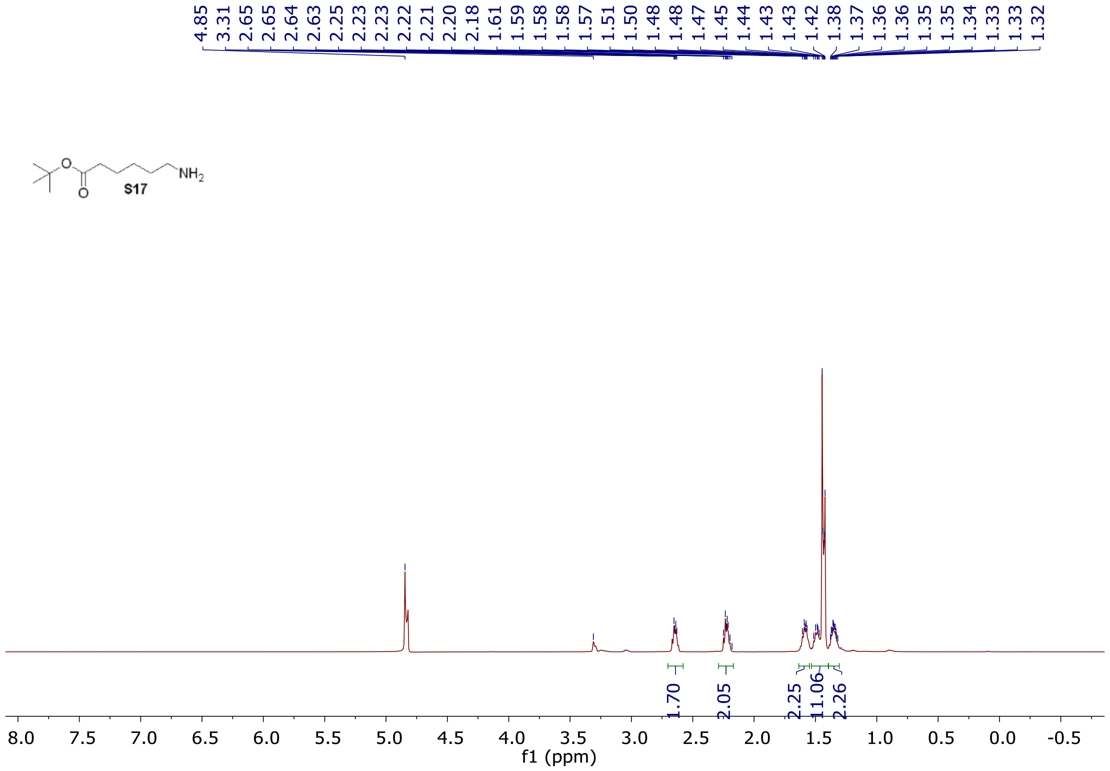

^{1}H NMR of S17

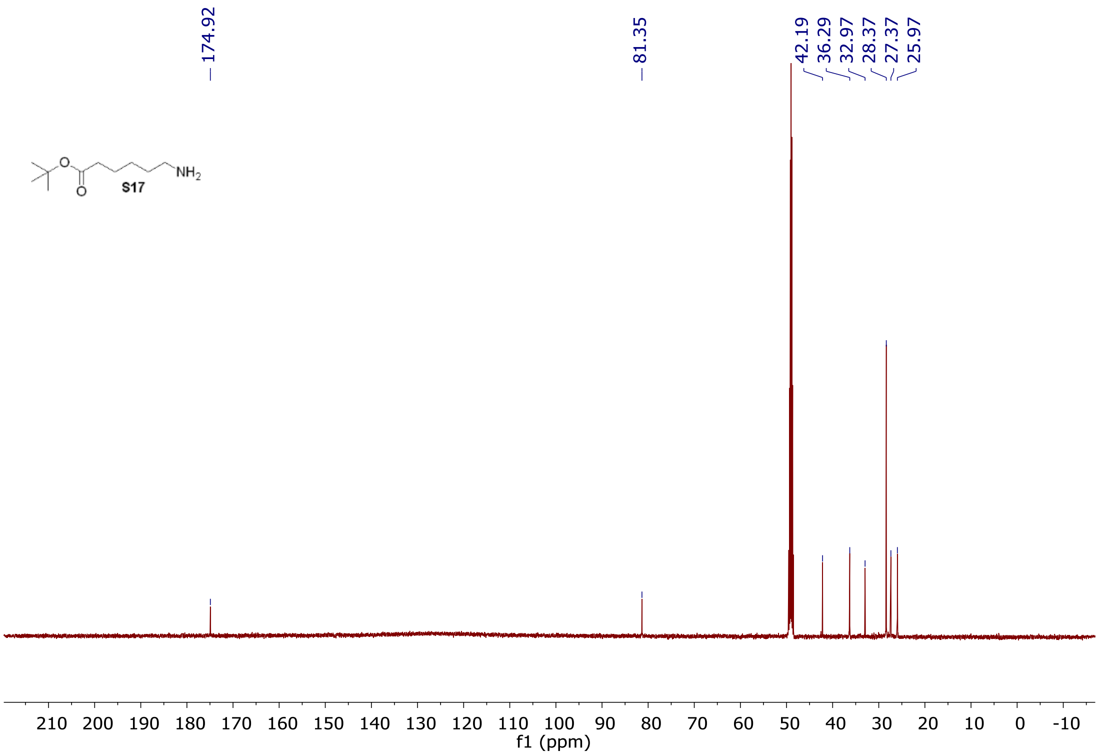

^{13}C NMR of S17

Supplementary Fig. 82. NMR of C3-boc-Tz

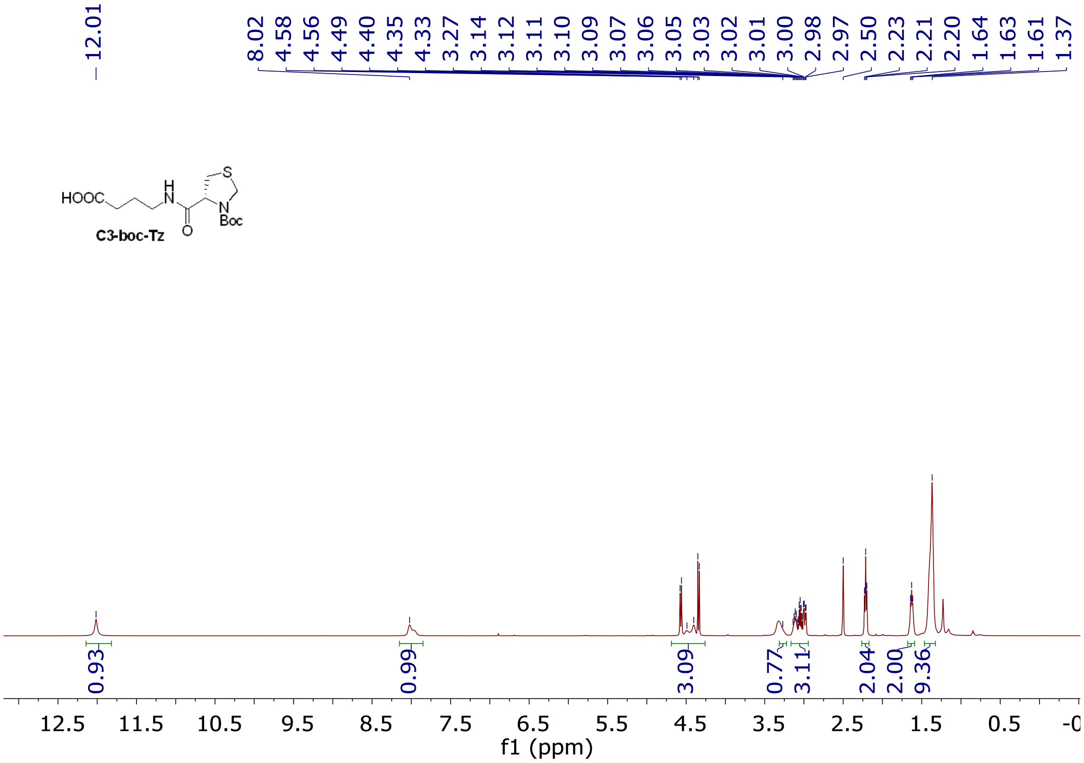

^{1}H NMR of C3-boc-Tz

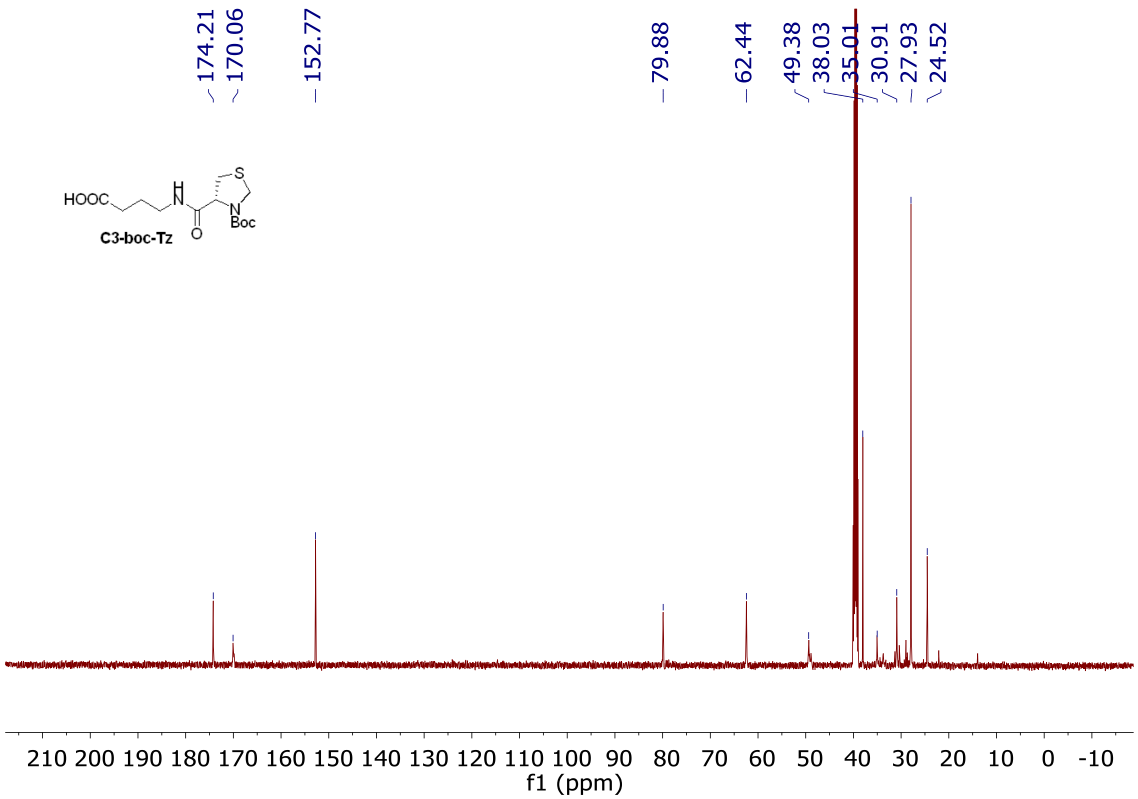

^{13}C NMR of C3-boc-Tz

Supplementary Fig. 83. NMR of C7-boc-Tz

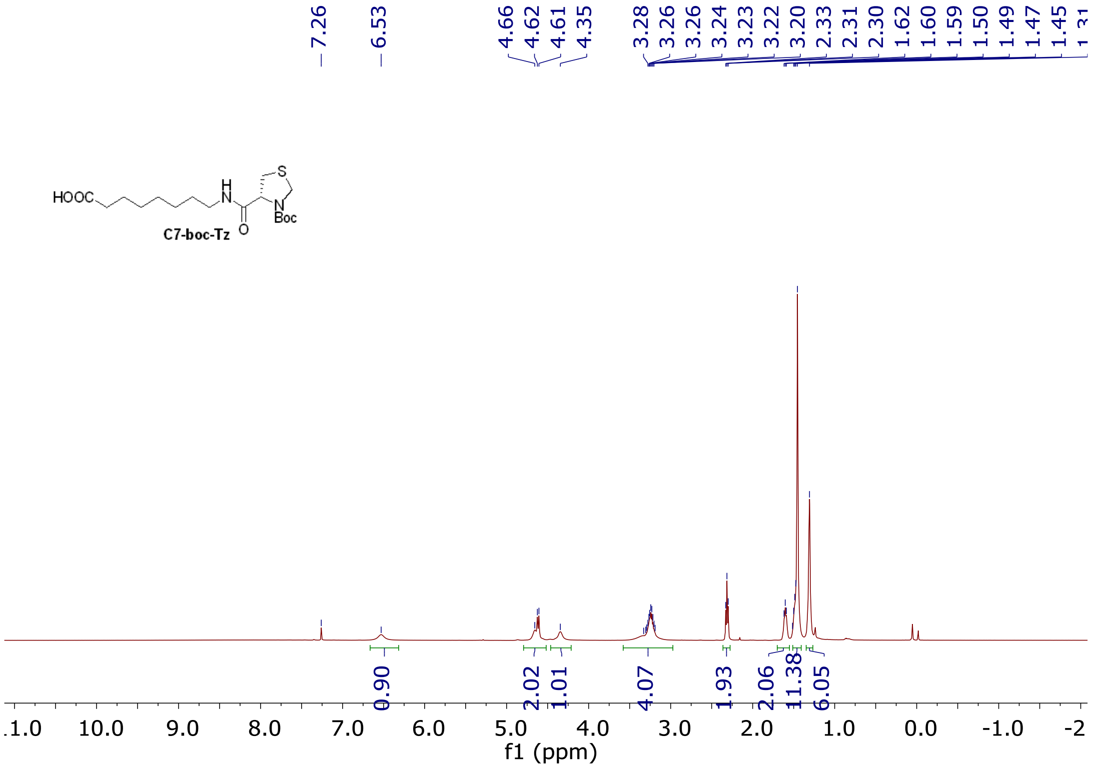

^{1}H NMR of C7-boc-Tz

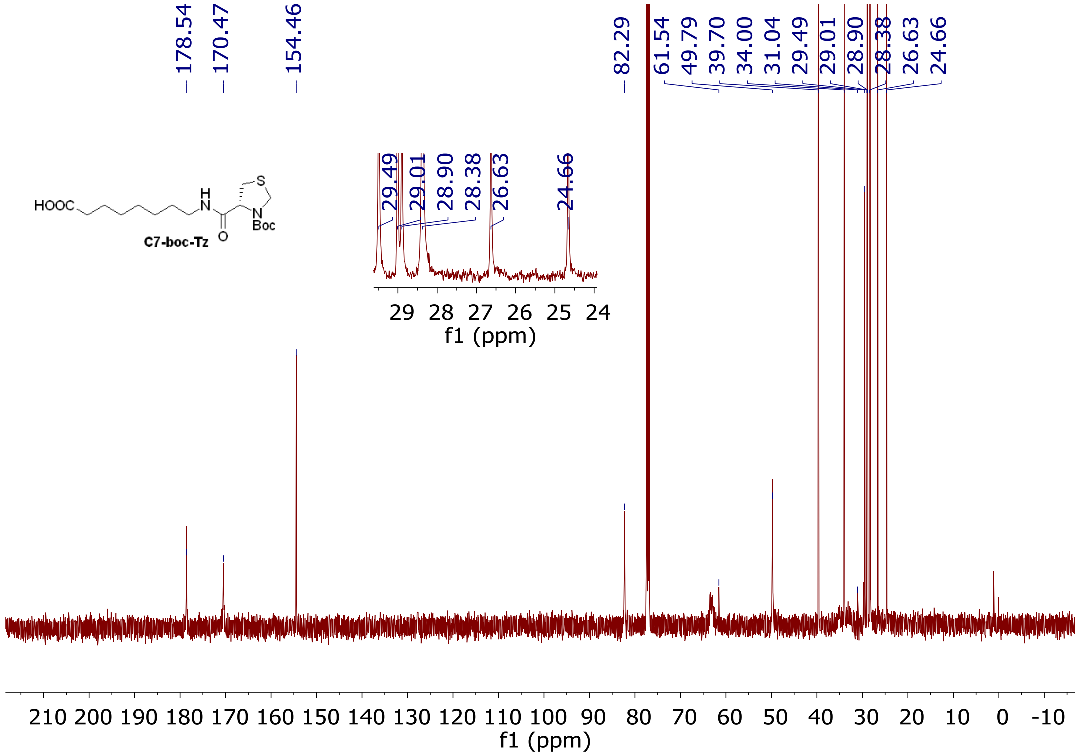

^{13}C NMR of C7-boc-Tz

Supplementary Fig. 84. NMR of C3Tz

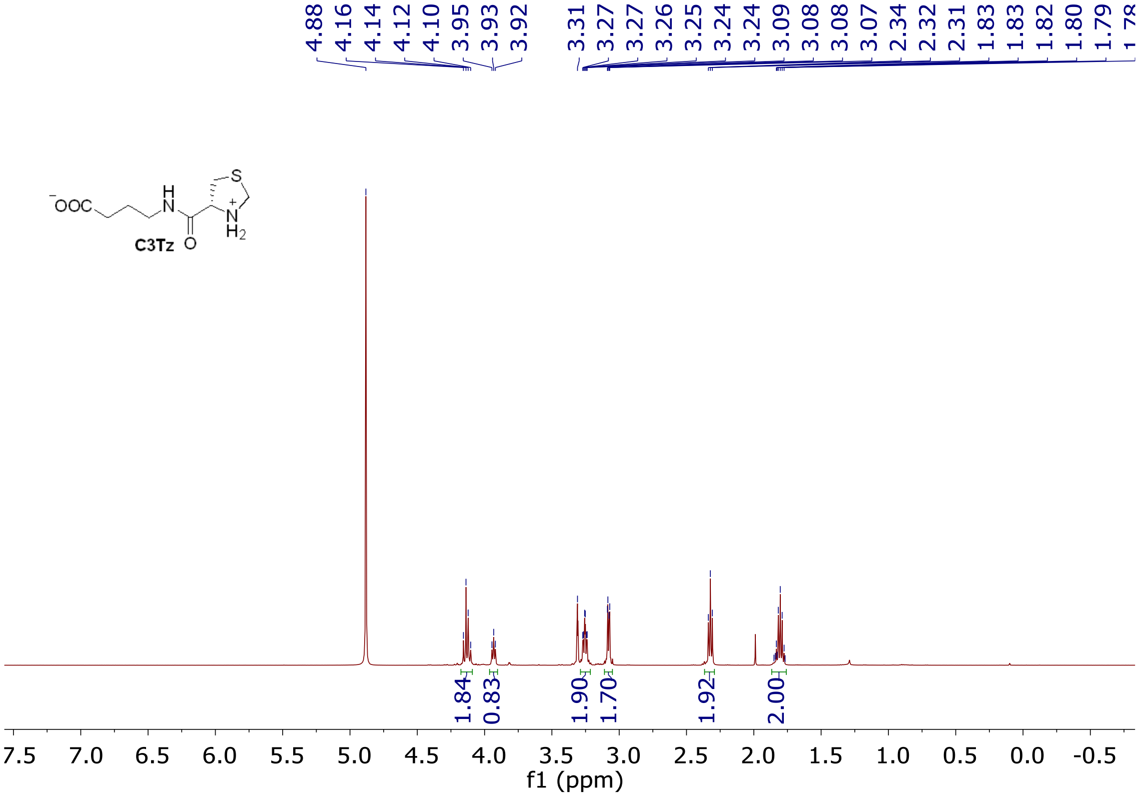

^{1}H NMR of C3Tz

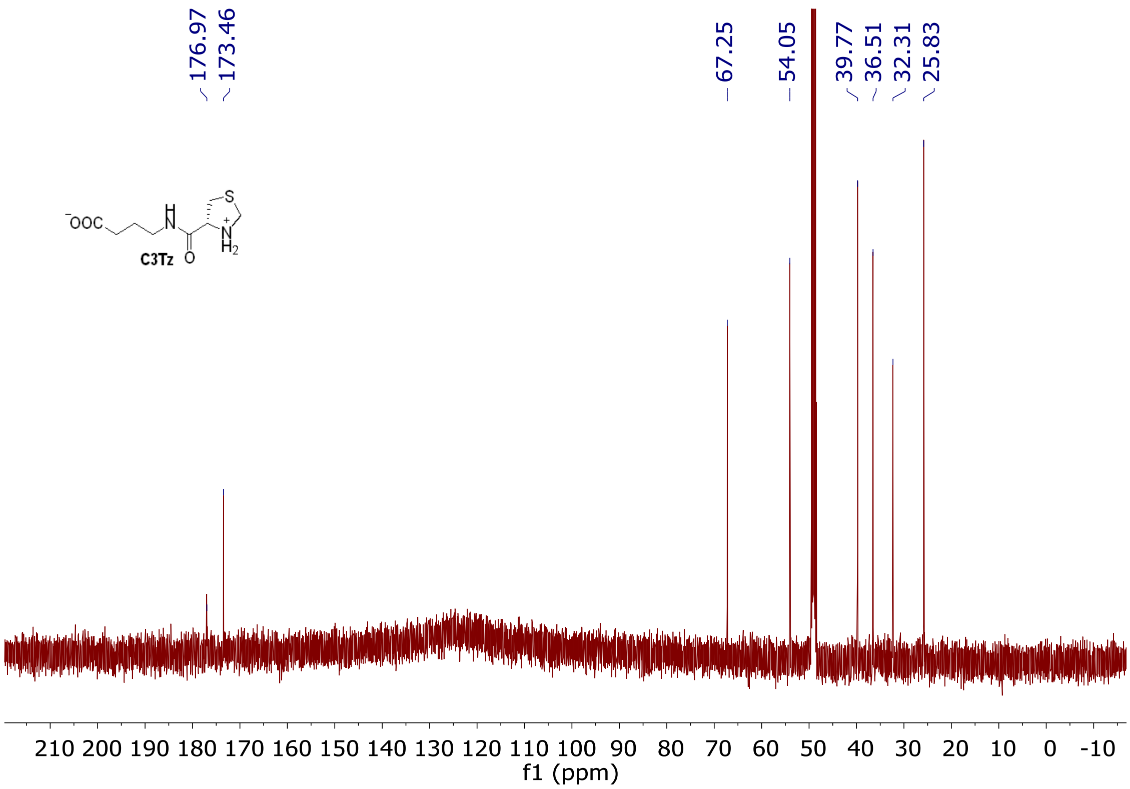

^{13}C NMR of C3Tz

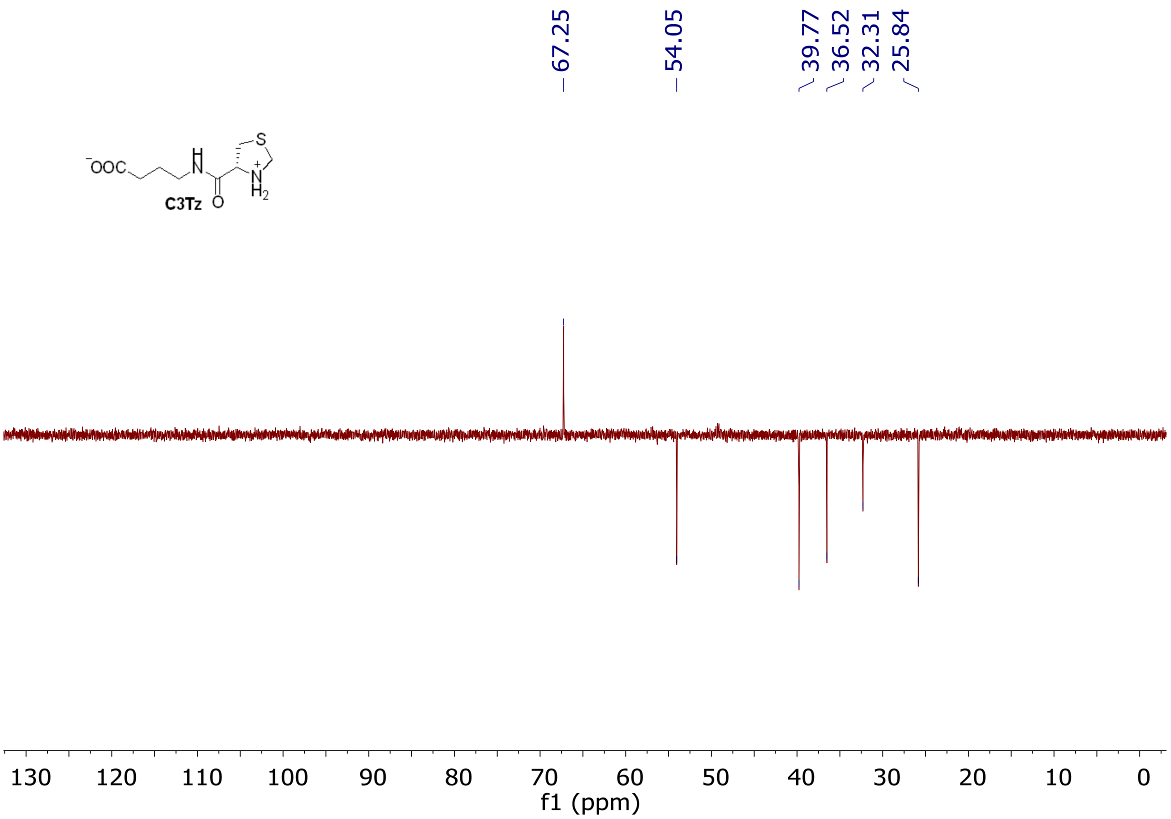

^{13}C DEPT-135 NMR of C3Tz

Supplementary Fig. 85. NMR of C7Tz

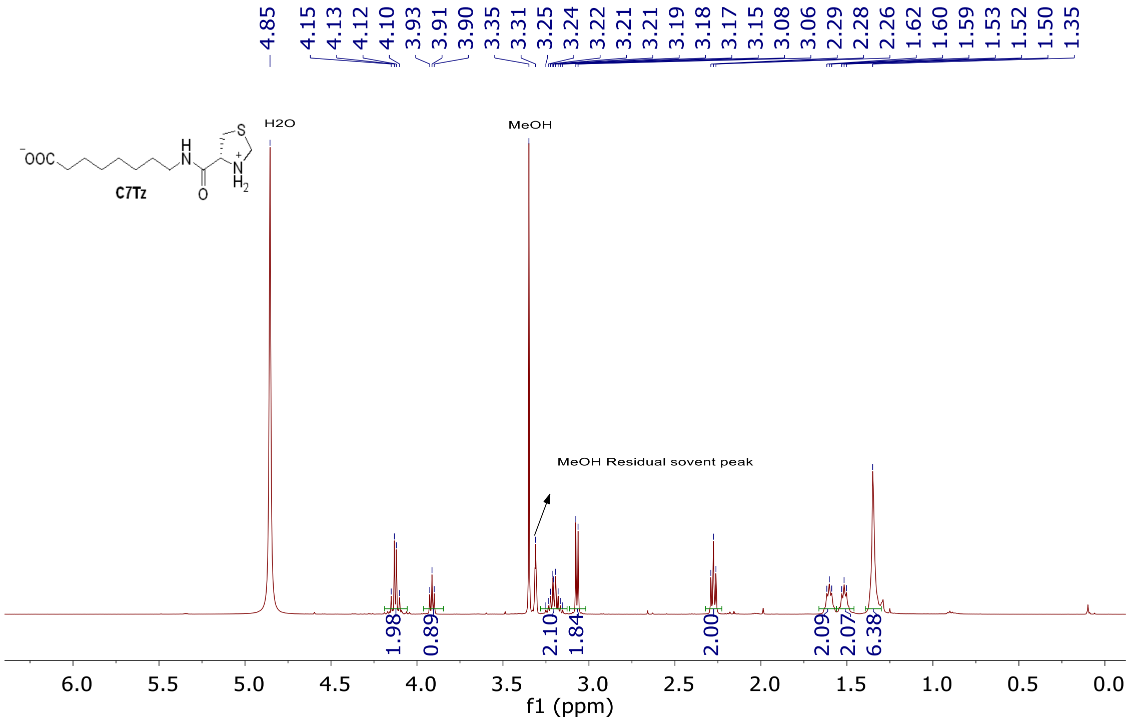

^{1}H NMR of C7Tz

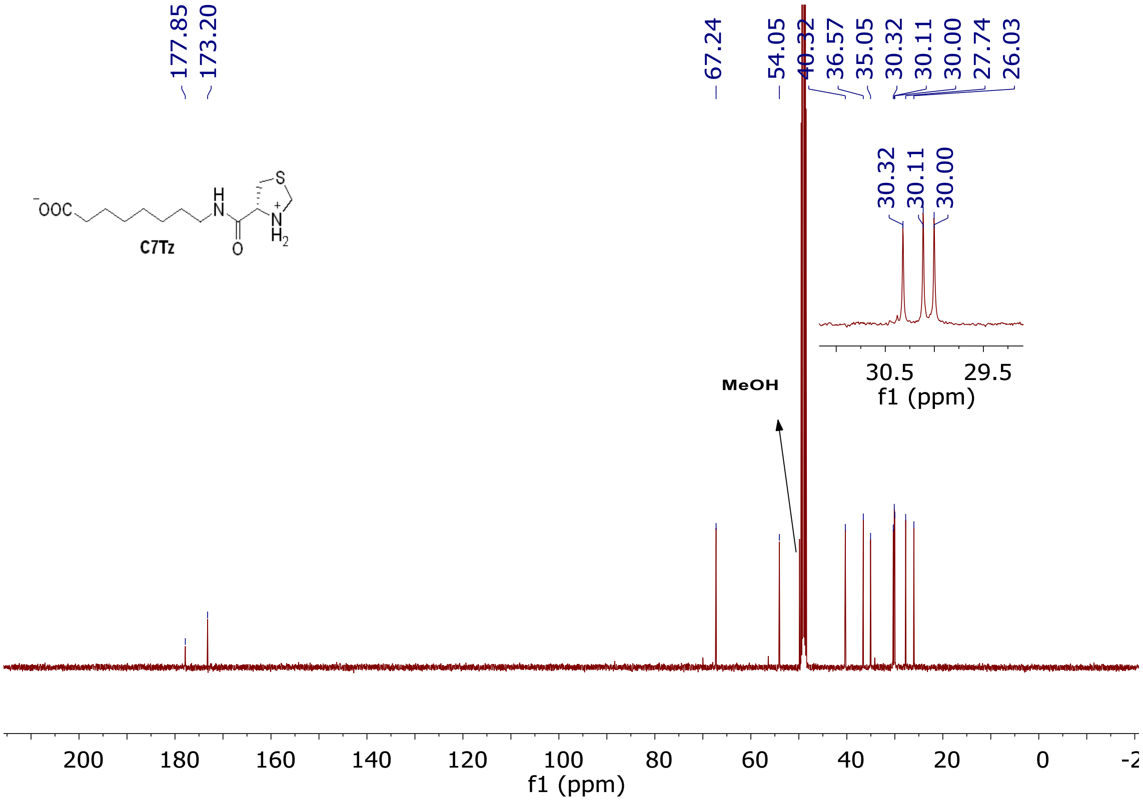

^{13}C NMR of C7Tz

Supplementary Fig. 86. NMR of C2-boc-Tz

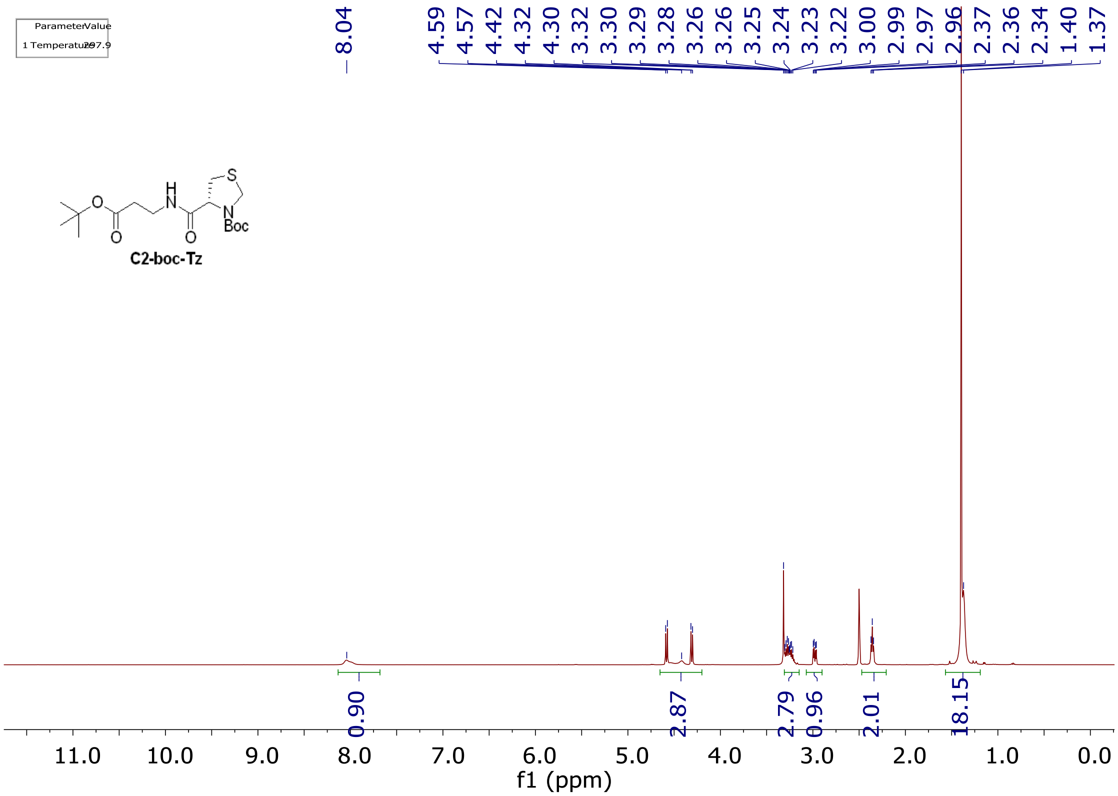

^{1}H NMR of C2-boc-Tz (T = 289K)

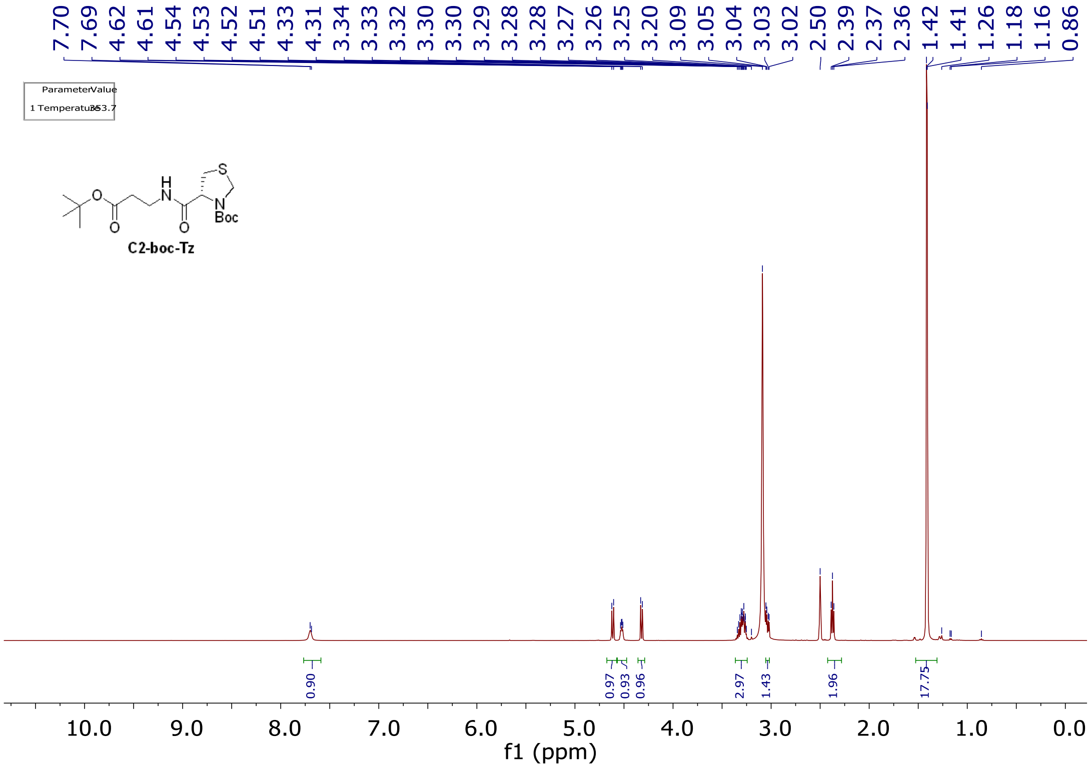

^{1}H NMR of C2-boc-Tz (T = 353K)

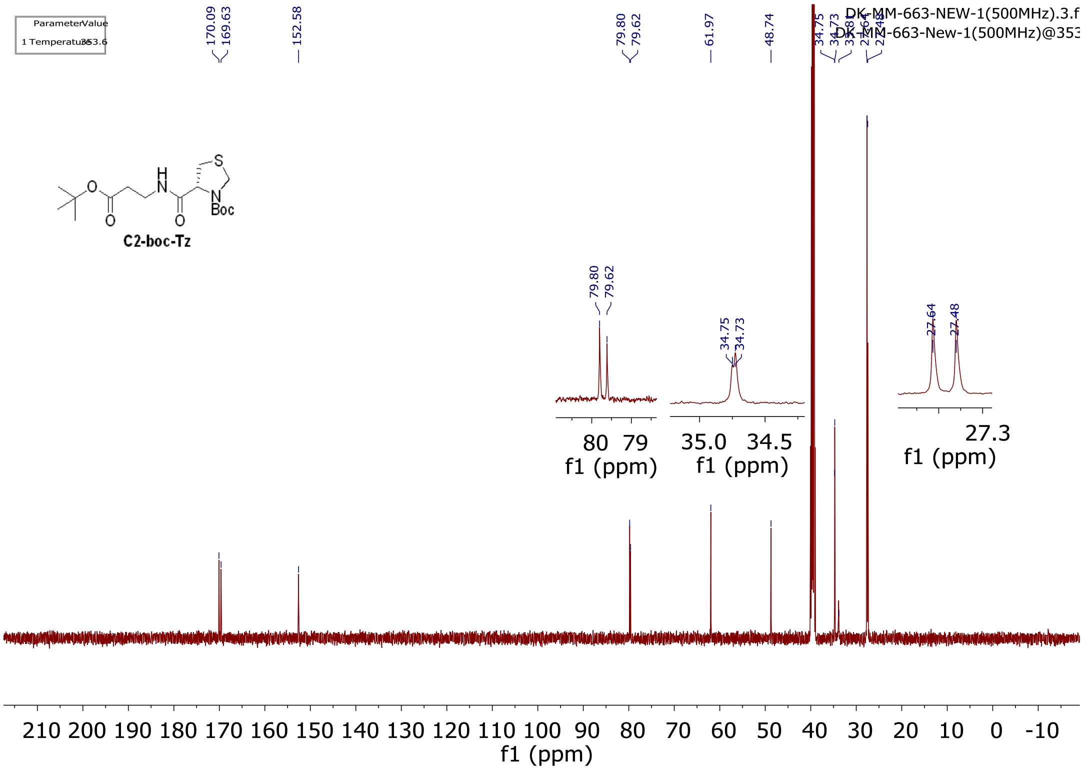

^{13}C NMR of C2-boc-Tz (T = 353K)

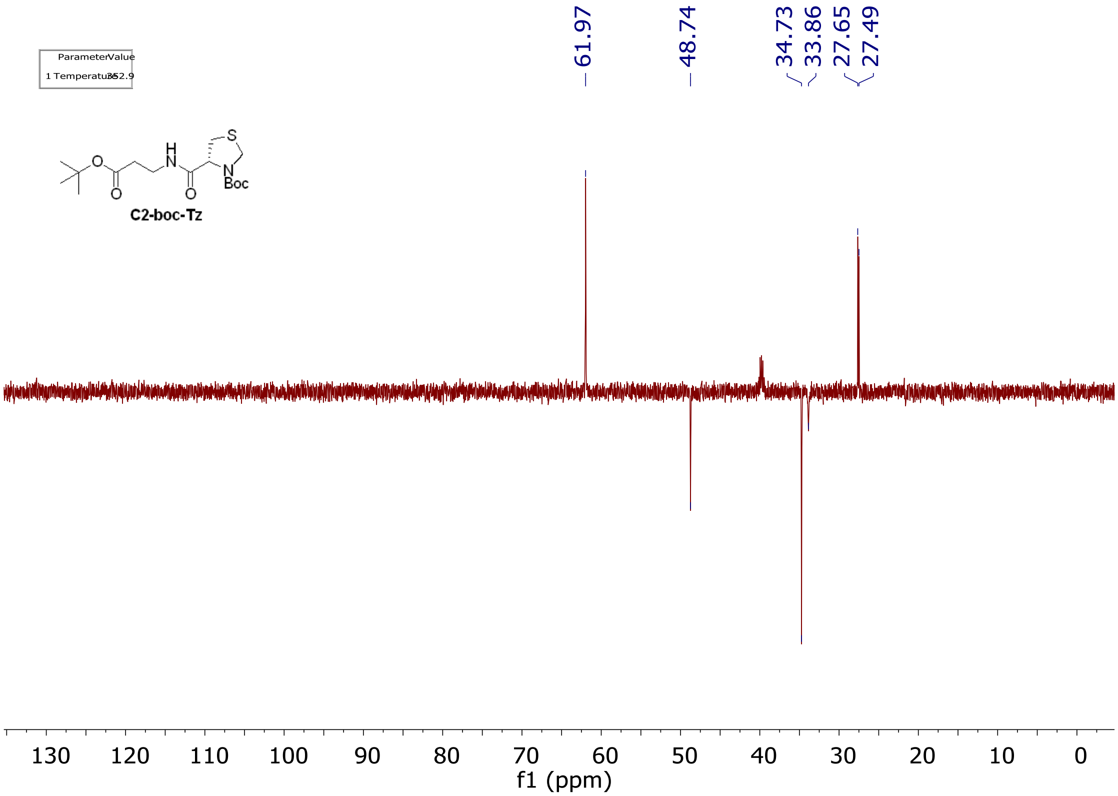

^{13}C DEPT-135 NMR of C2-boc-Tz (T = 353K)

Supplementary Fig. 87. NMR of C4-boc-Tz

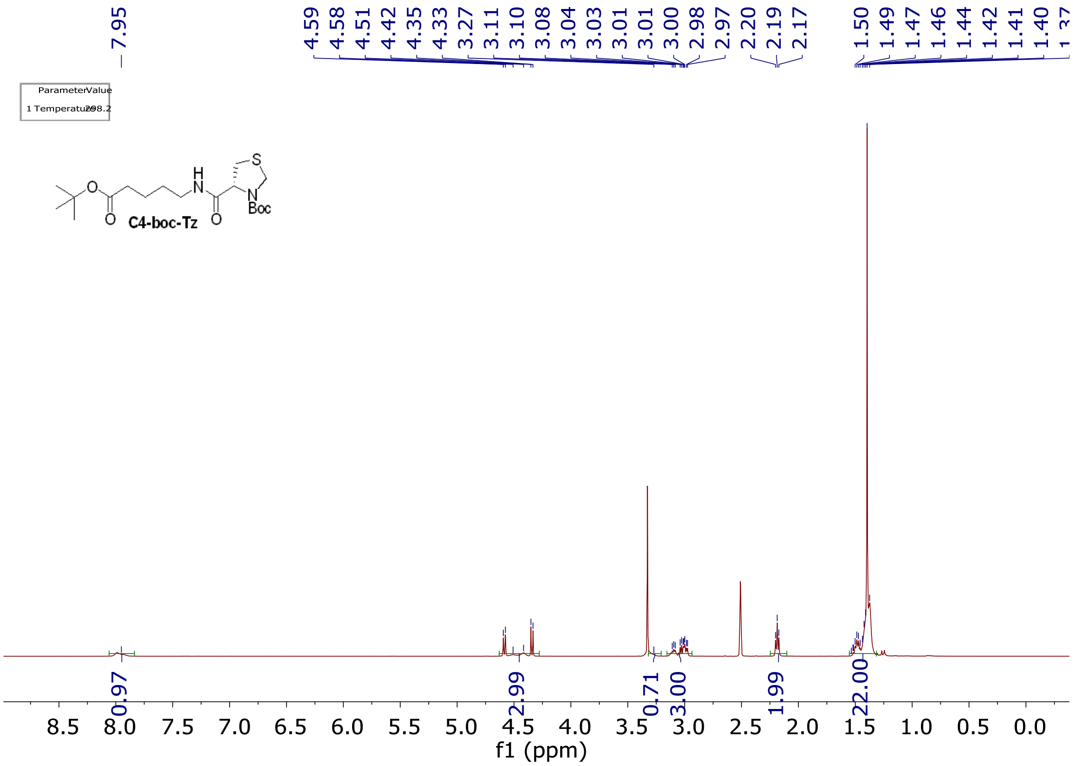

^{1}H NMR of C4-boc-Tz (T = 289K)

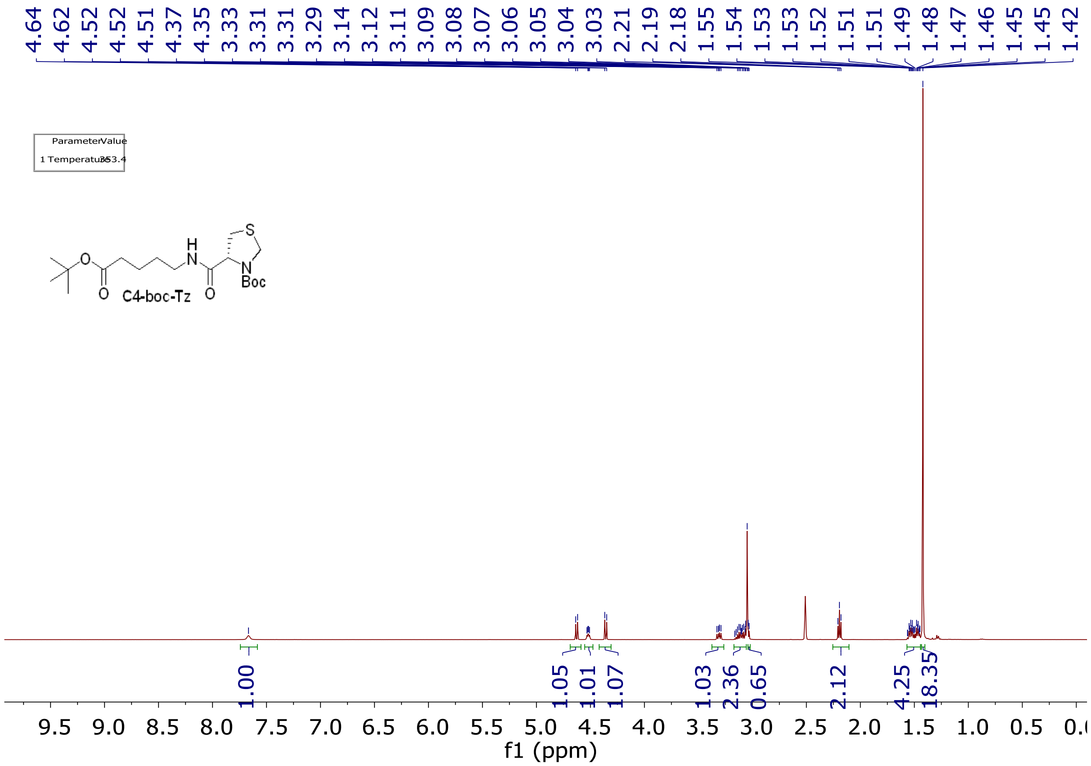

^{1}H NMR of C4-boc-Tz (T = 353K)

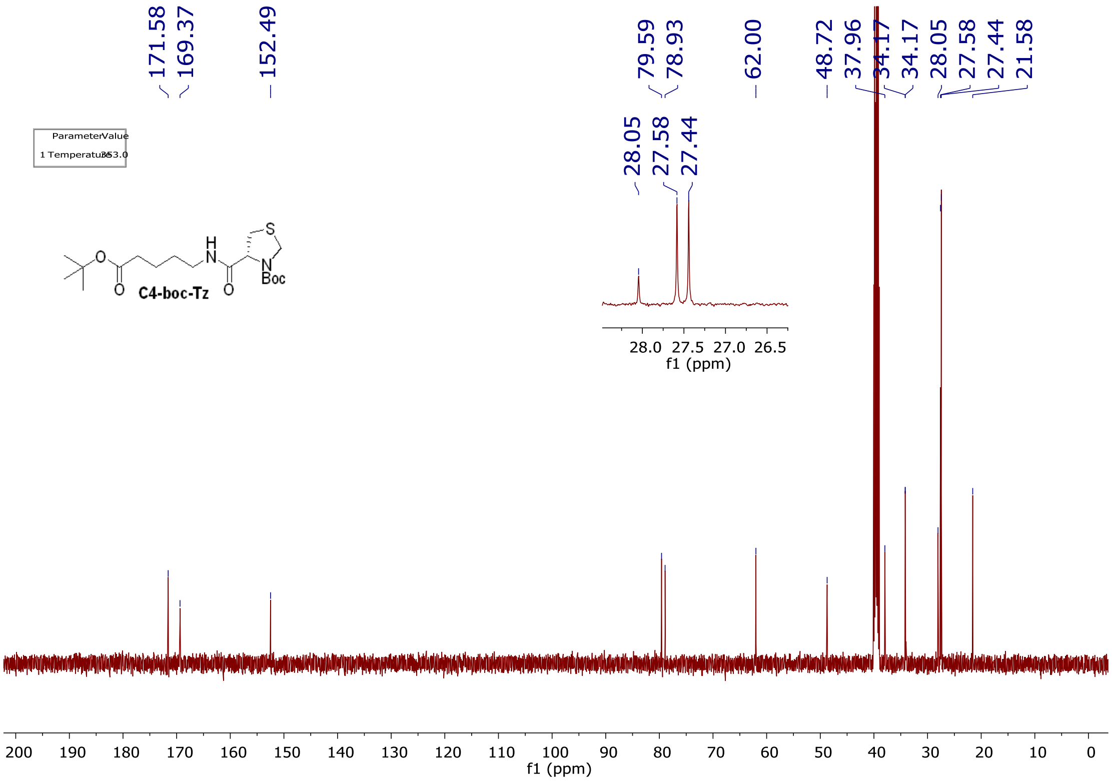

^{13}C NMR of C4-boc-Tz (T = 353K)

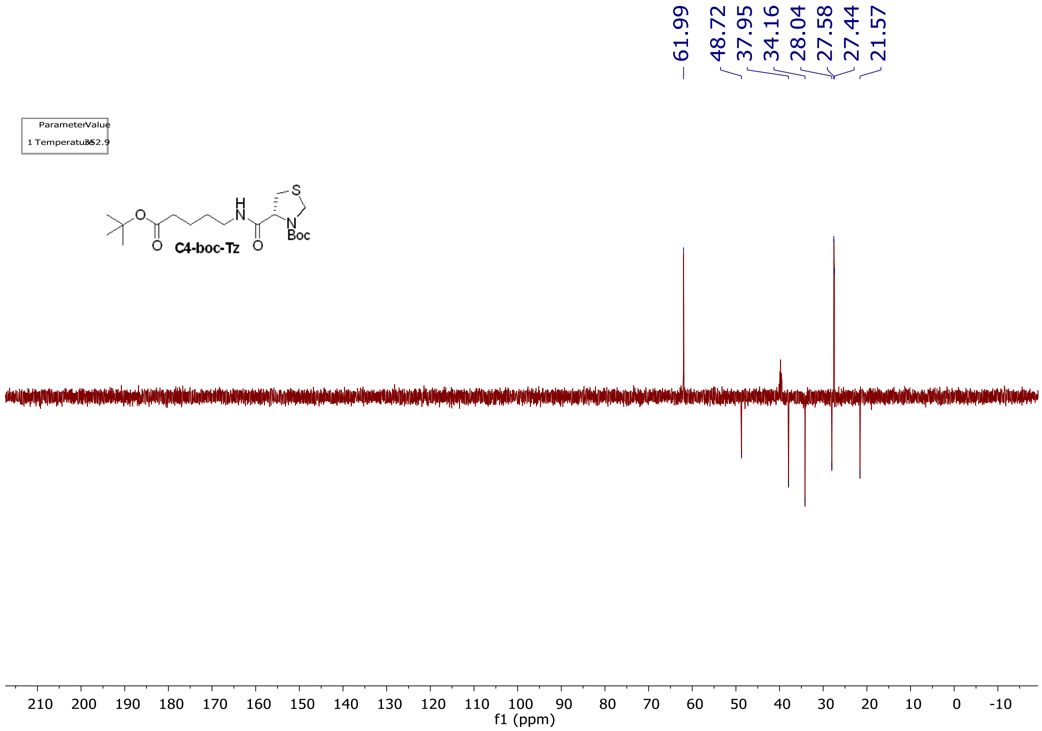

^{13}C DEPT-135 NMR of C4-boc-Tz (T = 353K)

Supplementary Fig. 88. NMR of C5-boc-Tz

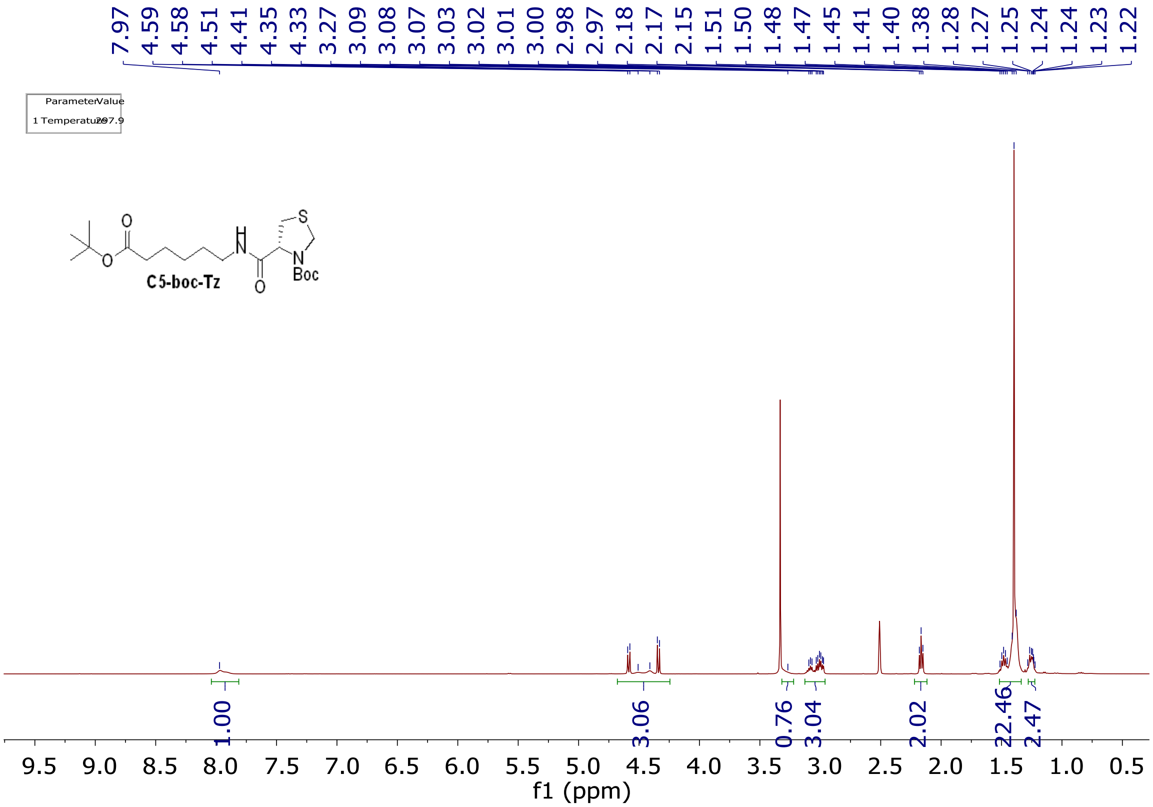

^{1}H NMR of C5-boc-Tz (T = 289K)

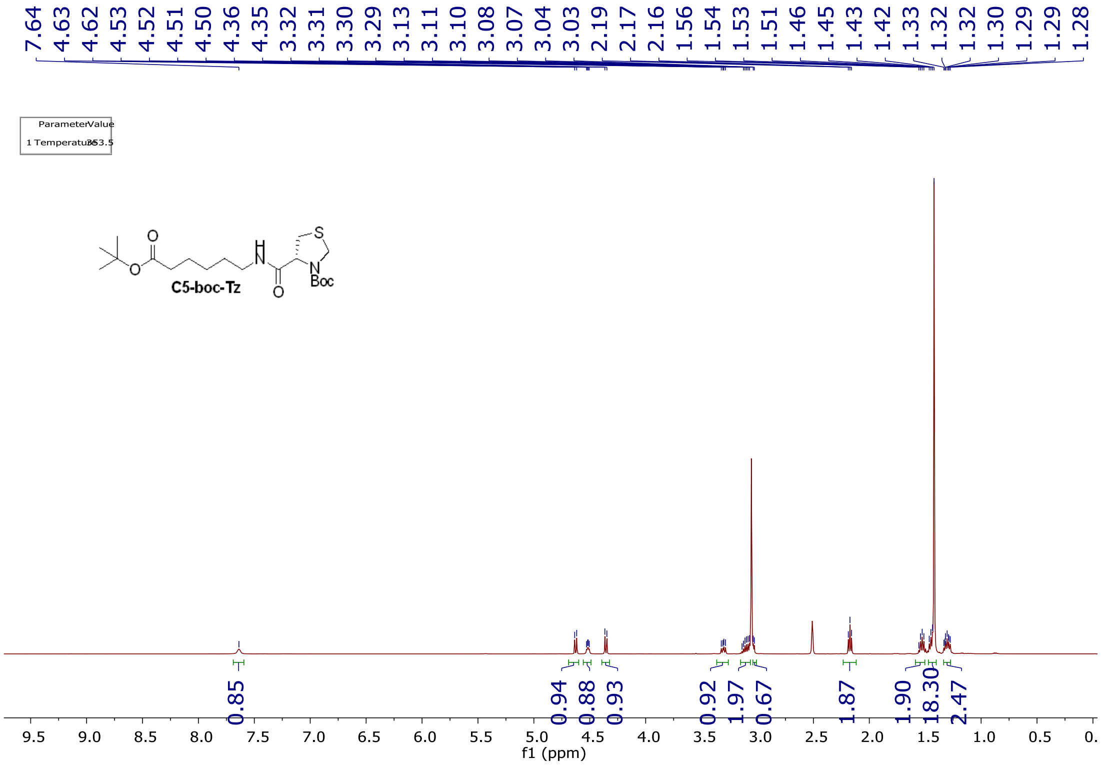

^{1}H NMR of C5-boc-Tz (T = 353K)

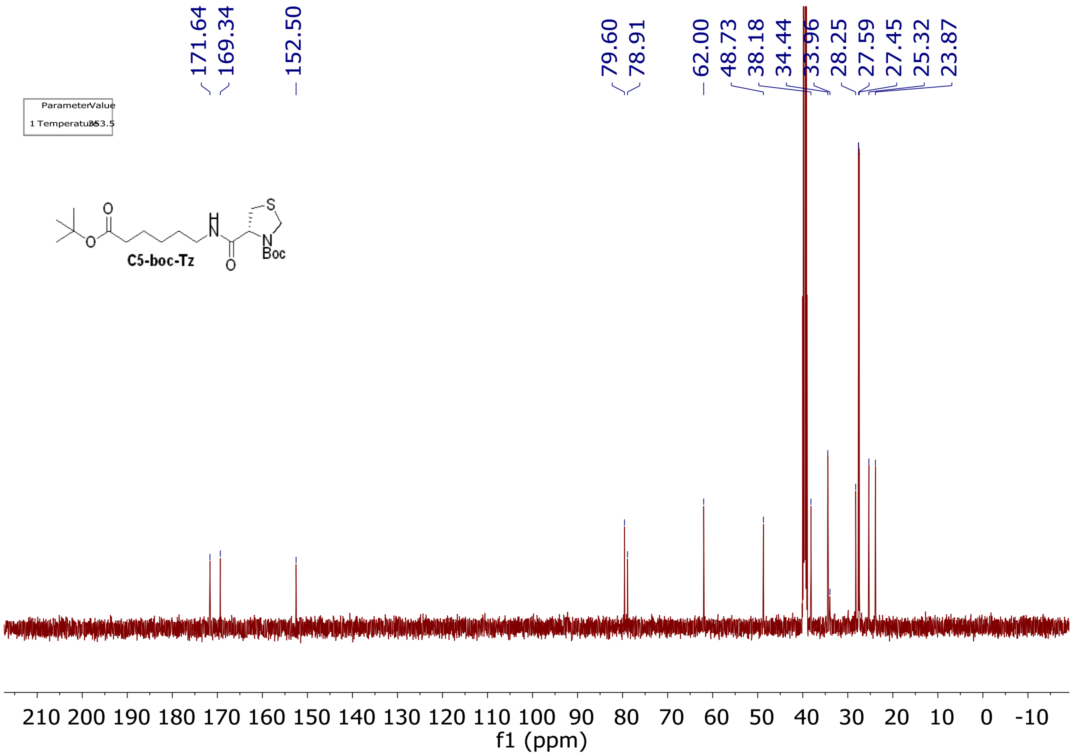

^{1}H NMR of C5-boc-Tz (T = 353K)

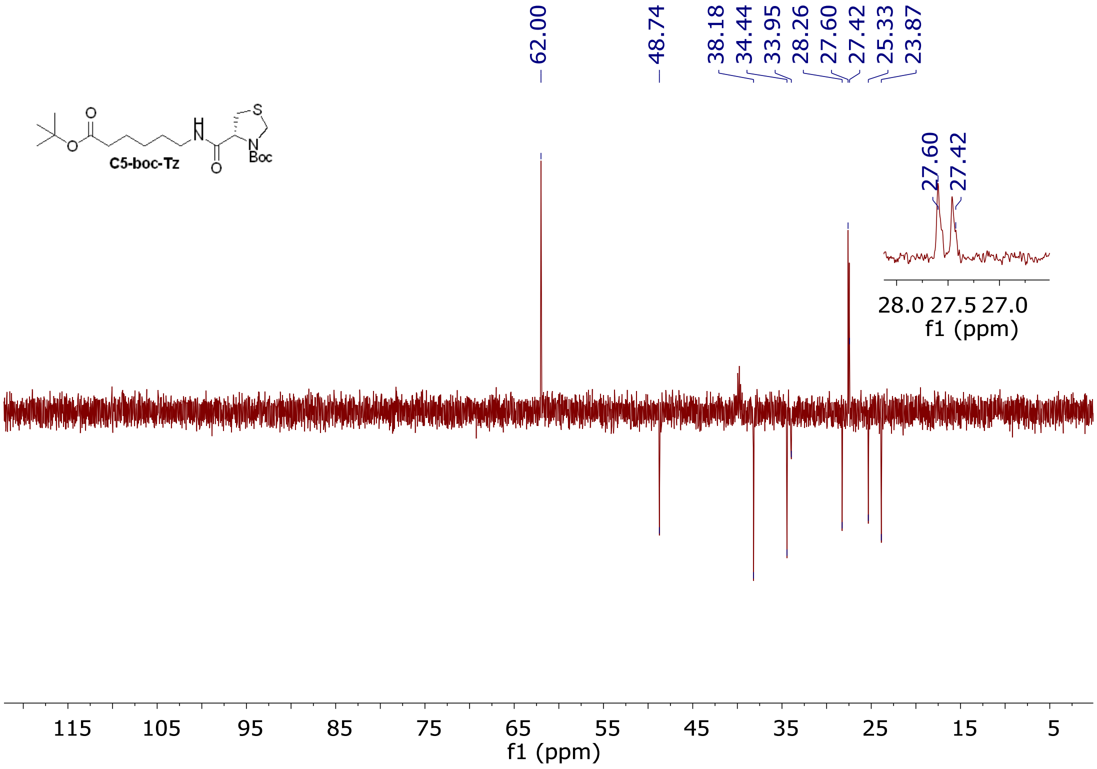

^{13}C DEPT-135 NMR of C5-boc-Tz (T = 353K)

Supplementary Fig. 89. NMR of C2Tz

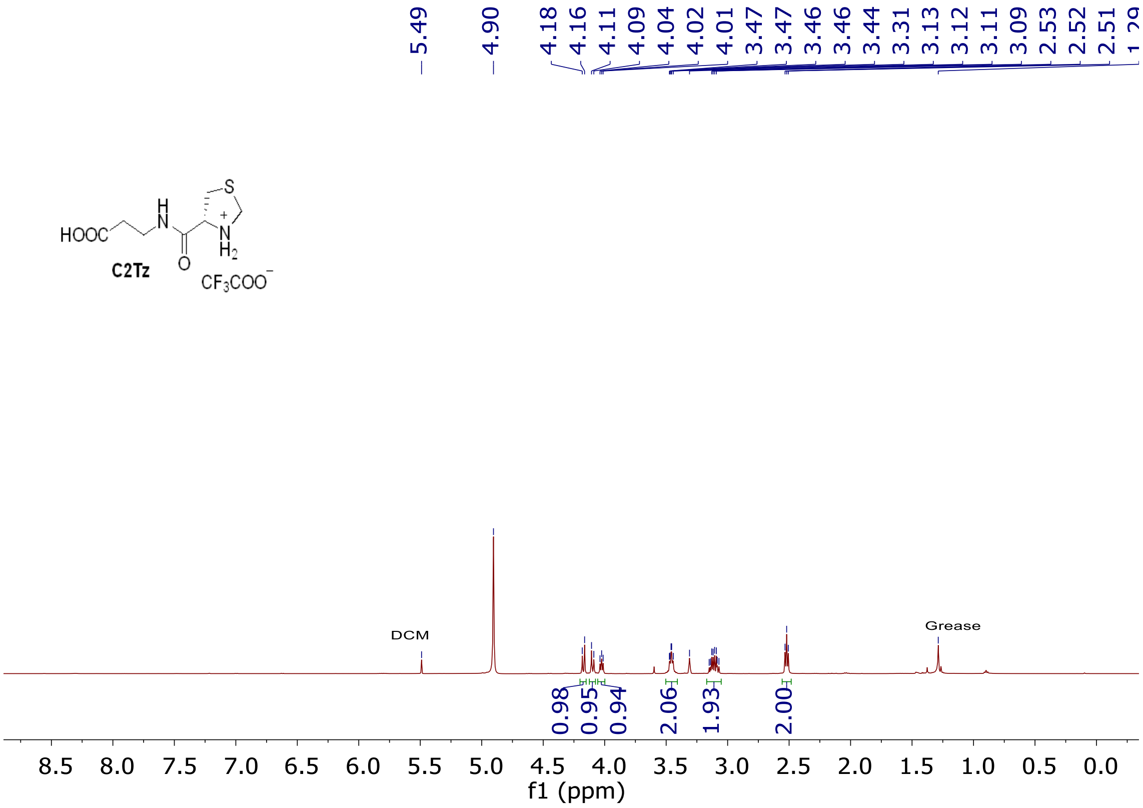

^{1}H NMR of C2Tz

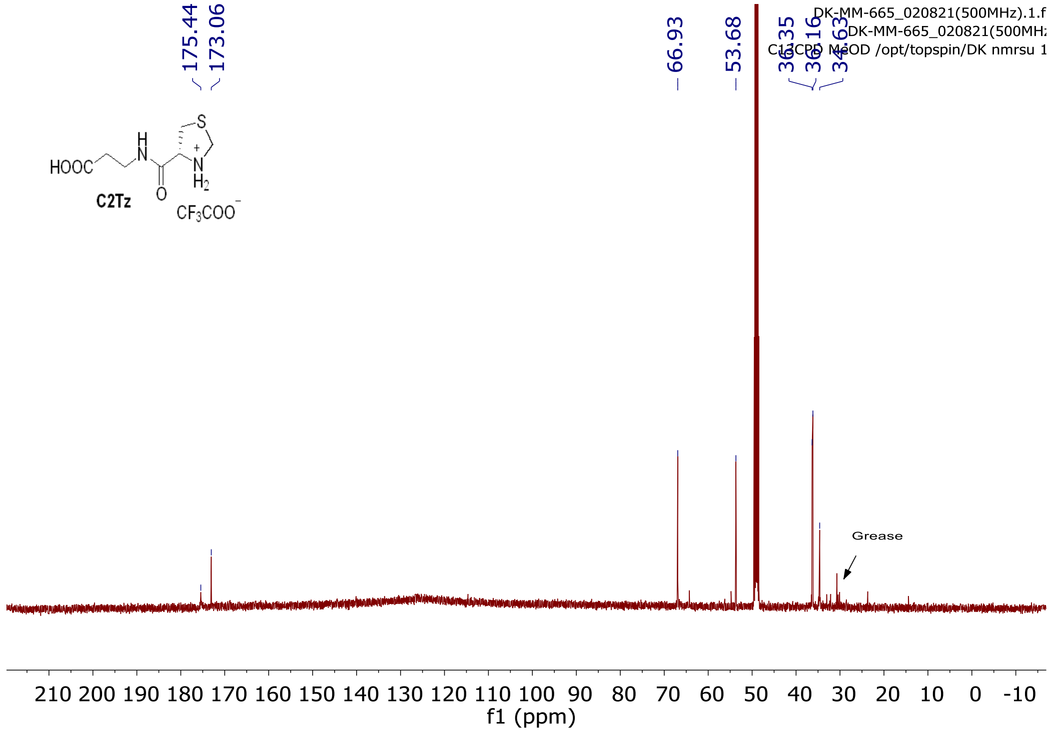

^{13}C NMR of C2Tz

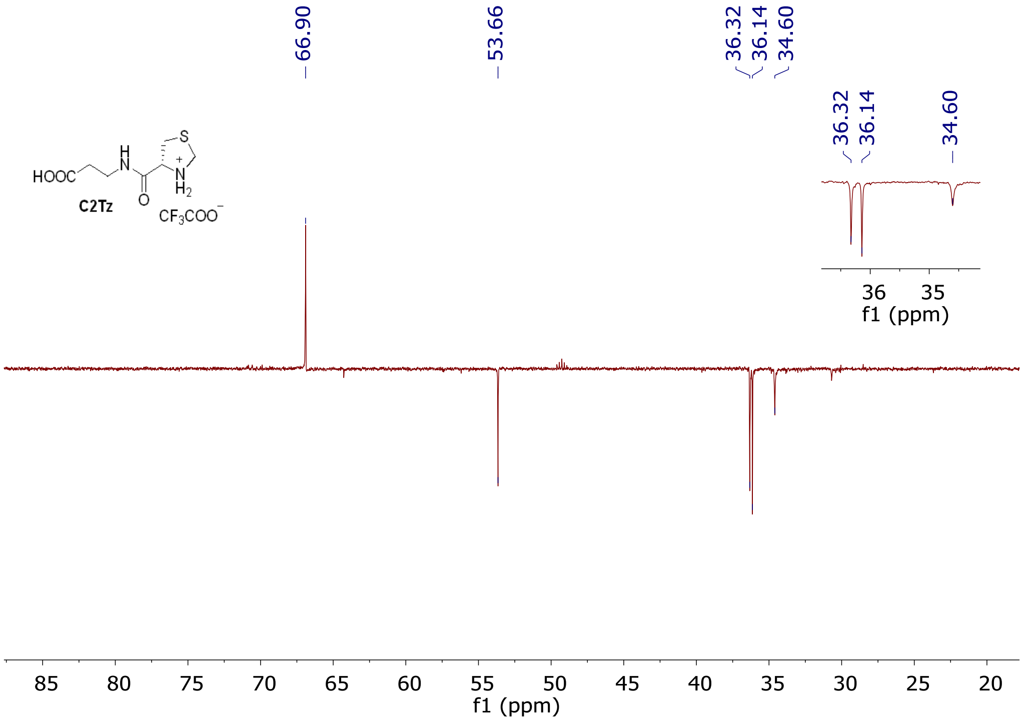

^{13}C DEPT-135 NMR of C2Tz

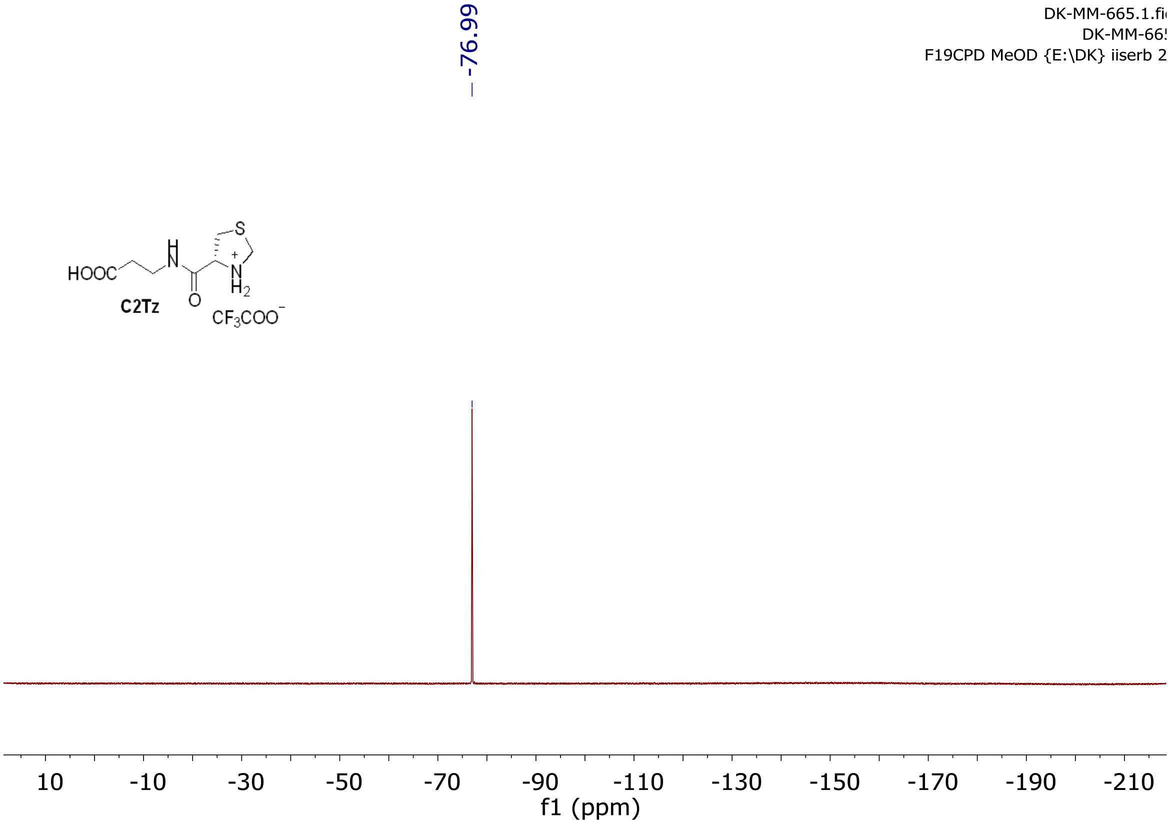

^{19}F NMR of C2Tz

Supplementary Fig. 90. NMR of C4Tz

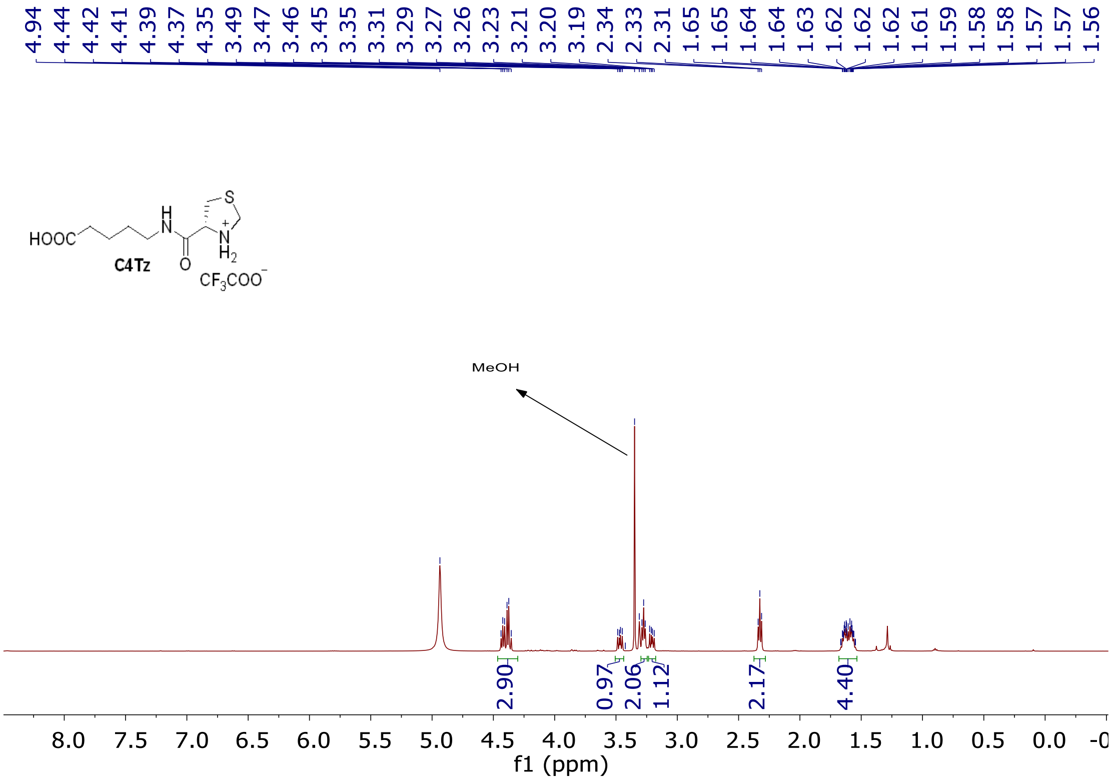

^{1}H NMR of C4Tz

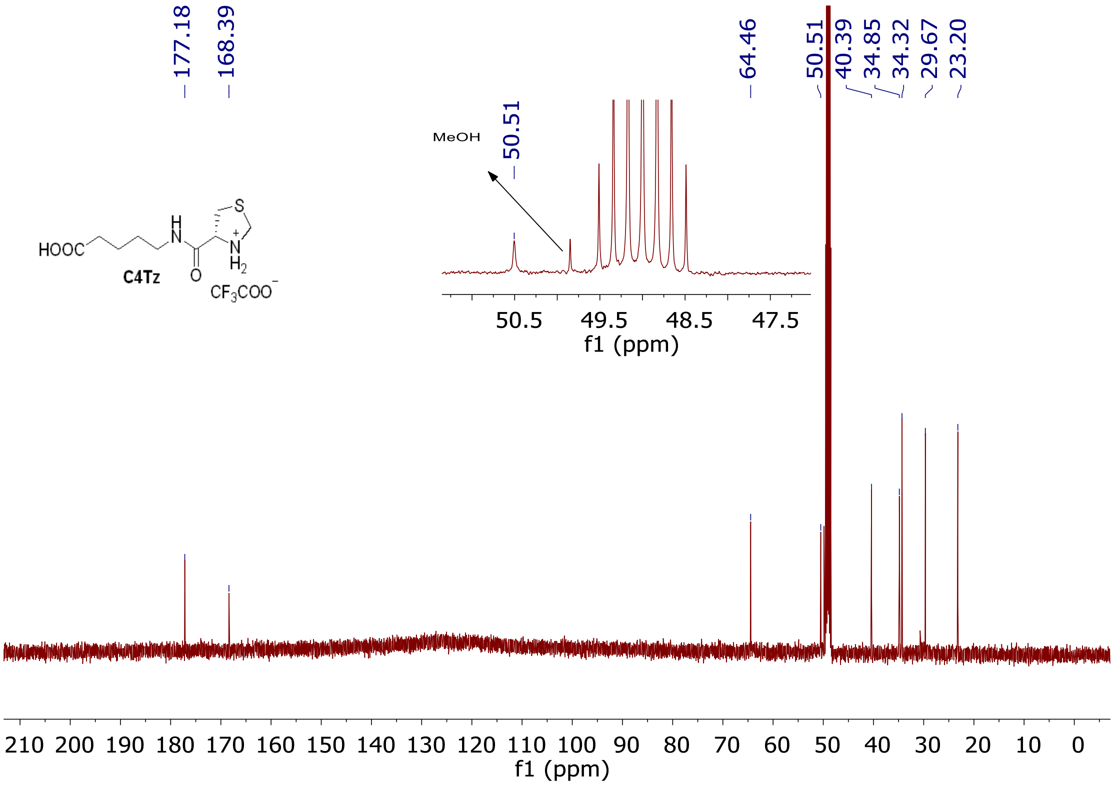

^{13}C NMR of C4Tz

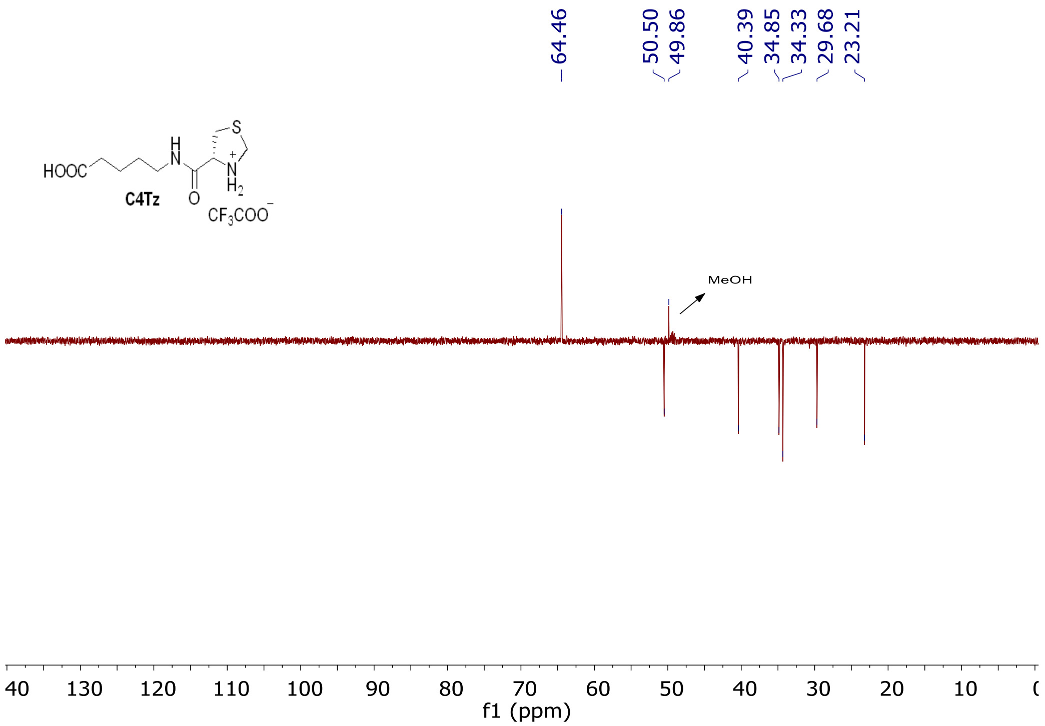

^{13}C DEPT-135 of C4Tz

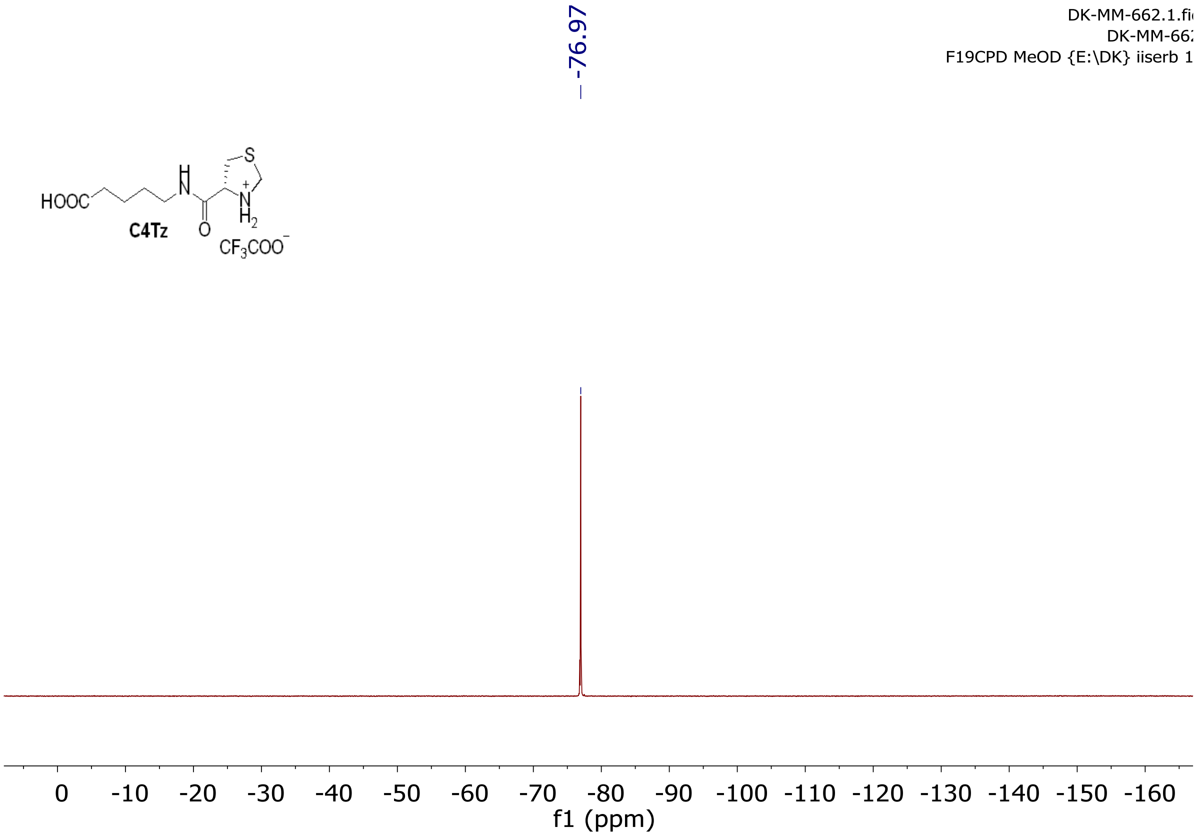

^{19}F NMR of C4Tz

Supplementary Fig. 91. NMR of C5Tz

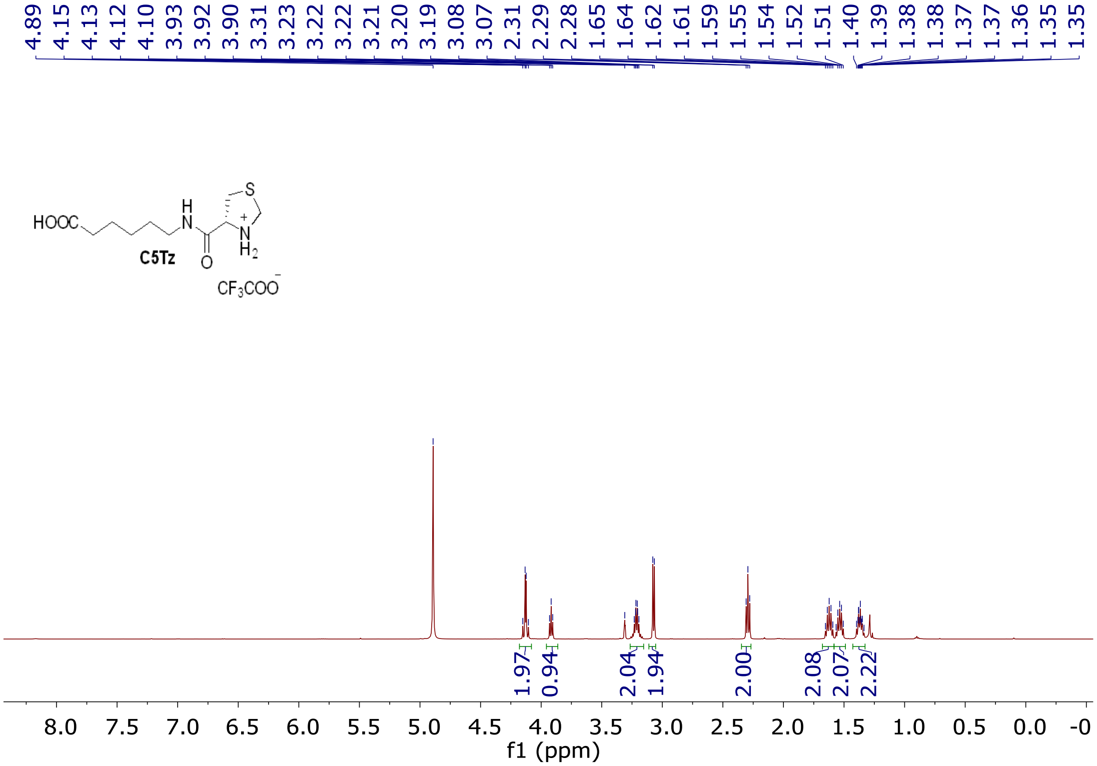

^{1}H NMR of C5Tz

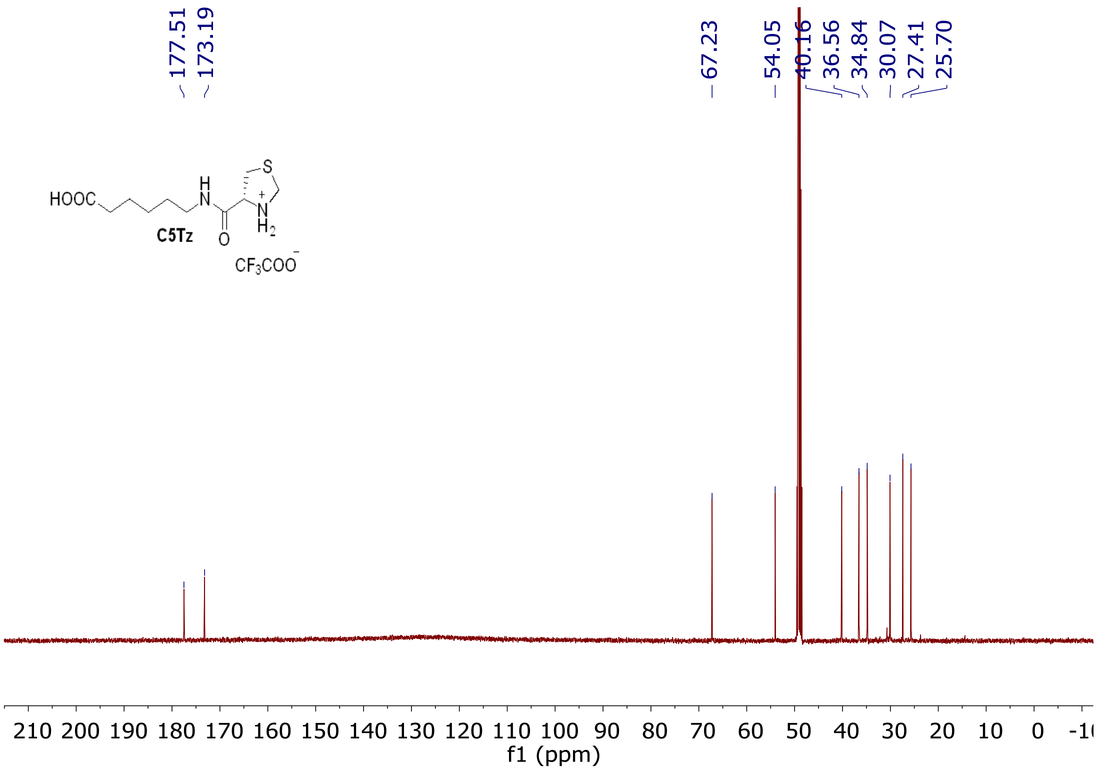

^{13}C NMR of C5Tz

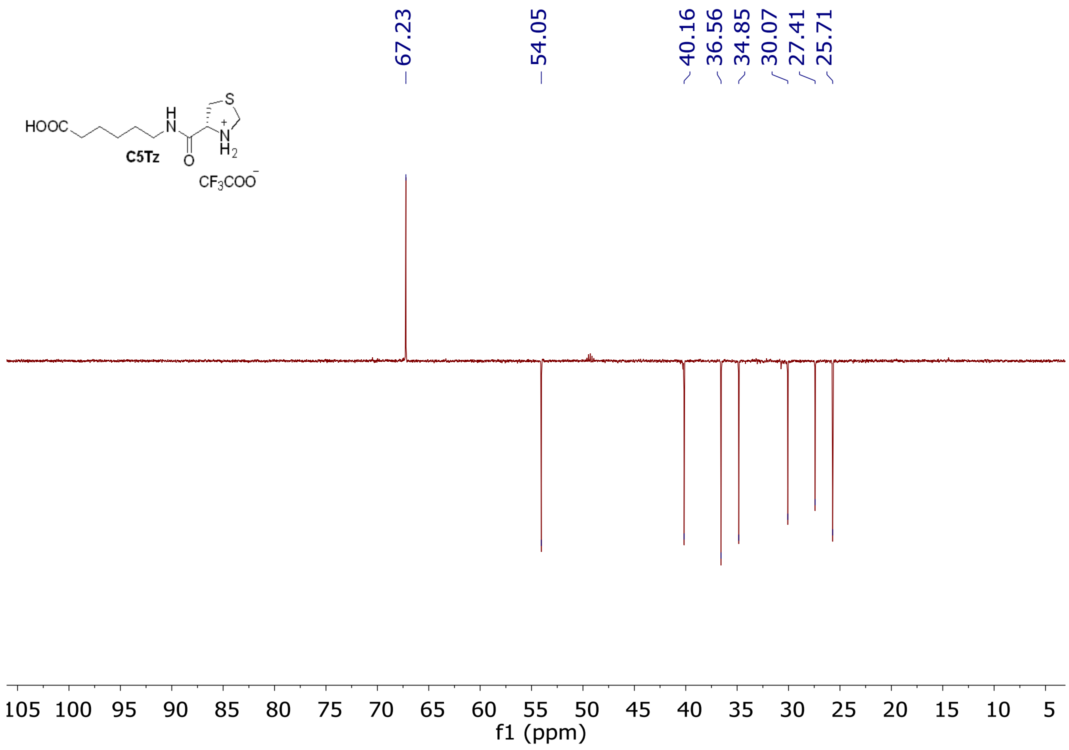

^{13}C DEPT-135 NMR of C5Tz

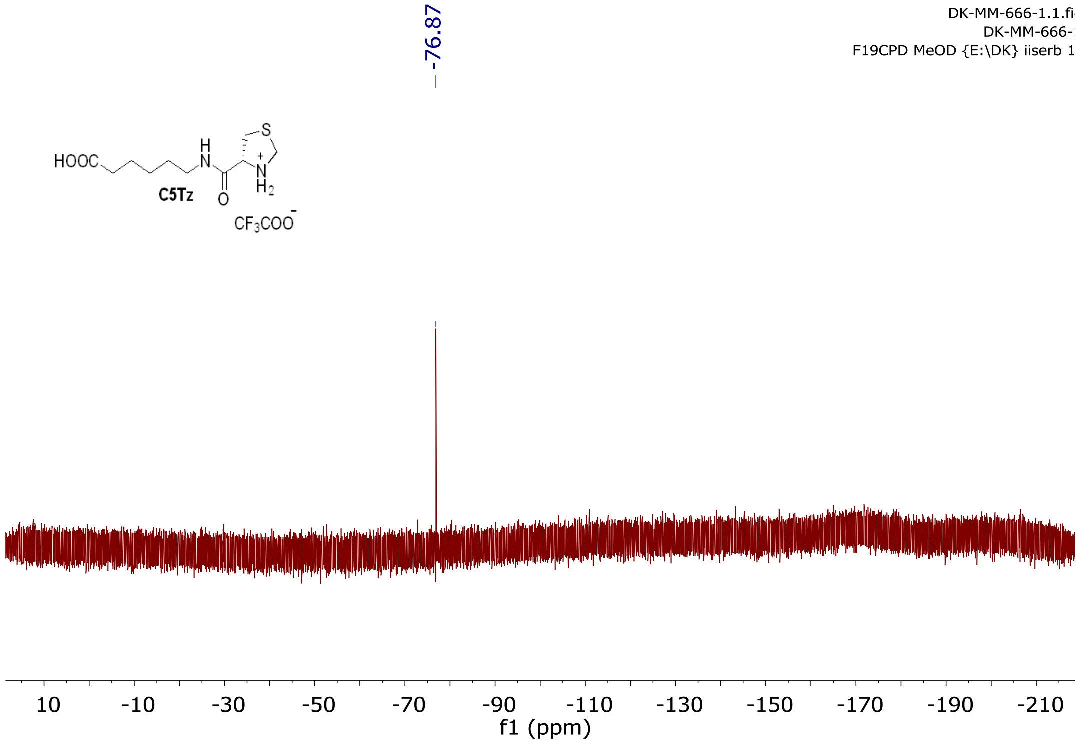

^{19}F NMR of C5Tz

Supplementary Fig. 92. NMR of CPO

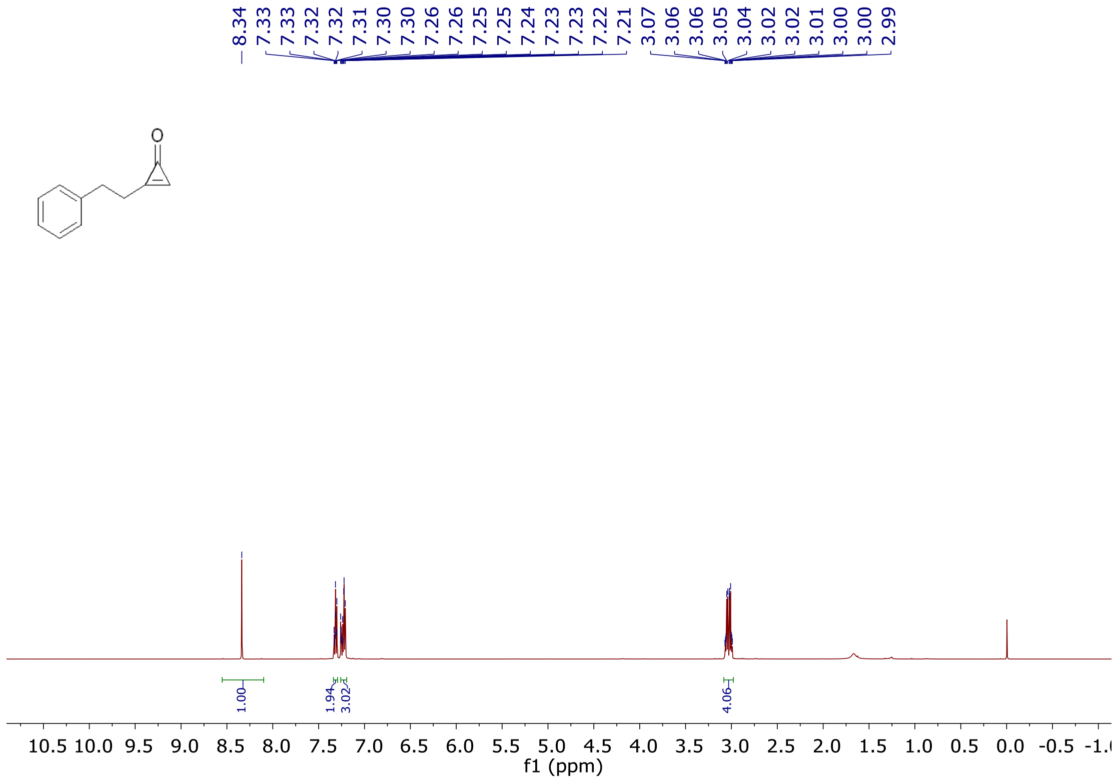

1

^{1}H NMR of CPO

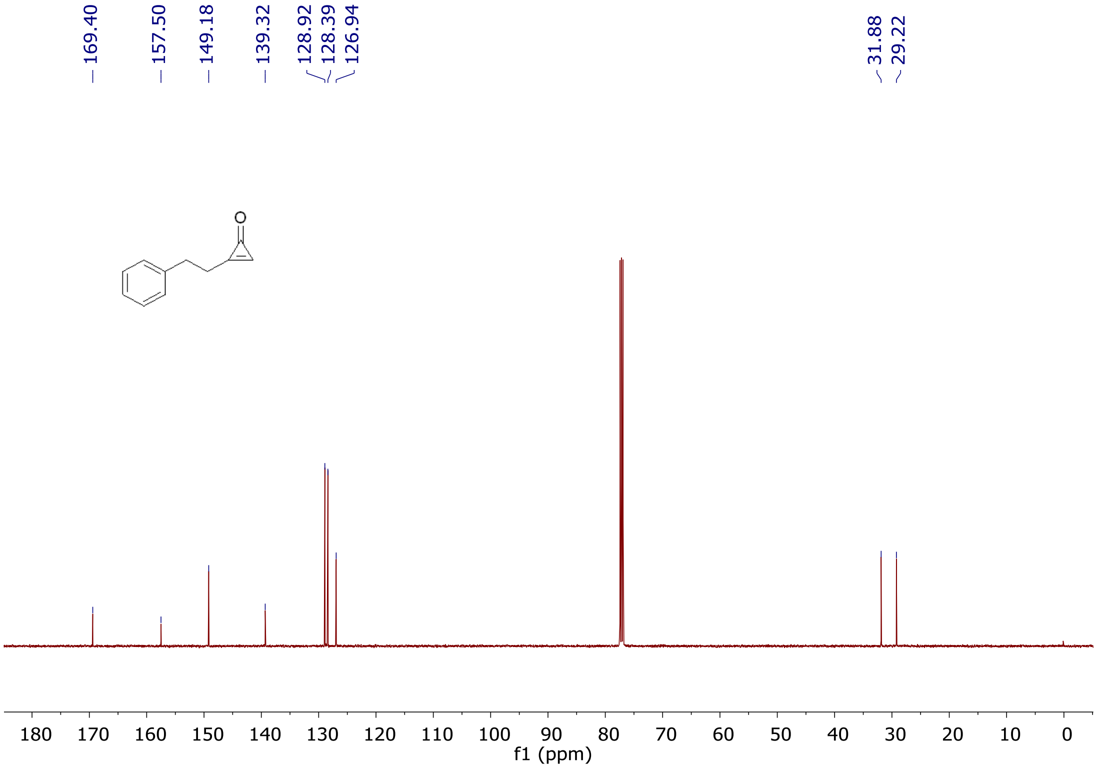

^{13}C NMR of CPO

19. Supplementary References

1 Castelo-Branco, F. S. *et al.* New hydrazides derivatives of isoniazid against Mycobacterium tuberculosis: Higher potency and lower hepatocytotoxicity. *Eur J Med Chem* **146**, 529-540, doi:10.1016/j.ejmech.2018.01.071 (2018).

2 Nizalapur, S. *et al.* Synthesis and biological evaluation of N-naphthoyl-phenylglyoxamide-based small molecular antimicrobial peptide mimics as novel antimicrobial agents and biofilm inhibitors. *Org Biomol Chem* **14**, 3623-3637, doi:10.1039/c6ob00298f (2016).

3 Turhanen, P. A. Synthesis of Triple-Bond-Containing 1-Hydroxy-1,1-bisphosphonic Acid Derivatives To Be Used as Precursors in "Click" Chemistry: Two Examples. *J Org Chem* **79**, 6330-6335, doi:10.1021/jo500831r (2014).

4 Wanat, P. *et al.* Ethynyl, 2-Propynyl, and 3-Butynyl C-Phosphonate Analogues of Nucleoside Di- and Triphosphates: Synthesis and Reactivity in CuAAC. *Org Lett* **17**, 3062-3065, doi:10.1021/acs.orglett.5b01346 (2015).

5 Sheldrick, G. M. Crystal structure refinement with SHELXL. *Acta Crystallogr C* **71**, 3-8, doi:10.1107/S2053229614024218 (2015).

6 Reddy, P. S. & Metanis, N. Small molecule diselenide additives for in vitro oxidative protein folding. *Chem Commun* **52**, 3336-3339, doi:10.1039/c5cc10451c (2016).

7 Tarozzi, A. *et al.* Combined inhibition of the EGFR/AKT pathways by a novel conjugate of quinazoline with isothiocyanate. *Eur J Med Chem* **117**, 283-291, doi:10.1016/j.ejmech.2016.04.002 (2016).

8 Istrate, A. *et al.* Platform for Orthogonal N-Cysteine-Specific Protein Modification Enabled by Cyclopropenone Reagents. *J Am Chem Soc*, doi:10.1021/jacs.2c02185 (2022).

9 Edelheit, O., Hanukoglu, A. & Hanukoglu, I. Simple and efficient site-directed mutagenesis using two single-primer reactions in parallel to generate mutants for protein structure-function studies. *BMC Biotechnol* **9**, doi.org/10.1186/1472-6750-9-61 (2009).

10 Gavins, G. C. *et al.* Live cell PNA labelling enables erasable fluorescence imaging of membrane proteins. *Nat Chem* **13**, 15-+, doi:10.1038/s41557-020-00584-z (2021).

11 Hong, V., Steinmetz, N. F., Manchester, M. & Finn, M. G. Labeling Live Cells by Copper-Catalyzed Alkyne-Azide Click Chemistry. *Bioconjugate Chem* **21**, 1912-1916, doi:10.1021/bc100272z (2010).

12 Gutmann, M. *et al.* Biocompatible Azide-Alkyne "Click" Reactions for Surface Decoration of Glyco-Engineered Cells. *Chembiochem* **17**, 866-875, doi:10.1002/cbic.201500582 (2016).

13 Fujiwara, K. *et al.* Crystal structure of lipoate-protein ligase a from *Escherichia coli* - Determination of the lipoic acid-binding site. *J Biol Chem* **280**, 33645-33651, doi:10.1074/jbc.M505010200 (2005).

14 Sastry, G. M., Adzhigirey, M., Day, T., Annabhimoju, R. & Sherman, W. Protein and ligand preparation: parameters, protocols, and influence on virtual screening enrichments. *J Comput Aid Mol Des* **27**, 221-234, doi:10.1007/s10822-013-9644-8 (2013).

15 Schrödinger Release 2022-3: Maestro, Schrödinger, LLC, New York, NY, 2021.

16 Roos, K. *et al.* OPLS3e: Extending Force Field Coverage for Drug-Like Small Molecules. *J Chem Theory Comput* **15**, 1863-1874, doi:10.1021/acs.jctc.8b01026 (2019).

17 Halgren, T. A. *et al.* Glide: A new approach for rapid, accurate docking and scoring. 2. Enrichment factors in database screening. *J Med Chem* **47**, 1750-1759, doi:10.1021/jm030644s (2004).

18 Genheden, S. & Ryde, U. The MM/PBSA and MM/GBSA methods to estimate ligand-binding affinities. *Expert Opin Drug Dis* **10**, 449-461, doi:10.1517/17460441.2015.1032936 (2015).

19 Fujiwara, K. *et al.* Global Conformational Change Associated with the Two-step Reaction Catalyzed by *Escherichia coli* Lipoate-Protein Ligase A. *J Biol Chem* **285**, 9971-9980, doi:10.1074/jbc.M109.078717 (2010).

20 Jbara, M. *et al.* Palladium prompted on-demand cysteine chemistry for the synthesis of challenging and uniquely modified proteins. *Nat Commun* **9**, doi:10.1038/s41467-018-05628-0 (2018).

20. Abbreviations

POI: Protein of interest

SDS-PAGE: Sodium dodecyl-sulfate polyacrylamide gel electrophoresis

DTT: Dithiothreitol

PMSF: Phenylmethyl sulfonyl fluoride

IPTG: Isopropyl β-d-1-thiogalactopyranoside

MBP: Maltose-binding protein

eGFP: Enhanced green fluorescent protein

LAP: LplA Acceptor Peptide

LplA: Lipoic acid ligase

TEV: Tobacco etch virus

Ni-NTA: Ni^{2+}/nitrilotriacetic acid

Cy-5 azide: Cyanine-5-azide

kDa: Kilo Dalton

PBS: Phosphate buffer saline

GSH: Glutathione

TBTA: *Tris*((1-benzyl-4-triazolyl) methyl) amine

THPTA: *Tris*-hydroxypropyltriazolylmethylamine

DMEM: Dulbecco's Modified Eagle Medium

21. Raw images of the gels reported in the supplementary figures. The data reported in the supplementary figures above is marked in red box.

Supplementary Fig. 33

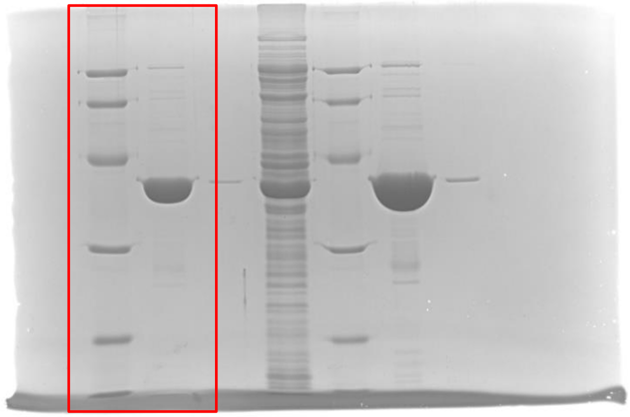

Supplementary Fig. 36

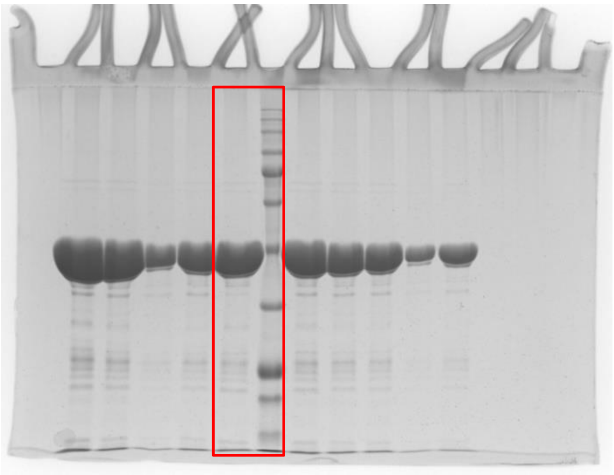

Supplementary Fig. 41

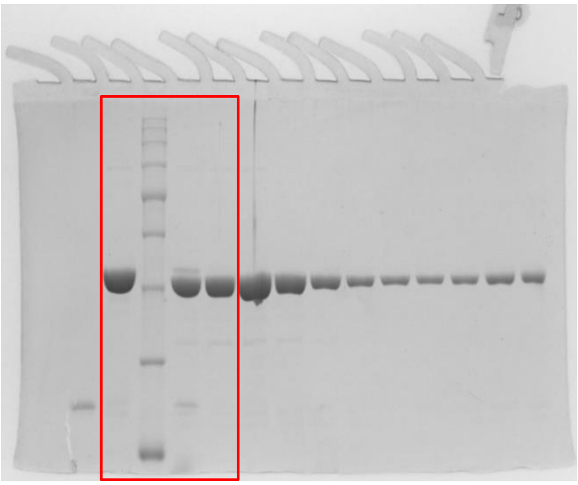

Supplementary Fig. 18 (Panel 1 from left)

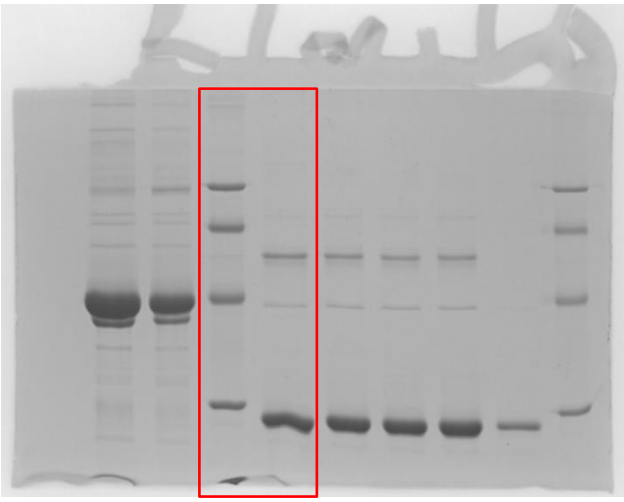

Supplementary Fig. 18 (Panel 2 from left)

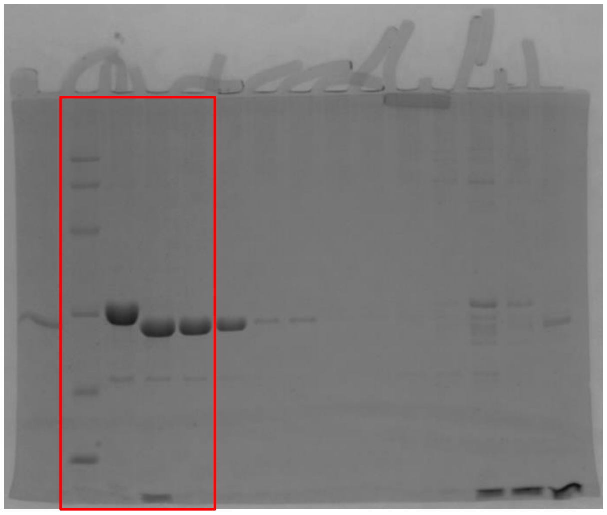

Supplementary Fig. 18 (Panel 3 from left)

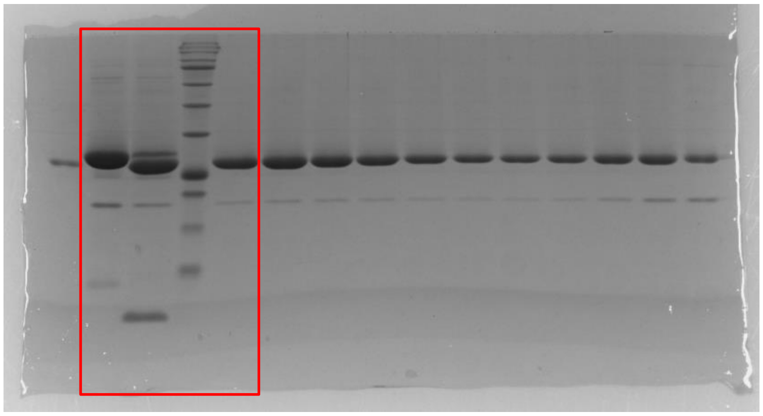

Supplementary Fig. 18 (Panel 4 from left)

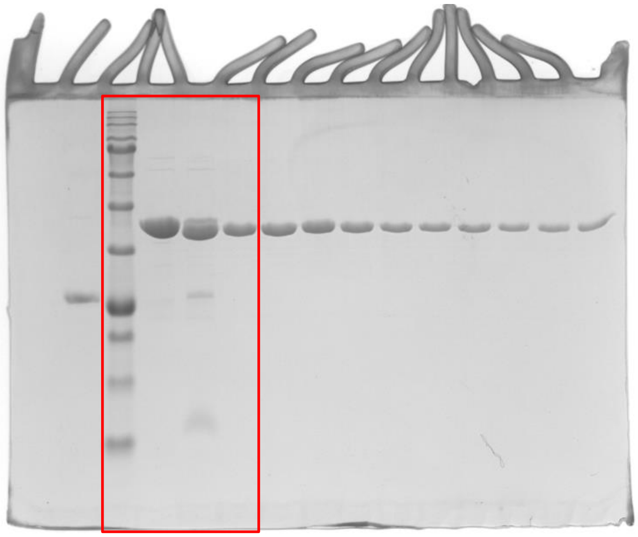

Supplementary Fig. 18 (Panel 5 from left)

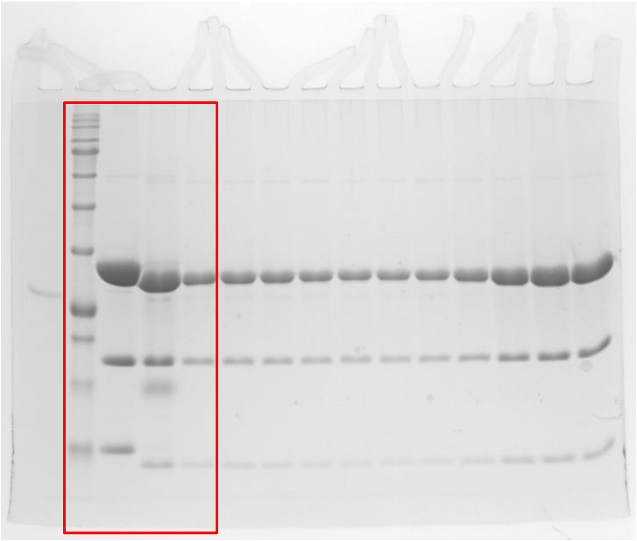

Supplementary Fig. 43

MBP-LAP

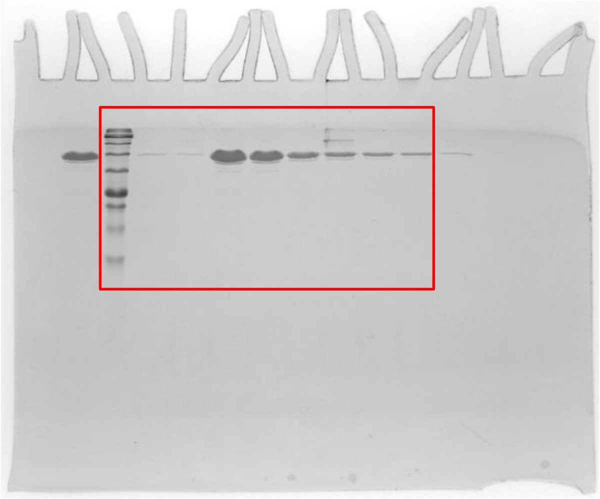

C7-**1D**

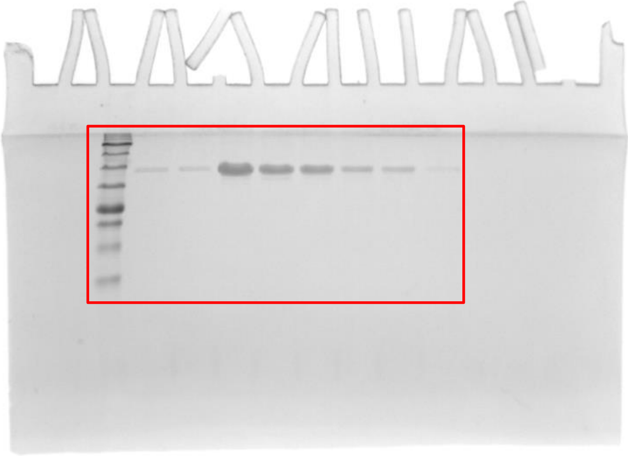

1D-C7-**1E**

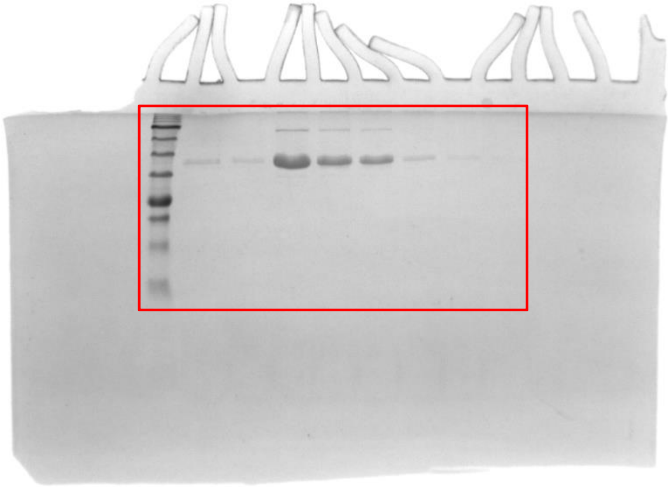

Supplement: Supplementary file 1 — Supplementary Information [file 41467_2024_45124_MOESM1_ESM.pdf]
