# Supplementary material for: Location-agnostic site-specific protein bioconjugation via Baylis Hillman adducts
Source: Nat Commun. 2024 Jan 29;15:859. doi: 10.1038/s41467-024-45124-2 (PMC10825175; doi:10.1038/s41467-024-45124-2)
Supplement: Supplementary file 3 — Reporting Summary [file 41467_2024_45124_MOESM3_ESM.pdf]

nature portfolio

Corresponding author(s): Dimpy Kalia

Last updated by author(s): Dec 19, 2023

Reporting Summary

Nature Portfolio wishes to improve the reproducibility of the work that we publish. This form provides structure for consistency and transparency in reporting. For further information on Nature Portfolio policies, see our Editorial Policies and the Editorial Policy Checklist.

Statistics

For all statistical analyses, confirm that the following items are present in the figure legend, table legend, main text, or Methods section.

n/a	Confirmed	
☐	☒	The exact sample size (*n*) for each experimental group/condition, given as a discrete number and unit of measurement
☒	☐	A statement on whether measurements were taken from distinct samples or whether the same sample was measured repeatedly
☒	☐	The statistical test(s) used AND whether they are one- or two-sided<br>*Only common tests should be described solely by name; describe more complex techniques in the Methods section.*
☒	☐	A description of all covariates tested
☒	☐	A description of any assumptions or corrections, such as tests of normality and adjustment for multiple comparisons
☐	☒	A full description of the statistical parameters including central tendency (e.g. means) or other basic estimates (e.g. regression coefficient) AND variation (e.g. standard deviation) or associated estimates of uncertainty (e.g. confidence intervals)
☒	☐	For null hypothesis testing, the test statistic (e.g. *F*, *t*, *r*) with confidence intervals, effect sizes, degrees of freedom and *P* value noted<br>*Give P values as exact values whenever suitable.*
☒	☐	For Bayesian analysis, information on the choice of priors and Markov chain Monte Carlo settings
☒	☐	For hierarchical and complex designs, identification of the appropriate level for tests and full reporting of outcomes
☒	☐	Estimates of effect sizes (e.g. Cohen's *d*, Pearson's *r*), indicating how they were calculated

Our web collection on statistics for biologists contains articles on many of the points above.

Software and code

Policy information about availability of computer code

Data collection	Agilent Open Lab (version A.01.11.138) was used for HPLC, Analyst software (version 1.6.3) was used for the MS data collection of protein conjugates, FV31S-SW was used for confocal microscopic imaging, Cary WinUV software (version 4,20(468)) was used for the UV-based kinetics experiments, Schrödinger Suite 2022-3 was used for docking, Agilent Mass Hunter Workstation for LC-MS/MS data collection
Data analysis	OriginPro 2021 (version 9.8.0.200) and MSExcel for making plots, MestReNova (version 10.0.2-15465) for processing NMR data, PeakView Software (Bio Tool Kit 1.0) for deconvoluting extracted ion chromatograms of protein conjugates, Agilent Bioconfirm Software 11.0 for the MS/MS analysis of digested protein conjugates, WinGX-Version 2020.1 software for XRD data analysis

For manuscripts utilizing custom algorithms or software that are central to the research but not yet described in published literature, software must be made available to editors and reviewers. We strongly encourage code deposition in a community repository (e.g. GitHub). See the Nature Portfolio guidelines for submitting code & software for further information.

Data

Policy information about availability of data

All manuscripts must include a data availability statement. This statement should provide the following information, where applicable:

- Accession codes, unique identifiers, or web links for publicly available datasets
- A description of any restrictions on data availability
- For clinical datasets or third party data, please ensure that the statement adheres to our policy

All data supporting the results and conclusions are available in the main text or the supplementary information. Crystallographic data for compounds 2A and 2C are available from the Cambridge Crystallographic Data Centre under CCDC deposition nos. 2259911 and 2259910, respectively.

Research involving human participants, their data, or biological material

Policy information about studies with human participants or human data. See also policy information about sex, gender (identity/presentation), and sexual orientation and race, ethnicity and racism.

Reporting on sex and gender	Not applicable
Reporting on race, ethnicity, or other socially relevant groupings	Not applicable
Population characteristics	Not applicable
Recruitment	Not applicable
Ethics oversight	Not applicable

Note that full information on the approval of the study protocol must also be provided in the manuscript.

Field-specific reporting

Please select the one below that is the best fit for your research. If you are not sure, read the appropriate sections before making your selection.

☒ Life sciences ☐ Behavioural & social sciences ☐ Ecological, evolutionary & environmental sciences

For a reference copy of the document with all sections, see nature.com/documents/nr-reporting-summary-flat.pdf

Life sciences study design

All studies must disclose on these points even when the disclosure is negative.

Sample size	No sample size calculations were performed but our N-Cys BHoPAL approach was employed to generate 17 protein conjugates, and the LAP TAG-mediated BHoPAL approach was successfully performed to generate 8 protein conjugates. Taken together, these large sample sizes establish the robustness of our methods.
Data exclusions	No data were excluded from the analyses.
Replication	All data are representative of at least two separate experiments. All attempts at replication were successful.
Randomization	Randomization was not relevant to our study because it involved synthetic chemistry and protein chemistry.
Blinding	Blinding was not relevant to our study because it involved synthetic chemistry and protein chemistry.

Reporting for specific materials, systems and methods

We require information from authors about some types of materials, experimental systems and methods used in many studies. Here, indicate whether each material, system or method listed is relevant to your study. If you are not sure if a list item applies to your research, read the appropriate section before selecting a response.

Materials & experimental systems

n/a	Involved in the study
☒	☐ Antibodies
☐	☒ Eukaryotic cell lines
☒	☐ Palaeontology and archaeology
☒	☐ Animals and other organisms
☒	☐ Clinical data
☒	☐ Dual use research of concern
☒	☐ Plants

Methods

n/a	Involved in the study
☒	☐ ChIP-seq
☒	☐ Flow cytometry
☒	☐ MRI-based neuroimaging

Eukaryotic cell lines

Policy information about cell lines and Sex and Gender in Research

Cell line source(s)	HEK293 cell lines were obtained from the Cell Repository at National Centre for Cell Science (NCCS), Ganeshkhind, Pune-411007, https://www.nccs.res.in/cellrepository
Authentication	Cell lines were used without authentication.
Mycoplasma contamination	All cell lines were tested for mycoplasma contamination routinely and were found to be not contaminated with mycoplasma.
Commonly misidentified lines (See ICLAC register)	No commonly misidentified cell lines were used in this study.
